# A General Strategy for C(sp^3^)─H Bond Etherification via Quinoline Derivative‐Mediated Electrolysis

**DOI:** 10.1002/advs.202416803

**Published:** 2025-04-26

**Authors:** Yousen Xu, Hao Wu, ChenXi Zhu, Minjun Tu, Lei Zhang

**Affiliations:** ^1^ Hangzhou Institute for Advanced Study University of Chinese Academy of Sciences 1 Sub‐lane Xiangshan Hangzhou 310024 China

**Keywords:** alcohols, C‐H bond, electrosynthesis, etherification, quinoline mediator

## Abstract

Electrooxidative coupling of C(sp^3^)─H bonds with nucleophiles offers an attractive method for constructing C─C and C─X bonds without sacrificial oxidants. However, the direct electrochemical approach requires the nucleophilic reagent to have a higher potential than the C(sp^3^)─H coupling partners, which restricts the substrate scope. In this study, a quinoline derivative is introduced as an electrochemical mediator, enabling efficient C─H bond etherification with reduced reliance on the electronic properties of substrates. The catalytic system demonstrates broad substrate compatibility, extending to C(sp^3^)─H coupling partners featuring a diverse range of C─H bonds, including tertiary benzylic C─H bonds and unactivated C(sp^3^)─H bonds. Mechanistic investigations confirm the role of the electrocatalyst in the hydrogen atom transfer (HAT) process. This method provides a versatile and efficient strategy for the late‐stage functionalization of bioactive molecules.

## Graphical abstract

A quinoline‐based mediator is introduced for the electrochemical etherification of C(sp^3^)─H bonds, enabling the process with minimal dependence on substrate electronic properties. The reaction demonstrates compatibility with various C─H bonds, including tertiary benzylic C─H bonds and unactivated C(sp^3^)─H bonds. This method offers a versatile and efficient strategy for the late‐stage functionalization of pharmaceuticals and bioactive molecules.

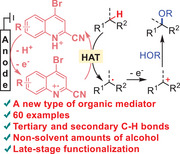

## 1 Introduction

**Scheme 1:**
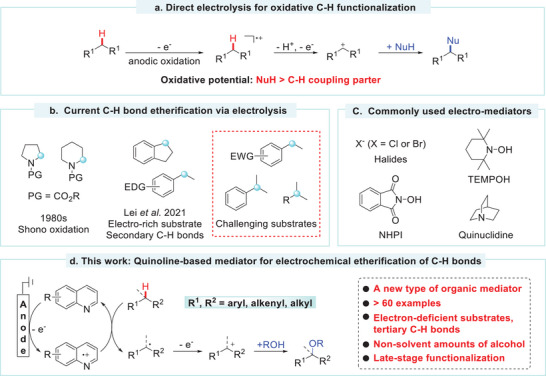
Electrochemical methods for C─H bond functionalization and etherification.

Electrochemical oxidative functionalization of C(sp^3^)─H bonds has emerged as a powerful method for constructing C─C, C─O, and C─N bonds with excellent atom economies.^[^ ^1^, ^2^, ^3^, ^4^, ^5^ ^]^ This technique offers a transition‐metal‐free approach for activating C(sp^3^)─H bonds without the use of sacrificial oxidants. The most commonly used methods involve direct electrolysis, in which the C(sp^3^)─H coupling partner is oxidized to form a radical‐cation intermediate for subsequent nucleophilic reactions (**Scheme** **1**a).^[^ ^6^, ^7^, ^8^ ^]^ According to the electrooxidation process, the C─H coupling partners must possess a lower potential than the nucleophiles to ensure selective anodic oxidation.^[^ ^9^ ^]^ It can be observed that the direct electrochemical approach often proves effective for electron‐poor nucleophiles,^[^ ^10^, ^11^, ^12^, ^13^, ^14^, ^15^ ^]^ such as trifluoroacetic acid (CF_3_COOH),^[^ ^15^, ^16^ ^]^ trimethylsilylmethylazide (TMSN_3_),^[^ ^12^ ^]^ and N,N‐dimethylformamide (DMF).^[^ ^17^ ^]^ However, the range of applicable substrates is significantly limited when utilizing nucleophiles with low oxidative potential.^[^ ^18^, ^19^, ^20^, ^21^ ^]^ In particular, the etherification of C(sp^3^)─H bonds presents a challenge when the C─H coupling partners exhibit a higher oxidative potential than alcohols. The electrochemical methoxylation of protected cyclic amines, known as Shono oxidation,^[^ ^22^, ^23^ ^]^ has been established since the early 1980s. Recent advancements in electrochemical methods have expanded the substrate scope to electron‐rich benzylic C(sp^3^)─H bonds (Scheme 1b).^[^ ^24^, ^25^, ^26^, ^27^, ^28^ ^]^ Nevertheless, the alkylation of electron‐deficient substrates persists as a significant challenge.^[^ ^29^, ^30^ ^]^ Additionally, the etherification of tertiary benzylic C─H bonds has not been systematically studied,^[^ ^31^ ^]^ even with the application of transition metal catalysts and photoredox catalysts. Considering the significance of dialkyl ethers, which are ubiquitous in natural products and bioactive molecules,^[^ ^32^, ^33^, ^34^, ^35^, ^36^ ^]^ the development of general etherification methods capable of addressing a diverse array of C(sp^3^)─H bonds is highly desirable.^[^ ^37^, ^38^, ^39^, ^40^, ^41^, ^42^ ^]^

Indirect electrolysis, which employs redox mediators to interact with the electrode surface, is expected to reduce the potential at the working electrode, modulate the reaction selectivity, and expand the substrate scope.^[^ ^9^, ^43^, ^44^, ^45^, ^46^, ^47^, ^48^, ^49^ ^]^ In C(sp^3^)─H bond functionalization, the mediated electrochemical method enables C─H bond activation via a hydrogen atom transfer (HAT) process, making the transformation less sensitive to the electronic effects of substrates.^[^ ^50^, ^51^, ^52^, ^53^, ^54^ ^]^ C─H coupling partners with a higher oxidative potential than the nucleophilic reagent can undergo effective functionalization. Despite the rapid advancement of electrosynthesis, the development of effective electrocatalysts—a crucial aspect of mediated electrolysis—has remained slow. The commonly used mediators include halides,^[^ ^55^ ^]^ 2,2,6,6‐tetramethylpiperidin‐1‐oxyl (TEMPO),^[^ ^56^ ^]^ and N‐hydroxyphthalimide (NHPI)^[^ ^49^, ^51^ ^]^ (Scheme 1c). These mediators are frequently employed at relatively high loading, presumably due to their low reactivity and stability. New types of electro‐mediator development are expected to enhance the versatility of electrochemical synthesis. The radical cation of pyridine‐based heterocycles has been demonstrated as a robust HAT reagent.^[^ ^57^, ^58^ ^]^ Nevertheless, the potential application of these nitrogen‐containing heterocycles in electrocatalytic reactions remains unexplored. We hypothesized that pyridine‐based heterocycles may undergo anodic oxidation to generate radical cations, which could promote C─H bond activation via the HAT process.

In this study, we disclosed a quinoline‐based redox mediator for the selective and efficient etherification of C─H bonds (Scheme 1d). An electron‐rich quinoline derivative has been rationally designed to promote the HAT process under electrolysis conditions. The reactions were conducted under constant current electrolysis with non‐solvent amounts of alcohol in an undivided cell. A diverse range of C(sp^3^)─H bonds, including tertiary benzylic C─H bonds, allyl C─H bonds, and unactivated C─H bonds, were suitable for alkylation using the current catalytic system. Mechanistic studies highlighted the pivotal role of the quinoline‐based electrocatalyst for C(sp^3^)─H bond etherification.

## 2 Results and Discussion

**Figure 1:**
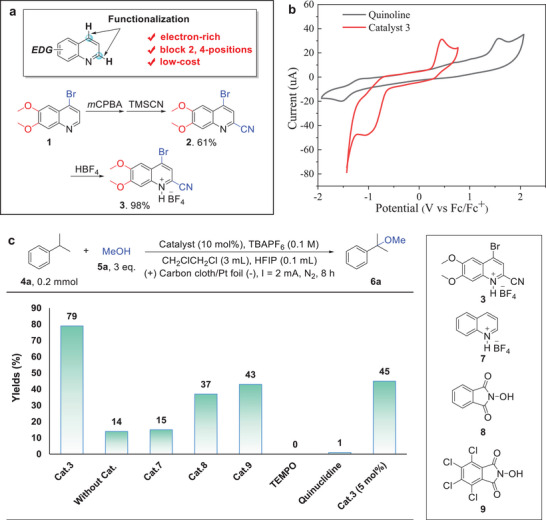
a) Design and preparation of quinoline‐based electrocatalysts. b) Cyclic voltammetry curves of **3** and quinoline. CV measurements were performed in CH_3_CN (3 mm), using TBAPF_6_ as the electrolyte, glassy carbon electrode as the working electrode, Pt wire as the counter electrode, and Ag/AgCl as the reference electrode. Scan rate: 100 mV s^−1^. c). C─H bond etherification of **4a** with different electro‐mediators. Reaction conditions: **4a** (0.2 mmol), **5a** (3 equiv.), catalyst (10 mol%), and *^n^* Bu_4_PF_6_ (0.1 m) in CH_2_ClCH_2_Cl under constant current electrolysis using carbon cloth (1.0 × 1.0 cm^2^) as the anode and Pt foil (1.0 × 1.0 cm^2^) as the cathode. Yields were determined by gas chromatography (GC) using mesitylene as an internal standard.

The ideal electro‐mediator for C(sp^3^)─H functionalization would possess two key characteristics: the capacity to function as a HAT reagent and display a relatively low oxidative potential. However, the inherent electron deficiency of pyridine‐based heterocycles results in a high oxidative potential, which presents a challenge to serve as an effective electro‐mediator. We postulated that quinoline compounds containing electron‐donating groups could be oxidized at a lower potential and serve as electrocatalysts for C─H bond functionalization. Notably, the 2,4‐position of quinoline is prone to radical reactions (**Figure** **1**a). The functionalization of these sites is anticipated to diversify the catalysts' structure and improve their stability. Furthermore, a straightforward method that utilizes readily available and cost‐effective starting materials to synthesize the electrocatalyst would enhance its practical value. Based on the above considerations, we selected commercially available 4‐bromo‐6,7‐dimethoxyquinoline **1** as the starting material, expected to have a low oxidative potential due to the presence of double methoxy groups. Compound **1** underwent sequential oxidation and cyanation reactions with 3‐chloroperoxybenzoic acid (*m*CPBA) and trimethylsilyl cyanide (TMSCN) to yield compound **2** with a 61% yield. Acidification of **2** with equivalent amounts of HBF_4_ afforded the product **3** in nearly quantitative yield. We recorded the cyclic voltammetry (CV) curve of **3** (Figure 1b). The oxidative potential is E_p/2_ = 0.43 V versus Fc^+^/Fc (Fc = ferrocene), which is significantly lower than unsubstituted quinoline (E_p/2_ = 1.45 V vs Fc^+^/Fc). The bond dissociation energy (BDE) of the N─H bond in **3** was determined to be 91.4 kcal mol^−1^ through a density functional theory (DFT) calculation. This value is slightly lower than that of the C─H bond in methanol (92 kcal mol^−1^) but higher than the BDE of the benzylic and allylic C‐H bonds (86–90 kcal mol^−1^).^[^ ^59^ ^]^

Compound **3** was evaluated for electrochemical etherification of C(sp^3^)─H bonds, using cumene **4a** as the model substrate with 3 equivalents of methanol in dichloromethane (DCE) (Figure 1c). The optimized method employed carbon cloth as the anode, Pt foil as the cathode, and tetrabutylammonium hexafluorophosphate (TBAPF_6_) as the electrolyte. 0.1 mL hexafluoroisopropanol (HFIP) was added as a co‐solvent to stabilize the quinoline radical cation. The reaction delivered the target product **6a** in a 79% yield under constant current electrolysis. The corresponding Faradic efficiency of the transformation is 53%. Notably, when the reaction was conducted without the electrocatalyst, the yield of **6a** was only 14%. The potential window of methanol (MeOH) is −1.1–1.3 V versus SCE,^[^ ^60^ ^]^ indicating that MeOH will be oxidized at a higher potential than 1.3 V. This potential is lower than that of cumene (1.58 V vs SCE, Figure S1, Supporting Information), which accounts for the low yield of the direct electrolysis. The electrolysis employing 10 mol% quinoline as an additive yielded 15% of **6a**, similar to the outcome observed without an electrocatalyst. The phenomenon may be attributed to the high potential of quinoline, which led to the direct electrolysis of cumene at the anode. Notably, N‐hydroxyphthalimide (NHPI) and N‐hydroxytetrachlorophthalimide (Cl_4_NHPI),^[^ ^49^ ^]^ established electrochemical mediators for C(sp^3^)─H functionalization, provided 37% and 43% yields, respectively. Additionally, the application of TEMPO and quinuclidine as mediators produced negligible products, which is likely attributable to their low activity for tertiary C(sp^3^)─H bond activation. Reducing the loading of **3** to 5 mol%, the catalytic reaction offered **6a** with a decreased yield of 45%. These observations demonstrate the efficiency of the quinoline‐based mediator for the electrochemical etherification of tertiary C─H bonds.

**Table 1:** Scope of electrochemical etherification of benzylic C─H bonds^a)^

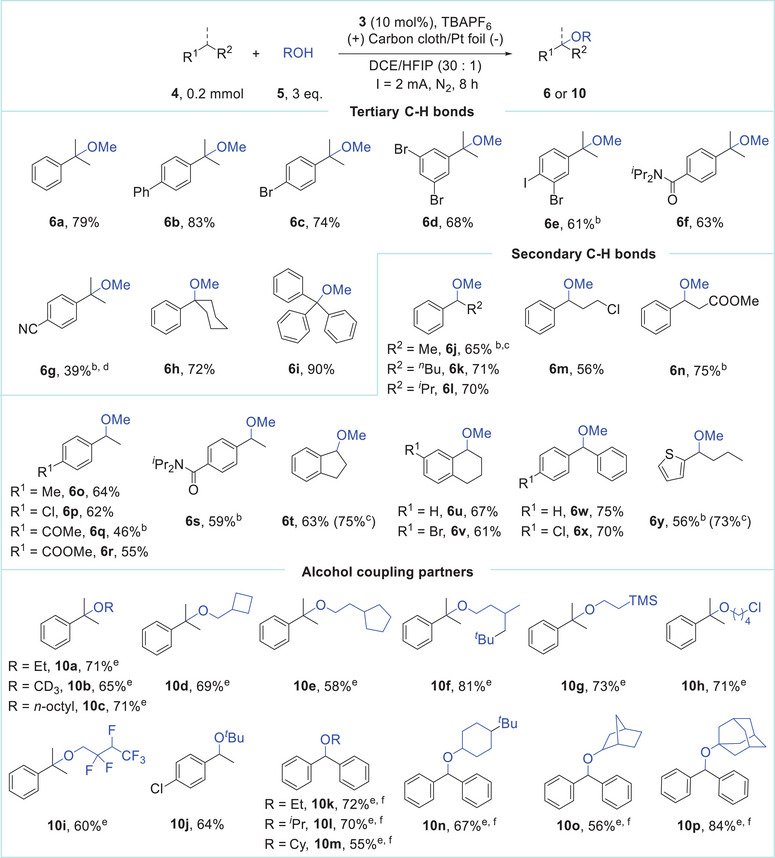

^a)^ Reaction conditions: **4** (0.2 mmol), **5** (0.6 mmol, 3 equiv.), **3** (10 mol%), and TBAPF_6_ (0.1 Mm) in DCE/HFIP (3.0 mL+0.1 mL) under constant current electrolysis (2 mA) using carbon cloth (1.0 × 1.0 cm^2^) as the anode and Pt foil (1.0 × 1.0 cm^2^) as the cathode at room temperature. Isolated yields;

^b)^ 5 equiv. of alcohols, t = 10 h;

^c)^ GC yield using mesitylene as an internal standard;

^d)^ 20 mol% of **3**;

^e)^ DCE (3.0 mL) was used as the solvent;

^f)^ t = 10 h.

With the established conditions in hand, we systematically explored the substrate scope of the electrochemical etherification of C─H bonds. The results are summarized in **Table** 1. The method demonstrated compatibility with a diverse range of substrates containing tertiary benzylic C(sp^3^)─H bonds (**6a‐i**). The catalytic system displayed excellent functional group tolerance, including substrates with electron‐withdrawing groups, such as bromides (**6c‐6e**), esters (**6n** & **6r**), and amides (**6f** & **6r**). It is noteworthy that iodide (**6e**), typically unstable under transition metal catalysis, was well‐tolerated. The substrate containing a cyanide group, challenging even in the Cu/NFSI catalytic system (yielding 10% of the corresponding product),^[^ ^38^ ^]^ exhibited improved performance with a 39% yield (**6g**) under the present conditions. Ethylbenzene (**6j**) and its derivatives bearing longer alkyl chains (**6k‐n**) afforded the desired products successfully. Notably, the etherification of 1‐ethyl‐4‐methylbenzene (**6o**) succeeded in selectively activating the secondary C─H bond. Indan (**6t**), tetralin (**6u**), and chromane (**6v**), common substructures that are found in pharmaceuticals, offered the corresponding products in good yields. Further experiments revealed the effective coupling of diarylmethanes (**6w** & **6x**) and triphenylmethane (**6i**) with methanol. The reaction also tolerated heterocycles, such as thiophene (**6y**). Moreover, the reaction can be readily scaled up, as evidenced by converting 5 mmol of cumene into the corresponding product **6a** with a yield of 70%.

**Table 2:** Scope of electrochemical etherification of allylic and unactivated C─H bonds^a)^.

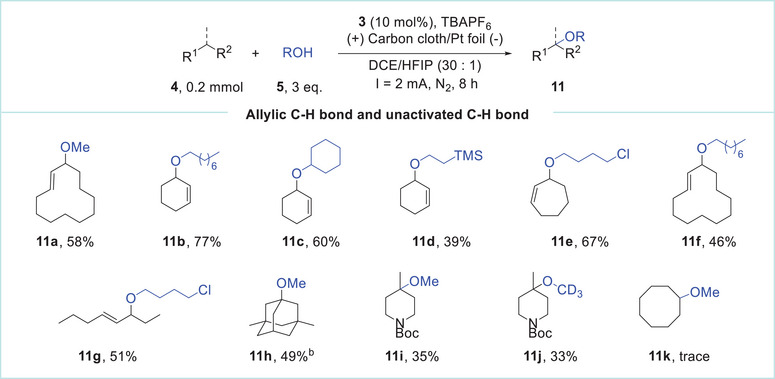

^a)^ Reaction conditions: **4** (0.2 mmol), **5** (0.6 mmol, 3 equiv.), **3** (10 mol%), and TBAPF_6_ (0.1 m) in DCE (3.0 ml) under constant current electrolysis (2 mA) using carbon cloth (1.0 × 1.0 cm^2^) as the anode and Pt foil (1.0 × 1.0 cm^2^) as the cathode at room temperature. t = 8 h. Isolated yields;

^b)^ **3** (50 mol %), 10 equiv. of alcohols, t = 24 h.

The reaction scope concerning the alcohol coupling partners was further investigated. Aliphatic alcohols with longer alkyl and cycloalkyl groups showed good reactivity with cumene (**10a**–**10f**). The use of CD_3_OD as the nucleophile provided the deuterated product **10b** in 65% yield. Primary alcohols containing various functional groups, such as trimethyl silyl, halogen, and ester groups, did not affect the etherification process (**10g**–**10i**). In addition, secondary and tertiary alcohols displayed efficient reactivity with diphenylmethane as coupling partners (**10l**–**10p**, **10j**). Unfortunately, benzyl alcohols proved incompatible in this catalytic system, likely due to the presence of the weak benzylic C(sp^3^)─H bond which is susceptible to hydrogen atom transfer processes.

Next, the coupling partners containing allylic C─H bonds and unactivated C(sp^3^)─H bonds were subjected to the mediated electrochemical etherification process (**Table** 2). Cyclododecene, cyclohexene, and cycloheptene displayed considerable reactivity with a range of alcohols, achieving moderate to high yields (**11a–11f**). The catalytic system is also applicable to linear allylic C─H bonds. However, several isomers were produced that are challenging to separate by column. For instance, the etherification of (*E*)‐4‐octene resulted in the formation of two isomers (3:1), with a combined yield of 51% (**11g**). Further experiments demonstrated the feasibility of etherification on unactivated C(sp^3^)─H bonds. The reaction of 1,3‐dimethyladamantane with methanol delivered a tertiary C(sp^3^)─H activation product in 49% yield (**11h**). Notably, the application of Boc‐protected 4‐methyl‐piperidine as a substrate unexpectedly resulted in tertiary C(sp^3^)─H etherification products (**11i** and **11j**), rather than the Shono oxidation products.

**Table 3:** Etherification of pharmaceuticals and bioactive molecules^a)^.

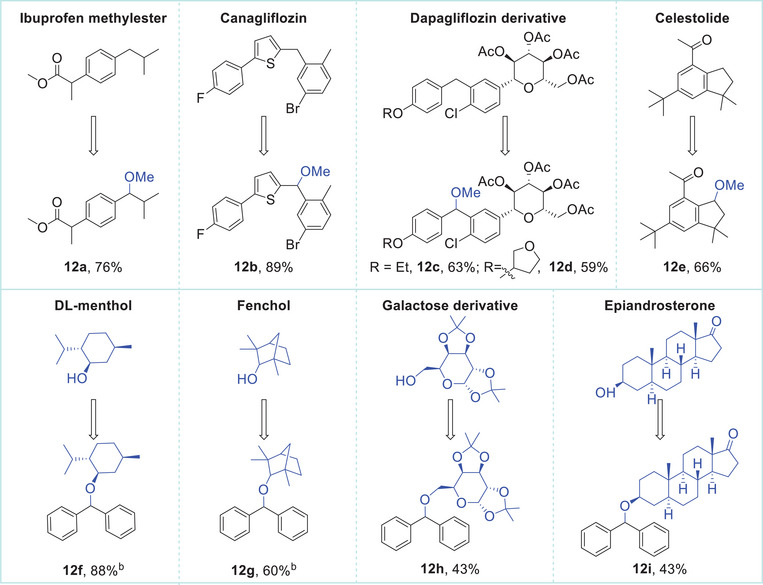

^a)^ Reaction conditions: **4** (0.2 mmol), **5** (0.6 mmol, 3 equiv.), **3** (10 mol%), and TBAPF_6_ (0.1 m) in DCE/HFIP (3.0 mL+0.1 mL) under constant current electrolysis (2 mA) using carbon cloth (1.0 × 1.0 cm^2^) as the anode and Pt foil (1.0 × 1.0 cm^2^) as the cathode at room temperature. Isolated yields;

^b)^ DCE (3.0 mL) was used as the solvent.

C─H bond methoxylation presents a valuable approach for altering the structure of drug molecules and modulating pharmacological properties. Consequently, this electrochemical approach was extended to the late‐stage functionalization of several pharmaceuticals and bioactive molecules (**Table** 3).^[^ ^3^ ^]^ The methylation of the nonsteroidal anti‐inflammatory drug ibuprofen afforded the corresponding product **12a** with a 76% yield. Several drugs used to lower blood sugar levels in type 2 diabetes, such as canagliflozin and dapagliflozin derivatives, underwent effective coupling with methanol (**12b**–**12d**). Additionally, the methylation of celestolide, a polycyclic musk fragrance, delivered **12e** in 66% yield. The application of non‐solvent amounts of alcohols as nucleophilic reagents allows for the transformation of complex molecules containing hydroxy groups, such as menthol, fenchol, and epiandrosterone. All of these molecules reacted smoothly with diphenylmethane (**12f**–**12i**).

**Figure 2:**
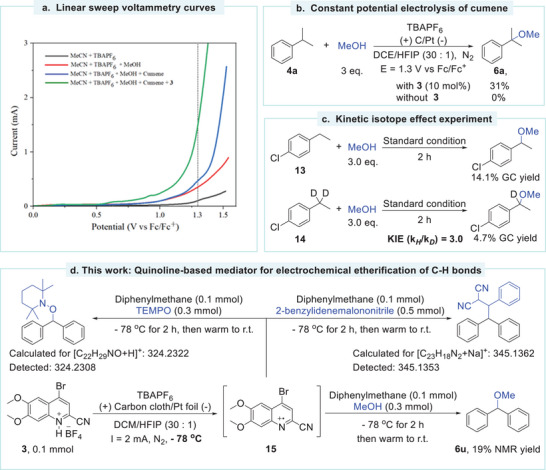
a) Linear sweep voltammetry curves of the reaction mixture and related. LSV measurements were performed using carbon cloth (1.0 × 1.0 cm^2^) as the working electrode, Pt foil (1.0 × 1.0 cm^2^) as the counter electrode, and Ag/AgCl as the reference electrode. Scan rate: 100 mV s^−1^. b) Constant potential electrolysis of cumene with and without **3**. c) Kinetic isotope effect experiment. d) Radical trapping experiments via the “cation pool” strategy.

A series of control experiments were performed to elucidate the mechanism of the C(sp^3^)─H bond etherification. Initially, constant potential electrolysis was conducted with the anodic potential set to 1.1 V versus Fc/Fc^+^. According to the CV curve (Figure 1b; Figure S1, Supporting Information), compound **3** can be oxidized to a radical cation under this potential, while cumene, the C(sp^3^)─H coupling partner, remains unoxidized. The reaction produced an 8% yield of **6a** in the presence of **3** after 8 h of electrolysis. The low yield was attributed to the slow electrolysis as only 0.8 mA current was observed during the reaction. Notably, no desired product was obtained in direct electrolysis without **3**. Since overpotential depends significantly on electrode materials and solvents, linear sweep voltammetry (LSV) was performed under standard electrolysis conditions (**Figure** **2**a). The results showed a notable current of 1.4 mA in the presence of **3** at a potential of 1.3 V versus Fc/Fc^+^ (green line). In comparison, cumene underwent minimal oxidation at this potential (blue line). The constant potential electrolysis at 1.3 V versus Fc/Fc^+^ using **3** as the catalyst delivered **6a** in a 31% yield (Figure 2b). In contrast, the electrolysis at the same potential without **3** did not yield product **6a**. Furthermore, we have closely monitored the anode potential during the constant current electrolysis (Figure S2, Supporting Information). At the onset of the reaction, the potential is 0.8 V versus Fc^+^/Fc. As the reaction progressed, the potential gradually increased probably due to the decomposition of the electrocatalyst. Notably, during the reaction, the potential remained below or close to the oxidative potential of cumene. These observations underscore the crucial role of **3** as an electrocatalyst and suggest that the reaction involves the oxidation of **3** at the anode rather than the C(sp^3^)─H coupling partners. The kinetic isotope effect (KIE) experiment was measured by parallel experiments using 4‐chloroethylbenzene **13** or isotope‐labeled 4‐chloroethylbenzene **14** as substrates (Figure 2c). The GC analysis of this reaction yielded a KIE value (k_H_/k_D_) of 3.0, which indicated that the C─H activation is the rate‐determining step (RDS).

As initially proposed, the electrochemical etherification likely involves the formation of a radical intermediate via the HAT process. We attempted to validate the HAT process between quinoline radical cation and C─H bonds. Yoshida et. al. developed a “cation pool” strategy to stabilize aryl radical cations, which promoted the functionalization of aromatic compounds.^[^ ^61^, ^62^ ^]^ Inspired by their work, we designed a stoichiometric experiment where the “cation pool” strategy was employed to stabilize the quinoline radical cation. The experiment commenced with the electrolysis of **3** in the absence of C─H coupling partners at −78 °C, which is supposed to generate quinoline radical cation **15** (Figure 2d). Low‐temperature electrolysis allowed for the accumulation of intermediate **15**. We employed TEMPO and 2‐benzylidenemalononitrile as radical trapping agents under the “cation pool” experiment conditions. Both the expected products were detected by high‐resolution mass spectrometry (HR‐MS) (Figure S3, Supporting Information). The TEMPO adduct (HRMS‐ESI (m/z): calculated for [C_22_H_29_NO+H]^+^:324.2322) is confirmed at 324.2308 and the 2‐benzylidenemalononitrile adduct (HRMS‐ESI (m/z): calculated for [C_23_H_18_N_2_+Na]^+^:345.1362) is confirmed at 345.1353. Moreover, MeOH was also used as a reagent under the “cation pool” experiment conditions. Analysis of the resulting mixture by gas chromatography‐mass spectrometry (GC‐MS) and nuclear magnetic hydrogen spectroscopy (^1^H NMR) revealed the formation of product **6u** in a 19% NMR yield. Control experiments without the electrolysis of **3** but maintaining other conditions failed to produce **6u**. The results provide compelling evidence that the HAT process occurred between the quinoline radical cation and the C(sp^3^)─H bonds to deliver alkyl radicals. The generation of **6u** indicated that the alkyl radical could be further oxidized by **15** to form a carbocation. Additionally, electron paramagnetic resonance (EPR) experiments were performed to detect the presence of the quinoline radical cation, and a weak signal (g = 2.003) corresponding to the aryl radical cation^[^^63]^ was obtained due to its low stability (Figure S6, Supporting Information).

**Scheme 2:**
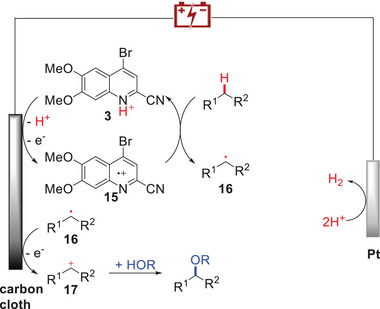
Proposed mechanism.

Based on the above experiments and previous literature, we proposed a mechanism for the quinoline derivative‐mediated electrochemical etherification (**Scheme** **2**). The electrocatalyst **3** undergoes deprotonation and initial oxidation at the anode, generating aryl radical cation **15**. Subsequently, intermediate **15** abstracts a hydrogen atom from the C(sp^3^)─H coupling partner to produce the radical **16** and regenerates the electrocatalyst **3**. Radical **16** is further oxidized by either the quinoline radical cation or the anode to form the carboncation **17**. **17** reacts with alcohol to yield the target product. Simultaneously, two protons are reduced to generate hydrogen at the cathode.

## 3 Conclusion

In summary, a quinoline derivative‐mediated electrochemical etherification of C─H bonds was revealed. This study features an elaborately designed quinoline derivative as a mediator for C─H bond functionalization with minimal reliance on substrate electronic properties. The method demonstrated compatibility with diverse substrates, including tertiary benzylic C─H bonds, allylic C─H bonds, and unactivated C(sp^3^)─H bonds. The practicability of the catalytic system is showcased through the late‐stage functionalization of pharmaceuticals and bioactive molecules. Control experiments, including constant potential electrolysis and stoichiometric experiments using the “cation pool” strategy, supported the proposed mechanism involving electro‐oxidation of the mediator, hydrogen atom transfer, and subsequent reactions with alcohols. From a broader perspective, this study unveils a powerful and selective method for C─H bond functionalization via mediated electrolysis, with the potential for broader applications in organic synthesis. Further applications of the quinoline‐based electrocatalysts are underway in our group.

## Conflict of Interest

The authors declare no conflict of interest.

## Supporting information



Supporting Information

## Data Availability

The data that support the findings of this study are available from the corresponding author upon reasonable request.
