# Supplementary material for: A General Strategy for C(sp3)─H Bond Etherification via Quinoline Derivative‐Mediated Electrolysis
Source: Adv Sci (Weinh). 2025 Apr 26;12(22):2416803. doi: 10.1002/advs.202416803 (PMC12165115; doi:10.1002/advs.202416803)
Supplement: Supplementary file 1 — Supporting Information [file ADVS-12-2416803-s001.pdf]

ADVANCED SCIENCE
Open Access

Supporting Information

for *Adv. Sci.*, DOI 10.1002/advs.202416803

A General Strategy for C(sp^3)—H Bond Etherification via Quinoline Derivative-Mediated Electrolysis

*Yousen Xu, Hao Wu, ChenXi Zhu, Minjun Tu and Lei Zhang**

Supporting Information

A General Strategy for C(sp^3)-H Bond Etherification via Quinoline Derivative-Mediated Electrolysis

*Yousen Xu, Hao Wu, Chenxi Zhu, Minjun Tu, and Lei Zhang**

Hangzhou Institute of Advanced Study, University of Chinese Academy of Sciences, 1 Sub-lane Xiangshan, Hangzhou 310024, China

zhanglei.chem@ucas.ac.cn

Table of Contents

1. General information……………………………………………S3
2. The preparation of quinoline-based catalyst……………..….S4
3. Reaction conditions optimization……………………...…S6
4. General procedure for C(sp^3)-H bonds etherification..........S9
5. Mechanistic studies……………………..………………..S35
6. References…………………………..………....………...S44
7. NMR spectra..S46

1. General Information

a. Materials

All manipulations were carried out in a nitrogen atmosphere glovebox or using standard Schlenk techniques unless noted otherwise. All reagents were purchased from commercial suppliers (Bidepharm, TCI, J&K, Macklin, Across, or Sinopharm). MeOH (J&K Chemical Reagent) and HFIP (Macklin Chemical Reagent) were distilled from calcium oxide under reduced pressure before use. All other reagents and solvents were purchased from commercial sources and used without purification.

b. Analytical Methods

NMR characterization was performed on 400 MHz spectrometers (101 MHz for ^{13}C NMR). ^{1}H NMR with TMS (δ = 0.0 ppm) or $CDCl_3$ (δ = 7.26 ppm) as the internal standard, ^{13}C NMR chemical shifts were referenced to the solvent resonance. Data for ^{1}H NMR are recorded as follows: chemical shift (δ, ppm), multiplicity (s = singlet, d = doublet, t = triplet, m = multiplet or unresolved, coupling constant (s) in Hz, integration). Data for ^{13}C NMR are reported in terms of chemical shift (δ, ppm). The GC measurements were conducted on Shimadzu Nexis GC-2030 with an FID detector. GC-MS measurements were conducted on Shimadzu GCMS-QP2020 NX. HRMS (ESI) measurements were conducted on Thermo Scientific Exactive Plus. Electrochemical experiments were conducted on Gamery 1010E potentiostat. EPR spectra were recorded using a Bruker EMX-plus X-band EPR spectrometer (Bruker Instruments, Billerica, MA).

2. The preparation of quinoline-based catalyst

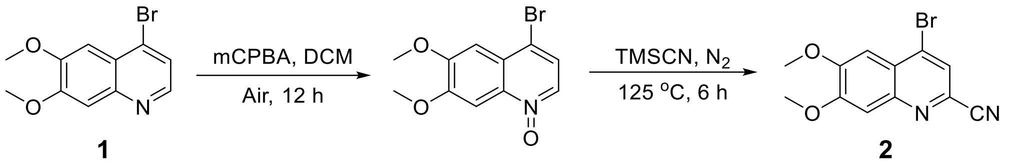

4-bromo-6,7-dimethoxyquinoline-2-carbonitrile (2): Under the air atmosphere, the 4-Bromo-6,7-dimethoxyquinoline **1** (3.028 g, 11.3 mmol) and DCM (100 ml) were added to a 250 mL three-necked flask equipped with a magnetic stir bar. The mixture was cooled to 0 °C. A solution of mCPBA (4.875 g, 22.6 mmol, 80 wt%) in DCM (50 ml) was added dropwise to the three-necked flask. The reaction stirred at room temperature overnight. Then excessive K_2SO_3 aqueous solution was used to quench the unreacted mCPBA. The aqueous phase was extracted with DCM (20 ml*3), and the organic layer was combined, washed with saturated NaCl (aq.), dried over anhydrous $MgSO_4$, and filtered. The solvent was removed under reduced pressure. The resulting residue was purified by flash column chromatography, eluting with EA to afford a white solid (2.79 g). This compound was further used to synthesize compound **2**. Under the nitrogen atmosphere, quinoline,4-bromo-6,7-dimethoxy-,1-oxide (2.79 g, 10.4 mmol), and TMSCN (3.97g, 40 mmol) were added to a 250 mL tube sealing. The reaction was stirred at 125 °C for 6 h. The black solid was obtained after cooling to room temperature, which was dissolved in DCM (20 mL). Excessive K_2CO_3 aqueous solution was added to the mixture. The aqueous phase was extracted with DCM (20 ml*3), and the organic layer was combined, washed with saturated NaCl (aq.), dried over anhydrous $MgSO_4$, and filtered. The solvent was removed under reduced pressure. The residue was further purified by flash column chromatography, eluting with PE/EA = 4: 1 to afford a white solid in 70% yield (2.044 g). ^1H NMR (400 MHz, $CDCl_3$) δ 7.8 (s, 1H, *aryl-H*), 7.4 (s, 1H, *aryl-H*), 7.4 (s, 1H, *aryl-H*), 4.1 (s, 3H, -OCH_3), 4.1 (s, 3H, -OCH_3). ^{13}C NMR (101 MHz, $CDCl_3$) δ 154.4 (*aryl-C*), 153.3 (*aryl-C*), 145.7 (*aryl-C*), 131.9 (*aryl-C*), 130.4 (*aryl-C*), 125.6 (*aryl-C*), 125.1 (*aryl-C*), 116.8 (*C*N), 108.3 (*aryl-C*), 104.0 (*aryl-C*),

56.6 (O*C*H_3), 56.6 (O*C*H_3). HRMS-ESI (*m/z*): calculated for [$C_{12}H_9BrN_2O_2$]: 291.9847; Found: 291.9843.

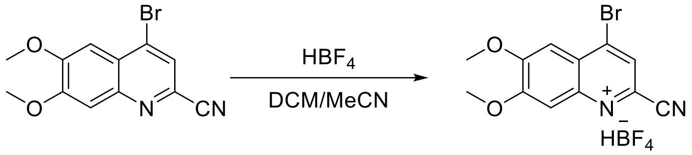

2 **3**

4-bromo-6,7-dimethoxyquinoline-2-carbonitrile tetrafluoroborate (3): 4-bromo-6,7-dimethoxyquinoline-2-carbonitrile **2** (876 mg, 3mmol), HBF_4 (790 mg, 9 mmol) DCM (25 mL) and MeCN (5 mL) were added to a 50 mL flask. The mixture was stirred at room temperature overnight. Then the solvent was removed under reduced pressure, resulting in a yellow solid with a 98% yield (1.12g). ^{1}H NMR (400 MHz, CD_3CN) δ 8.38 (s, 1H, *aryl-H*), 7.57 (s, 1H, *aryl-H*),), 7.41 (s, 1H, *aryl-H*),), 4.12 (d, *J* = 2.2 Hz, 6H, (OC*H*$_3$)$_2$). ^{13}C NMR (101 MHz, CD_3CN) δ 160.1 (*aryl-C*), 156.2 (*aryl-C*), 140.6 (*aryl-C*), 139.3 (*aryl-C*), 128.6 (*aryl-C*), 128.0 (*aryl-C*), 117.9 (*C*N), 112.3 (*aryl-C*), 105.6 (*aryl-C*), 101.2 (*aryl-C*), 58.1 (O*C*H_3), 57.6 (O*C*H_3). HRMS-ESI (*m/z*): calculated for [$C_{12}H_{10}BBrF_4N_2O_2$+Na]$^+$: 402.9853; Found: 402.9853.

3. Reaction Conditions Optimization

Table S1. Screening of the solvents.

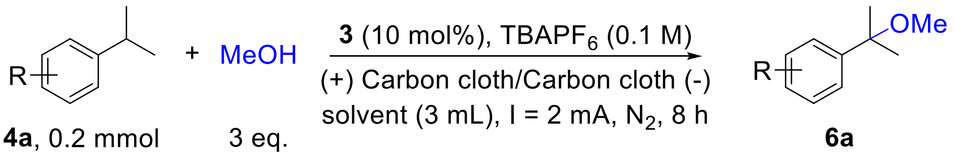

entry	solvent	yield(%)
1	DCM	45
2	DCE	65
3	DMF	6
4	MeCN	22
5	MeCN/PhCl (1 : 1)	7
6	DMSO	0

Reaction conditions: **4a** (0.2 mmol), MeOH (0.6 mmol), **3** (10 mol%), $TBAPF_6$ (0.1 M) in solvent (3 mL). Carbon cloth was used as the anode and the cathode under the N_2 atmosphere at room temperature for 8 h. The yield was determined by GC analysis using mesitylene as an internal standard.

Table S2. Screening of the electrolytes.

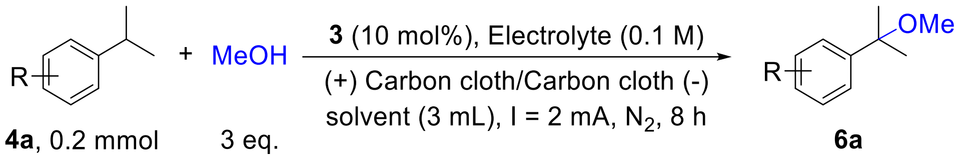

entry	electrolyte	yield(%)
1	$TBABF_4$	57
2	$TBAPF_6$	65
3	TBAI	0
4	TBABr	0
5	$LiBF_4$	trace
6	TBAOAc	trace

Reaction conditions: **4a** (0.2 mmol), MeOH (0.6 mmol), **3** (10 mol%), electrolyte (0.1 M), in DCE (3 mL) under the N_2 atmosphere at room temperature for 8 h. The yield was determined by GC analysis using mesitylene as an internal standard.

Table S3. Screening of the electrodes.

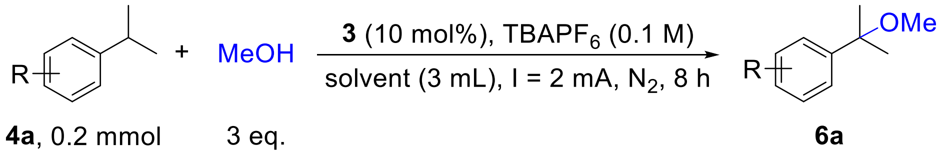

entry	electrode	yield(%)
1	(+) Carbon cloth/Carbon cloth (-)	64
2	(+) Graphite sheet/Carbon cloth (-)	27
3	(+) Carbon cloth/Pt plate (-)	73
4	(+) Graphite sheet/Pt plate (-)	36

Reaction conditions: **4a** (0.2 mmol), MeOH (0.6 mmol), **3** (10 mol%), $TBAPF_6$ (0.1 M), in DCE (3 mL) under the N_2 atmosphere at room temperature for 8 h. The yield was determined by GC analysis using mesitylene as an internal standard.

Table S4. Screening of the current.

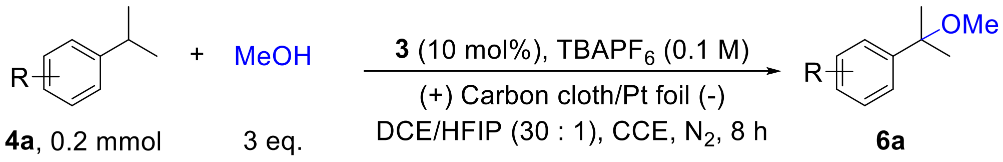

entry	current	yield(%)
1	1 mA	20
2	2 mA	79
3	3 mA	50
4	5 mA	15

Reaction conditions: **4a** (0.2 mmol), MeOH (0.6 mmol), **3** (10 mol%), $TBAPF_6$ (0.1 M), in DCE (3 mL) and HFIP (0.1 mL) under the N_2 atmosphere at room temperature for 8 h. The yield was determined by GC analysis using mesitylene as an internal standard.

Table S5. Screening of reaction time.

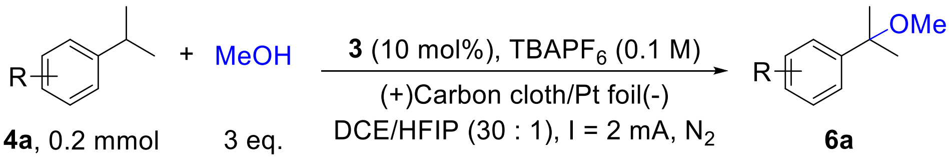

entry	time	yield(%)
1	8 h	79
2	10 h	71
3	12 h	63

Reaction conditions: **4a** (0.2 mmol), MeOH (0.6 mmol), **3** (10 mol%), $TBAPF_6$ (0.1 M), in DCE (3 mL) and HFIP (0.1 mL) under the N_2 atmosphere at room temperature. The yield was determined by GC analysis using mesitylene as an internal standard.

4. General procedure for C(sp^3)-H bonds etherification

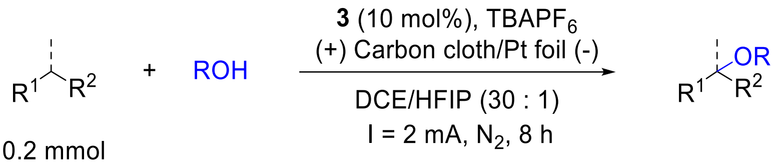

In the N_2-filled glovebox, catalyst **3** (7.6 mg, 10 mol%) and $TBAPF_6$ (116 mg, 0.1 M). substrates (0.2 mmol, 1 equiv.), alochols (0.6 mmol, 3 equiv.), DCE (3 mL) and HFIP (100 μL) were added to an oven-dried 10 mL tube equipped with a stir bar. Then the tube was sealed with a rubber plug equipped with carbon cloth (1.0×1.0 cm^2) and Pt foil (1.0×1.0 cm^2) electrodes. The reaction was performed under constant current electrolysis for 8 h. The resulting solution was diluted with ethyl acetate (5 mL) and filtered through celite and the filter liquor was concentrated under reduced pressure. Next, the residue was purified by chromatography or preparative thin-layer on silica gel, eluting with the mixture of ethyl acetate/hexane to give the corresponding products.

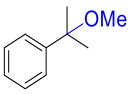

(1-Methoxy-1-methylethyl)benzene (6a) Colorless oil (23.8 mg, 79%). ^{1}H NMR (400 MHz, $CDCl_3$) δ = 7.36 (d, *J* = 8.3 Hz, 2H, *aryl-H*), 7.32 - 7.24 (m, 2H, *aryl-H*), 7.18 (t, *J* = 6.6 Hz, 1H, *aryl-H*), 3.01 (s, 3H, C-O-*CH*$_3$), 1.48 (s, 6H, *CH*$_3$-C-*CH*$_3$). ^{13}C NMR (101 MHz, $CDCl_3$) δ 146.0 (*aryl-C*), 128.3 (*aryl-C*), 126.9 (*aryl-C*), 125.9 (*aryl-C*), 76.8 (aryl-*C*-OCH_3), 50.7 (CH_3-C-O*C*H_3), 28.0 (*C*H_3-C-*C*H_3). These spectroscopic data correspond to the reported data[1].

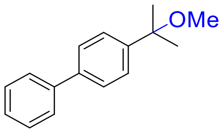

4-(1-Methoxy-1-methylethyl)-1,1′-biphenyl (6b) Colorless oil (37.1mg, 82%). ^{1}H NMR (400 MHz, $CDCl_3$) δ 7.65 - 7.55 (m, 4H, *aryl-H*), 7.52 - 7.40 (m, 4H, *aryl-H*), 7.35 (dd, *J* = 7.4, 1.3 Hz, 1H, *aryl-H*), 3.13 (s, 3H, C-O-C*H*$_3$), 1.58 (s, 6H, C(C*H*$_3$)$_2$). ^{13}C NMR (101 MHz, CDCl3) δ 145.1 (aryl-C), 141.0 (aryl-C), 139.8 (aryl-C),128.9 (aryl-C), 127.3 (*aryl-C*), 127.2 (*aryl-C*), 127.0 (*aryl-C*), 126.4 (*aryl-C*), 76.8 ((CH_3)$_2$*C*-OCH_3), 50.8 ((CH_3)$_2$-C-O*C*H_3), 28.1 ((*C*H_3)$_2$-C-OCH_3). These spectroscopic data correspond to the reported data[2].

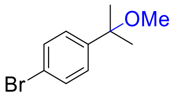

1-Bromo-4-(1-methoxy-1-methylethyl)benzene (6c) Pale yellow oil (33.2 mg, 73%). ^{1}H NMR (400 MHz, $CDCl_3$) δ = 7.38 (d, *J* = 8.5 Hz, 2H, *aryl-H*), 7.20 (d, *J* = 8.5 Hz, 2H, *aryl-H*), 2.98 (s, 3H, C-O-C*H*$_3$), 1.42 (s, 6H, C*H*$_3$-C-C*H*$_3$). ^{13}C NMR (101 MHz, $CDCl_3$) δ = 145.2 (*aryl-C*), 131.4 (*aryl-C*), 127.8 (*aryl-C*), 120.9 (*aryl-C*), 76.9 ((CH_3)$_2$*C*-OCH_3), 50.8 ((CH_3)$_2$-C-O*C*H_3), 27.9 ((*C*H_3)$_2$-C-OCH_3). These spectroscopic data correspond to the reported data[3].

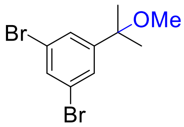

1,3-Dibromo-5-(1-methoxy-1-methylethyl)benzene (6d) Primrose yellow oil (42.0 mg, 68%). ^{1}H NMR (400 MHz, $CDCl_3$) δ = 7.55 (s, 1H, *aryl-H*), 7.47 (s, 2H, *aryl-H*), 3.08 (s, 3H, C-O-C*H*$_3$), 1.48 (s, 6H, C*H*$_3$-C-C*H*$_3$). ^{13}C NMR (101 MHz, $CDCl_3$) δ = 150.7 (*aryl-C*), 132.6 (*aryl-C*), 128.0 (*aryl-C*), 123.1 (*aryl-C*), 76.4 ((CH_3)$_2$*C*-OCH_3), 51.0 ((CH_3)$_2$-C-O*C*H_3), 27.8 ((*C*H_3)$_2$-C-OCH_3). HRMS-ESI (m/z): calculated for $[C_{10}H_{12}Br_2O+Na]^+$: 329.9153; found: 329.9152.

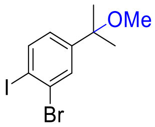

2-Bromo-1-iodine-4-(1-methoxy-1-methylethyl)benzene (6e) Colorless oil (43.0 mg, 61%). ^{1}H NMR (400 MHz, $CDCl_3$) δ = 7.81 (d, *J* = 8.2 Hz, 1H, *aryl-H*), 7.65 (s, 1H, *aryl-H*), 7.04 (d, *J* = 1.9 Hz, 1H, *aryl-H*), 3.07 (s, 3H, C-O-C*H*$_3$), 1.48 (s, 6H, C*H*$_3$-C-C*H*$_3$). ^{13}C NMR (101 MHz, $CDCl_3$) δ = 148.8 *(aryl-C)*, 140.2 *(aryl-C)*, 130.5 *(aryl-C)*, 129.9 *(aryl-C)*, 126.4 *(aryl-C)*, 99.1 *(aryl-C)*, 76.4 ((CH_3)$_2$*C*-OCH_3), 50.9 ((CH_3)$_2$-C-O*C*H_3), 27.8 ((*C*H_3)$_2$-C-OCH_3). HRMS-ESI (m/z): calculated for $[C_{10}H_{12}BrIO+Na]^+$: 376.9014; found: 376.9010.

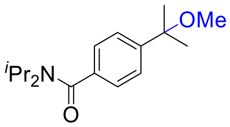

N, N-diisopropyl-4-(1-methoxy-1-methylethyl)benzene (6f) Yellow oil (35.2 mg, 63%). ^{1}H NMR (400 MHz, $CDCl_3$) δ = 7.66 (d, *J* = 8.4 Hz, 2H, *aryl-H*), 7.43 (d, *J* = 8.4 Hz, 2H, *aryl-H*), 4.06 (m, 2H, CH3-*CH*-CH_3) 3.17 (s, 3H, C-O-C*H*$_3$), 1.61 (s, 6H, C*H*$_3$-C-C*H*$_3$). 1.28 (d, 12H, (C*H*$_3$-CH-C*H*$_3$)$_2$). ^{13}C NMR (101 MHz, CDCl3) δ =171.5 (*C*O), 149.5 *(aryl-C)*, 136.2 *(aryl-C)*, 126.4 *(aryl-C)*, 125.7 *(aryl-C)*, 77.5 ((CH_3)$_2$*C*-OCH_3), 50.3 ((CH_3)$_2$-C-O*C*H_3), 34.0, 23.8, 20.7. HRMS (m/z): calculated for $[C_{17}H_{27}NO_2]$: 277.2042; found:277.2045.

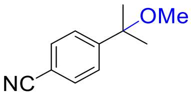

4-(1-Methoxy-1-methylethyl) benzonitrile (6g) Colorless oil (13.7 mg, 39%). ^{1}H NMR (400 MHz, $CDCl_3$) δ = 7.63 (d, *J* = 8.3 Hz, 2H, *aryl-H*), 7.52 (d, *J* = 8.3 Hz, 2H, *aryl-H*), 3.08 (s, 3H, OC*H*$_3$), 1.52 (s, 6H, C-(C*H*$_3$)$_2$). ^{13}C NMR (101 MHz, $CDCl_3$) δ = 152.1 (*aryl-C*), 132.3 (*aryl-C*), 126.7 (*aryl-C*), 119.0 (*aryl-C*), 110.9 (aryl-*C*N), 76.8 ((CH_3)$_2$*C*-OCH_3), 50.9 ((CH_3)$_2$-C-O*C*H_3), 27.8 ((*C*H_3)$_2$-C-OCH_3). These spectroscopic data correspond to the reported data[4].

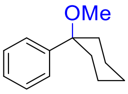

(1-Methoxycyclohexyl) benzene (6h) Colorless oil (27.0 mg, 72%). ^{1}H NMR (400 MHz, $CDCl_3$) δ = 7.39 (d, 2H, *aryl-H*), 7.32 (dd, *J* = 7.6, 7.6 Hz, 2H, *aryl-H*), 7.22 (t, J = 7.3 Hz, 1H, *aryl-H*), 2.94 (s, 3H, C-OC*H*$_3$), 1.99 (m, 2H, Cy-*H*), 1.72 - 1.62 (m, 5H, Cy-*H*), 1.55 (m, 2H, Cy-*H*), 1.23 (m, 1H, Cy-*H*). ^{13}C NMR (101 MHz, $CDCl_3$) δ = 146.1 (*aryl-C*), 128.3 (*aryl-C*), 126.9 (*aryl-C*), 126.2 (*aryl-C*), 77.4 (aryl-*C*-OCH_3), 49.7 (aryl-C-O*C*H_3), 35.5, 25.8, 22.1. These spectroscopic data correspond to the reported data[5].

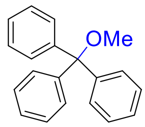

(methoxymethanetriyl)tribenzene (6i) White solid (49.1 mg, 90%) ^{1}H NMR (400 MHz, $CDCl_3$) δ = 7.49 (d, J = 7.3 Hz, 6H, *aryl-H*), 7.37 - 7.32 (m, 6H, *aryl-H*), 7.29 (d, J = 7.1 Hz, 3H, *aryl-H*), 3.11 (s, 3H, C-OC*H*$_3$). ^{13}C NMR (101 MHz, $CDCl_3$) δ = 144.1 (*aryl-C*), 128.9 (*aryl-C*), 127.9 (*aryl-C*), 127.0 (*aryl-C*), 87.2 ((*aryl-)*$_3$*-C*), 52.2 (C-O*C*H_3). These spectroscopic data correspond to the reported data[6].

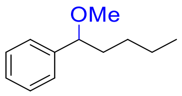

(1-Methoxypentyl) benzene (6k) Colorless oil (25.2 mg, 71%). ^{1}H NMR (400 MHz, $CDCl_3$) δ = 7.38 (m, 2H, *aryl-H*), 7.32 (m, 3H, *aryl-H*), 4.11 (t, *J* = 6.7 Hz, 1H, aryl-C*H*-CH_2), 3.24 (s, 3H, O-C*H*$_3$), 1.84 (m, 1H), 1.67 (m, 1H), 1.43-1.28 (m, 4H), 0.90 (t, *J* = 7.1 Hz, 3H, CH_2-C*H*$_3$). ^{13}C NMR (101 MHz, $CDCl_3$) δ = 142.7 (*aryl-C*), 128.4 (*aryl-C*), 127.5 (*aryl-C*), 126.8 (*aryl-C*), 84.3 (*C*H-O-CH_3), 56.8 (CH-O*C*H_3), 38.0, 28.1, 22.8, 14.1. These spectroscopic data correspond to the reported data[7].

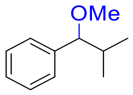

(1-Methoxy-2-methylpropyl)benzene (6l) Colorless oil (22.9 mg, 70%) ^{1}H NMR (400 MHz, $CDCl_3$) δ = 7.30 (t, *J* = 7.4 Hz, 1H, *aryl-H*), 7.25 - 7.18 (m, 4H, *aryl-H*), 3.73 (d, *J* = 7.3 Hz, 1H, C*H*-OCH_3), 3.17 (s, 3H, CH-OC*H₃*), 1.89 (dq, *J* = 13.8, 6.5 Hz, 1H, CH_3-C*H*-CH_3), 0.97 (d, *J* = 6.5 Hz, 3H, CH_3-CH-CH_3), 0.71 (d, *J* = 6.9 Hz, 3H, CH_3-CH-CH_3). ^{13}C NMR (101 MHz, $CDCl_3$) δ = 141.2 (*aryl-C*), 128.2 (*aryl-C*), 127.6 (*aryl-C*), 127.5 (*aryl-C*), 89.9 (*C*H-O-CH_3), 57.1 (CH-O-*C*H_3), 34.9 (CH_3-*C*H-CH_3), 19.1 (*C*H_3-CH-*C*H_3), 19.1 (*C*H_3-CH-*C*H_3). These spectroscopic data correspond to the reported data[7].

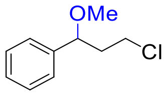

(3-Chloro-1-methoxypropyl)benzene (6m) Colorless oil (20.0 mg, 56%) ^{1}H NMR (400 MHz, $CDCl_3$) δ = 7.36 (t, *J* = 6.9 Hz, 1H, *aryl-H*), 7.31 - 7.29 (m, 4H, *aryl-H*), 4.37 (dd, *J* = 8.4, 4.9 Hz, 1H, C*H*-OCH_3), 3.75 - 3.67 (m, 1H, CH-CH_2), 3.54 - 3.48 (m, 1H, CH-CH_2), 3.24 (s, 3H, -OCH_3), 2.28 - 2.20 (m, 1H, CH_2-Cl), 2.06 - 1.97 (m, 1H, CH_2-Cl). ^{13}C NMR (101 MHz, $CDCl_3$) δ = 141.3 (*aryl-C*), 128.7 (*aryl-C*), 128.0 (*aryl-C*), 126.7 (*aryl-C*), 80.5 (*C*H-OCH_3), 56.9 (CH-O*C*H_3), 41.8 (CH-CH_2-*C*H_2-Cl), 41.1 (CH-*C*H_2-CH_2-Cl). These spectroscopic data correspond to the reported data[7].

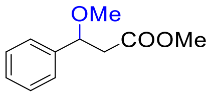

methyl 3-methoxy-3-phenylpropanoate (6n) Colorless oil (20.6 mg, 53%). ^{1}H NMR (400 MHz, $CDCl_3$) δ = 7.40 - 7.30 (m, 5H, *aryl-H*), 4.64 (dd, *J* = 9.6, 4.6 Hz, 1H, aryl-C*H*-OCH_3), 3.69 (s, 3H, OCH_3), 3.22 (s, 3H, CO-OCH_3), 2.81 (dd, *J* = 15.2, 9.2 Hz, 1H, CH_2-$COOCH_3$), 2.58 (dd, *J* = 15.3, 4.6 Hz, 1H, CH_2-$COOCH_3$). ^{13}C NMR (101 MHz, $CDCl_3$) δ = 171.6 (*C*$OOCH_3$), 140.7 (*aryl-C*), 128.7 (*aryl-C*), 128.2 (*aryl-C*), 126.7 (*aryl-C*), 80.2 (aryl-*C*H-OCH_3), 57.0 (CO-O*C*H_3), 51.9 (CH-O*C*H_3), 43.5 (*C*H_2-$COOCH_3$). These spectroscopic data correspond to the reported data[8].

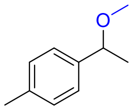

1-(1-(tert-butoxy)ethyl)-4-chlorobenzene (6o) Colorless oil (19.3 mg, 64%). ^{1}H NMR (400 MHz, CDCl$_3$) δ = 7.22 - 7.10 (m, 4H, *aryl-H*), 4.27 (q, *J* = 6.5 Hz, 1H, aryl-C*H*-OCH$_3$), 3.21 (s, 3H, OC*H$_3$*), 2.35 (s, 3H, aryl-C*H$_3$*), 1.43 (d, *J* = 6.4 Hz, 3H, CH-C*H$_3$*). ^{13}C NMR (101 MHz, CDCl$_3$) δ = 140.5(aryl-*C*), 137.3 (aryl-*C*), 129.2 (aryl-*C*), 126.3 (aryl-*C*), 79.6 (aryl-*C*H-CH$_3$), 56.5 (O-*C*H-(CH$_3$)$_3$), 24.1 (CH-*C*H$_3$), 21.3 (aryl-*C*H$_3$). These spectroscopic data correspond to the reported data[9].

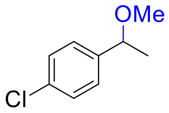

1-chloro-4-(1-methoxyethyl)benzene (6p) Colorless oil (21.0 mg, 62%). ^{1}H NMR (400 MHz, CDCl$_3$) δ = 7.32 (d, *J* = 8.5 Hz, 2H, *aryl-H*), 7.24 (d, *J* = 8.5 Hz, 2H, *aryl-H*), 4.27 (q, *J* = 6.5 Hz, 1H, C*H*-CH$_3$), 3.21 (s, 3H, -OC*H*$_3$), 1.41 (d, *J* = 6.5 Hz, 3H, CH-C*H*$_3$). ^{13}C NMR (101 MHz, CDCl$_3$) δ = 142.2 (*aryl-C*), 133.2 (*aryl-C*), 128.7 (*aryl-C*), 127.7 (*aryl-C*), 79.1 (*C*H-OCH$_3$), 56.6 (CH-O*C*H$_3$), 23.9 (CH-*C*H$_3$). These spectroscopic data correspond to the reported data[10].

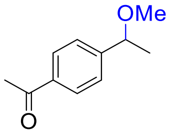

1-[4-(1-Methoxyethyl)phenyl]ethanone (6q) Colorless oil (16.4 mg, 46%) ^{1}H NMR (400 MHz, CDCl$_3$) δ = 7.94 (d, *J* = 6.5 Hz, 2H, *aryl-H*), 7.39 (d, *J* = 6.5 Hz, 2H, *aryl-H*), 4.44 - 4.16 (m, 1H, aryl-C*H*-OCH$_3$), 3.24 (s, 3H, CH-OC*H$_3$*), 2.59 (s, 3H, CO-C*H$_3$*), 1.42 (d, *J* = 3.0 Hz, 3H, aryl-CH-C*H$_3$*). ^{13}C NMR (101 MHz, CDCl$_3$) δ = 197.8 (*C*O), 149.3 (*aryl-C*), 136.6 (*aryl-C*), 128.7 (*aryl-C*), 126.4 (*aryl-C*), 79.3 (*C*H-OCH$_3$), 56.8 (CH-O*C*H$_3$), 26.7, (CO-*C*H$_3$) 23.8 (CH-*C*H$_3$). These spectroscopic data correspond to the reported data[11].

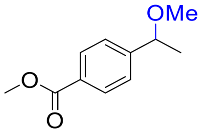

methyl 4-(1-methoxyethyl)benzoate (6r) Colorless oil (21.3 mg, 55%) ^{1}H NMR (400 MHz, $CDCl_3$) δ = 7.31 (d, *J* = 8.5 Hz, 2H, *aryl-H*), 7.06 (d, *J* = 8.5 Hz, 2H, *aryl-H*), 4.29 (q, *J* = 6.4 Hz, 1H, C*H*-CH_3), 3.22 (s, 3H, -OCH_3), 2.29 (s, 3H, COO-CH_3), 1.42 (d, *J* = 6.4 Hz, 3H, CH-CH_3). ^{13}C NMR (101 MHz, $CDCl_3$) δ = 169.6 (*C*O-OCH_3), 150.0 (*aryl-C*), 141.2 (*aryl-C*), 127.3 (*aryl-C*), 121.6 (*aryl-C*), 79.2 (*C*H-OCH_3), 56.6 (CH-OCH_3), 24.0 (CO-OCH_3), 21.3 (CH-CH_3). These spectroscopic data correspond to the reported data[12].

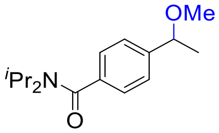

N, N-diisopropyl-4-(1-methoxyethyl)benzamide (6s) Yellow oil (31.0 mg, 59%). ^{1}H NMR (400 MHz, $CDCl_3$) δ = 7.69 (d, J = 8.8 Hz, 2H, *aryl-H*), 7.34 (d, J = 9.3 Hz, 2H, *aryl-H*), 4.64 (q, J = 6.4 Hz, 1H, C*H*-CH_3), 4.05 (m, 2H, CH3-*CH*-CH3), 3.30 (s, 3H, C-O-CH_3), 1.46 (d, J = 6.4 Hz, 3H, CH-CH_3). 1.28 (d, 12H, (C*H3*-CH-C*H3)*$_2$). ^{13}C NMR (101 MHz, $CDCl_3$) δ = 171.4 (*C*O-N), 144.9 (*aryl-C*), 136.1 (*aryl-C*), 127.8 (*aryl-C*), 125.6 (*aryl-C*), 77.4 (aryl-*C*H-OCH_3), 53.4 (aryl-CH-OCH_3), 50.1, 28.6, 20.7. HRMS (m/z): calculated for [$C_{16}H_{25}NO_2$]: 263.1885; found: 263.1880.

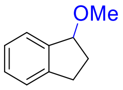

1-methoxy-2,3-dihydro-1H-indene (6t) Colorless oil (18.6 mg, 63%). ^{1}H NMR (400 MHz, $CDCl_3$) δ = 7.43 (d, *J* = 6.9 Hz, 1H, *aryl-H*), 7.29 (d, *J* = 8.3 Hz, 1H, *aryl-H*), 7.23 (dd, *J* = 3.8, 3.4 Hz, 2H, *aryl-H*), 4.85 (dd, *J* = 6.6, 3.9 Hz, 1H, *Cp-H*), 3.44 (s, 3H, -OCH_3), 3.15 - 3.06 (m, 1H, *Cp-H*), 2.89 - 2.80 (m, 1H, *Cp-H*), 2.41 - 2.31 (m, 1H, *Cp-*

H), 2.16 - 2.07 (m, 1H, *Cp-H*). ^{13}C NMR (101 MHz, $CDCl_3$) δ = 144.2 (*aryl-C*), 142.7 (*aryl-C*), 128.5 (*aryl-C*), 126.4 (*aryl-C*), 125.2 (*aryl-C*), 125.0 (*aryl-C*), 84.7 (*C*H-OCH_3), 56.2 (CH-O*C*H_3), 32.0 (CH-*C*H_2-CH_2), 30.3 (CH-CH_2-*C*H_2). These spectroscopic data correspond to the reported data[7].

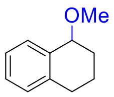

1-methoxy-1,2,3,4-tetrahydronaphthalene (6u) Colorless oil (21.7 mg, 67%). ^{1}H NMR (400 MHz, $CDCl_3$) δ = 7.36 (d, *J* = 7.3 Hz, 1H, *aryl-H*), 7.19 (dd, *J* = 6.3, 2.9 Hz, 2H, *aryl-H*), 7.10 (d, *J* = 6.6 Hz, 1H), 4.32 (t, *J* = 4.8 Hz, 1H), 3.45 (s, 3H), 2.84 - 2.81 (m, 1H), 2.77 - 2.73 (m, 1H), 2.09 - 2.02 (m, 2H), 1.93 - 1.85 (m, 1H), 1.81 - 1.69 (m, 1H). ^{13}C NMR (101 MHz, $CDCl_3$) δ = 137.6 (*aryl-C*), 136.7 (*aryl-C*), 129.5 (*aryl-C*), 129.1 (*aryl-C*), 127.7 (*aryl-C*), 125.8 (*aryl-C*), 76.9 (*C*H-OCH_3), 56.3 (CH-O*C*H_3), 29.2 (CH-*C*H_2-CH_2), 27.5 (CH-CH_2-*C*H_2), 18.8 (CH_2-CH_2-*C*H_2). These spectroscopic data correspond to the reported data[7].

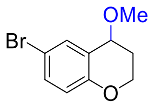

6-bromo-4-methoxychromane (6v) Light brown oil (29.0 mg, 61%). ^{1}H NMR (400 MHz, $CDCl_3$) δ = 7.40 (s, 1H, *aryl-H*), 7.30 (d, *J* = 8.8 Hz, 1H, *aryl-H*), 6.74 (d, *J* = 8.8 Hz, 1H, *aryl-H*), 4.27 (m, 1H, C*H*-OCH_3), 4.25 (m, 2H, O-C*H*$_2$-CH_2), 3.46 (s, 3H, -OC*H*$_3$), 2.19 - 2.09 (m, 1H), 2.09 - 1.98 (m, 1H). ^{13}C NMR (101 MHz, $CDCl_3$) δ = 154.0 (*aryl-C*), 133.0 (*aryl-C*), 132.6 (*aryl-C*), 123.9 (*aryl-C*), 119.0 (*aryl-C*), 112.0 (*aryl-C*), 71.6 (*C*H-OCH_3), 62.4 (-O-*C*H_2-CH_2), 56.1 (-*C*H-OCH_3), 26.9 (*C*H_2-CH_2). These spectroscopic data correspond to the reported data[7].

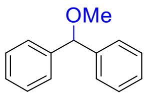

(methoxymethylene)dibenzene (6w) Colorless oil (29.7 mg, 75%). ^{1}H NMR (400 MHz, $CDCl_3$) δ = 7.41 - 7.35 (m, 8H, *aryl-H*), 7.31 - 7.26 (m, 2H, *aryl-H*), 5.29 (s, 1H, aryl-C*H*-aryl), 3.35 (s, 3H, CH-OCH_3). ^{13}C NMR (101 MHz, $CDCl_3$) δ = 142.2 (*aryl-C*), 128.5 (*aryl-C*), 127.6 (*aryl-C*), 127.1 (*aryl-C*), 85.6 (aryl-*C*H-aryl), 57.1 (CH-O*C*H_3). These spectroscopic data correspond to the reported data[7].

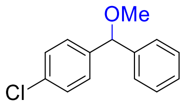

1-chloro-4-(methoxy(phenyl)methyl)benzene (6x) Colorless oil (35.3mg, 76%). ^{1}H NMR (400 MHz, $CDCl_3$) δ = 7.45 - 7.29 (m, 9H, *aryl-H*), 5.26 (s, 1H, aryl-C*H*-aryl), 3.40 (s, 3H, CH-OCH_3). ^{13}C NMR (101 MHz, $CDCl_3$) δ = 141.7 (*aryl-C*), 140.9 (*aryl-C*), 133.3 (*aryl-C*), 128.7 (*aryl-C*), 128.7 (*aryl-C*), 128.4 (*aryl-C*), 127.8 (*aryl-C*), 127.0 (*aryl-C*), 84.9 (aryl-*C*H-aryl), 57.1 (CH-O*C*H_3). These spectroscopic data correspond to the reported data[13].

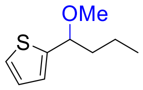

2-(1-methoxybutyl)thiophene (6y) Colorless oil (19.0 mg, 56%). ^{1}H NMR (400 MHz, $CDCl_3$) δ = 7.55 (d, J = 6.1 Hz, 1H, *aryl-H*), 7.25 (d, J = 5.4 Hz, 2H, *aryl-H*), 4.65 (t, J = 6.9 Hz, 1H, aryl-C*H*-OCH_3), 3.54 (s, 3H, CH-OCH_3), 2.23 - 2.12 (m, 1H, CH-CH_2), 2.03 - 1.95 (m, 1H, CH-CH_2), 1.72 - 1.64 (m, 1H, CH_2-CH_2), 1.58 (dd, J = 9.6, 7.3 Hz, 1H, CH_2-CH_2), 1.20 (t, J = 7.4 Hz, 3H, CH_2-CH_3). ^{13}C NMR (101 MHz, $CDCl_3$) δ = 146.5 (*aryl-C*), 126.4 (*aryl-C*), 125.4 (*aryl-C*), 124.9 (*aryl-C*), 79.3 (ar-*C*H-OCH_3), 56.5 (aryl-CH-O*C*H_3), 40.5, 19.2, 14.0. HRMS-ESI (m/z): calculated for [$C_9H_{14}OS$]: 170.0765; found: 170.0761.

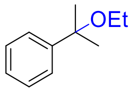

(1-Ethoxy-1-methylethyl)benzene (10a) Colorless oil (23.3 mg, 71%). ^{1}H NMR (400 MHz, $CDCl_3$) δ = 7.46 (dd, *J* = 8.4, 1.4 Hz, 2H, *aryl-H*), 7.40 - 7.35 (m, 2H, *aryl-H*), 7.29 (dd, *J* = 5.4, 1.8 Hz, 1H, *aryl-H*), 3.25 (q, *J* = 7.0 Hz, 2H, O-CH_2-CH_3), 1.57 (s, 6H, ($CH3$)$_2$-C-OEt), 1.19 (t, *J* = 7.0 Hz, 3H, O-CH_2-CH_3). ^{13}C NMR (101 MHz, $CDCl_3$) δ = 146.8 (*aryl-C*), 128.3 (*aryl-C*), 126.9 (*aryl-C*), 125.8 (*aryl-C*), 76.6 ((CH_3)$_2$-*C*-OEt), 58.3 (O-*C*H_2-CH_3), 28.7 ((*C*H_3)$_2$-C-OEt), 16.0 (O-CH_2-*C*H_3). These spectroscopic data correspond to the reported data[14].

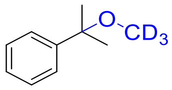

(2-(methoxy-d3)propan-2-yl)benzene (10b) Colorless oil (20.1 mg, 65%). ^{1}H NMR (400 MHz, $CDCl_3$) δ = 7.44 (d, *J* = 8.6 Hz, 2H, *aryl-H*), 7.37 (t, *J* = 7.6 Hz, 2H, *aryl-H*), 7.28 (t, *J* = 7.2 Hz, 1H, *aryl-H*), 1.6 (s, 6H, aryl-C-(CH_3)$_2$). ^{13}C NMR (101 MHz, $CDCl_3$) δ = 146.1 (*aryl-C*), 128.3 (*aryl-C*), 126.9 (*aryl-C*), 125.9 (*aryl-C*), 76.8 (aryl-*C*-(CH_3)$_2$), 28.1 (aryl-C-(*C*H_3)$_2$). These spectroscopic data correspond to the reported data[15].

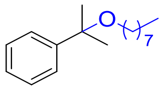

[1-Methyl-1-(octyloxy)ethyl]benzene (10c) Colorless oil (35.0 mg, 71%). ^{1}H NMR (400 MHz, $CDCl_3$) δ = 7.46 (d, *J* = 7.7 Hz, 2H, *aryl-H*), 7.36 (dd, *J* = 4.1, 4.1 Hz, 2H, *aryl-H*), 7.29 (t, *J* = 6.3 Hz, 1H, *aryl-H*), 3.17 (t, *J* = 6.0 Hz, 2H, O-CH_2-CH_2), 1.57 (s, 6H, (CH_3)$_2$-C-O), 1.29 (m, 12H), 0.91 (t, *J* = 6.1 Hz, 3H, CH_2-CH_3). ^{13}C NMR (101 MHz, $CDCl_3$) δ = 146.9 (*aryl-C*), 128.2 (*aryl-C*), 126.8 (*aryl-C*), 125.9 (*aryl-C*), 76.3 ((CH_3)$_2$-*C*-O), 62.9 (C-O-*C*H_2), 32.0 (O-*C*H_2), 30.6 (O-CH_2-*C*H_2), 29.6, 29.4, 28.6 ((*C*H_3)$_2$-C-O), 26.3, 22.8, 14.2 (CH_2-CH_2-*C*H_3). These spectroscopic data correspond to the reported data[16].

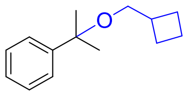

(2-(cyclobutylmethoxy)propan-2-yl)benzene (10d) Colorless oil (28.2 mg, 69%). ^{1}H NMR (400 MHz, $CDCl_3$) δ = 7.46 (d, *J* = 7.9 Hz, 2H, *aryl-H*), 7.37 (t, *J* = 7.6 Hz, 2H, *aryl-H*), 7.29 (d, *J* = 7.6 Hz, 1H, *aryl-H*), 3.15 (d, *J* = 7.0 Hz, 2H, O-C*H*2-CH), 2.55 (dt, *J* = 8.0, 7.8 Hz, 1H, CH_2-C*H*-CH_2), 2.11 - 2.03 (m, 2H, CBu-H), 1.97 – 1.83 (m, 2H, CBu-H), 1.70 - 1.64 (m, 2H, CBu-H), 1.56 (s, 6H, $(CH_3)_2$-C-O). ^{13}C NMR (101 MHz, $CDCl_3$) δ = 146.9 (*aryl-C*), 128.2 (*aryl-C*), 126.8 (*aryl-C*), 126.0 (*aryl-C*), 76.1 ($(CH_3)_2$-*C*-O), 67.6 (O-CH_2), 35.8 (Bu-*C*), 28.5 ($CH_3)_2$-*C*-O), 25.4 (Bu-*C*), 18.7 (Bu-*C*). HRMS-ESI (m/z): calculated for $[C_{14}H_{20}O+NH_4]^+$: 222.1858; found: 222.1857.

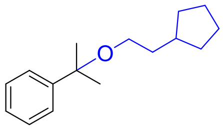

(2-(2-cyclopentylethoxy)propan-2-yl)benzene (10e) Colorless oil (26.9 mg, 58%). ^{1}H NMR (400 MHz, $CDCl_3$) δ = 7.48 - 7.44 (m, 2H, *aryl-H*), 7.40 - 7.34 (m, 2H, *aryl-H*), 7.28 (t, *J* = 7.3 Hz, 1H, *aryl-H*), 3.20 (t, *J* = 7.0 Hz, 2H, O-CH_2-CH_2), 1.85 (td, 2H, O-CH_2-CH_2-Cp), 1.77 - 1.71 (m, 2H, *Cp-H*), 1.69 - 1.47 (m, 15H). ^{13}C NMR (101 MHz, $CDCl_3$) δ = 146.8 (*aryl-C*), 128.1 (*aryl-C*), 126.7 (*aryl-C*), 125.7 (*aryl-C*), 76.3 (aryl-*C*-$(CH_3)_2$), 62.2 (OCH_2-CH_2), 36.9 (*Cp-C*), 36.8 (*Cp-C*), 32.8 (*Cp-C*), 32.7 (*Cp-C*), 28.5 (aryl-C-$(CH_3)_2$), 25.1 (*Cp-C*). HRMS-ESI (m/z): calculated for $[C_{16}H_{24}O+H]^+$: 233.1905; found: 233.1899.

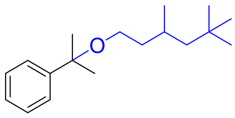

(2-((3,5,5-trimethylhexyl)oxy)propan-2-yl)benzene (10f) Colorless oil (42.4 mg, 81%). ^{1}H NMR (400 MHz, $CDCl_3$) δ = 7.46 (d, *J* = 7.9 Hz, 2H, *aryl-H*), 7.37 (t, *J* = 7.7 Hz, 2H, *aryl-H*), 7.29 (t, *J* = 7.9 Hz, 1H, *aryl-H*), 3.27 – 3.14 (m, 2H, O-CH_2-CH_2),

1.65 – 1.60 (m, 2H, O-CH$_2$-C*H$_2$*), 1.57 (s, 6H, aryl-C-(C*H$_3$*)$_2$), 1.46 – 1.39 (m, 1H), 1.23 (dd, *J* = 13.9, 3.6 Hz, 1H), 1.07-1.03 (dd, *J* = 13.9, 5.9 Hz, 1H), 0.91 (s, 9H, C-(C*H$_3$*)$_3$), 0.89 (d, *J* = 6.5 Hz, 3H, CH-C*H$_3$*). ^{13}C NMR (101 MHz, CDCl$_3$) δ = 147.0 (*aryl-C*), 128.2 (*aryl-C*), 126.8 (*aryl-C*), 125.8 (*aryl-C*), 76.4 (aryl-*C*-O), 61.3 (O-*C*H$_2$-CH$_2$), 51.5, 40.1, 30.1, 28.5 (aryl-C-(*C*H$_3$)$_2$),28.4, 26.5, 22.9. HRMS-ESI (m/z): calculated for [$C_{17}H_{28}O$+H] $^+$: 249.2218; found: 249.2219.

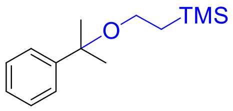

trimethyl(2-((2-phenylpropan-2-yl)oxy)ethyl)silane (10g) Light yellow oil (34.0 mg, 73%). ^{1}H NMR (400 MHz, CDCl$_3$) δ = 7.45 (d, *J* = 8.3 Hz, 2H, *aryl-H*), 7.40 - 7.35 (m, 2H, *aryl-H*), 7.27 (t, *J* = 7.3 Hz, 1H, *aryl-H*), 3.28 (t, *J* = 8.2 Hz, 2H, O-C*H$_2$*-CH$_2$), 1.57 (s, 6H, aryl-C-(C*H$_3$*)$_2$), 0.94 (t, *J* = 8.2 Hz, 2H, CH$_2$-C*H$_2$*-TMS), 0.00 (s, 9H, CH$_2$-Si(C*H$_3$*)$_3$). ^{13}C NMR (101 MHz, CDCl$_3$) δ = 147.0 (*aryl-C*), 128.2 (*aryl-C*), 126.8 (*aryl-C*), 125.8 (*aryl-C*), 76.5 (aryl-*C*-(CH$_3$)2), 59.7 (O-*C*H$_2$-CH$_2$), 28.7 (aryl-C-(*C*H$_3$)2), 19.1 (-O-CH$_2$-*C*H$_2$), 0.0 (Si-(*C*H$_3$)$_3$). HRMS-ESI (m/z): calculated for [$C_{14}H_{24}OSi$]: 236.1596; found: 236.1601.

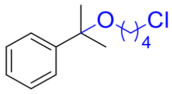

(2-(4-chlorobutoxy)propan-2-yl)benzene (10h) Colorless oil (32.1 mg, 71%). ^{1}H NMR (400 MHz, CDCl$_3$) δ = 7.43 (d, *J* = 8.0 Hz, 2H, *aryl-H*), 7.37 (t, *J* = 7.6 Hz, 2H, *aryl-H*), 7.28 (t, *J* = 7.8 Hz, 1H, *aryl-H*), 3.55 (t, *J* = 6.8 Hz, 2H, OC*H$_2$*-CH$_2$), 3.20 (t, *J* = 6.3 Hz, 2H, C*H$_2$*-Cl), 1.91 - 1.82 (m, 2H, OCH$_2$-C*H$_2$*), 1.74 - 1.66 (m, 2H, C*H$_2$*-CH$_2$-Cl), 1.56 (s, 6H, aryl-C-(C*H$_3$*)$_2$). ^{13}C NMR (101 MHz, CDCl$_3$) δ = 146.5 (*aryl-C*), 128.3 (*aryl-C*), 126.9 (*aryl-C*), 125.8 (*aryl-C*), 76.4 (aryl-*C*-OCH$_2$), 61.8 (O*C*H$_2$-CH$_2$), 45.1 (CH$_2$-*C*H$_2$-Cl), 29.7 (O-CH$_2$-*C*H$_2$), 28.4 (aryl-C-(*C*H$_3$)$_2$), 27.8 (*C*H$_2$-CH$_2$-Cl). HRMS-ESI (m/z): calculated for [$C_{13}H_{19}ClO$+K]$^+$: 265.0756; found: 265.0762.

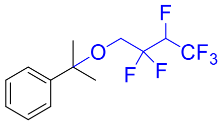

(2-(2,2,3,4,4,4-hexafluorobutoxy)propan-2-yl)benzene (10i) Pink oil (36.0 mg, 60%). ^{1}H NMR (400 MHz, $CDCl_3$) δ = 7.39 (d, *J* = 6.9 Hz, 4H, *aryl-H*), 7.30 (t, *J* = 6.5 Hz, 1H, *aryl-H*), 5.16 (m, 1H, CF_2-C*H*F-CF_3), 3.64 - 3.40 (m, 2H, O-CH_2-CF_2), 1.60 (s, 6H, aryl-C-(CH_3)$_2$). ^{13}C NMR (101 MHz, $CDCl_3$) δ = 144.2 (*aryl-C*), 128.7 (*aryl-C*), 127.7 (*aryl-C*), 125.9 (*aryl-C*), 118.6, 83.2, 81.3, 77.2 (aryl-*C*-(CH_3)$_2$), 60.4, 27.0, 26.6. ^{19}F NMR (377MHz, $CDCl_3$) δ = -74.0, -117.7, -214.3. HRMS-ESI (m/z): calculated for $[C_{13}H_{14}F_6O+Na]^+$: 323.0847; found: 323.0850.

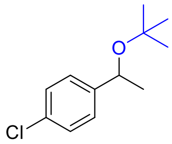

1-(1-(tert-butoxy)ethyl)-4-chlorobenzene (10j) Colorless oil (27.0 mg, 64%). ^{1}H NMR (400 MHz, $CDCl_3$) δ = 7.24 - 7.17 (m, 4H, *aryl-H*), 4.55 (q, *J* = 6.6 Hz, 1H, aryl-C*H*-CH_3), 1.26 (d, *J* = 6.5 Hz, 3H, CH-CH_3), 1.07 (s, 9H, O-CH(CH_3)$_3$). ^{13}C NMR (101 MHz, $CDCl_3$) δ = 146.2 (aryl-*C*), 132.2 (aryl-*C*), 128.4 (aryl-*C*), 127.1 (aryl-*C*), 74.4 (aryl-*C*H-CH_3), 69.4 (O-*C*H-(CH_3)$_3$), 28.6, 26.8. These spectroscopic data correspond to the reported data.[17]

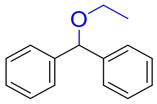

Benzhydryl ethyl ether (10k) Colorless oil (30.5 mg, 72%). ^{1}H NMR (400 MHz, $CDCl_3$) δ = 7.54 - 7.26 (m, 10H, *aryl-H*), 5.40 (s, 1H, aryl-C*H*-aryl), 3.57 (q, *J* = 7.1 Hz, 2H, OCH_2-CH_3), 1.31 (t, *J* = 7.1 Hz, 3H, OCH_2-CH_3). ^{13}C NMR (101 MHz, $CDCl_3$) δ = 142.7 (*aryl-C*), 128.5 (*aryl-C*), 127.5 (*aryl-C*), 127.1 (*aryl-C*), 83.6 (aryl-*C*H-aryl), 64.7 (O*C*H_2-CH_3), 15.5 (OCH_2-CH_3). These spectroscopic data correspond to the reported data[18].

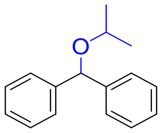

1,1′-[(1-Methylethoxy)methylene]bis[benzene] (10l) Colorless oil (31.6 mg, 70%). ^{1}H NMR (400 MHz, $CDCl_3$) δ = 7.46 - 7.26 (m, 10H, *aryl-H*), 5.54 (s, 1H, aryl-C*H*-aryl), 3.78 - 3.67 (m, 1H, O-C*H*-$(CH_3)_2$), 1.28 (d, *J* = 6.1 Hz, 6H, CH-$(CH_3)_2$). ^{13}C NMR (101 MHz, $CDCl_3$) δ = 143.1 (*aryl-C*), 128.4 (*aryl-C*), 127.4 (*aryl-C*), 127.2 (*aryl-C*), 80.6 (aryl-*C*H-aryl), 69.2 (O-*C*H-$(CH_3)_2$), 22.4 (O-CH-$(CH_3)_2$). These spectroscopic data correspond to the reported data[19].

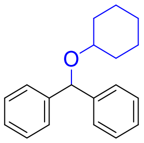

1,1′-[(Cyclohexyloxy)methylene]bis[benzene] (10m) Colorless oil (29.3 mg, 55%). ^{1}H NMR (400 MHz, $CDCl_3$) δ = 7.37 (m, 8H, aryl-*H*), 7.31 - 7.25 (m, 2H, aryl-*H*), 5.60 (s, 1H, aryl-C*H*-aryl), 3.42 (dt, *J* = 8.0, 3.9 Hz, 1H, Cy-*H*), 1.98 (m, 2H, Cy-*H*), 1.80 (m, 2H, Cy-H), 1.59 - 1.44 (m, 3H, Cy-H), 1.27 (m, 3H, Cy-H). ^{13}C NMR (101 MHz, $CDCl_3$) δ = 143.2 (aryl-*C*), 128.4 (aryl-*C*), 127.3 (aryl-*C*), 127.3 (aryl-*C*), 80.0 (aryl-*C*H-aryl), 75.2 (O-*C*H-$(CH_2)_2$), 32.5 (Cy-*C*), 26.0 (Cy-*C*), 24.3 (Cy-*C*). These spectroscopic data correspond to the reported data[20].

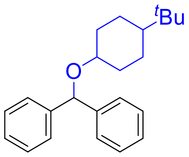

(((4-(tert-butyl)cyclohexyl)oxy)methylene)dibenzene (10n) Colorless oil (43.2 mg, 67%). (a mixture of two diastereomers): d.r. = 3.1:1 (by ^{1}H NMR spectroscopy. Reported spectral values are of the mixture). Major diastereomer: ^{1}H NMR (400 MHz, $CDCl_3$) δ = 7.34 - 7.29 (m, 8H, *aryl-H*), 7.24 - 7.28 (m, 2H, *aryl-H*). 5.60 (s, 1H, aryl-C*H*-aryl), 3.93 (m, 1H, *Cy-H*), 1.82 (dddd, *J* = 13.2, 7.9, 5.3, 3.8 Hz, 2H, *Cy-H*), 1.66 -

1.58 (m, 2H, *Cy-H*), 1.54 (dddd, *J* = 13.2, 7.9, 5.3, 3.8 Hz, 2H, *Cy-H*), 1.37 (dddd, J = 13.3, 8.1, 7.1, 5.4 Hz, 2H, *Cy-H*), 1.13 (tt, *J* = 5.9 Hz, 1H, *Cy-H*), 0.89 (s, 9H). ^{13}C NMR (101 MHz, $CDCl_3$) δ = 143.2 (*aryl-C*), 128.4 (*aryl-C*), 127.3 (*aryl-C*), 127.3 (*aryl-C*), 80.3 (aryl-*C*H-aryl), 76.4 (O-*C*H-CH_2), 47.6 (*Cy-C*), 33.0 (*Cy-C*), 32.4 (*Cy-C*), 27.8 (*Cy-C*), 25.8 (^{t}*Bu-C*). HRMS-ESI (m/z): calculated for $[C_{23}H_{30}O+K]^+$: 361.1934; found: 361.1929.

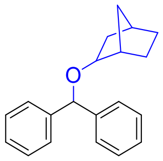

(1S,4R)-2-(benzhydryloxy)bicyclo[2.2.1]heptane (10o) Colorless oil (31.1 mg, 56%). Endo/exo = 4:1 (by ^{1}H NMR spectroscopy. Reported spectral values are of the mixture). ^{1}H NMR (400 MHz, $CDCl_3$) δ = 7.35 - 7.28 (m, 8H, aryl-*H*), 7.24 - 7.18 (m, 2H, aryl-*H*), 5.40 (s, 1H, aryl-C*H*-aryl, exo configuration), 5.33 (s, 1H, aryl-C*H*-aryl, endo configuration), 3.88 - 3.78 (m, 1H, CH-O-C*H*-CH_2, endo configuration), 3.41 (s, 1H, CH-O-C*H*-CH_2, exo configuration), 2.35 (s, 1H, endo configuration), 2.23, (s, 1H, exo configuration), 2.13 (s, 1H, endo configuration), 2.02 - 1.92 (m, 1H), 1.83 - 1.71 (m, 1H), 1.66 (d, *J* = 9.8 Hz, 1H, exo configuration), 1.52 (d, *J* = 4.4 Hz, 1H), 1.39 - 1.31 (m, 2H), 1.24 - 1.18 (m, 2H), 1.11 - 1.05 (m, 1H), 0.98 - 0.83 (m, 1H, exo configuration). ^{13}C NMR (101 MHz, $CDCl_3$) δ = 143.1 (aryl-*C*), 142.9 (aryl-*C*), 128.4 (aryl-*C*), 127.3 (aryl-*C*), 81.2 (O-*C*-CH_2, endo configuration), 80.5 (O-*C*-CH_2, exo configuration), 80.3 (aryl-*C*H-aryl,, exo configuration),77.8 (aryl-*C*H-aryl, endo configuration), 40.9 (exo configuration), 39.9 (endo configuration), 39.8 (exo configuration), 37.3 (endo configuration), 37.0 (endo configuration), 36.8 (endo configuration), 35.4 (exo configuration), 35.3 (exo configuration), 30.1, 28.7 (exo configuration), 24.6 (exo configuration), 20.8 (endo configuration). HRMS-ESI (m/z): calculated for $[C_{20}H_{22}O+H]^+$: 279.1749; found: 279.1746.

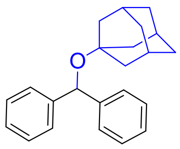

(3s,5s,7s)-1-(benzhydryloxy)adamantane (10p) white solid (53.4 mg, 84%). ^{1}H NMR (400 MHz, $CDCl_3$) δ = 7.55 - 7.27 (m, 8H, *aryl-H*), 7.22 (t, *J* = 7.1 Hz, 2H, *aryl-H*), 5.79 (s, 1H, aryl-C*H*-aryl), 2.14 (s, 3H), 1.92 - 1.76 (m, 6H), 1.74 - 1.58 (m, 6H). ^{13}C NMR (101 MHz, $CDCl_3$) δ = 145.3 (*aryl-C*), 128.2 (*aryl-C*), 127.2 (*aryl-C*), 126.9 (*aryl-C*), 74.4 (aryl-*C*H-aryl), 73.8, 42.9, 36.5, 30.8. HRMS-ESI (m/z): calculated for [$C_{23}H_{26}O$]: 318.1984; found: 318.1988.

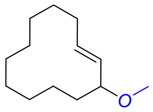

3-Methoxycyclododecene (11a) Colorless oil (21.9 mg, 56%, Z/E = 1: 2.9). ^{1}H NMR (400 MHz, $CDCl_3$) δ = 5.64 - 5.57 (m, 1H, alkenes-*H*), 5.30 - 5.16 (m, 1H, alkenes-*H*), 4.11 (q, *J* = 7.8 Hz, 1H, C*H*-OCH_3, Z configuration), 3.52 (td, *J* = 9.2, 3.9 Hz, 1H, C*H*-OCH_3, E configuration), 3.24 (s, 3H, OC*H*$_3$), 2.46 (m, 1H, C*H*$_2$-CH=CH, Z configuration), 2.23 (s, 1H, E configuration), 2.08 - 1.99 (m, 1H, E configuration), 1.90 - 1.95 (m, 1H, Z configuration), 1.81 - 1.75 (m, 1H), 1.62 - 1.10 (m, 16H). ^{13}C NMR (101 MHz, $CDCl_3$) δ = 135.1 (alkene-*C*, E configuration), 134.6 (alkene-*C*, Z configuration), 131.6 (alkene-*C*, E configuration), 131.1 (alkene-*C*, Z configuration), 83.4 (E configuration), 74.5 (Z configuration), 55.9 (E configuration), 55.9 (Z configuration), 33.8 (Z configuration), 33.0 (E configuration), 31.9 (E configuration), 26.8 (Z configuration), 26.1 (E configuration), 25.7 (E configuration), 25.1 (E configuration), 25.1 (E configuration), 24.8 (Z configuration), 24.8 (Z configuration), 24.6 (E configuration), 24.6 (E configuration), 24.4 (Z configuration), 24.4 (Z configuration), 22.9 (Z configuration), 22.8 (Z configuration), 22.6 (E configuration), 22.0 (Z configuration). HRMS-ESI (m/z): calculated for [$C_{13}H_{24}O$+H]$^{+}$:197.1900; found: 197.1897.

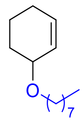

3-(octyloxy)cyclohex-1-ene (11b) Colorless oil (26.9 mg, 64%). ^{1}H NMR (400 MHz, $CDCl_3$) δ = 5.86 - 5.81 (m, 1H, alkenes-*H*), 5.78 - 5.74 (m, 1H, alkenes-*H*), 3.82 (s, 1H, C*H*-OCH_2), 3.59 - 3.39 (m, 2H, OCH_2-CH_2), 2.06 - 1.95 (m, 2 H), 1.85 - 1.72 (m, 2H), 1.71 - 1.63 (m, 1H), 1.60 - 1.52 (m, 3H), 1.32 - 1.26 (m, 10H), 0.87 (t, *J* = 6.6 Hz, 3H). ^{13}C NMR (101 MHz, $CDCl_3$) δ = 130.7 (alkenes-*C*), 128.3 (alkenes-*C*), 72.9 (*C*H-OCH_2), 68.5 (CH-O*C*H_2), 32.0, 30.4, 29.6, 29.4, 28.5, 26.4, 25.4, 22.8, 19.5, 14.2. HRMS-ESI (m/z): calculated for $[C_{14}H_{26}O+H]^+$: 211.2057; found: 211.2051.

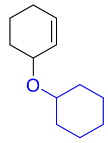

3-(cyclohexyloxy)cyclohex-1-ene (11c) Colorless oil (23.4 mg, 65%). ^{1}H NMR (400 MHz, $CDCl_3$) δ = 5.83 - 5.79 (m, 1H, alkenes-*H*), 5.72 - 5.68 (m, 1H, alkenes-*H*), 3.95 (s, 1H), 3.36 – 3.30 (m, 1H, C*H*-O-Cy), 2.06 - 1.99 (m, 1H), 1.95 – 1.85 (m, 3H), 1.77 - 1.72 (m, 4H), 1.66 - 1.59 (m, 1H), 1.56 - 1.47 (m, 2H), 1.32 - 1.17 (m, 5H). ^{13}C NMR (101 MHz, $CDCl_3$) δ = 130.3 (alkenes-*C*), 129.2 (alkenes-*C*), 75.6 (*C*H-OCH-CH_2), 70.2 (CH-O*C*H-CH_2), 33.7, 33.2, 29.6, 26.0, 25.4, 24.7, 24.6, 19.6. HRMS-ESI (m/z): calculated for $[C_{12}H_{20}O+H]^+$: 181.1587; found: 181.1583.

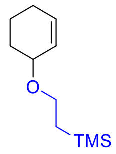

(2-(cyclohex-2-en-1-yloxy)ethyl)trimethylsilane (11d) Colorless oil (15.4 mg, 39%). ^{1}H NMR (400 MHz, $CDCl_3$) δ = 5.84 - 5.75 (m, 2H, alkenes-*H*), 3.83 (s, 1H, C*H*-O-CH_2), 3.62 - 3.43 (m, 2H, CH-O-CH_2), 2.04 – 2.03 (m, 1H), 1.84 - 1.79 (m, 1H), 1.76 - 1.73 (m, 1H), 1.68 - 1.63 (m, 1H), 1.58 - 1.51 (m, 1H), 1.28 - 1.24 (m, 1H), 0.96 - 0.92

(t, J = 7.4 Hz, 2H), 0.01 (s, 9H, TMS-H). ^{13}C NMR (101 MHz, $CDCl_3$) δ = 130.6 (alkenes-C), 128.3 (alkenes-C), 72.5 (CH-O-CH_2), 65.3 (CH-O-CH_2), 28.7, 25.4, 19.5, 18.8, -1.2 (TMS-C). HRMS-ESI (m/z): calculated for $[C_{11}H_{22}OSi+H]^+$: 199.1513; found: 199.1514.

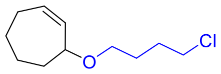

3-(4-chlorobutoxy)cyclohept-1-ene (11e) Colorless oil (19.4 mg, 48%). ^{1}H NMR (400 MHz, $CDCl_3$) δ = 5.75 (s, 2H, alkenes-H), 3.95 (d, J = 9.9 Hz, 1H, CH-O-CH_2), 3.57 (t, J = 6.7 Hz, 2H, CH_2-Cl), 3.50 - 3.44 (m, 2H), 2.18 - 2.13 (m, 1H), 2.04 - 1.95 (m, 2H), 1.89 - 1.84 (m, 3H), 1.75 - 1.69 (m, 2H), 1.60 - 1.51 (m, 3H), 1.37 - 1.25 (m, 1H). ^{13}C NMR (101 MHz, $CDCl_3$) δ = 136.6 (alkenes-C), 130.7 (alkenes-C), 79.6 (CH-O-CH_2), 67.7 (CH-OCH_2), 45.1, 33.1, 29.9, 28.7, 27.6, 27.5, 26.8. HRMS-ESI (m/z): calculated for $[C_{11}H_{19}ClO+Na]^+$: 225.1017; found: 225.1024.

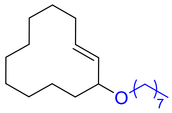

(E)-3-(octyloxy)cyclododec-1-ene (11f) Colorless oil (27.4 mg, 46%). ^{1}H NMR (400 MHz, $CDCl_3$) δ = 5.60 - 5.52 (m, 1H, alkenes-H), 5.30 - 5.23 (m, 1H, alkenes-H), 3.75 - 3.65 (m, 1H, CH-O-CH_2), 3.46 - 3.41 (m, 1H, O-CH_2-CH_2), 3.26 - 3.21 (m, 1H, O-CH_2-CH_2), 2.25 - 2.22 (d, J = 12.0 Hz, 1H), 2.06 - 1.97 (m, 1H),1.82 - 1.74 (m, 1H), 1.57 - 1.50 (m, 4H), 1.46 - 1.38 (m, 4H), 1.28 - 1.22 (m, 19H), 0.87 (t, J = 6.9 Hz, 3H). ^{13}C NMR (101 MHz, $CDCl_3$) δ = 141.7 (alkenes-C), 132.5 (alkenes-C), 81.7 (CH-O-CH_2), 68.2 (O-CH$_2$-CH_2), 33.2, 32.0, 31.8, 30.1, 29.6, 29.4, 26.4, 26.2, 25.7, 25.3, 25.1, 24.7, 24.6, 22.8, 22.7, 14.2. HRMS-ESI (m/z): calculated for $[C_{20}H_{38}O+H]^+$: 295.2996; found: 295.2990.

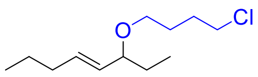

(E)-3-(4-chlorobutoxy)oct-4-ene(11g) Colorless oil (22.2 mg, 51%). ^{1}H NMR (400 MHz, $CDCl_3$) δ = δ 5.67 - 5.50 (m, 1H, alkenes-*H*), 5.32 - 5.19 (m, 1H, alkenes-*H*), 3.56 (t, *J* = 7.0 Hz, 2H, CH_2-Cl), 3.51 - 3.42 (m, 2H), 3.28 - 3.23 (m, 1H), 2.10-1.98 (m, 2H), 1.90-1.80 (m, 2H), 1.74 - 1.54 (m, 4H), 1.46 - 1.36 (m, 2H), 0.93 - 0.84 (m, 6H). ^{13}C NMR (101 MHz, $CDCl_3$) δ = 133.8 (alkenes-*C*), 131.2 (alkenes-*C*), 82.6, 67.1, 45.2, 34.4, 29.8, 28.7, 27.4, 25.4, 22.6, 13.8, 10.1. HRMS-ESI (m/z): calculated for $[C_{12}H_{23}ClO+Na]^+$: 241.1329; found: 241.1331.

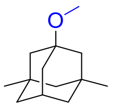

1-Methoxy-3,5-dimethyltricyclo[3.3.1.1^{3,7}]decane (11i) Colorless oil (19.0 mg, 49%). ^{1}H NMR (400 MHz, $CDCl_3$) δ = 3.22 (s, 3H, O-CH_3), 2.20 (m, 1H, $CH(CH_2)_3$), 1.58 (d, *J* = 3.3 Hz, 2H), 1.42 - 1.33 (m, 4H), 1.31 - 1.24 (m, 4H), 1.12 (s, 2H), 0.87 (s, 6H, CH_3). ^{13}C NMR (101 MHz, $CDCl_3$) δ = 73.8 (*C*-OCH_3), 51.0 (C-O*C*H_3), 48.2, 47.2, 43.0, 39.6, 33.6, 31.0, 30.3. These spectroscopic data correspond to the reported data[21].

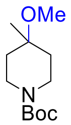

1,1-Dimethylethyl 4-methoxy-4-methyl-1-piperidinecarboxylate (11j) Colorless oil (16.0 mg, 35%). ^{1}H NMR (400 MHz, $CDCl_3$) δ = 3.66 (ddd, *J* = 13.4, 4.2, 4.2 Hz, 2H, CH_2-NBoc), 3.15 (s, 3H, -OCH_3), 3.11 - 3.01 (m, 2H, CH_2-NBOc), 1.69 - 1.65 (m, 2H, NBoc-CH_2-CH_2), 1.41 (s, 9H, CO-O-$(CH_3)_3$), 1.40 - 1.31 (m, 2H, NBoc-CH_2-CH_2), 1.11 (s, 3H, OCH_3-C-CH_3). ^{13}C NMR (101 MHz, $CDCl_3$) δ = 155.0 (*C*O-O-^{t}Bu), 79.3 (CH_2-NBoc), 71.5 (CH_2-NBoc), 48.7 (C-OCH_3), 39.8 (CO-O-*C*-$(CH_3)_3$), 35.2 (NBoc-CH_2-CH_2), 28.5 ($(CH_3)_3$-C), 23.9 (OCH_3-C-CH_3). These spectroscopic data correspond to the reported data[22].

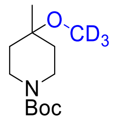

1,1-Dimethylethyl 4-(methoxy-*d*3)-4-methyl-1-piperidinecarboxylate (11k)

Colorless oil (15.2 mg, 33%). ^{1}H NMR (400 MHz, $CDCl_3$) δ = 3.64 (d, *J* = 13.3 Hz, 2H, C*H2*-NBoc), 3.07 (t, *J* = 10.6 Hz, 2H, C*H2*-NBoc), 1.66 (d, *J* = 13.3 Hz, 2H), 1.40 (s, 9H, ^{t}Bu-*H*), 1.35 - 1.32 (m, 2H), 1.09 (s, 3H, -C*H3*). ^{13}C NMR (101 MHz, $CDCl_3$) δ = 155.0 (*C*O), 79.3 (COO-*C*(CH_3)$_3$), 71.4 (*C*-OCD_3), 47.9 (O*C*D_3), 39.8, 35.3, 28.5, 23.8. These spectroscopic data correspond to the reported data[23].

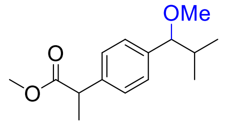

methyl 2-(4-(1-methoxy-2-methylpropyl)phenyl)propanoate (12a) Colorless oil (38.0 mg, 76%). (a mixture of two diastereomers): d.r. = 1:1 (by ^{13}C NMR spectroscopy. Reported spectral values are of the mixture).^{1}H NMR (400 MHz, $CDCl_3$) δ = 7.22 (d, *J* = 6.4 Hz, 2H, *aryl-H*), 7.15 (d, *J* = 6.3 Hz, 2H, *aryl-H*), 3.70 (m, 2H, aryl-C*H*-OCH_3, aryl-C*H*-CH_3), 3.62 (s, 3H, CO-OC*H3*), 3.13 (s, 3H, aryl-CH-OC*H3*), 1.87 - 1.81 (m, 1H, C*H*-(CH_3)$_2$), 1.47 (d, *J* = 7.3 Hz, 3H, aryl-CH-C*H3*), 0.94 (d, *J* = 8.6 Hz, 3H, CH-(C*H3*)$_2$), 0.70 (d, *J* = 6.8 Hz, 3H, CH-(C*H3*)$_2$). ^{13}C NMR (101 MHz, $CDCl_3$) δ = 175.1 (*C*O-OCH_3), 140.0 (*aryl-C*), 139.4 (*aryl-C*), 127.7 (*aryl-C*), 127.1 (*aryl-C*), 89.4 (aryl-*C*H-OCH_3), 57.0 (CH-O*C*H_3), 52.0 (CO-O*C*H_3), 45.1 (aryl-*C*H-CO), 34.7 (*C*H-(CH_3)$_2$), 19.0 (CH-CH-(*C*H_3)$_2$), 18.9 (CH-CH-(*C*H_3)$_2$), 18.7 (d, *J* = 2.5 Hz, aryl-CH-*C*H_3, main basis for judging it as diastereoisomer). These spectroscopic data correspond to the reported data[7].

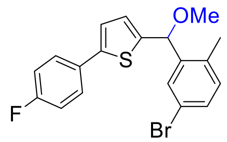

2-((5-bromo-2-methylphenyl)(methoxy)methyl)-5-(4-fluorophenyl)thiophene (12b)

White solid (64.1 mg, 89%). ^{1}H NMR (400 MHz, $CDCl_3$) δ = 7.74 (d, *J* = 2.3 Hz, 1H,

aryl-H), 7.51 (dd, J = 8.8, 5.2 Hz, 2H, *aryl-H*), 7.36 (dd, J = 8.1, 2.3 Hz, 1H, *aryl-H*), 7.16 - 6.98 (m, 4H, *aryl-H*), 6.76 (d, J = 3.7 Hz, 1H, *aryl-H*), 5.53 (s, 1H, aryl-C*H*-OCH3), 3.43 (s, 3H, CH-OC*H3*), 2.25 (s, 3H, aryl-C*H3*). ^{13}C NMR (101 MHz, $CDCl_3$) δ = 162.5 (d, J = 247.4 Hz, *aryl-C*), 143.9 (*aryl-C*), 143.8 (*aryl-C*), 141.3 (*aryl-C*), 134.7 (*aryl-C*), 132.4 (*aryl-C*), 130.9 (*aryl-C*), 129.2 (*aryl-C*), 127.5 (*aryl-C*), 127.5 (*aryl-C*), 127.0 (*aryl-C*), 122.4 (*aryl-C*), 120.2 (*aryl-C*), 115.9 (d, J = 21.8 Hz, *aryl-C*), 78.1 (aryl-*C*H-aryl), 57.1 (aryl-CH-OC*H3*), 18.9 (aryl-C*H3*). ^{19}F NMR (376 MHz, $CDCl_3$) δ = -114.5. These spectroscopic data correspond to the reported data[7].

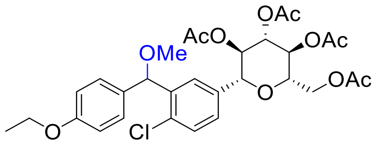

(2S,3R,4S,5R,6R)-6-(4-chloro-3-((4-ethoxyphenyl)(methoxy)methyl)phenyl) tetrahydr-2H-pyran-2,3,4,5-tetrayl tetraacetate (12c) white solid (64.2 mg, 64%). (a mixture of two diastereomers): d.r. = 1.3:1 (by 1H NMR spectroscopy. Reported spectral values are of the mixture). Major diastereomer: ^{1}H NMR (400 MHz, $CDCl_3$) δ = 7.54 (d, J = 2.1 Hz, 1H, major diastereomer), 7.48 (d, J = 2.1 Hz, 1H, minor diastereomer), 7.33 - 7.35 (d, J = 9.6 Hz, 1H, minor diastereomer), 7.31 (d, J = 9.6, 1H, major diastereomer), 7.27 - 7.21 (m, 3H), 6.85 - 6.80 (m, 2H), 5.61 (s, 1H, minor diastereomer), 5.55 (s, 1H, major diastereomer), 5.36 - 5.30 (m, 5H, minor diastereomer), 5.29 - 5.21 (t, J = 9.7 Hz, 3H), 5.12 (t, J = 9.6 Hz, 1H, major diastereomer), 5.06 (t, J = 9.6 Hz, 1H), 4.42 – 4.39 (d, J = 9.6 Hz, 1H), 4.30 - 4.26 (dd, J = 12.3, 4.6, 1H), 4.18 (d, J = 12.9, 1H), 4.00 (q, J = 7.1 Hz, 2H, major diastereomer), 3.98 (q, J = 7.0 Hz, 2H), 3.85 - 3.82 (m, 3H), 3.37 (s, 3H, minor diastereomer), 3.32 (s, 3H, major diastereomer), 2.09 (s, 3H), 2.07 (s, 3H), 2.02 (s, 3H, minor diastereomer), 1.99 (s, 3H, major diastereomer), 1.88 (s, 3H, major diastereomer), 1.62 (s, 3H, minor diastereomer), 1.41 – 1.36 (m, 8H), 1.26 (s, 1 H). ^{13}C NMR ($CDCl_3$, 101 MHz): 170.9 (*C*O), 170.5 (*C*O), 169.7 (*C*O), 169.1 (*C*O), 168.9 (*C*O), 168.8 (*C*O), 158.6 (*C*O), 158.5 (*C*O), 139.9 (*aryl-C*), 139.7 (*aryl-C*), 135.7 (*aryl-C*), 135.5 (*aryl-C*), 133.3 (*aryl-C*),

133.1 (*aryl-C*), 132.5 (*aryl-C*), 132.0 (*aryl-C*), 130.0 (*aryl-C*), 129.9 (*aryl-C*), 128.9 (*aryl-C*), 128.5 (*aryl-C*), 127.1 (*aryl-C*), 126.9 (*aryl-C*), 126.8 (*aryl-C*), 126.6 (*aryl-C*), 114.4 (*aryl-C*), 114.3 (*aryl-C*), 114.2 (*aryl-C*), 80.9 (aryl-*C*H-aryl), 80.7 (aryl-*C*H-aryl), 79.7, 79.5, 77.2, 76.2,76.0, 74.2, 74.1, 72.7, 72.6, 68.6, 63.5, 63.4, 62.4, 57.3, 56.9, 20.9, 20.8, 20.6, 20.4, 20.3, 15.0, 14.8. These spectroscopic data correspond to the reported data[7].

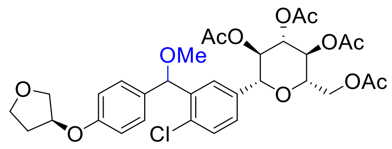

(2S,3R,4S,5R,6R)-6-(4-chloro-3-(methoxy(4-(((S)-tetrahydrofuran-3-yl)oxy)phenyl)methyl)phenyl)tetrahydro-2H-pyran-2,3,4,5-tetrayl tetraacetate (12d). White solid (76.5 mg, 59%). white solid (64.2 mg, 64%). (a mixture of two diastereomers): d.r. = 1.3:1 (by 1H NMR spectroscopy. Reported spectral values are of the mixture). Major diastereomer: ^{1}H NMR (400 MHz, $CDCl_3$) δ = 7.53 (s, 1H, major diastereomer), 7.49 (s, 1H, minor diastereomer), 7.34 (s, 1H), 7.32 – 7.30 (d, *J* = 8.5 Hz, 2H, major diastereomer), 7.25 - 7.21 (m, 4H), 6.81 - 6.77 (m, 5H), 5.60 (s, 1H, minor diastereomer), 5.55 (s, 1H, major diastereomer), 5.36 - 5.28 (m, 5H), 5.25 - 5.20 (t, *J* = 9.7 Hz, 3H), 5.15- 5.06 (m, 3H), 4.88 (m, 2H), 4.41 - 4.38 (d, *J* = 9.6 Hz, 2H), 4.29 - 4.25 (dd, *J* = 12.3, 4.6 Hz, 3H), 4.19 - 4.11 (m, 3H), 4.00 – 3.97 (m, 3H, major diastereomer), 3.98 -3.93 (m, 4H), 3.85 - 3.82 (m, 3H), 3.37 (s, 3H, minor diastereomer), 3.32 (s, 3H, major diastereomer), 2.09 - 2.07 (m, 8H), 2.06 - 2.04 (m, 8H), 2.01 – 1.99 (d, *J* = 7.7 Hz, 8H), 1.87 (s, 3H, major diastereomer), 1.62 (s, 3H, minor diastereomer), 1.58 (s, 4H), 1.28 – 1.25 (m, 4H). ^{13}C NMR ($CDCl_3$, 101 MHz): 170.8 (*C*O), 170.5 (*C*O), 170.5 (*C*O), 169.6 (*C*O), 168.9 (*C*O), 168.8 (*C*O), 156.1 (*C*O), 156.1 (*C*O), 139.1 (*aryl-C*), 139.1 (*aryl-C*), 135.3 (*aryl-C*), 135.3 (*aryl-C*),134.8 (*aryl-C*), 131.7 (*aryl-C*), 130.1 (*aryl-C*), 130.0 (*aryl-C*), 129.9 (*aryl-C*), 126.3 (*aryl-C*), 115.6 (*aryl-C*), 79.6 (aryl-*C*H-aryl), 77.4 (aryl-*C*H-aryl), 76.3.6, 74.3, 73.3, 72.7, 68.7, 67.3, 62.4, 38.4, 33.2,

20.9, 20.8, 20.4. HRMS-ESI (m/z): calculated for $[C_{32}H_{38}ClO_{12}+H]^+$: 649.2025; found: 649.2021.

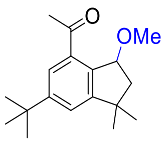

1-(6-(tert-butyl)-3-methoxy-1,1-dimethyl-2,3-dihydro-1H-inden-4-yl)ethan-1-one (12e) Colorless oil (36.0 mg, 66%). ^{1}H NMR (400 MHz, $CDCl_3$) δ = 7.66 (s, 1H, *aryl-H*), 7.36 (s, 1H, *aryl-H*), 5.21 (dd, *J* = 5.8, 3.1 Hz, 1H, aryl-C*H*-OCH_3), 3.42 (s, 3H, CH-OC*H₃*), 2.62 (s, 3H, aryl-CO-C*H₃*), 2.10 – 2.03 (m, 2H, aryl-CH-C*H₂*), 1.35 (s, 3H), 1.34 (s, 9H, ^{t}Bu-H), 1.30 (s, 3H). ^{13}C NMR (101 MHz, $CDCl_3$) δ = 200.8 (*C*O-CH_3), 154.3 (*aryl-C*), 152.7 (*aryl-C*), 137.9 (*aryl-C*), 135.3 (*aryl-C*), 125.1 (*aryl-C*), 123.3 (*aryl-C*), 81.7 (aryl-*C*H-OCH_3), 57.2 (CH-O*C*H_3), 46.2, 42.7, 35.0, 31.5, 31.3, 29.8, 28.8. These spectroscopic data correspond to the reported data[7].

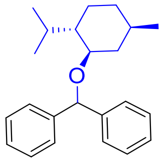

(((2-isopropyl-5-methylcyclohexyl)oxy)methylene)dibenzene (12f) White crystal (57.1 mg, 88%). ^{1}H NMR (400 MHz, $CDCl_3$) δ = 7.39 - 7.27 (m, 8H, *aryl-H*), 7.25 - 7.17 (m, 2H, *aryl-H*), 5.54 (s, 1H, aryl-C*H*-aryl), 3.15 (td, J = 10.4, 4.1 Hz, 1H), 2.44 - 2.29 (m, 1H), 2.17 (d, *J* = 12.3 Hz, 1H), 1.66 - 1.56 (m, 2H), 1.39 - 1.31 (m, 1H), 1.26 (s, 1H), 0.89 (dd, *J* = 6.8, 4.0 Hz, 9H), 0.44 (d, *J* = 6.9 Hz, 3H). ^{13}C NMR (101 MHz, $CDCl_3$) δ = 140.0 (*aryl-C*), 142.7 (*aryl-C*), 128.4 (*aryl-C*), 128.3 (*aryl-C*), 128.1 (*aryl-C*), 127.6 (*aryl-C*), 127.1 (*aryl-C*), 126.8 (*aryl-C*), 80.0 (aryl-*C*H-aryl), 76.0 ((aryl)$_2$-CH-O-*C*H), 48.8, 40.5, 34.7, 31.6, 25.2, 23.0, 22.6, 21.5, 15.8. These spectroscopic data correspond to the reported data[20].

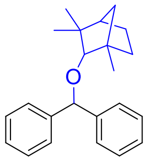

2-(benzhydryloxy)-1,3,3-trimethylbicyclo[2.2.1]heptane (12g) Colorless solid (40.3 mg, 63%). ^{1}H NMR (400 MHz, $CDCl_3$) δ = 7.43 (m, 4H, *aryl-H*), 7.36 (m, 4H, *aryl-H*), 7.30 (t, *J* = 8.1 Hz, 2H, *aryl-H*), 5.43 (s, 1H, aryl-C*H*-aryl), 3.14 (s, 1H, O-C*H*-C), 2.06 (t, *J* = 13.8 Hz, 1H, Cy-*H*), 1.83 (m, 1H), 1.67 (m, 1H), 1.45 (m, 2H), 1.11 (m, 2H), 1.04 (s, 6H), 0.96 (s, 3H). ^{13}C NMR (101 MHz, $CDCl_3$) δ = 143.7 (*aryl-C*), 143.1 (*aryl-C*), 128.2 (*aryl-C*), 128.2 (*aryl-C*), 127.8 (*aryl-C*), 127.4 (*aryl-C*), 127.1 (*aryl-C*), 127.1 (*aryl-C*), 88.8 (aryl-*C*H-aryl), 82.9, 49.4, 49.0, 41.5, 39.6, 31.4, 26.4, 26.4, 21.6, 20.2. HRMS-ESI (m/z): calculated for $[C_{23}H_{28}O+H]^+$: 321.2185; found: 321.2189.

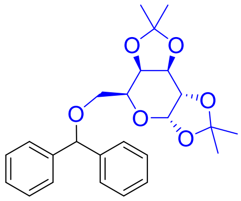

5-((benzhydryloxy)methyl)-2,2,7,7-tetramethyltetrahydro-5H-bis([1,3]dioxolo)[4,5-b:4',5'-d]pyran (12h) Pale yellow oil (36.6 mg, 43%). ^{1}H NMR (400 MHz, $CDCl_3$) δ = 7.42 - 7.31 (m, 8H, *aryl-H*), 7.29 (d, *J* = 8.9 Hz, 2H, *aryl-H*), 5.58 (d, *J* = 5.0 Hz, 1H), 5.51 (s, 1H, aryl-C*H*-aryl), 4.65 (dd, *J* = 8.1, 2.4 Hz, 1H), 4.41 - 4.33 (m, 2H), 4.15 - 4.09 (m, 1H), 3.75 - 3.63 (m, 2H), 1.60 (s, 3H), 1.45 (s, 3H), 1.38 (s, 6H). ^{13}C NMR (101 MHz, $CDCl_3$) δ = 142.2 (*aryl-C*), 128.4 (*aryl-C*), 128.4 (*aryl-C*), 127.6 (*aryl-C*), 127.5 (*aryl-C*), 127.3 (*aryl-C*), 127.2 (*aryl-C*), 109.2 (*aryl-C*), 108.7 (*aryl-C*), 96.4, 84.0, 71.2, 70.8, 70.7, 67.7, 67.1, 53.6, 26.3, 26.1, 25.1, 24.6. HRMS-ESI (m/z): calculated for $[C_{25}H_{30}O_6]$: 426.2042; found: 426.2047.

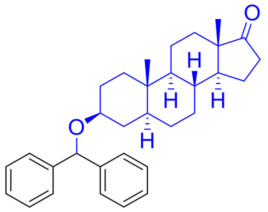

3-(benzhydryloxy)-10-methylhexadecahydro-17H-cyclopenta[a]phenanthren-17-one (12i) White solid (39.2 mg, 43%). ^{1}H NMR (400 MHz, $CDCl_3$) δ = 7.39 - 7.33 (m, 8H, *aryl-H*), 7.28 - 7.24 (m, 2H, *aryl-H*), 5.60 (s, 1H, aryl-C*H*-aryl), 3.36 (m, 1H), 2.49 – 2.41 (m, 1H), 2.12 - 2.03 (m, 2H), 1.97 - 1.94 (m, 3H), 1.79 - 1.67 (m, 4H), 1.57 - 1.48 (m, 3H), 1.33 - 1.23 (m, 6H), 1.05 (s, 1H), 0.87 (s, 6H), 0.68 - 0.63 (m, 1H). ^{13}C NMR (101 MHz, $CDCl_3$) δ = 193.2 (*C*O), 143.1 (*aryl-C*), 128.4 (*aryl-C*), 127.4 (*aryl-C*), 127.2 (*aryl-C*), 80.3, 76.2 (aryl-*C*H-aryl), 60.5, 54.6, 51.5, 47.9, 44.9, 37.1, 36.1, 35.9, 35.1, 35.1, 31.7, 31.0, 28.6, 28.5, 21.9, 20.6, 13.9, 12.4. HRMS-ESI (m/z): calculated for [$C_{32}H_{40}O_2$]: 456.3028; found: 456.3030.

Scale-up experiment.

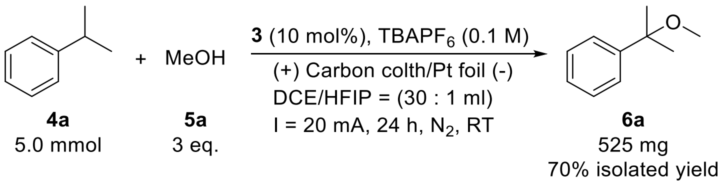

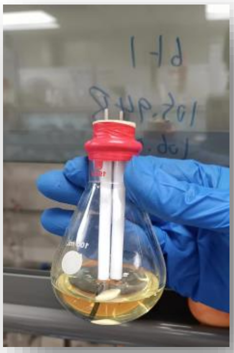

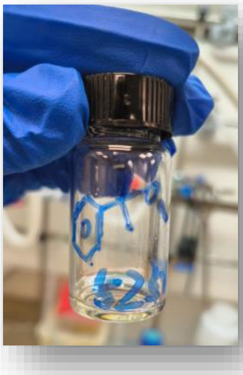

General procedure for scale-up experiment: In a N_2 atmosphere glovebox, a mixture of cumene (**4a**, 5.0 mmol), MeOH (**5a**, 15 mmol), catalyst 3 (0.5 mmol), $TBAPF_6$ (0.1M), DCE (30 ml) and HFIP (1.0 ml) were added to a 100 mL round bottom flask. The flask was sealed with a rubber plug equipped with carbon cloth (2.0×2.0 cm^2) and Pt foil (1.0×1.0 cm^2) electrodes. The reaction was electrolyzed in an undivided cell under a constant current (20 mA) for 24 h. After the reaction was completed, the solvent was removed under reduced pressure. The residue was purified by flash column chromatography, eluting with PE/EA = 20: 1 to afford the colorless oil in 70% yield (525 mg).

5. Mechanistic study

1). CV of cumene

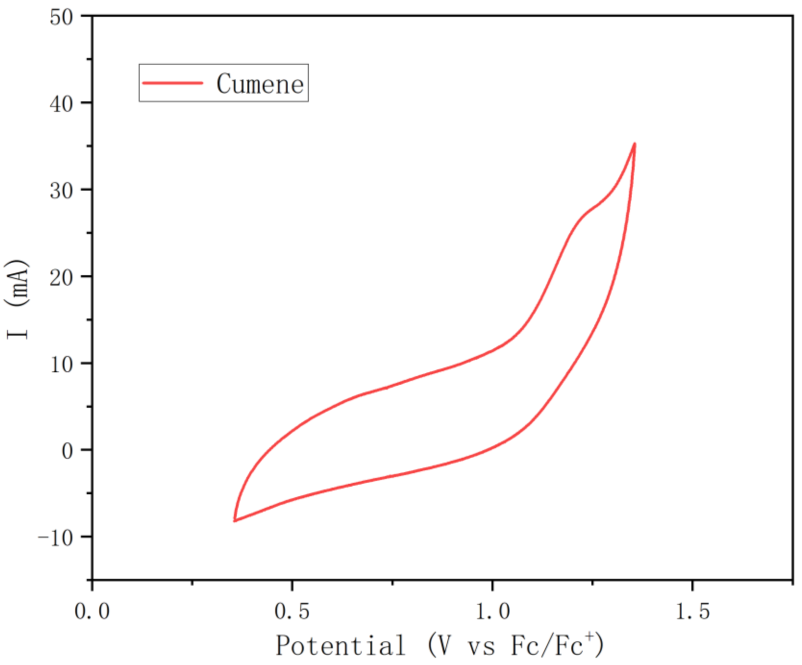

Figure S1. Cyclic voltammetry curves of cumene. CV measurements were performed with cumene (15 mM) in CH_3CN, using $TBAPF_6$ (0.1 M) as the electrolyte, glassy carbon electrode as the working electrode, Pt wire as the counter electrode, and Ag/AgCl as the reference electrode. Scan rate: 50 mV/s. For conversion to the SCE reference, it is known that Fc/Fc^+ is 380 mV more positive than SCE in MeCN[23].

2) The anode potential during the reaction

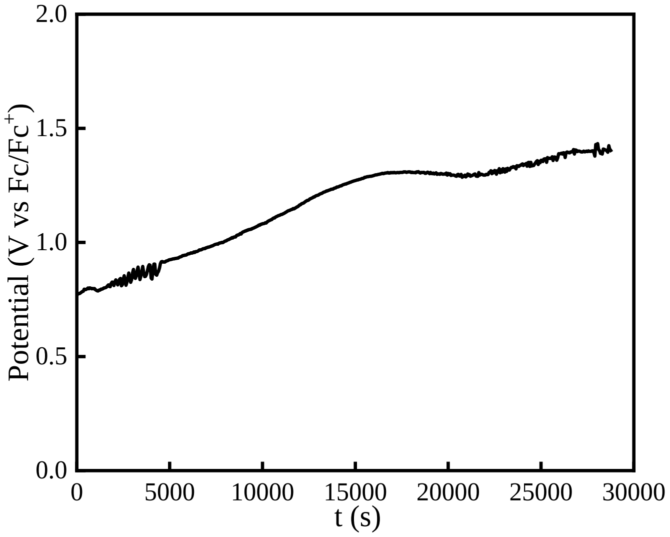

Figure S2. The anode potential of the reaction under standard conditions

2). KIE experiments

The kinetic isotope effect (KIE) experiment (Figure 3C) was measured by parallel experiments using 4-chloroethylbenzene or isotope-labeled 4-chloroethylbenzene as substrates. The reactions were carried out under standard conditions for 2 h. The GC analysis of this reaction yielded a KIE value (k_H/k_D) of 3.0.

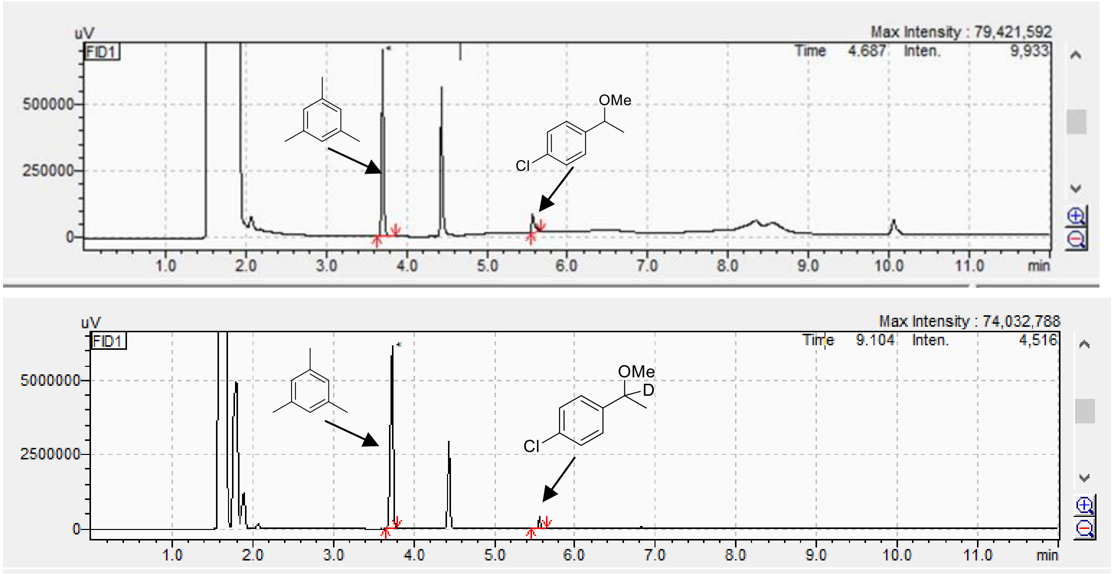

Figure S3. GC spectra of the KIE experiments

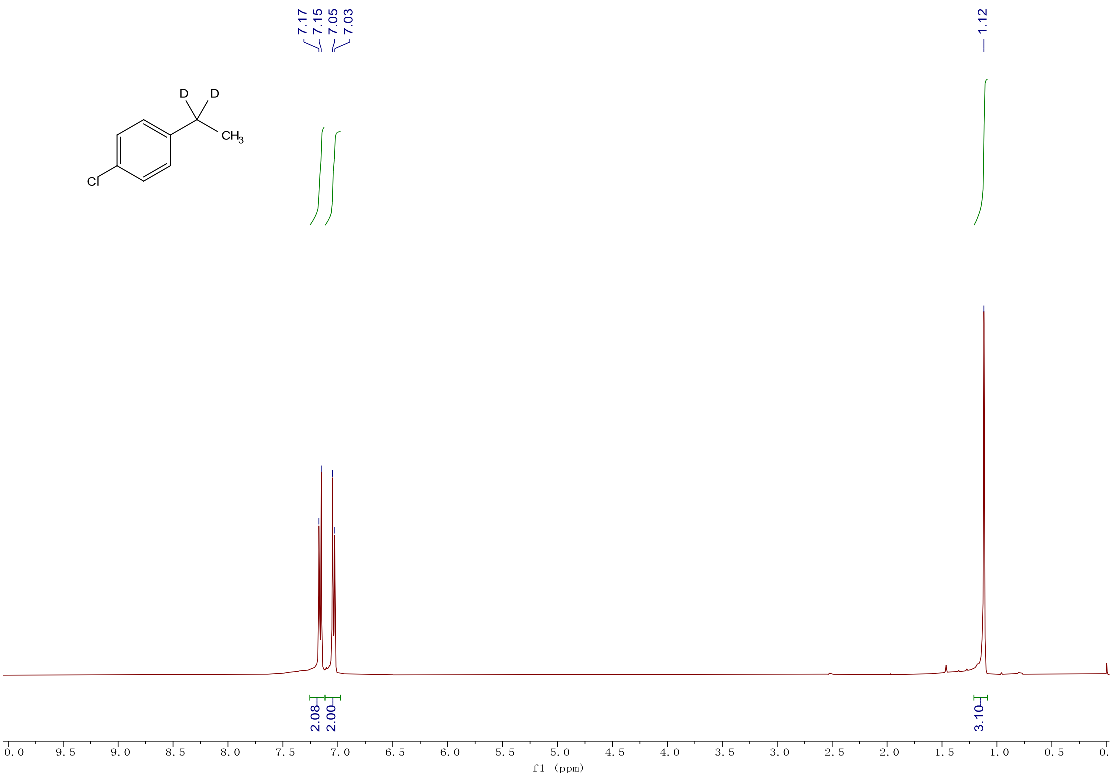

^{1}H NMR (400 MHz, $CDCl_3$) of **14**

3). Radical trapping experiments via the "cation pool" strategy.

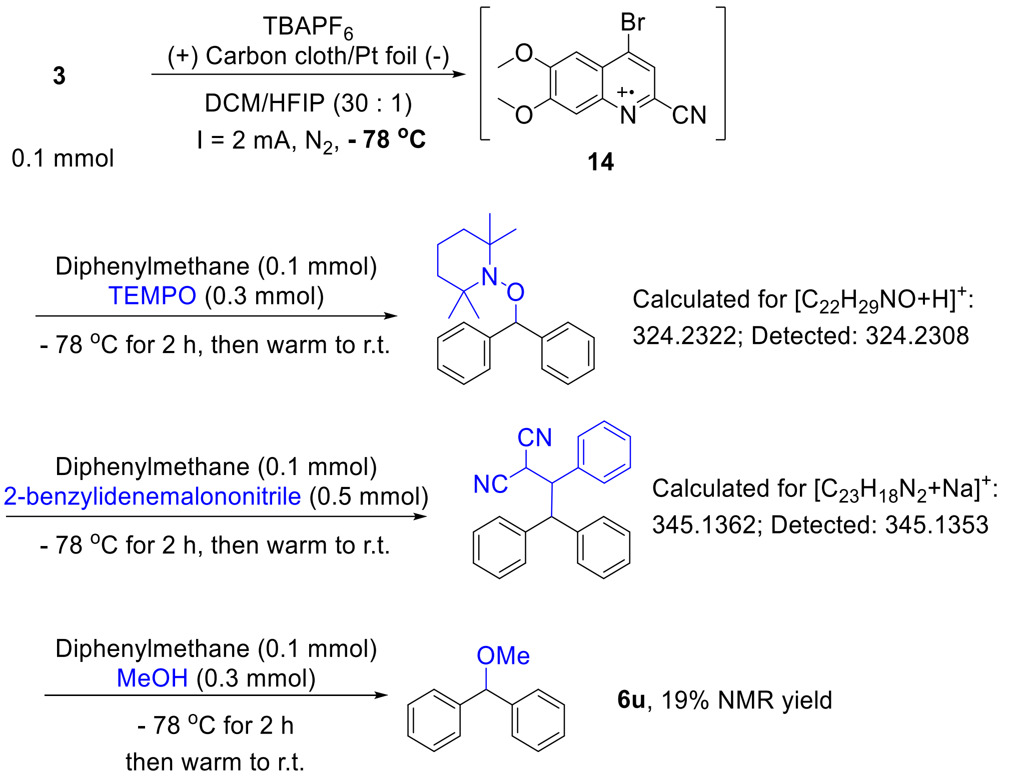

Procedure for radical trapping experiments:

(1) In a N_2 atmosphere glovebox, a mixture of catalyst **3** (0.1 mmol), $TBAPF_6$ (0.1M), DCM (3 ml), and HFIP (100 μL) were added to a 10 mL tube equipped with a stir bar. The tube was sealed with a rubber plug equipped with carbon cloth (1.0×1.0 cm^2) and Pt foil (1.0×1.0 cm^2) electrodes. The mixture was cooled to -78 °C and the reaction was electrolyzed in an undivided cell under a constant current (2 mA) for 2 h. After that, the electrolysis was stopped and a solution of diphenylmethane (0.1 mmol) and TEMPO (0.3 mmol)/2-benzylidenemalononitrile (0.5 mmol) in DCM was added dropwise. The reaction was stirred for an extra 2 hours at - 78 °C and then allowed to warm to room temperature. The desired product was detected by HR-MS.

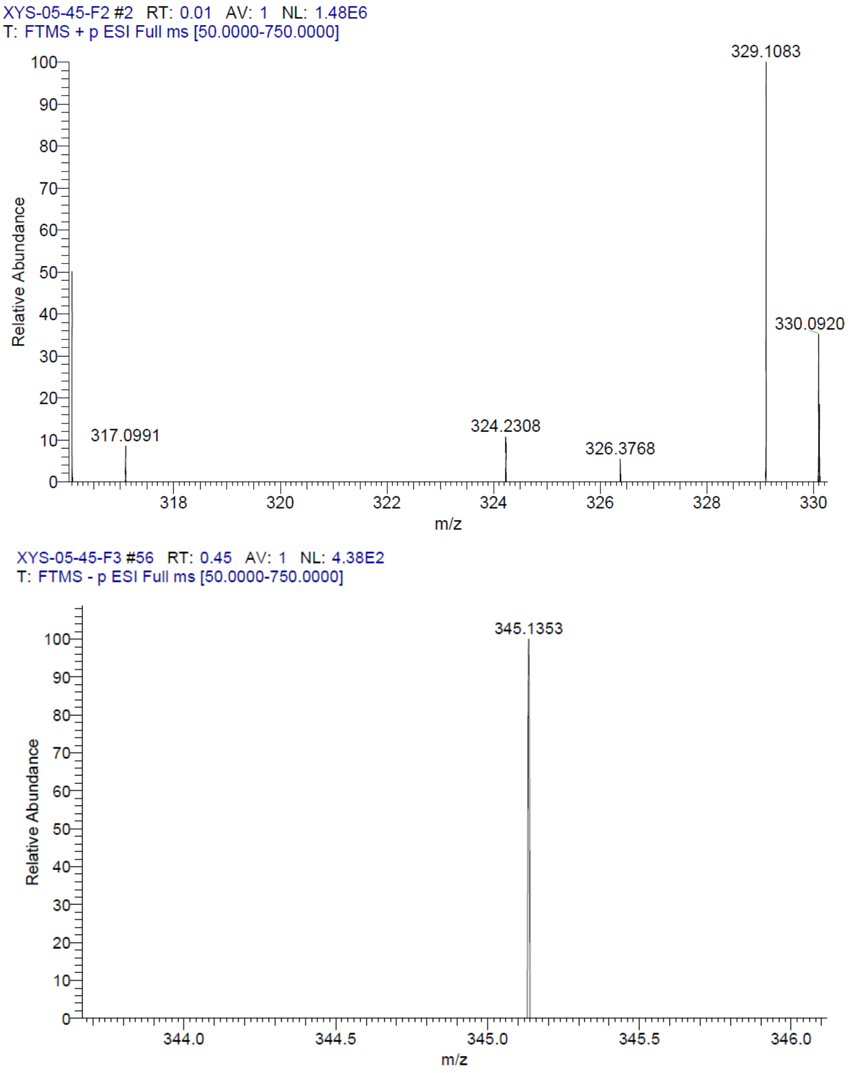

Figure S4. The HR-MS spectra of the radical trapping products

(2) In a N_2 atmosphere glovebox, a mixture of catalyst **3** (0.1 mmol), $TBAPF_6$ (0.1M), DCM (3 ml), and HFIP (100 μL) were added to a 10 mL tube equipped with a stir bar. The tube was sealed with a rubber plug equipped with carbon cloth (1.0×1.0 cm^2) and Pt foil (1.0×1.0 cm^2) electrodes. The mixture was cooled to -78 °C and the reaction was electrolyzed in an undivided cell under a constant current (2 mA) for 2 h. After that, the electrolysis was stopped and a solution of diphenylmethane (0.2 mmol) and MeOH (0.6 mmol) in DCM was added dropwise. The reaction was stirred for an extra 2 hours at -78 °C and then allowed to warm to room temperature. The desired product was detected by GC-MS and the NMR yield (19%) was obtained with CH_2Br_2 (0.05 mmol) as the internal standard.

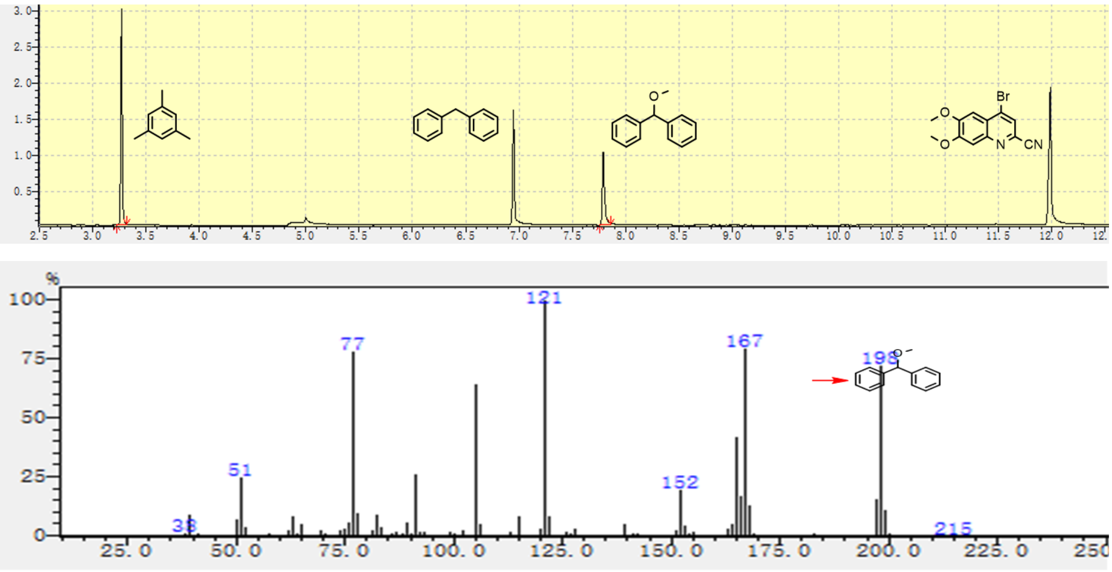

Figure S5. The GC spectrum of the reaction and the mass spectrogram of the product

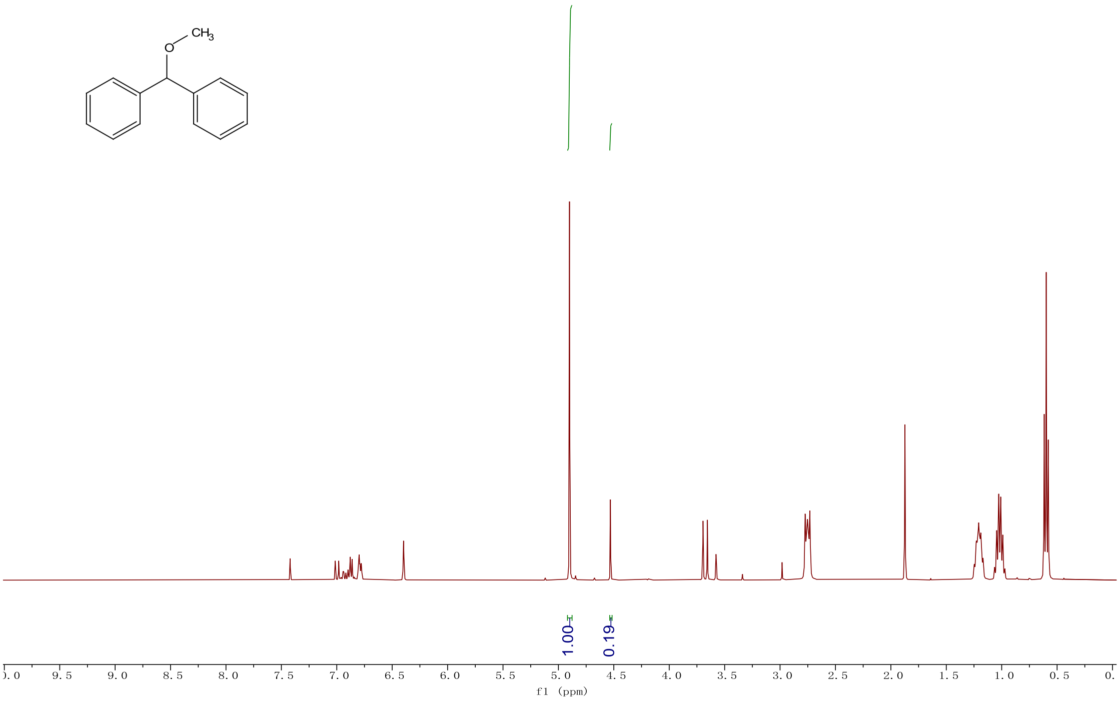

^{1}H NMR (400 MHz, $CDCl_3$) of **17**

4). EPR experiment

EPR experiment was conducted as follows to identify the presence of the quinoline radical cation. In a N_2 atmosphere glovebox, a mixture of catalyst 3 (0.1 mmol), TBAPF6 (0.1M), DCM (3 ml), and HFIP (100 μL) were added to a 10 mL tube equipped

with a stir bar. The tube was sealed with a rubber plug equipped with carbon cloth (1.0×1.0 cm^2) and Pt foil (1.0×1.0 cm^2) electrodes. The mixture was cooled to -78 ºC and the reaction was electrolyzed in an undivided cell under a constant current (2 mA) for 2 h. The mixture was transferred to an EPR tube for EPR measurement under ambient conditions.

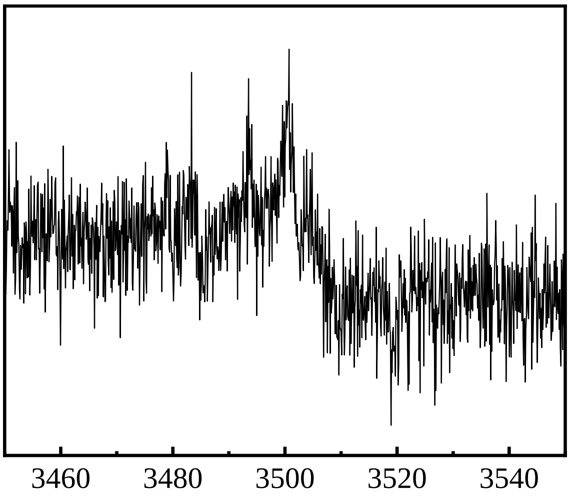

Figure S6. EPR spectra of the radical cation

5). DFT calculations

All calculations were carried out using the Gaussian09 package.[24] Geometries of stationary points were optimized using the B3LYP functional in the 6-311++G(d,p) basis set. Solvation effects were modeled using the IEFPCM model (dichloroethane). All key structures are given in the .xyz format (visualized with CYLview26 [25]) with energies in Hartrees:

Hydrogen：

Zero-point correction= 0.000000 (Hartree/Particle)
Thermal correction to Energy= 0.001416
Thermal correction to Enthalpy= 0.002360
Thermal correction to Gibbs Free Energy= -0.010654
Sum of electronic and zero-point Energies= -0.502281
Sum of electronic and thermal Energies= -0.500865
Sum of electronic and thermal Enthalpies= -0.499921
Sum of electronic and thermal Free Energies= -0.512935

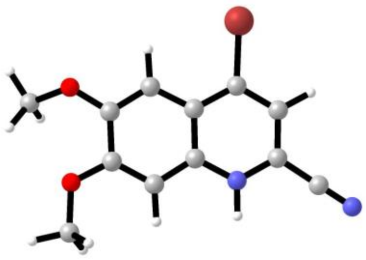

Zero-point correction= 0.200698 (Hartree/Particle)
Thermal correction to Energy= 0.216635
Thermal correction to Enthalpy= 0.217579
Thermal correction to Gibbs Free Energy= 0.155740
Sum of electronic and zero-point Energies= -3297.176261
Sum of electronic and thermal Energies= -3297.160324
Sum of electronic and thermal Enthalpies= -3297.159380
Sum of electronic and thermal Free Energies= -3297.221219

Charge = 1 Multiplicity = 1

C	-4.55896	3.58477	0.77081
C	-3.18616	3.58477	0.77081
C	-2.46422	4.80989	0.77081
C	-3.18209	6.03376	0.77118
C	-4.6035	6.00438	0.77134
C	-5.27501	4.80641	0.77105
H	-2.62137	2.64045	0.77059
C	-2.45934	7.25848	0.7713
H	-5.15074	6.95884	0.77142
C	-1.08648	7.25855	0.77103
C	-0.37046	6.03666	0.77057
H	-0.52578	8.20488	0.77085
N	-1.04249	4.83943	0.77055
O	-6.7046	4.77213	0.77118
O	-5.28791	2.35451	0.77071
C	-4.80125	1.51123	1.81806
H	-5.52359	0.75026	2.02796
H	-3.88254	1.05619	1.51177
H	-4.63349	2.09624	2.69812
C	-7.17063	4.28994	-0.49183
H	-7.56712	3.30328	-0.37267
H	-7.93623	4.93978	-0.86125
H	-6.35726	4.26597	-1.18663
Br	-3.43913	8.89803	0.77175

C	1.16909	6.07371	0.77031
N	2.31536	6.1013	0.77012
H	-0.33497	4.19219	-0.1137

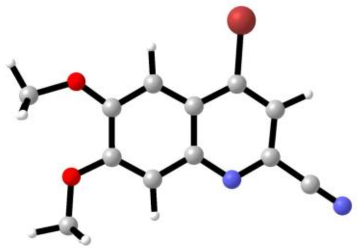

Zero-point correction= 0.186880 (Hartree/Particle)
Thermal correction to Energy= 0.202500
Thermal correction to Enthalpy= 0.203444
Thermal correction to Gibbs Free Energy= 0.142505
Sum of electronic and zero-point Energies= -3296.530351
Sum of electronic and thermal Energies= -3296.514732
Sum of electronic and thermal Enthalpies= -3296.513787
Sum of electronic and thermal Free Energies= -3296.574727

Charge = 1 Multiplicity = 2

C	-2.08216	-4.75377	-0.00037
C	-0.70936	-4.75377	-0.00037
C	0.01258	-3.52865	-0.00037
C	-0.70529	-2.30479	0.
C	-2.12671	-2.33416	0.00015
C	-2.79821	-3.53213	-0.00014
H	-0.14457	-5.69809	-0.0006
C	1.43431	-3.49911	-0.00063
H	-2.67395	-1.3797	0.00024
C	1.39032	-1.07999	-0.00016
C	2.10633	-2.30188	-0.00062
H	3.20596	-2.27542	-0.0008
N	0.01746	-1.08006	0.00012
O	-2.81111	-5.98403	-0.00047
O	-4.2278	-3.56641	0.
C	-3.46001	-6.15428	-1.26334
H	-3.31649	-7.15783	-1.60567
H	-4.50678	-5.9605	-1.15548
H	-3.04232	-5.4719	-1.97383

C	-4.72526	-2.72928	1.0472
H	-4.67202	-1.7054	0.74105
H	-5.74255	-2.9865	1.25662
H	-4.13342	-2.86955	1.92752
Br	2.38443	-5.15602	-0.0006
C	2.17532	0.24492	-0.00041
N	2.75978	1.23137	-0.00059

6. References

1. Kumar, A.; Singh, S. K.; Sharma, C., Esterification of fluorinated aromatic carboxylic acids with methanol by using UiO-66-NH2 as a heterogeneous catalyst and process optimization by the Taguchi method. *RSC Adv.* **2023,** *13*, 16712-16723.
2. Yamashita, K.; Fujiwara, Y.; Hamashima, Y., Amide-Ligand-Promoted Silver-Catalyzed C–H Fluorination via Radical/Polar Crossover. *J. Org. Chem,* **2023,** *88*, 1865-1874.
3. Kleban, I.; Radchenko, D. S.; Tymtsunik, A. V.; Shuvakin, S.; Konovets, A. I.; Rassukana, Y.; Grygorenko, O. O., Cyclopropyl boronic derivatives in parallel synthesis of sp3-enriched compound libraries. Monatsh. Chem, **2020,** *151*, 953-962.
4. Maslak, P.; Chapman, W. H., Jr., Photochemically generated ion and radical pairs. Self-destructive charge-transfer complexes. *J. Org. Chem,* **1990,** *55*, 6334-6347.
5. Xue, T.; Zhang, Z.; Zeng, R., Photoinduced Ligand-to-Metal Charge Transfer (LMCT) of Fe Alkoxide Enabled C–C Bond Cleavage and Amination of Unstrained Cyclic Alcohols. *Org. Lett* **2022,** *24*, 977-982.
6. Redwan, I. N.; Grøtli, M., Method for Activation and Recycling of Trityl Resins. *J. Org. Chem,* **2012,** *77*, 7071-7075.
7. Hu, H.; Chen, S.-J.; Mandal, M.; Pratik, S. M.; Buss, J. A.; Krska, S. W.; Cramer, C. J.; Stahl, S. S., Copper-catalysed benzylic C–H coupling with alcohols via radical relay enabled by redox buffering. *Nat. Catal.* **2020,** *3*, 358-367.
8. Cosp, A.; Romea, P.; Urpí, F.; Vilarrasa, J., Enantiopure β-methoxy carboxyl derivatives from a chiral titanium enolate and dimethyl acetals. *Tetrahedron Lett.* **2001,** *42*, 4629-4631.
9. Shigehisa, H.; Aoki, T.; Yamaguchi, S.; Shimizu, N.; Hiroya, K., Hydroalkoxylation of Unactivated Olefins with Carbon Radicals and Carbocation Species as Key Intermediates. *J. Am. Chem. Soc.* **2013,** *135*, 10306-10309.
10. Sahoo, P. K.; Gawali, S. S.; Gunanathan, C., Iron-Catalyzed Selective Etherification and Transetherification Reactions Using Alcohols. *ACS Omega* **2018,** *3*, 124-136.
11. Kurosawa, H.; Okada, H.; Hattori, T., Electron-transfer chain-substitution in hydrodemercuration of alkylmercury(II) compounds with n-benzyl-1,4-dihydronicotinamide. *Tetrahedron Lett.* **1981,** *22*, 4495-4498.
12. Penner, A.; Bätzner, E.; Wagenknecht, H.-A., Chemical Photocatalysis with 1-(N,N-Dimethylamino)pyrene. *Synlett* **2012,** *23*, 2803-2807.
13. Muramatsu, W.; Nakano, K.; Li, C.-J., Simple and Direct sp3 C–H Bond Arylation of Tetrahydroisoquinolines and Isochromans via 2,3-Dichloro-5,6-dicyano-1,4-benzoquinone Oxidation under Mild Conditions. *Org. Lett.* **2013,** *15*, 3650-3653.
14. Seyfert, F.; Wagenknecht, H.-A., N-Arylbenzo[b]phenothiazines as Reducing Photoredox Catalysts for Nucleophilic Additions of Alcohols to Styrenes: Shift towards Visible Light. *Synlett* **2021,** *32*, 582-586.
15. Mitsuhashi, T., Facile heterolysis of a carbon-carbon bond. Arylazodicyanomethanides as the leaving group capable of generating tert-cumyl cation and the hydrogen-bond-insusceptible behavior of the leaving group anions. *J. Am. Chem. Soc.* **1986,** *108*, 2394-2400.

16. Boyer, B.; Keramane, E.-M.; Roque, J.-P.; Pavia, A. A., BiBr3, an efficient catalyst for the benzylation of alcohols: 2-phenyl-2-propyl, a new benzyl-type protecting group. *Tetrahedron Lett.* **2000,** *41*, 2891-2894.

17. Cullen, A.; Muller, A. J.; Williams, D. B. G., Protecting group-free use of alcohols as carbon electrophiles in atom efficient aluminium triflate-catalysed dehydrative nucleophilic displacement reactions. *RSC Adv.* **2017,** *7*, 42168-42171.

18. Meng, S.-S.; Wang, Q.; Huang, G.-B.; Lin, L.-R.; Zhao, J.-L.; Chan, A. S. C., B(C6F5)3 catalyzed direct nucleophilic substitution of benzylic alcohols: an effective method of constructing C–O, C–S and C–C bonds from benzylic alcohols. *RSC Adv.* **2018,** *8*, 30946-30949.

19. Li, J.; Zhang, X.; Shen, H.; Liu, Q.; Pan, J.; Hu, W.; Xiong, Y.; Chen, C., Boron Trifluoride·Diethyl Ether-Catalyzed Etherification of Alcohols: A Metal-Free Pathway to Diphenylmethyl Ethers. *Adv. Synth. Catal.* **2015,** *357*, 3115-3120.

20. Wanka, L.; Cabrele, C.; Vanejews, M.; Schreiner, P. R., *Eur. J. Org. Chem.* **2007**, 1474-1490.

21. Xiang, J.; Shang, M.; Kawamata, Y.; Lundberg, H.; Reisberg, S. H.; Chen, M.; Mykhailiuk, P.; Beutner, G.; Collins, M. R.; Davies, A.; Del Bel, M.; Gallego, G. M.; Spangler, J. E.; Starr, J.; Yang, S.; Blackmond, D. G.; Baran, P. S., Hindered dialkyl ether synthesis with electrogenerated carbocations. *Nature* **2019,** *573*, 398-402.

22. Wu, Y. PDE9 INHIBITOR AND USE THEREOF [P]. US2021130366A1 . 2021-05-06.

23. Connelly, N. G.; Geiger, W. E. *Chem. Rev.* **1996**, *96*, 877.

24. Gaussian 09, Revision D.01, M. J. Frisch, G. W. Trucks, H. B. Schlegel, G. E. Scuseria, M. A. Robb, J. R. Cheeseman, G. Scalmani, V. Barone, B. Mennucci, G. A. Petersson, H. Nakatsuji, M. Caricato, X. Li, H. P. Hratchian, A. F. Izmaylov, J. Bloino, G. Zheng, J. L. Sonnenberg, M. Hada, M. Ehara, K. Toyota, R. Fukuda, J. Hasegawa, M. Ishida, T. Nakajima, Y. Honda, O. Kitao, H. Nakai, T. Vreven, J. A. Montgomery, Jr., J. E. Peralta, F. Ogliaro, M. Bearpark, J. J. Heyd, E. Brothers, K. N. Kudin, V. N. Staroverov, T. Keith, R. Kobayashi, J. Normand, K. Raghavachari, A. Rendell, J. C. Burant, S. S. Iyengar, J. Tomasi, M. Cossi, N. Rega, J. M. Millam, M. Klene, J. E. Knox, J. B. Cross, V. Bakken, C. Adamo, J. Jaramillo, R. Gomperts, R. E. Stratmann, O. Yazyev, A. J. Austin, R. Cammi, C. Pomelli, J. W. Ochterski, S35 R. L. Martin, K. Morokuma, V. G. Zakrzewski, G. A. Voth, P. Salvador, J. J. Dannenberg, S. Dapprich, A. D. Daniels, O. Farkas, J. B. Foresman, J. V. Ortiz, J. Cioslowski, and D. J. Fox, Gaussian, Inc., Wallingford CT, 2013.

25. CYLview, 1.0b; Legault, C. Y., Université de Sherbrooke, 2009 (http://www.cylview.org).

7. NMR Spectra

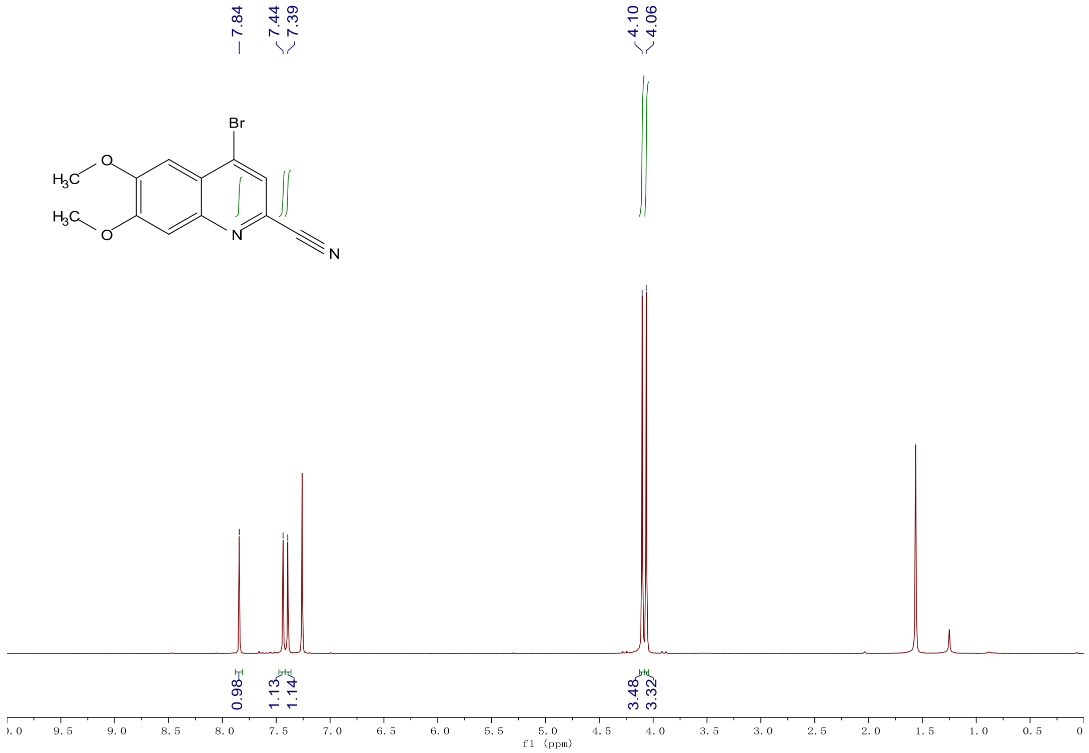

^{1}H NMR (400 MHz, $CDCl_3$) of catalyst **2**

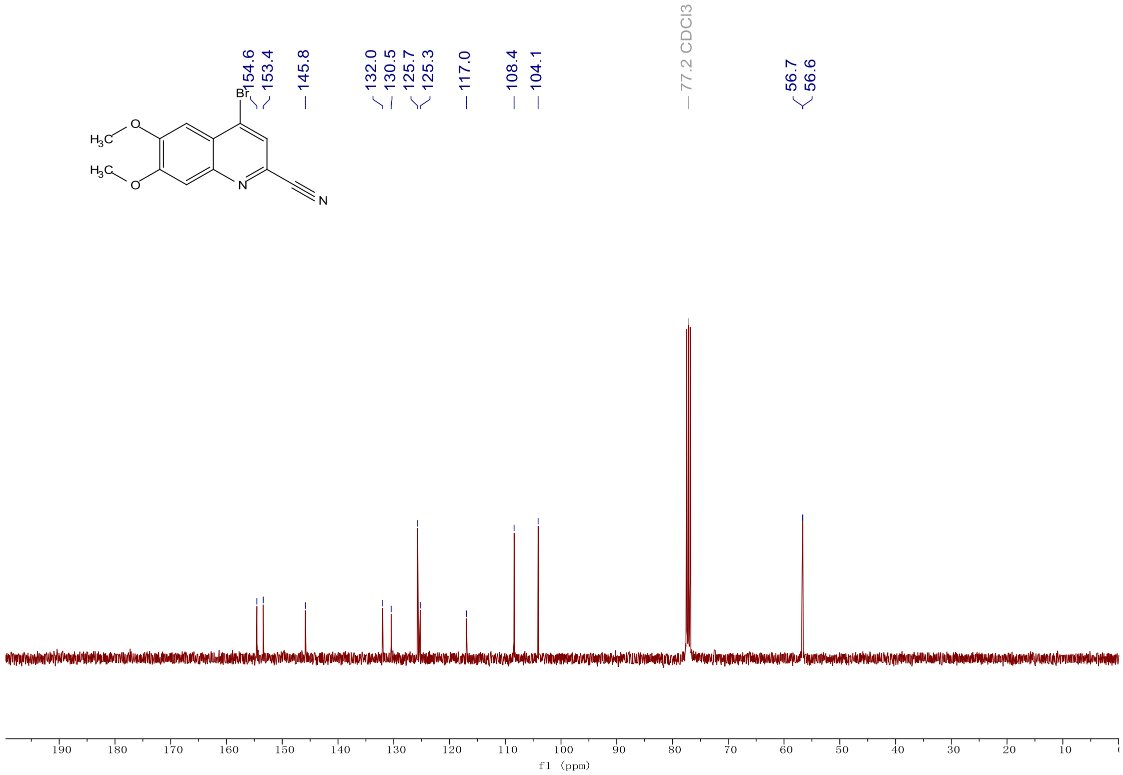

^{13}C NMR (101 MHz, $CDCl_3$) of catalyst **2**

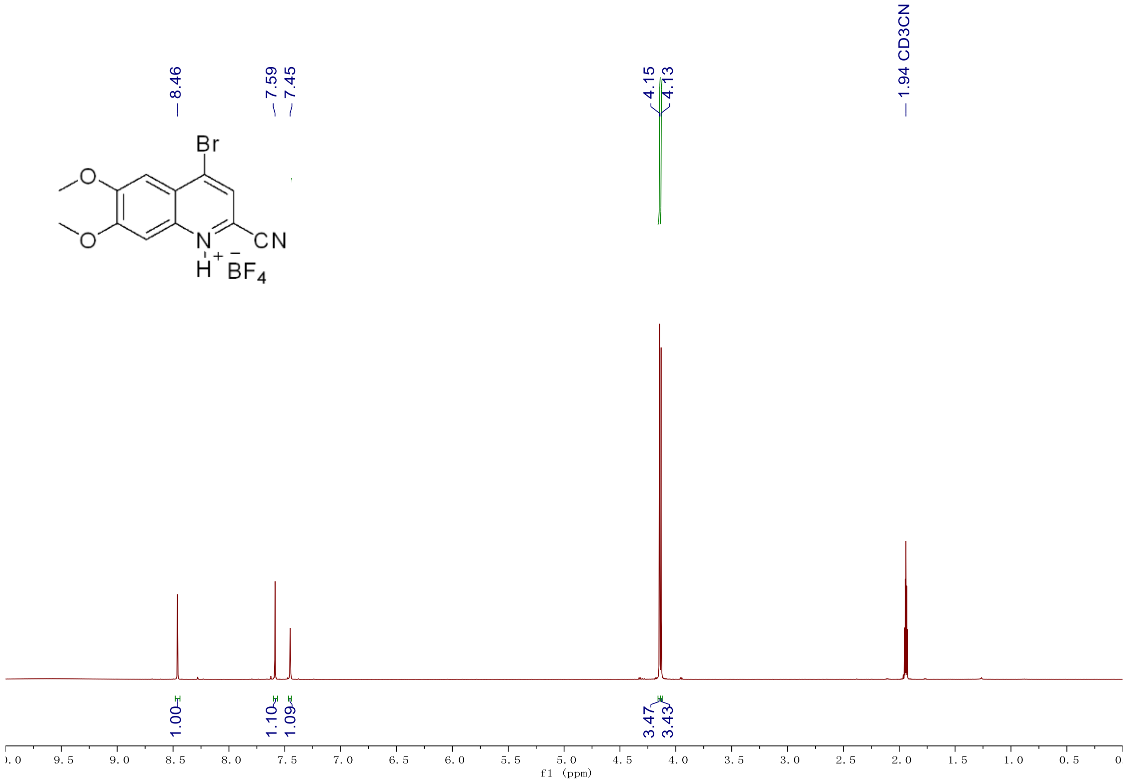

^{1}H NMR (400 MHz, CD_3CN) of catalyst **3**

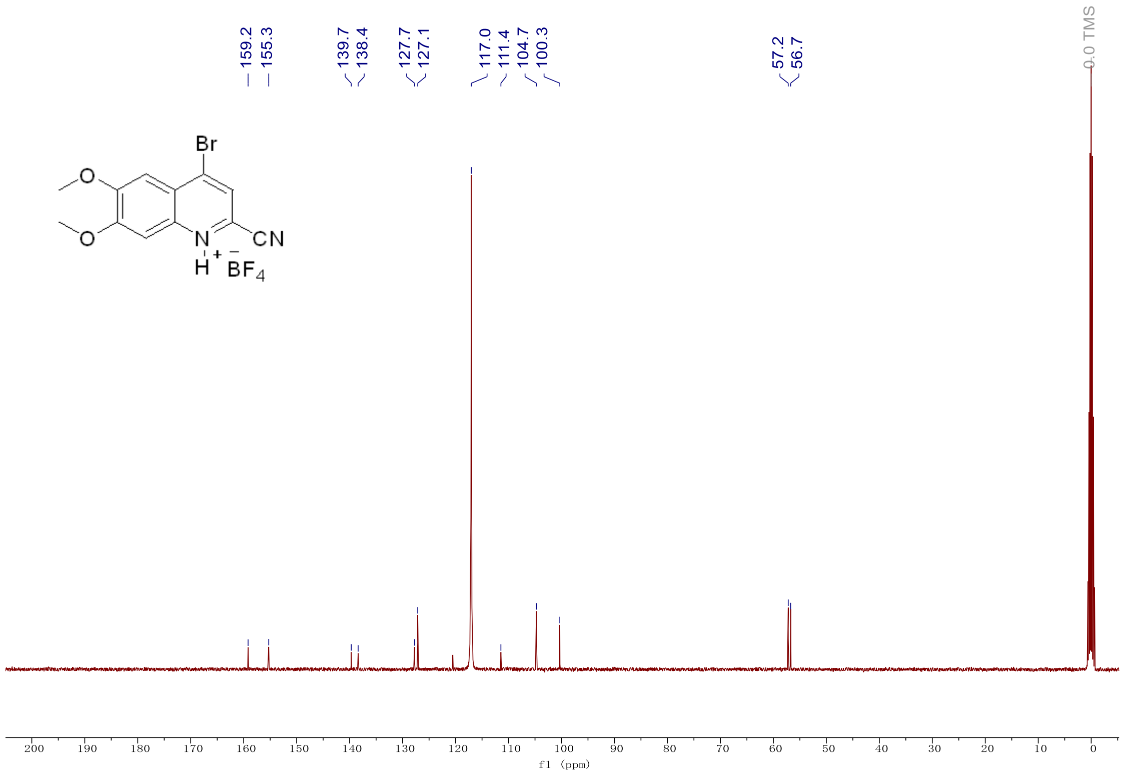

^{13}C NMR (101 MHz, CD_3CN) of catalyst **3**

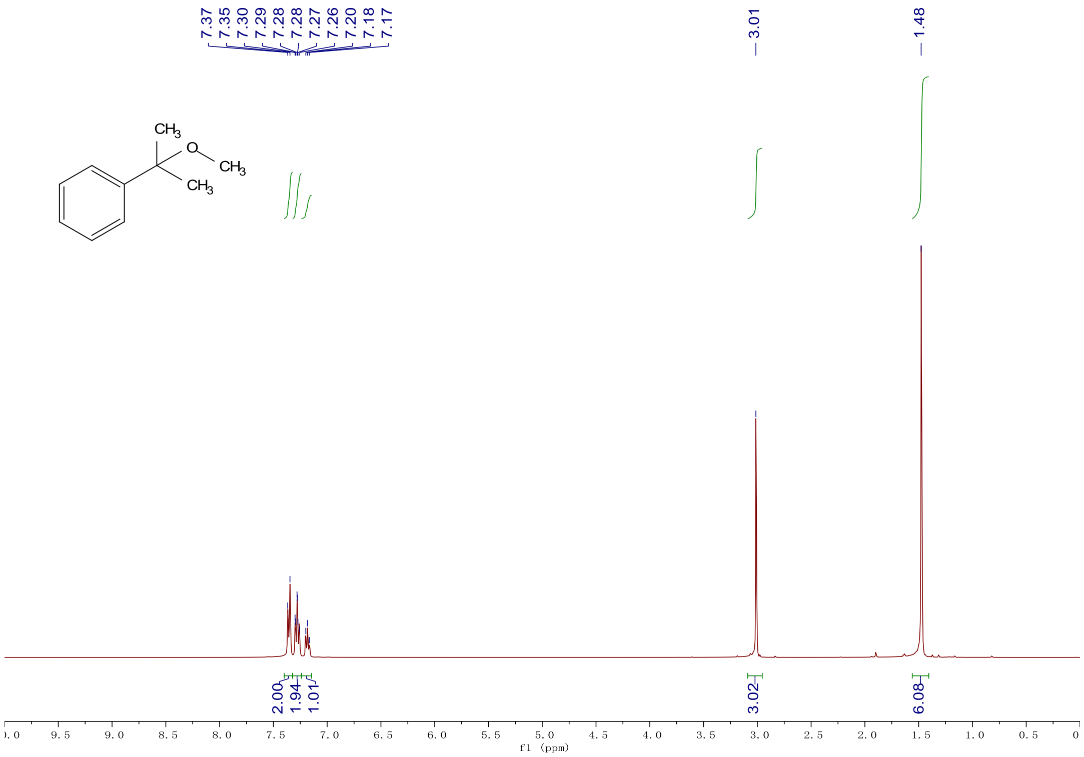

^{1}H NMR (400 MHz, $CDCl_3$) of **6a**

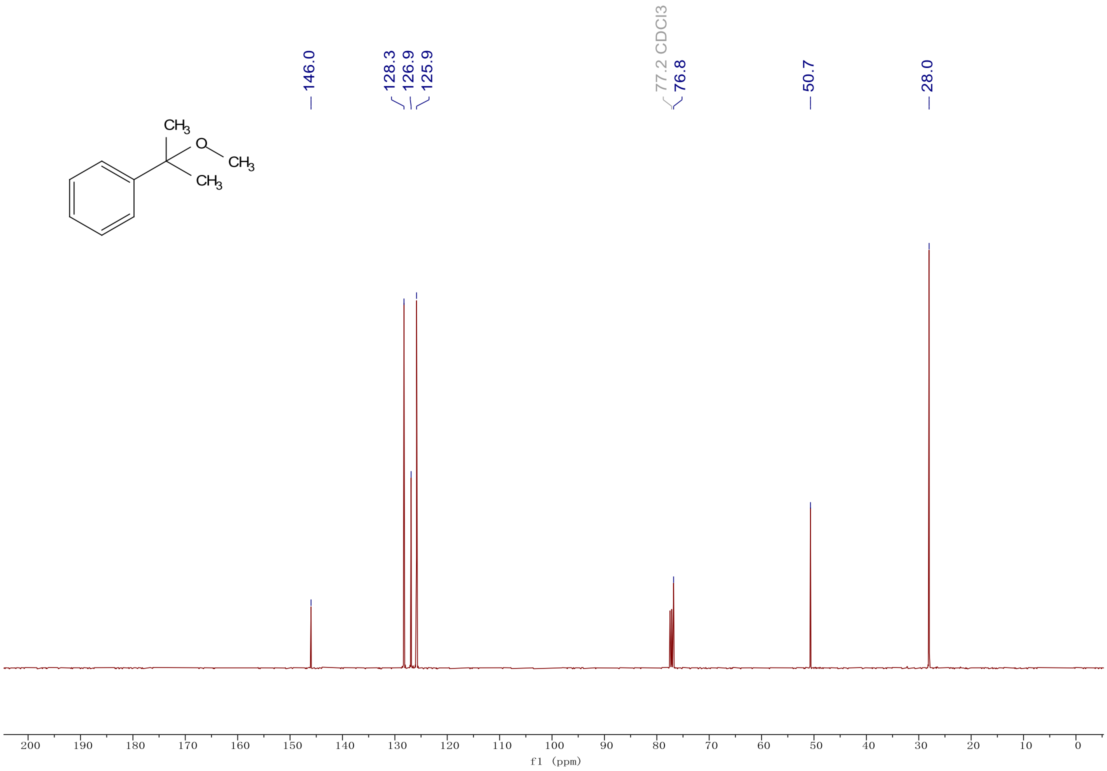

^{13}C NMR (101 MHz, $CDCl_3$) of **6a**

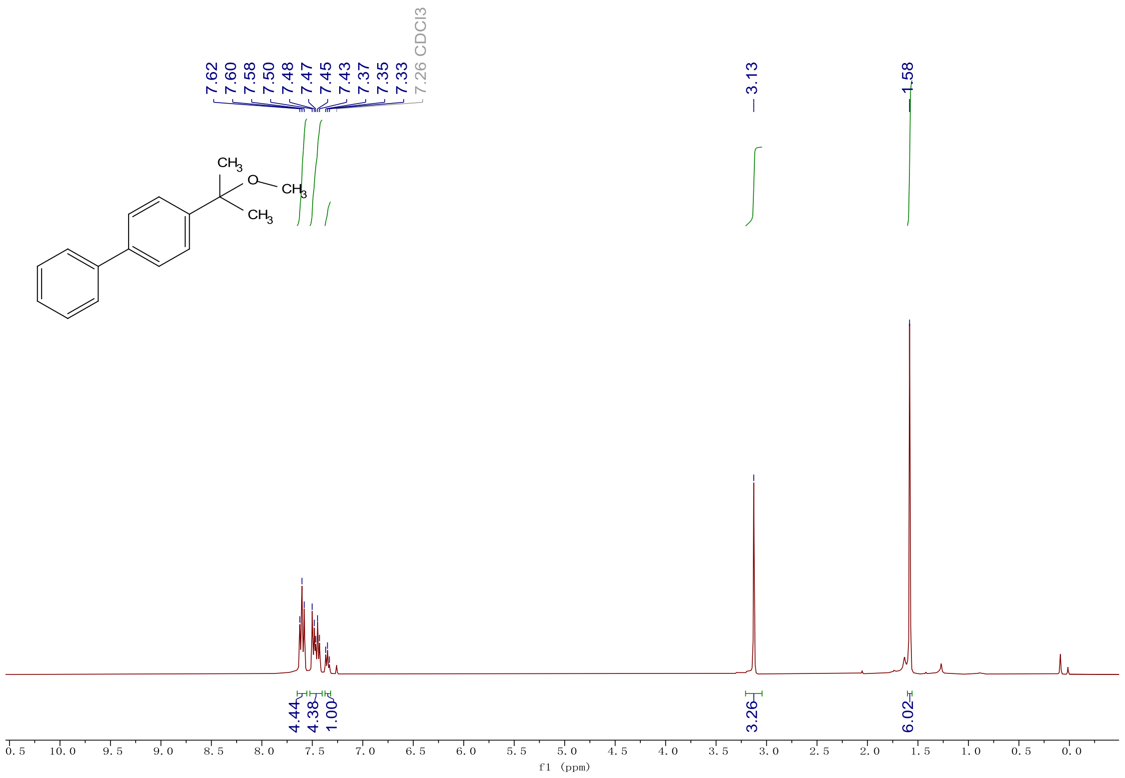

^1H NMR (400 MHz, $CDCl_3$) of **6b**

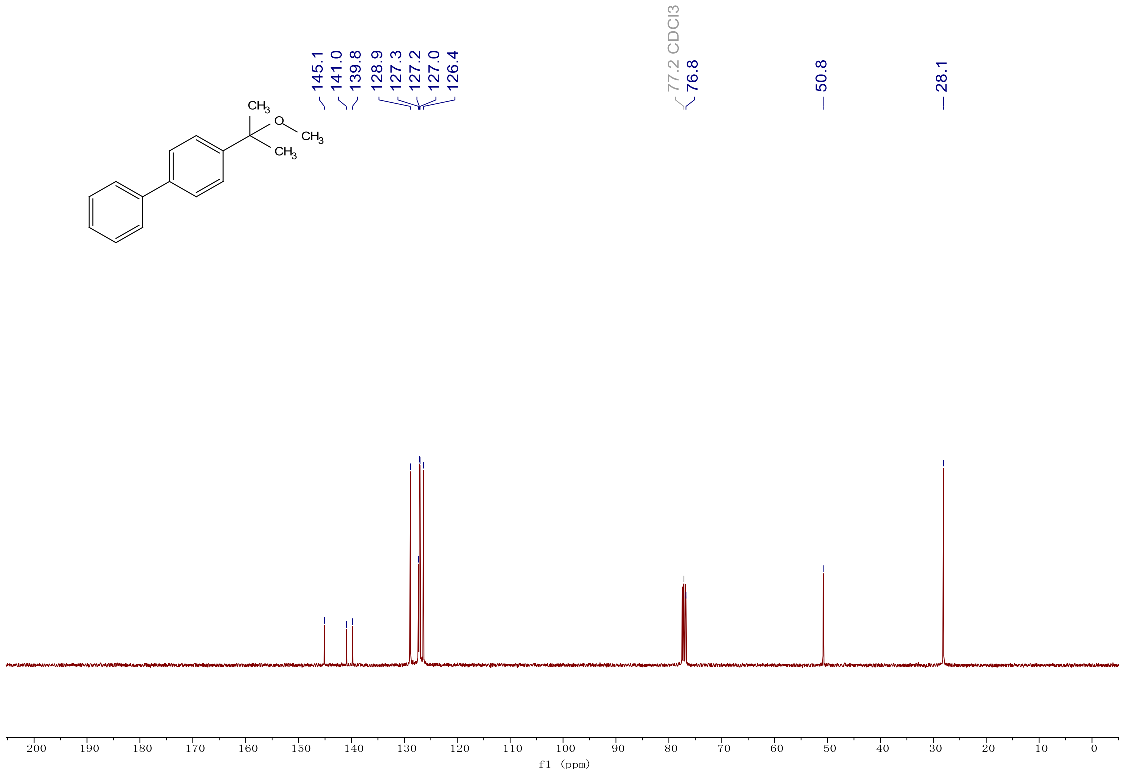

^{13}C NMR (101 MHz, $CDCl_3$) of **6b**

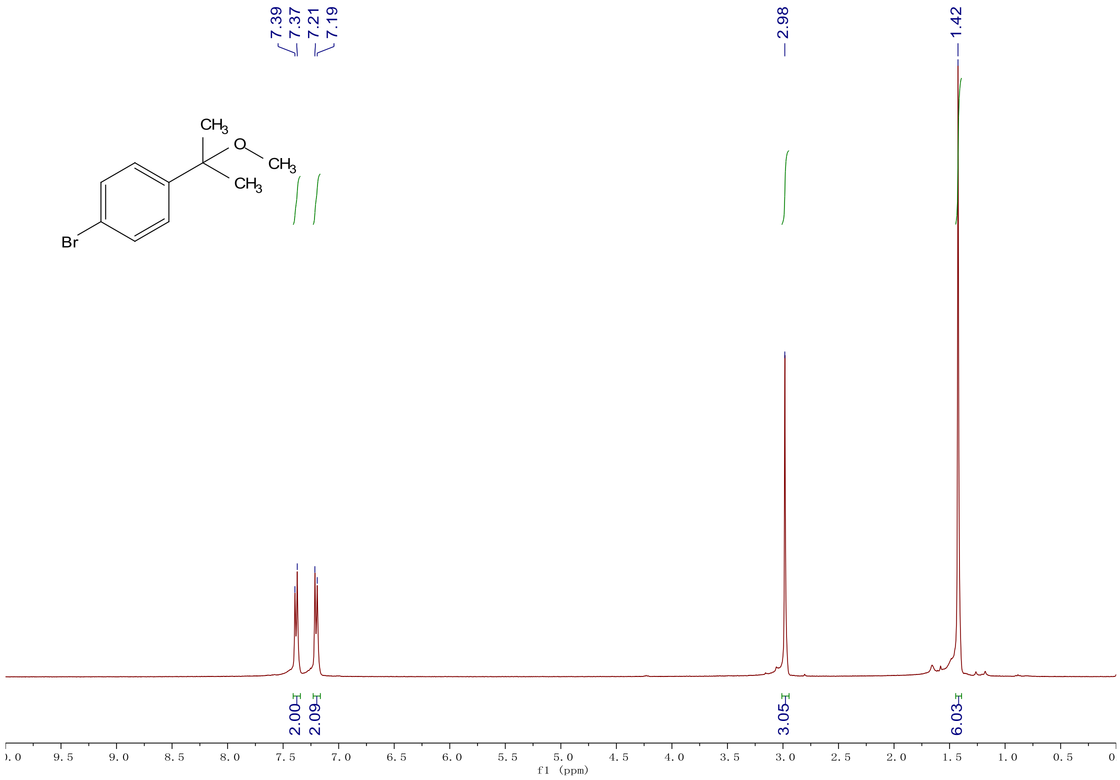

^{1}H NMR (400 MHz, $CDCl_3$) of **6c**

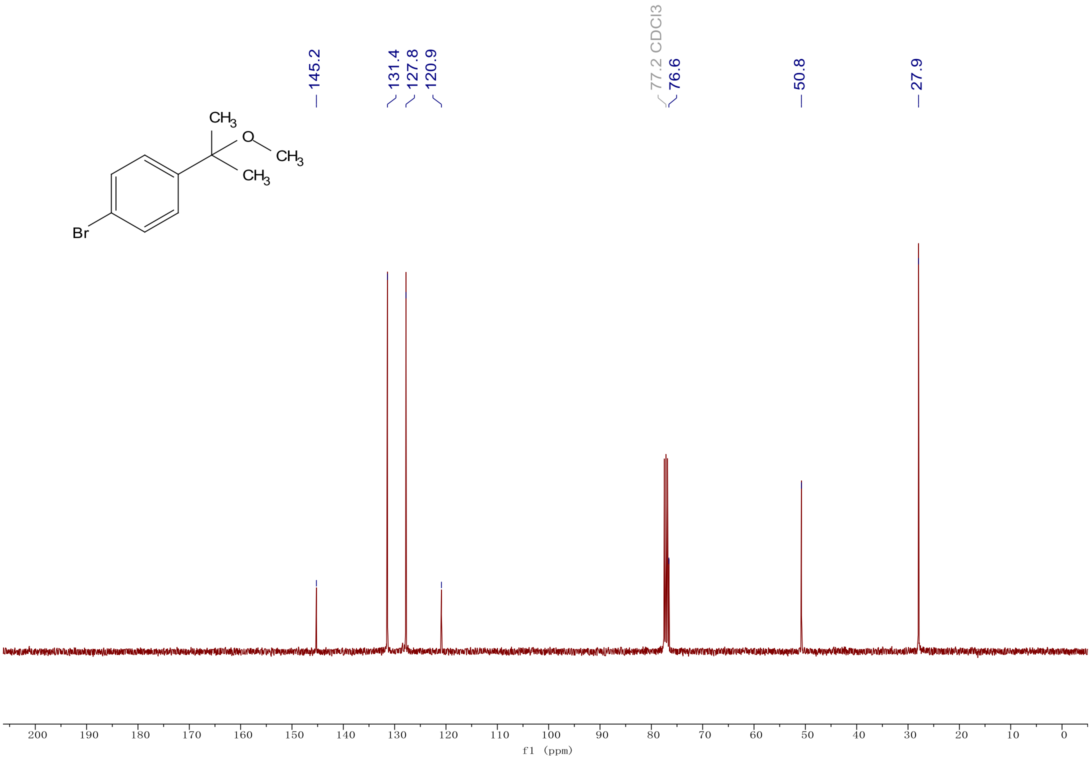

^{13}C NMR (101 MHz, $CDCl_3$) of **6c**

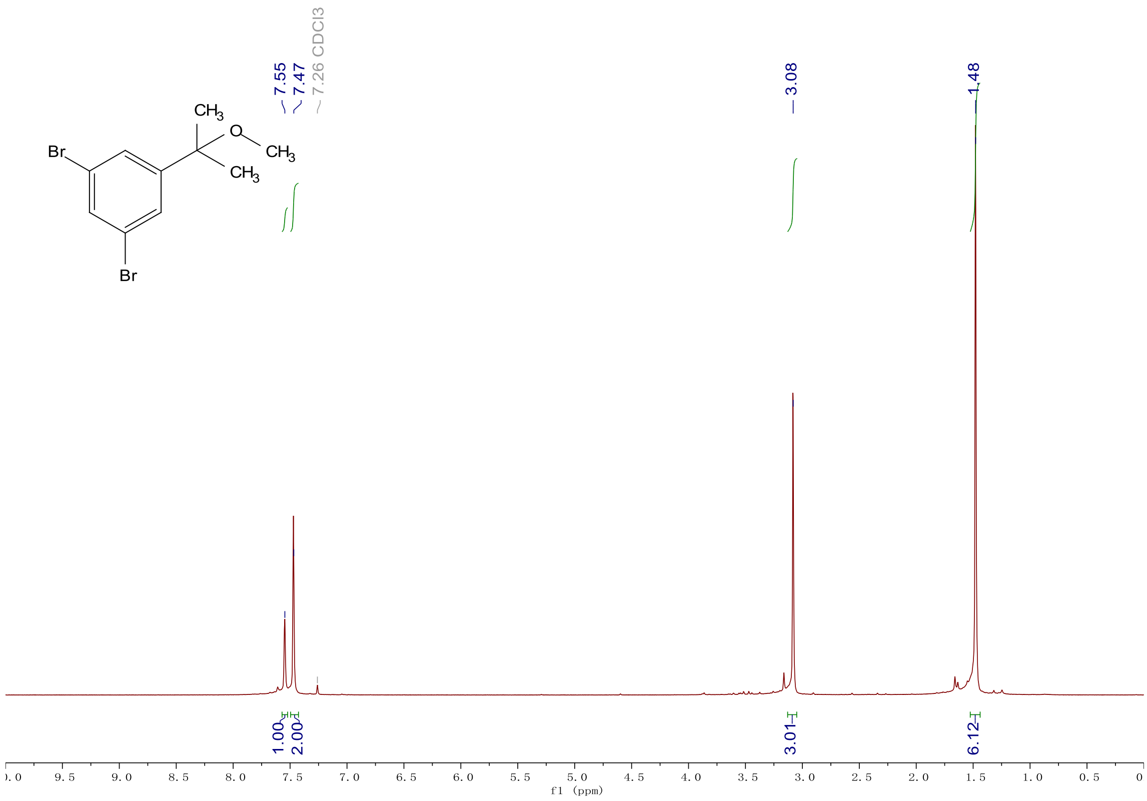

^{1}H NMR (400 MHz, $CDCl_3$) of **6d**

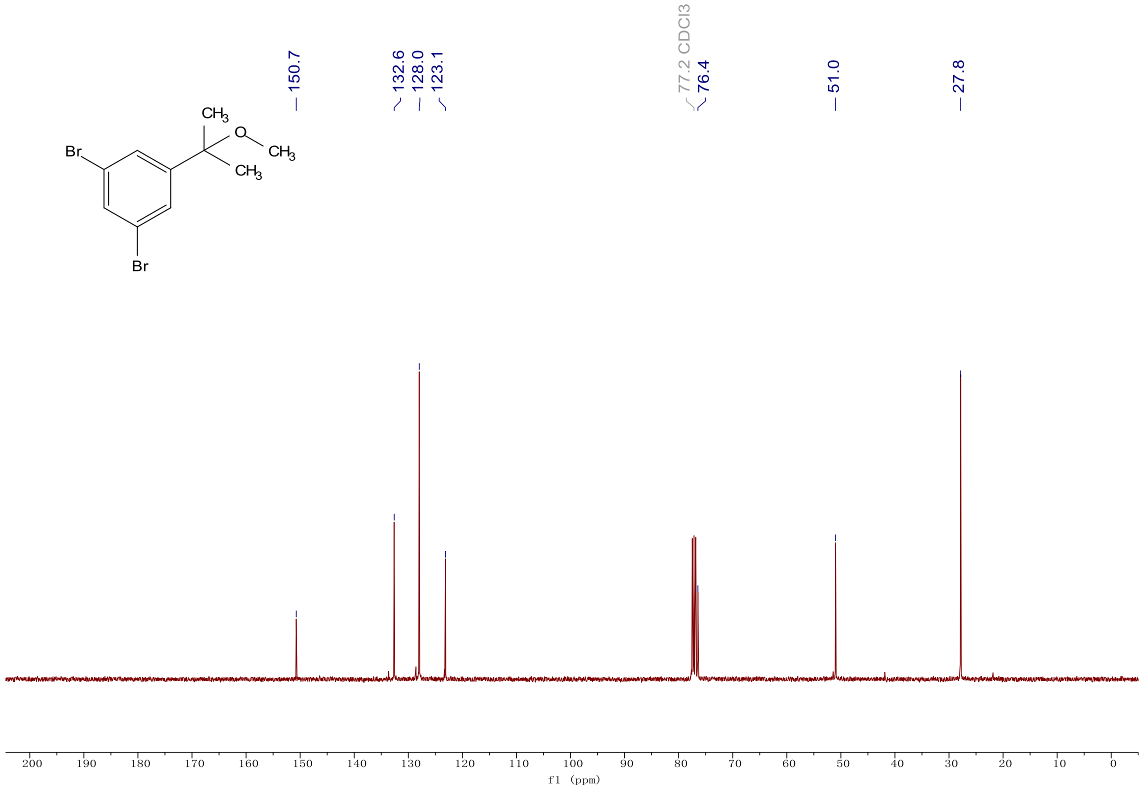

^{13}C NMR (101 MHz, $CDCl_3$) of **6d**

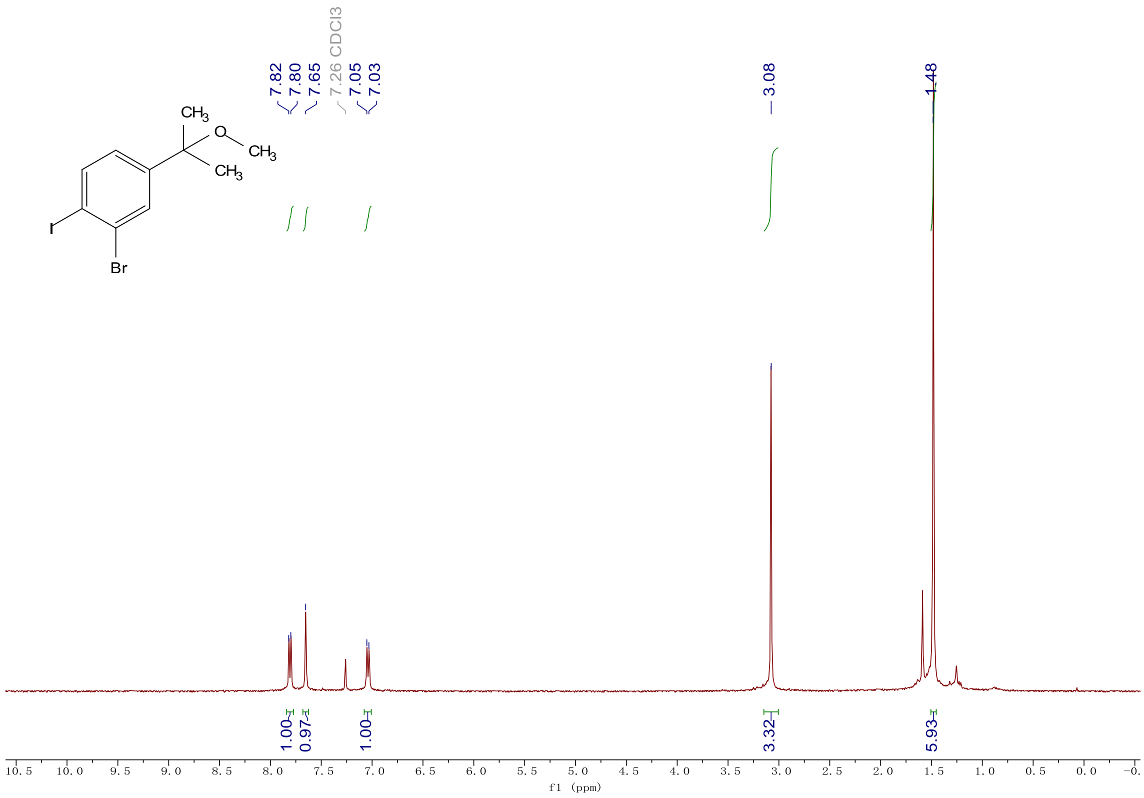

^{1}H NMR (400 MHz, $CDCl_3$) of **6e**

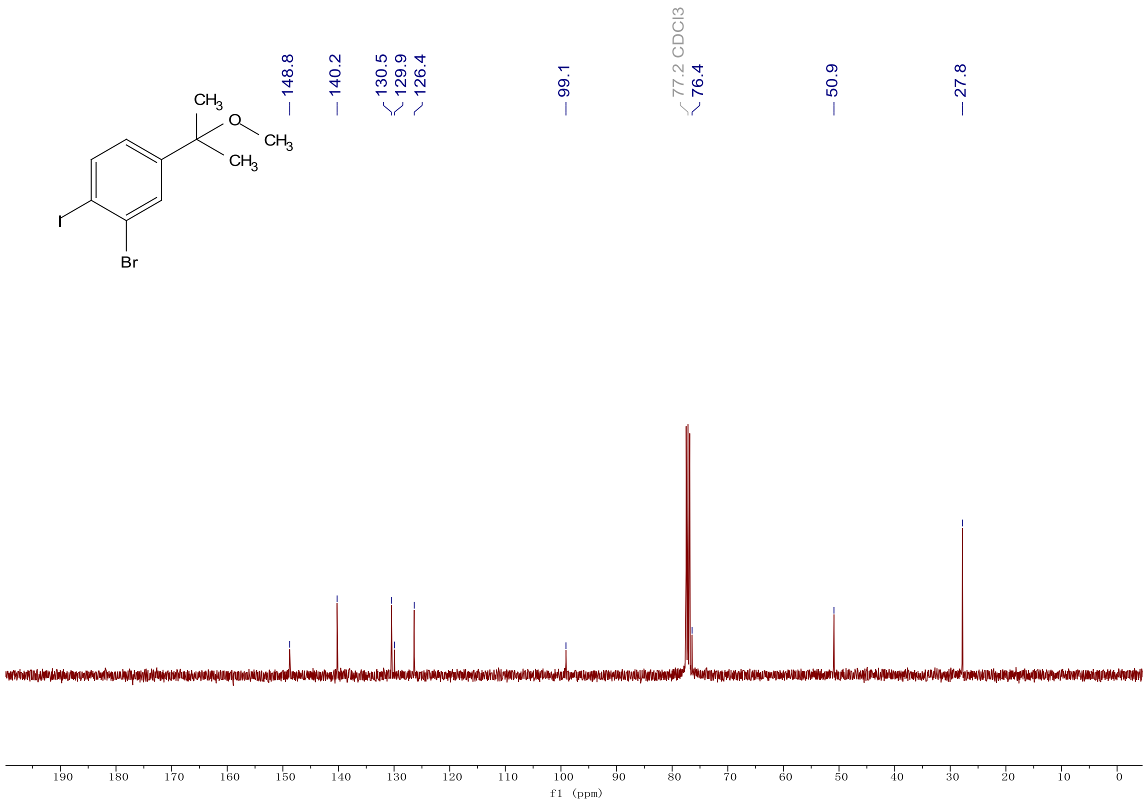

^{13}C NMR (101 MHz, $CDCl_3$) of **6e**

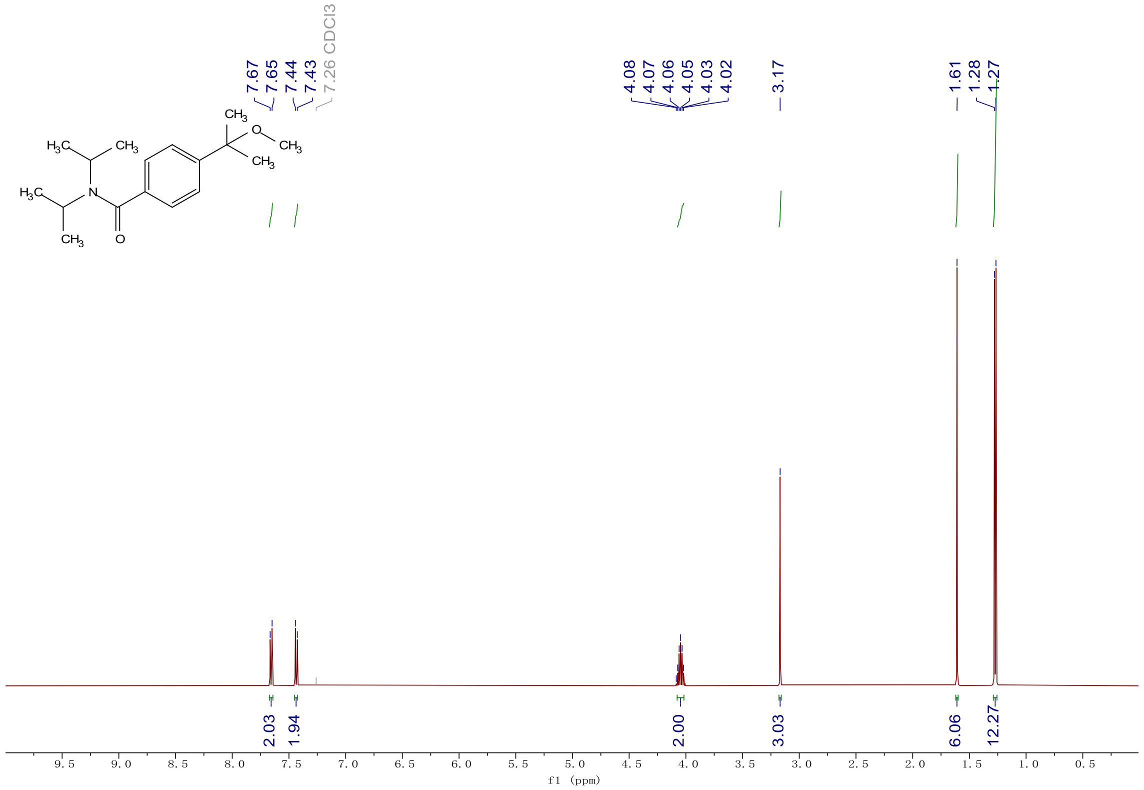

^{1}H NMR (400 MHz, $CDCl_3$) of **6f**

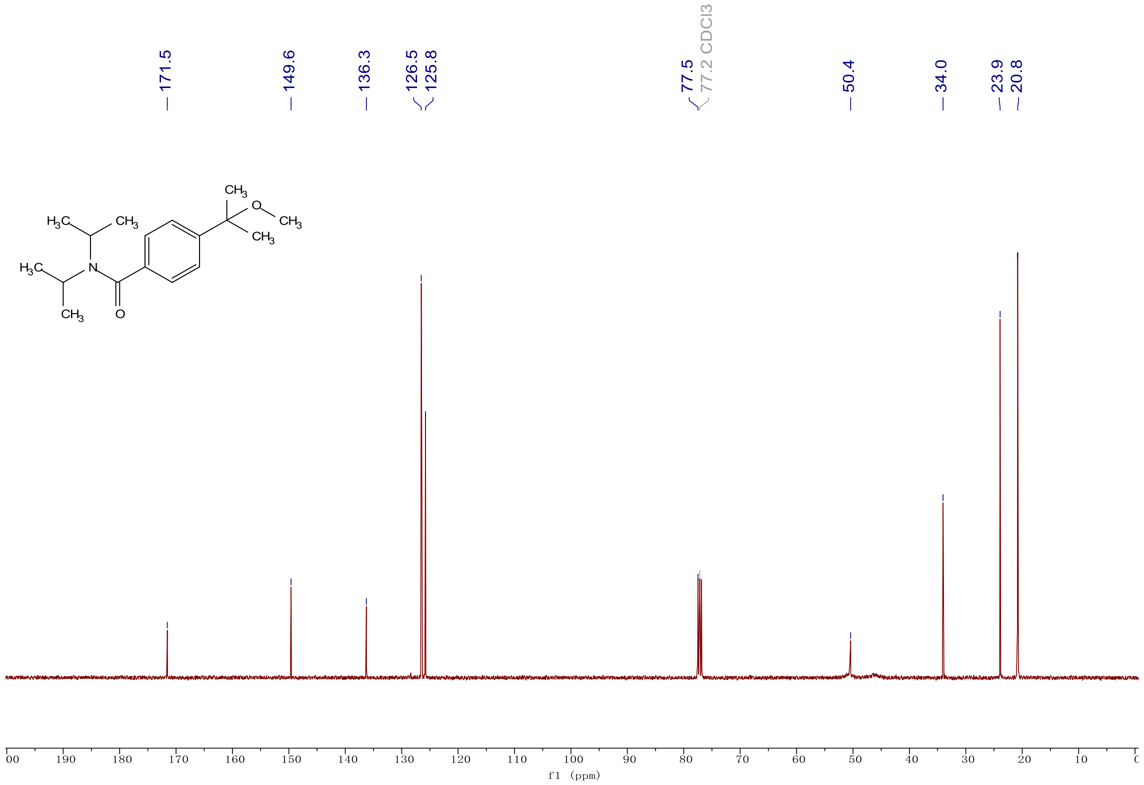

^{13}C NMR (101 MHz, $CDCl_3$) of **6f**

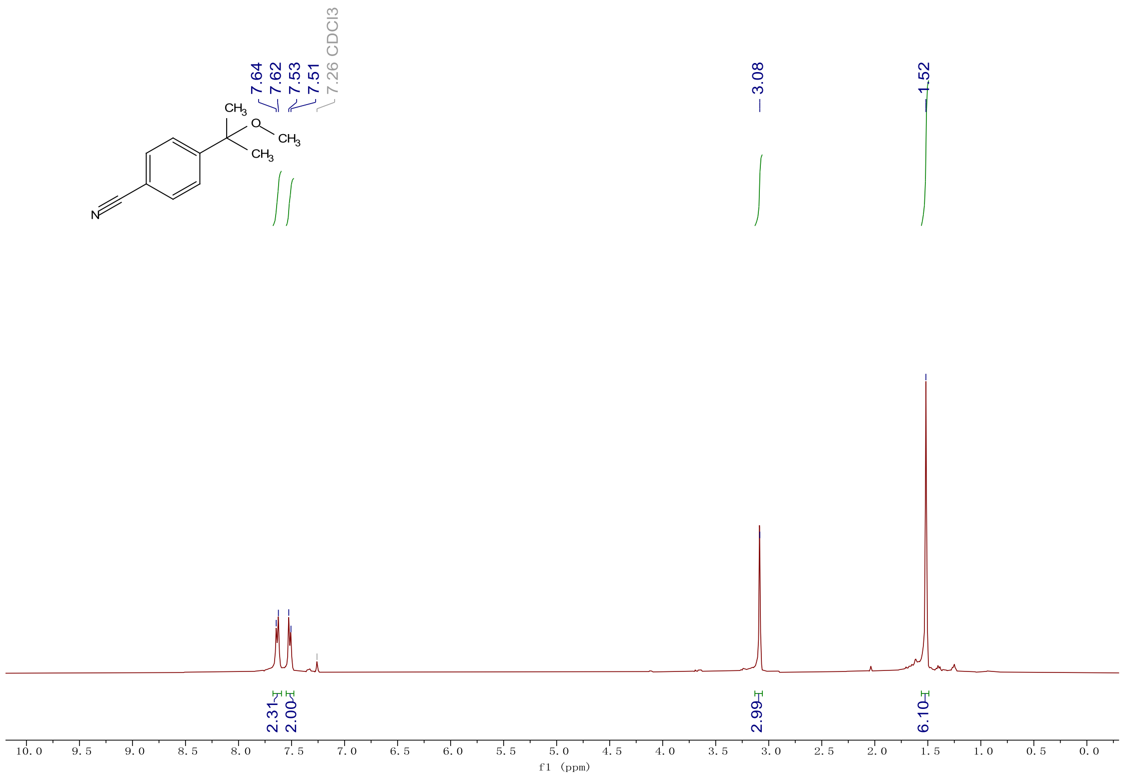

^{1}H NMR (400 MHz, $CDCl_3$) of **6g**

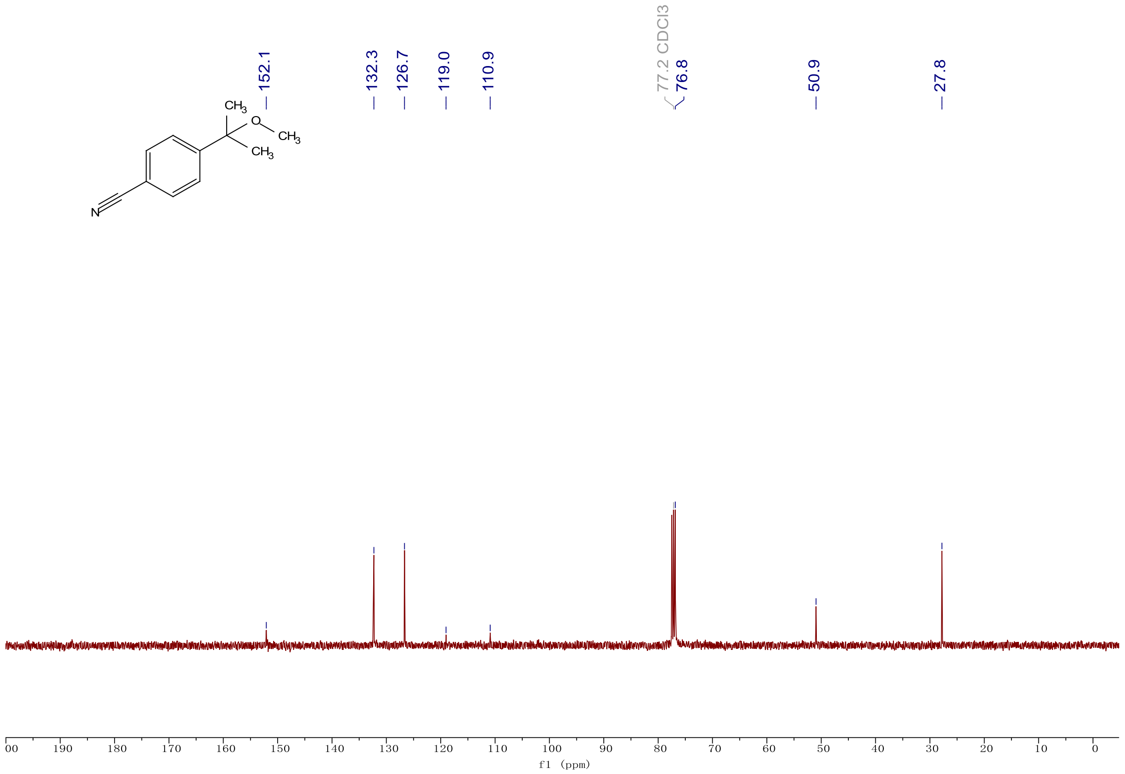

^{13}C NMR (101 MHz, $CDCl_3$) of **6g**

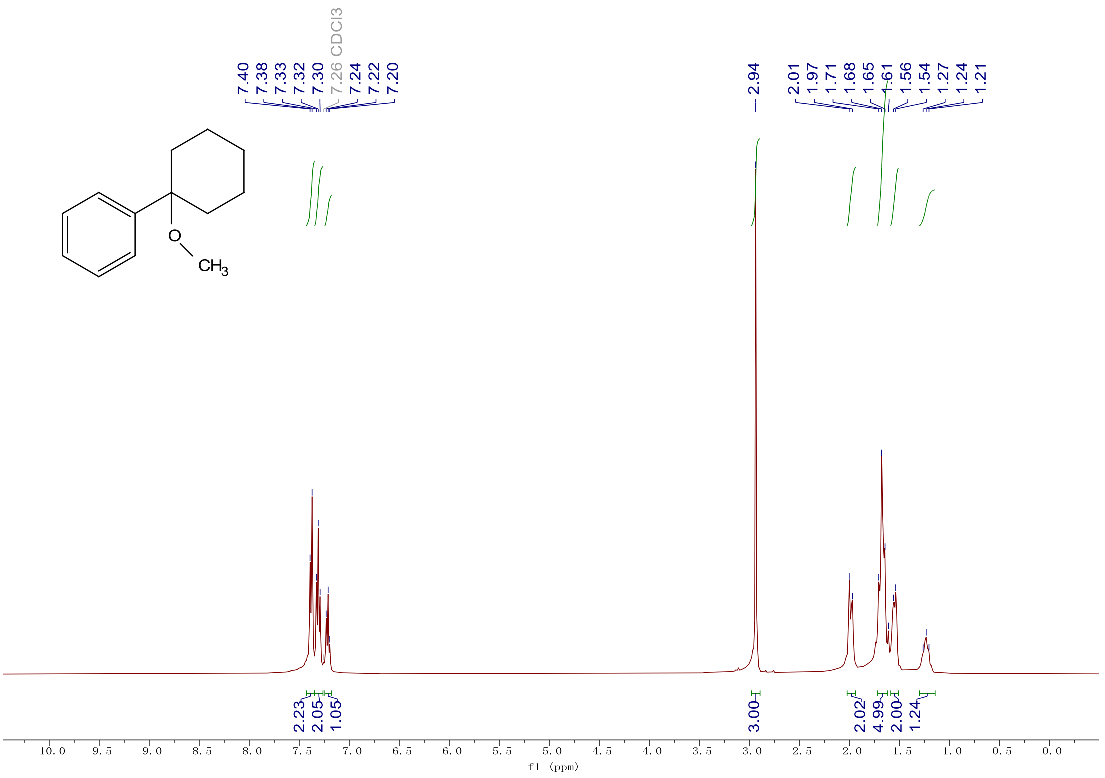

^{1}H NMR (400 MHz, $CDCl_3$) of **6h**

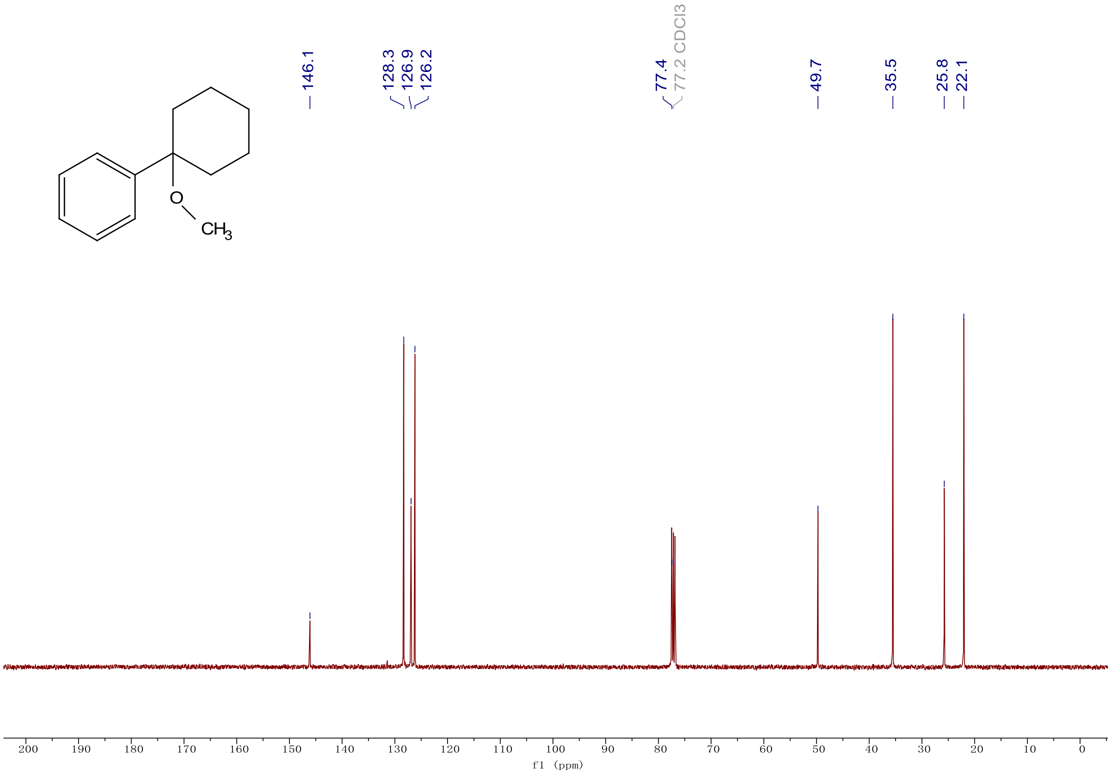

^{13}C NMR (101 MHz, $CDCl_3$) of **6h**

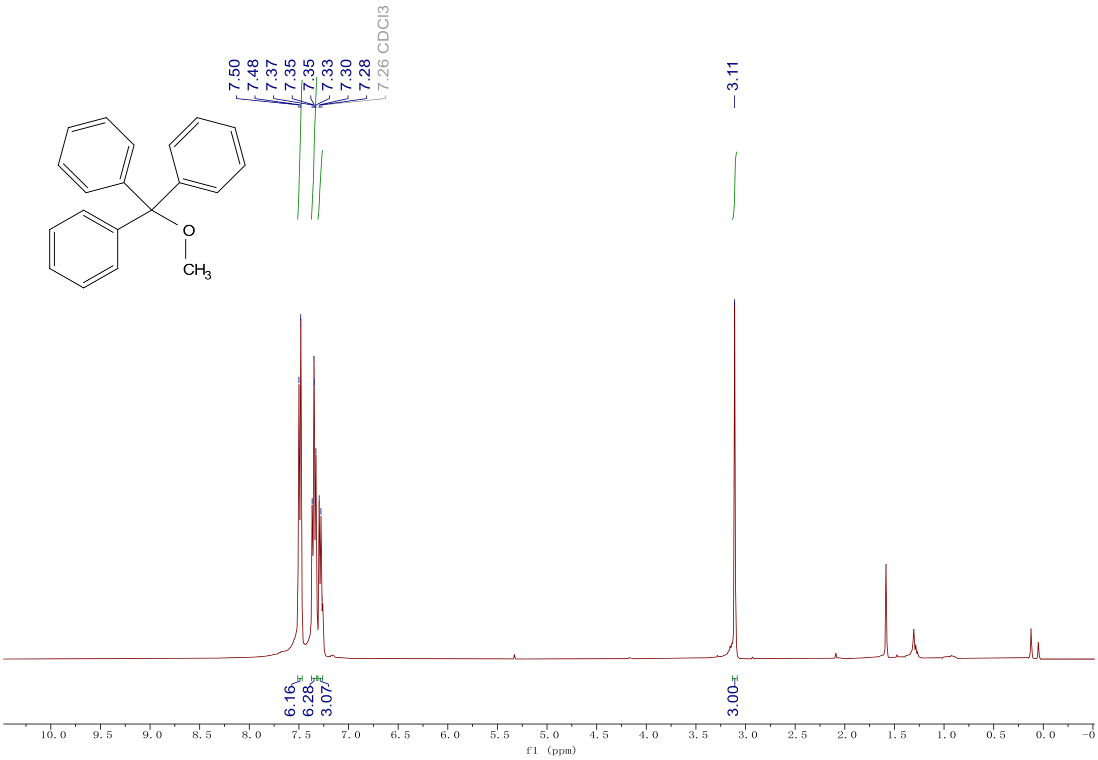

^{1}H NMR (400 MHz, $CDCl_3$) of **6i**

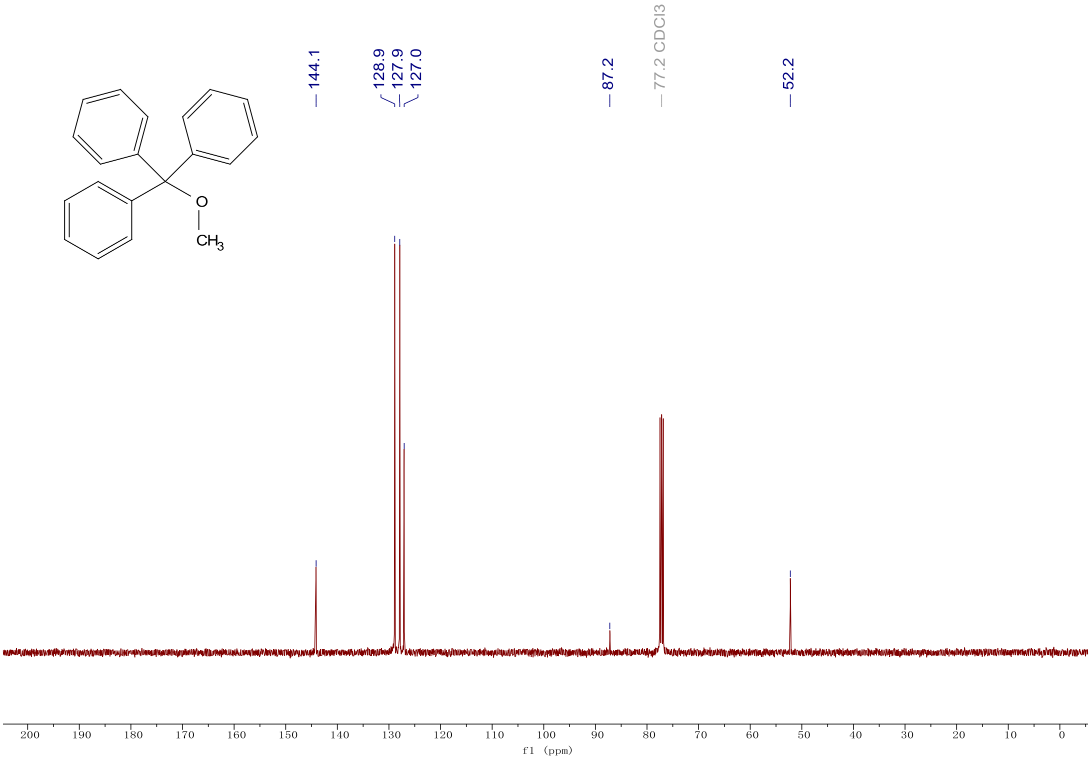

^{13}C NMR (101 MHz, $CDCl_3$) of **6i**

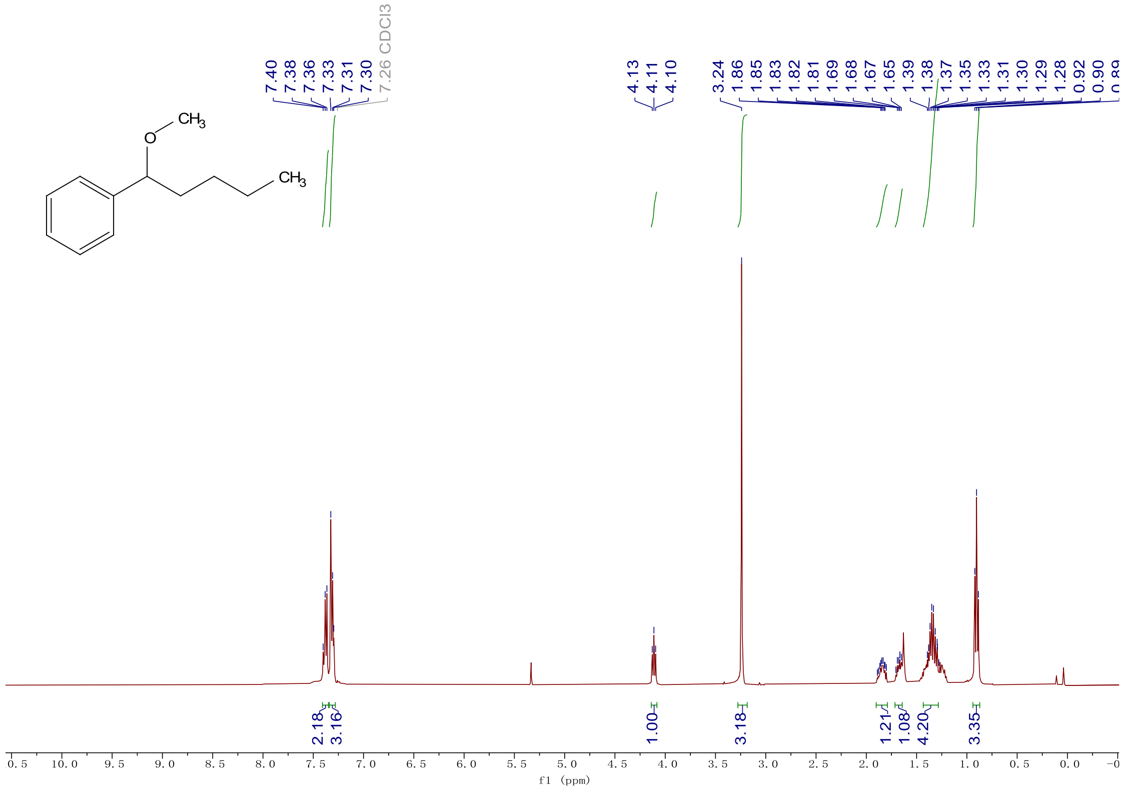

^{1}H NMR (400 MHz, $CDCl_3$) of **6k**

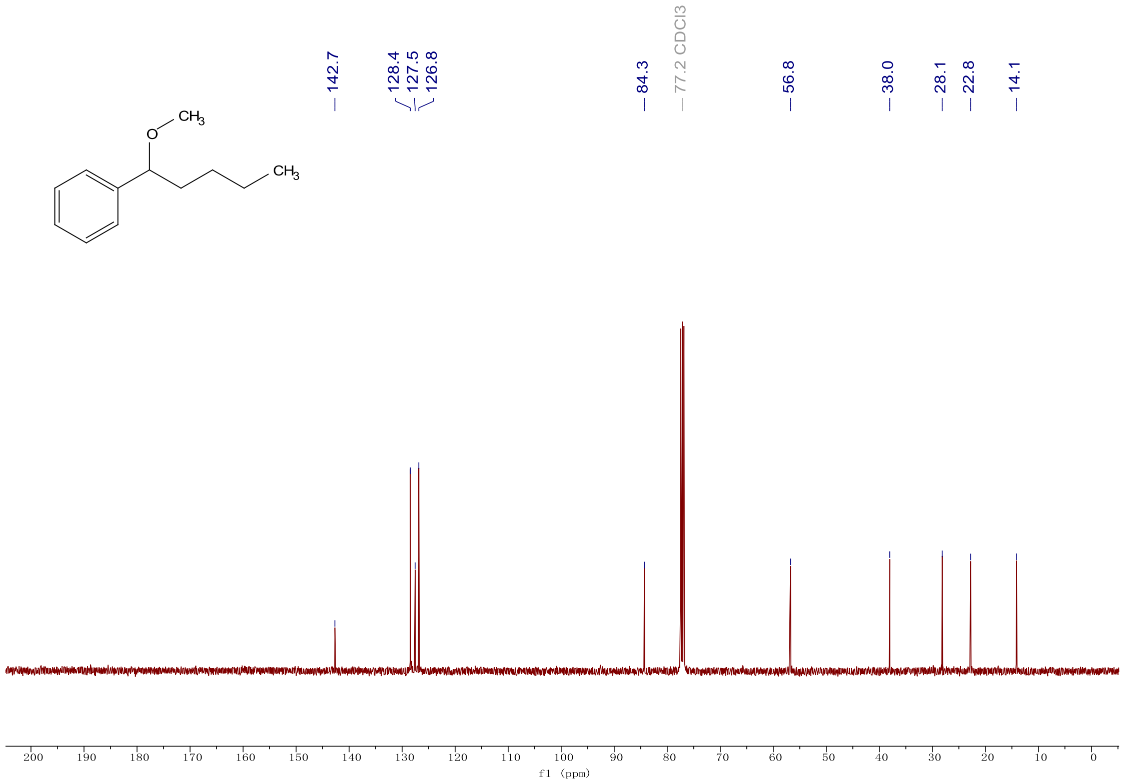

^{13}C NMR (101 MHz, $CDCl_3$) of **6k**

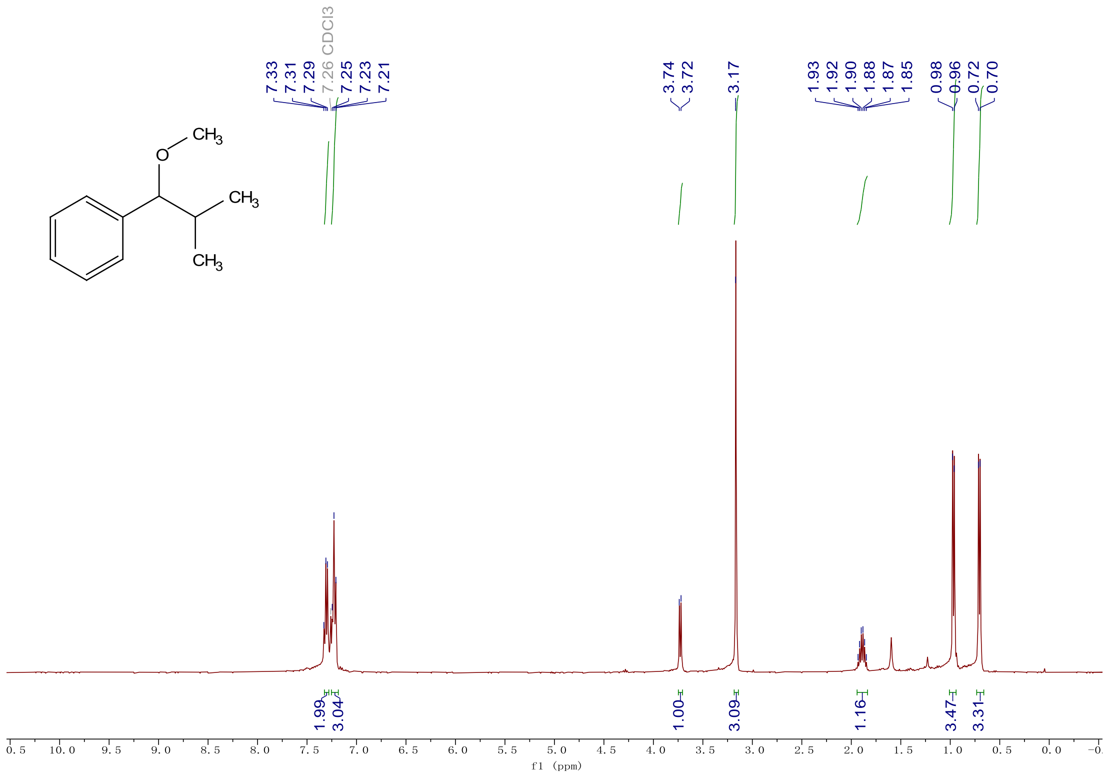

^{1}H NMR (400 MHz, $CDCl_3$) of **6l**

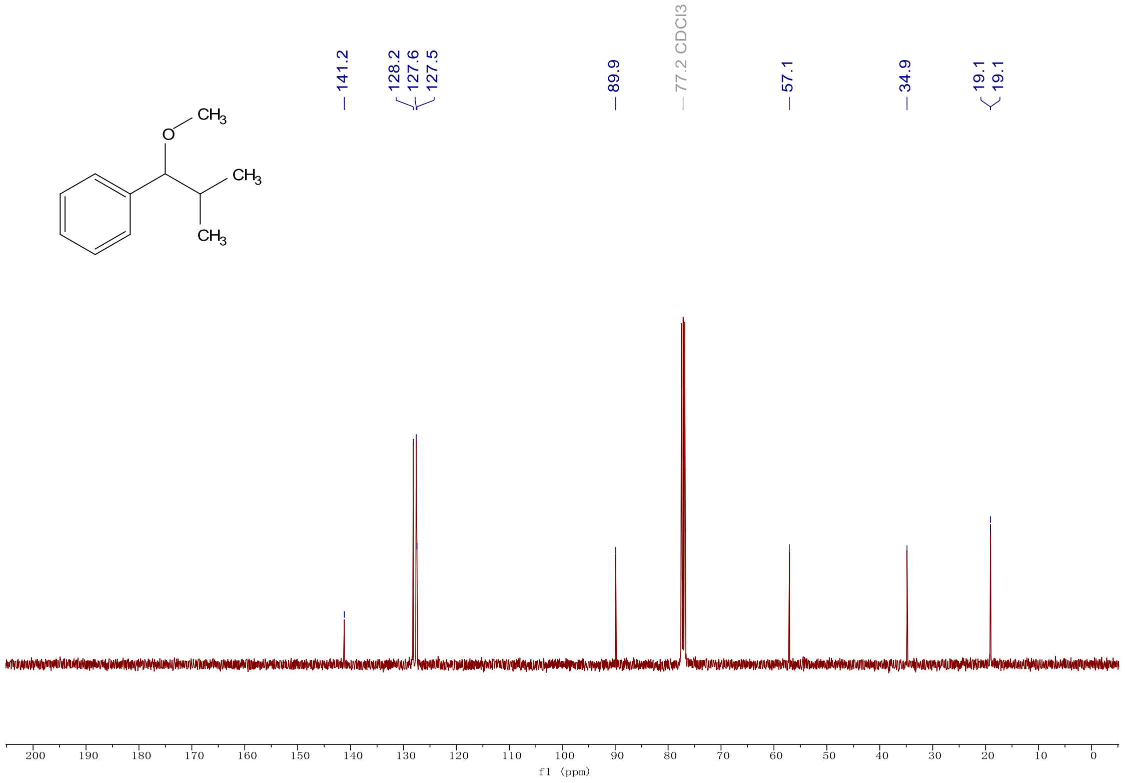

^{13}C NMR (101 MHz, $CDCl_3$) of **6l**

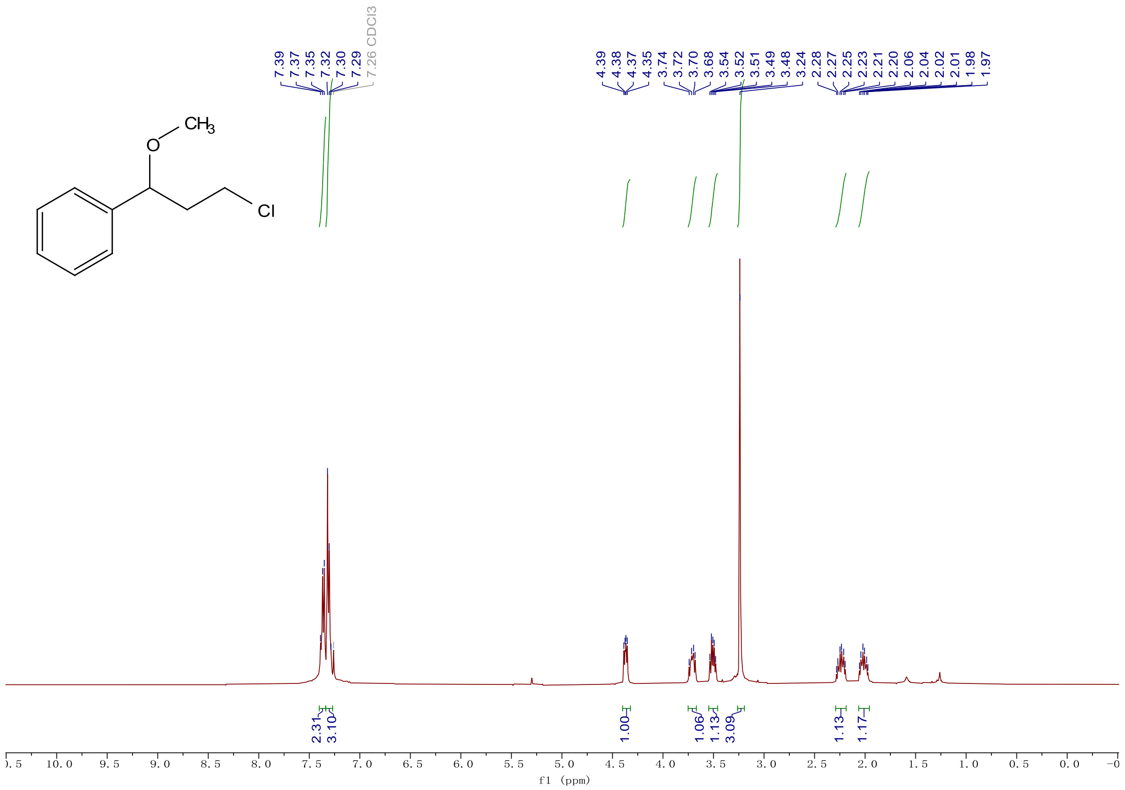

^{1}H NMR (400 MHz, $CDCl_3$) of **6m**

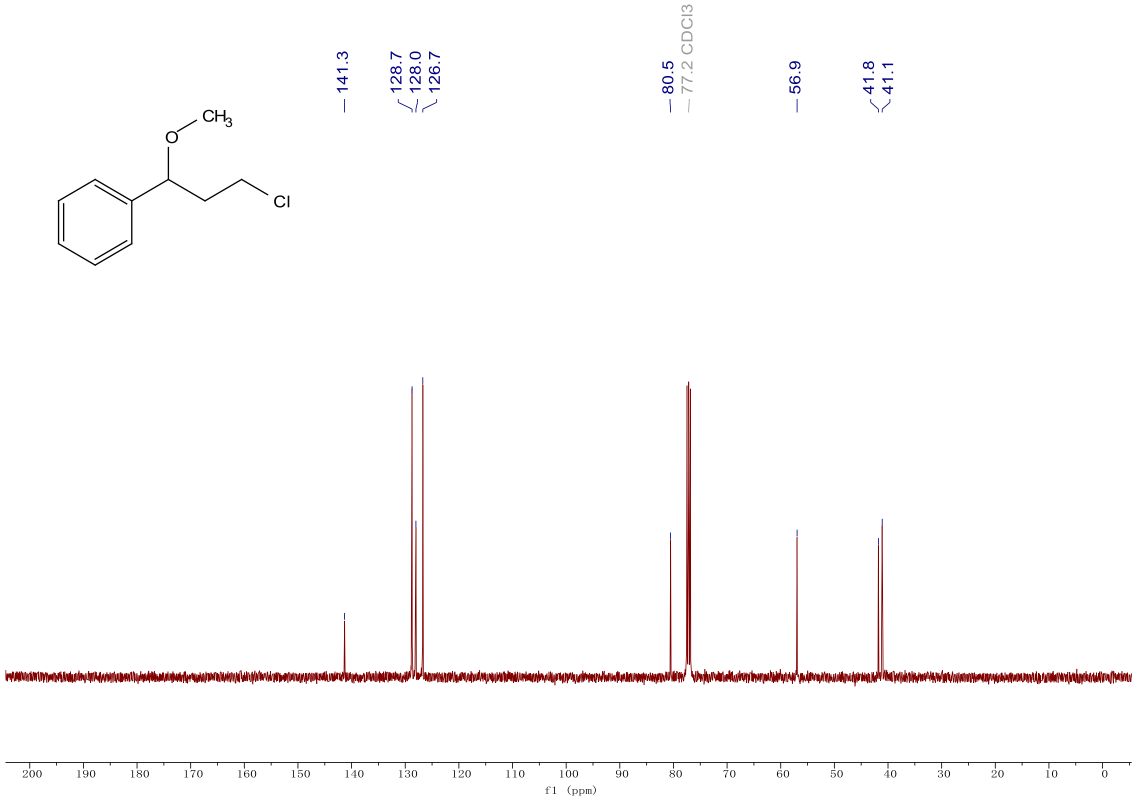

^{13}C NMR (101 MHz, $CDCl_3$) of **6m**

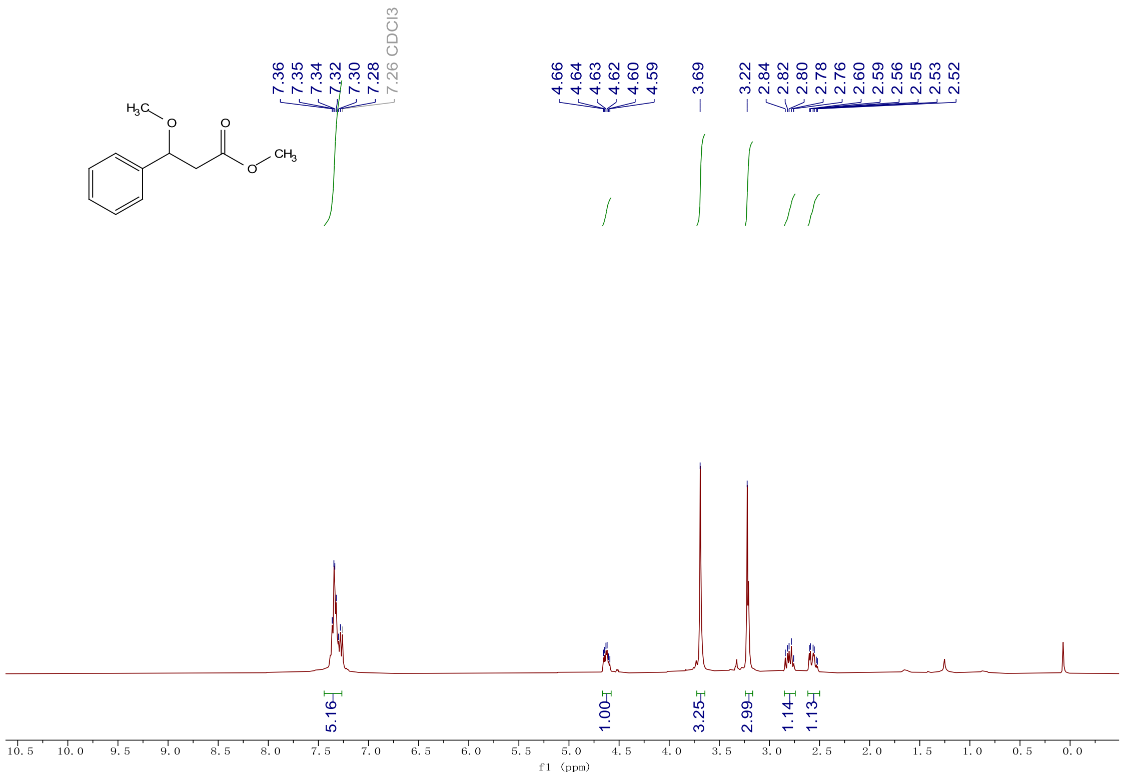

^{1}H NMR (400 MHz, $CDCl_3$) of **6n**

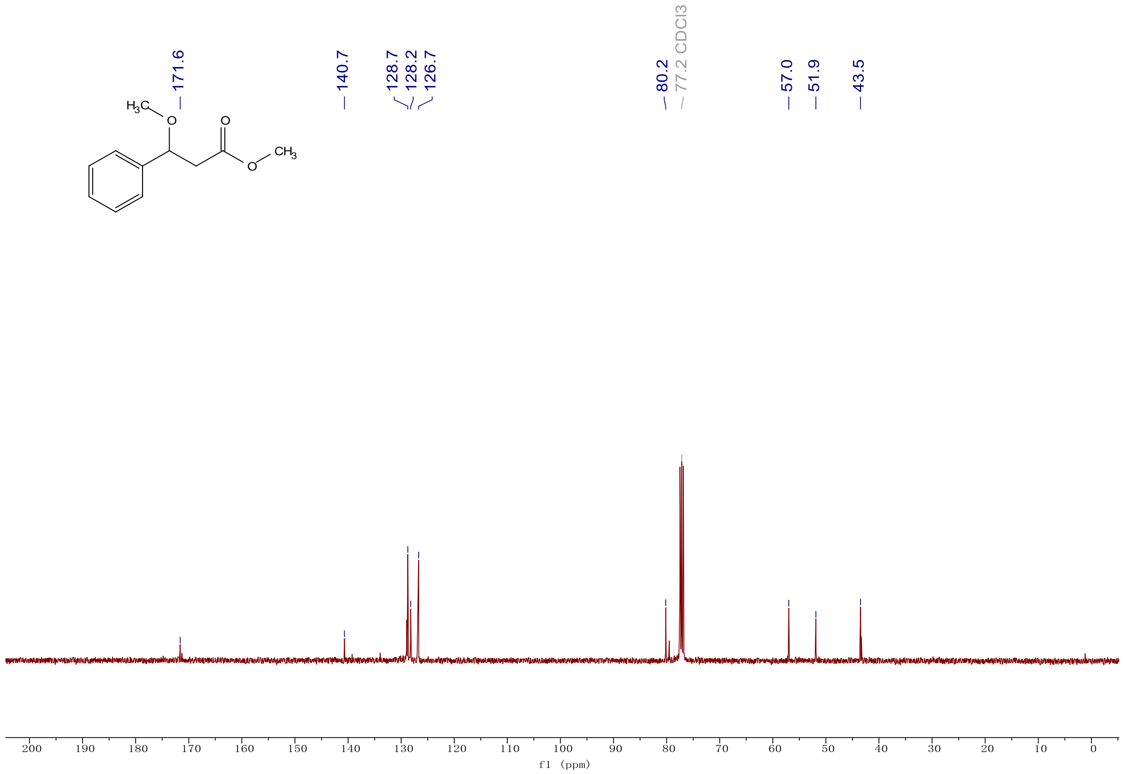

^{13}C NMR (101 MHz, $CDCl_3$) of **6n**

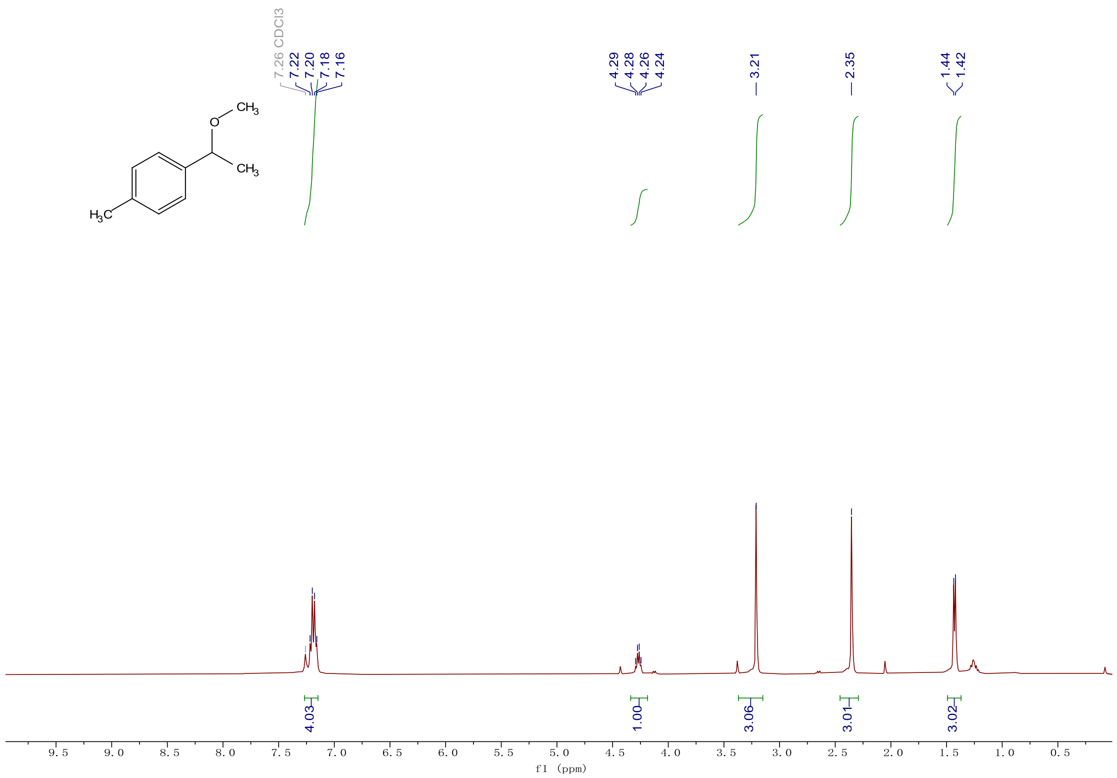

^{1}H NMR (101 MHz, $CDCl_3$) of **6o**

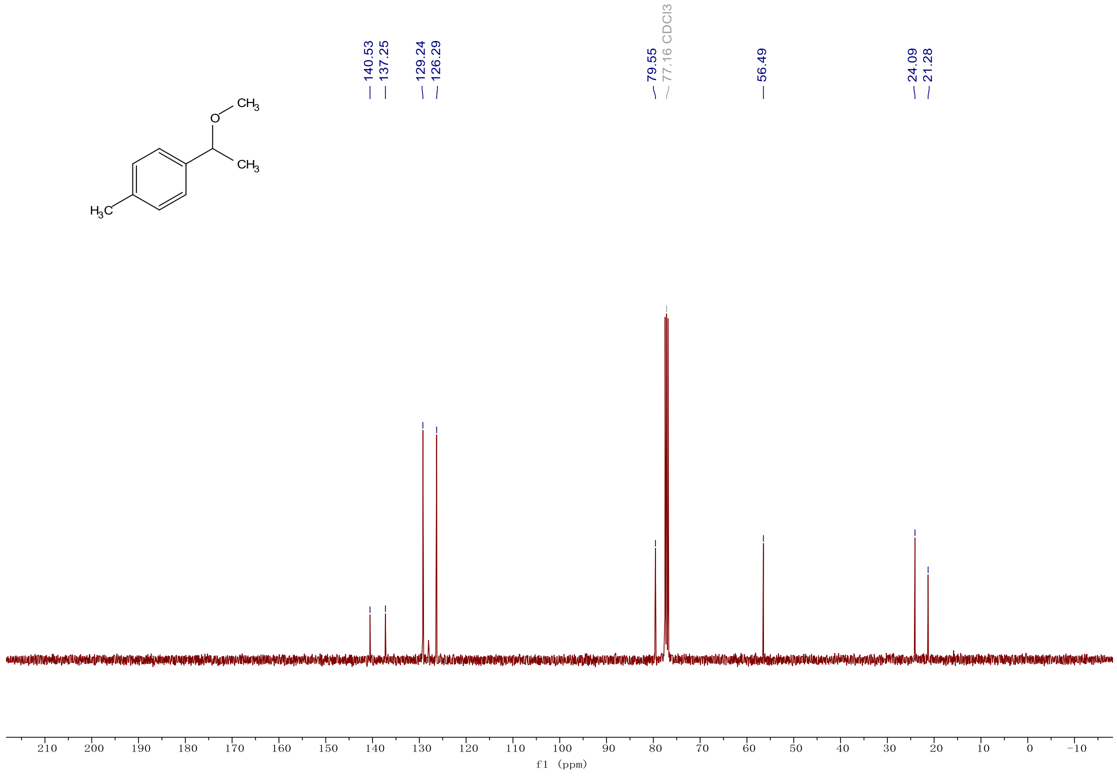

^{13}C NMR (101 MHz, $CDCl_3$) of **6o**

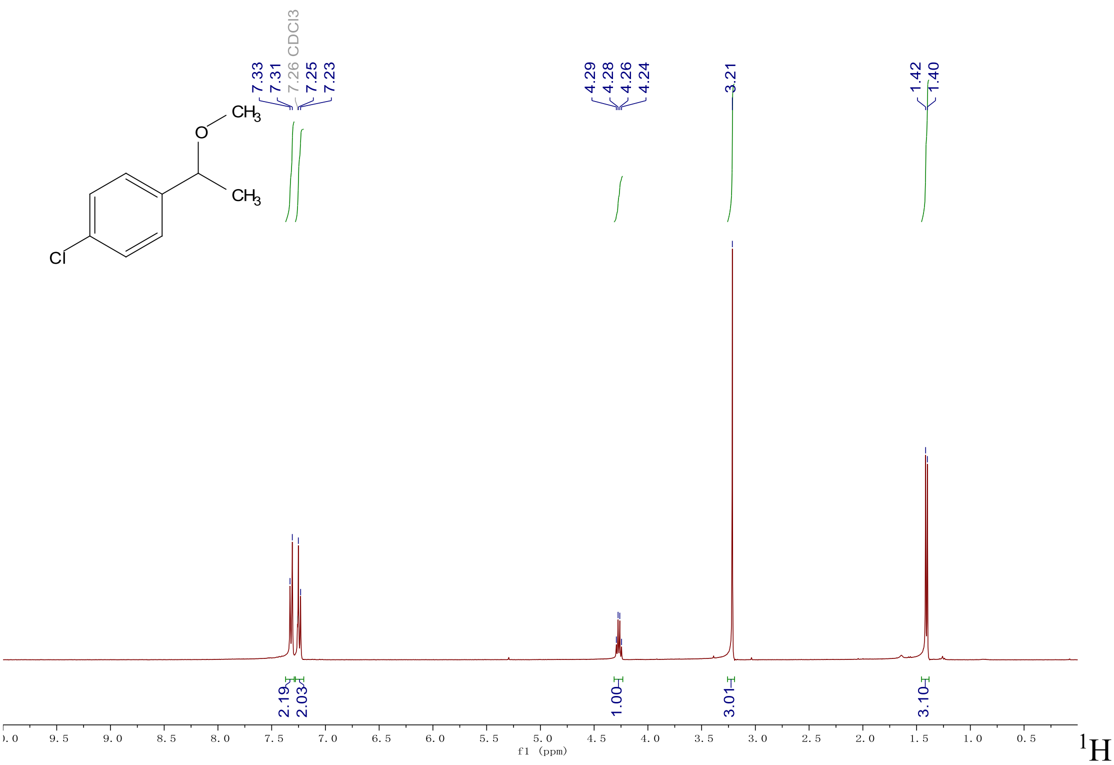

^{1}H NMR (400 MHz, $CDCl_3$) of **6p**

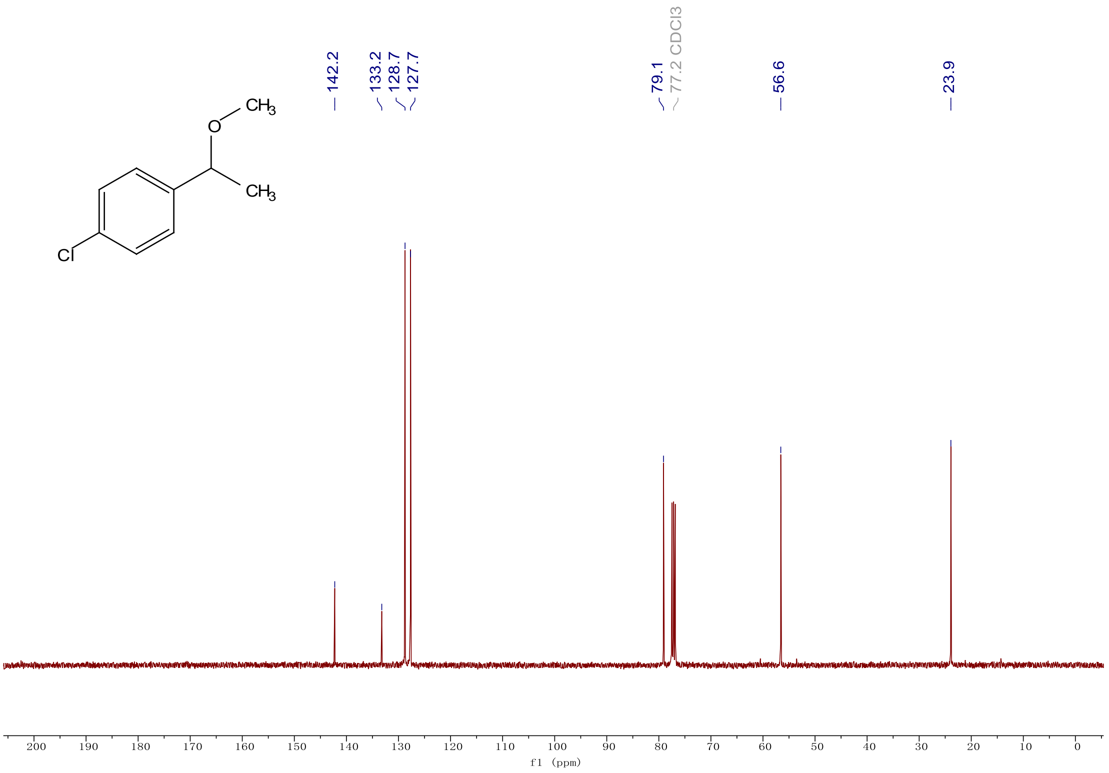

^{13}C NMR (101 MHz, $CDCl_3$) of **6p**

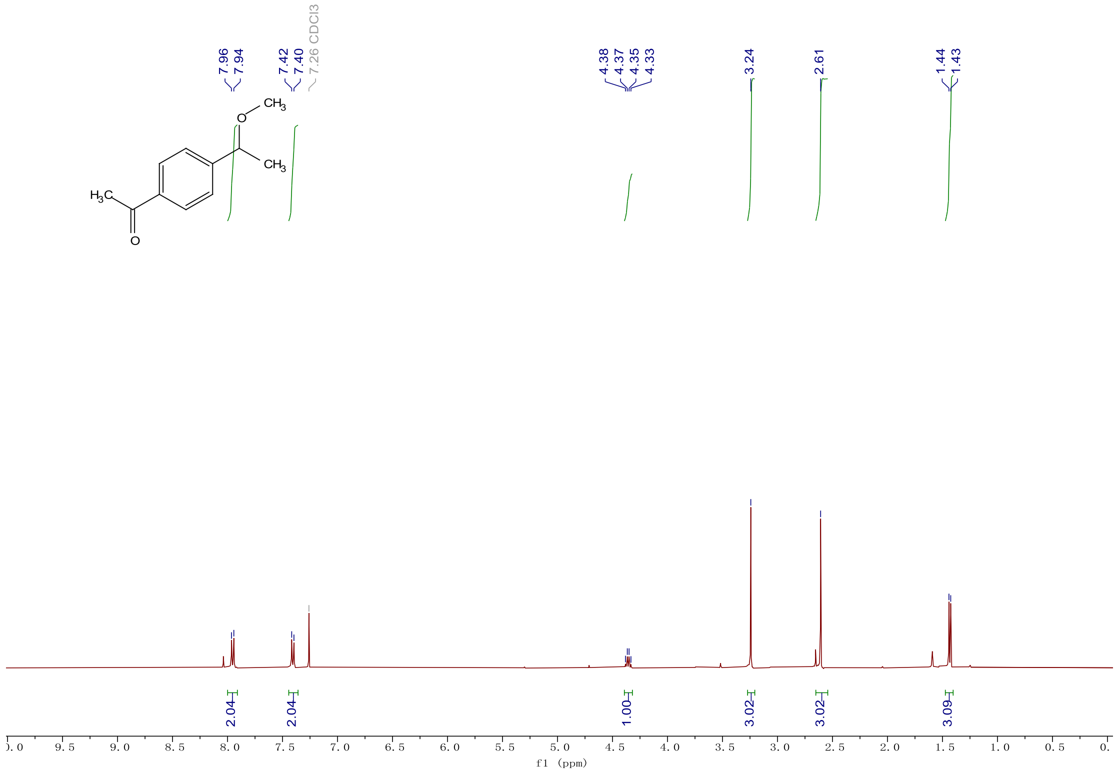

^{1}H NMR (400 MHz, $CDCl_3$) of **6q**

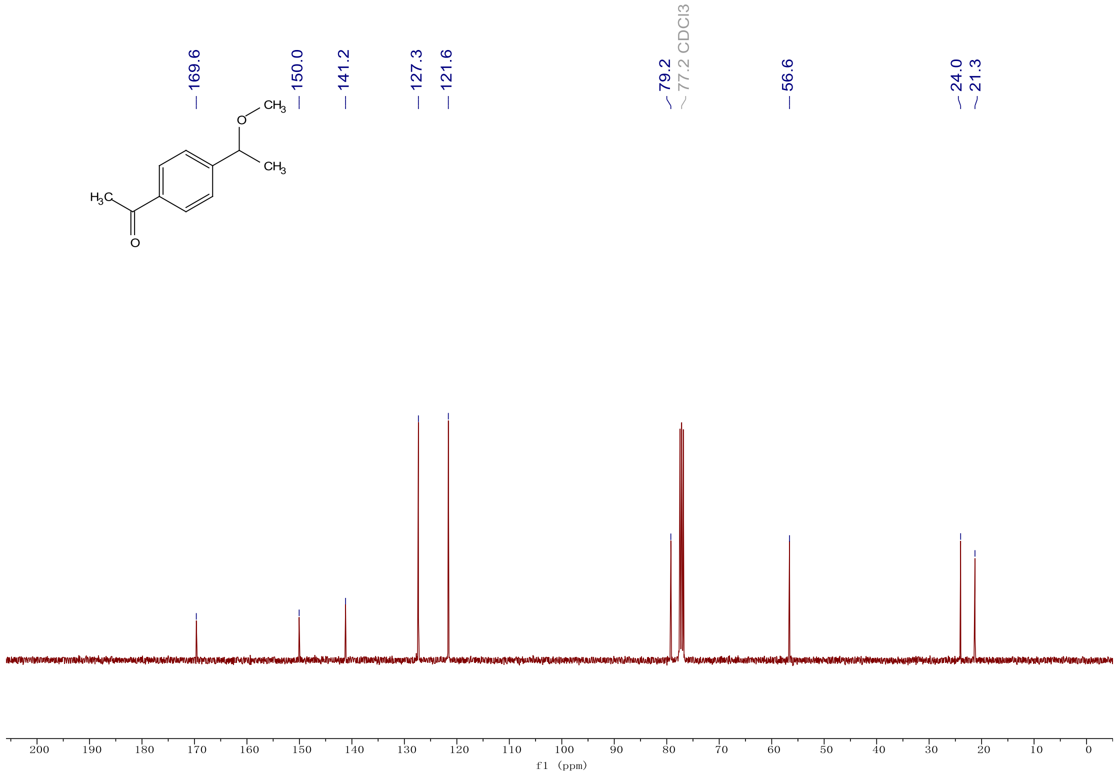

^{13}C NMR (101 MHz, $CDCl_3$) of **6q**

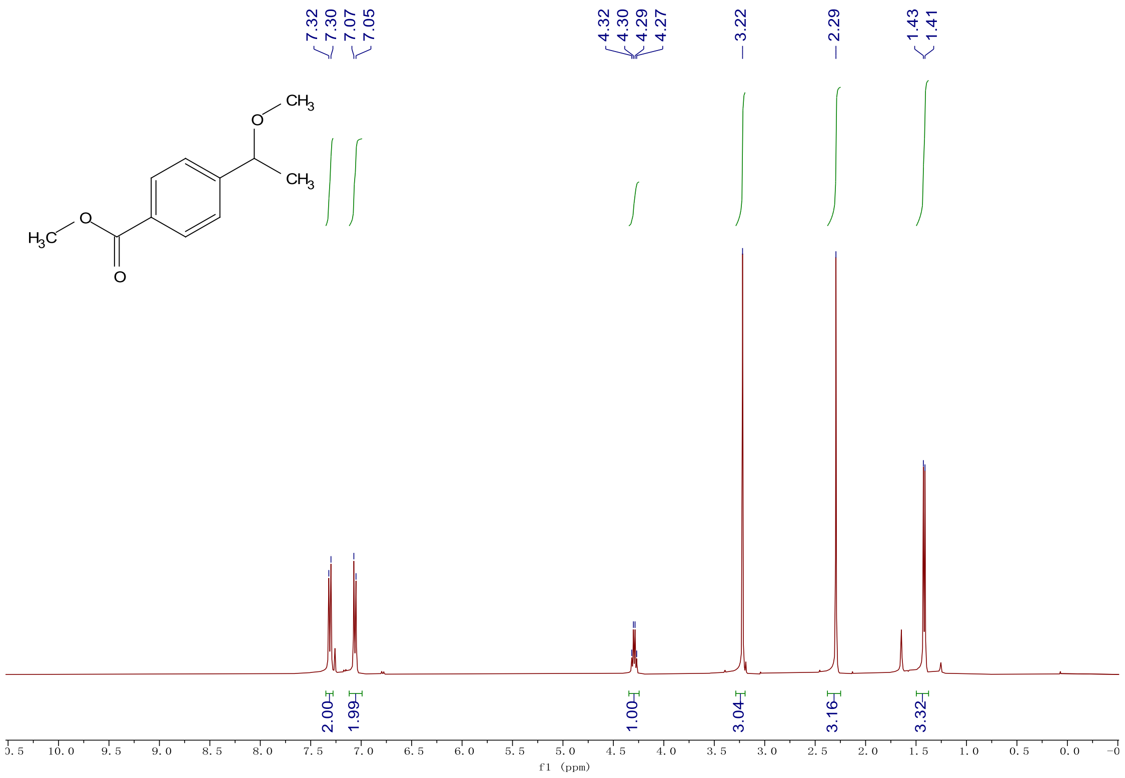

^{1}H NMR (400 MHz, CDCl$_3$) of **6r**

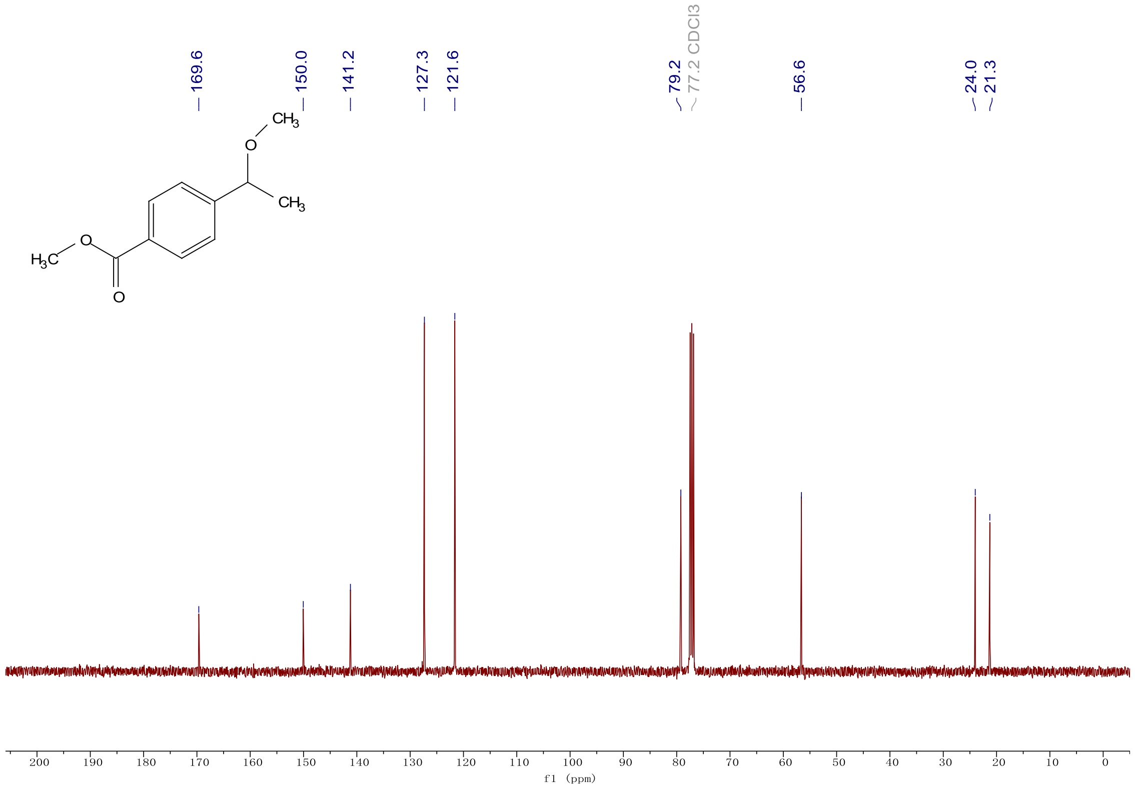

^{13}C NMR (101 MHz, CDCl$_3$) of **6r**

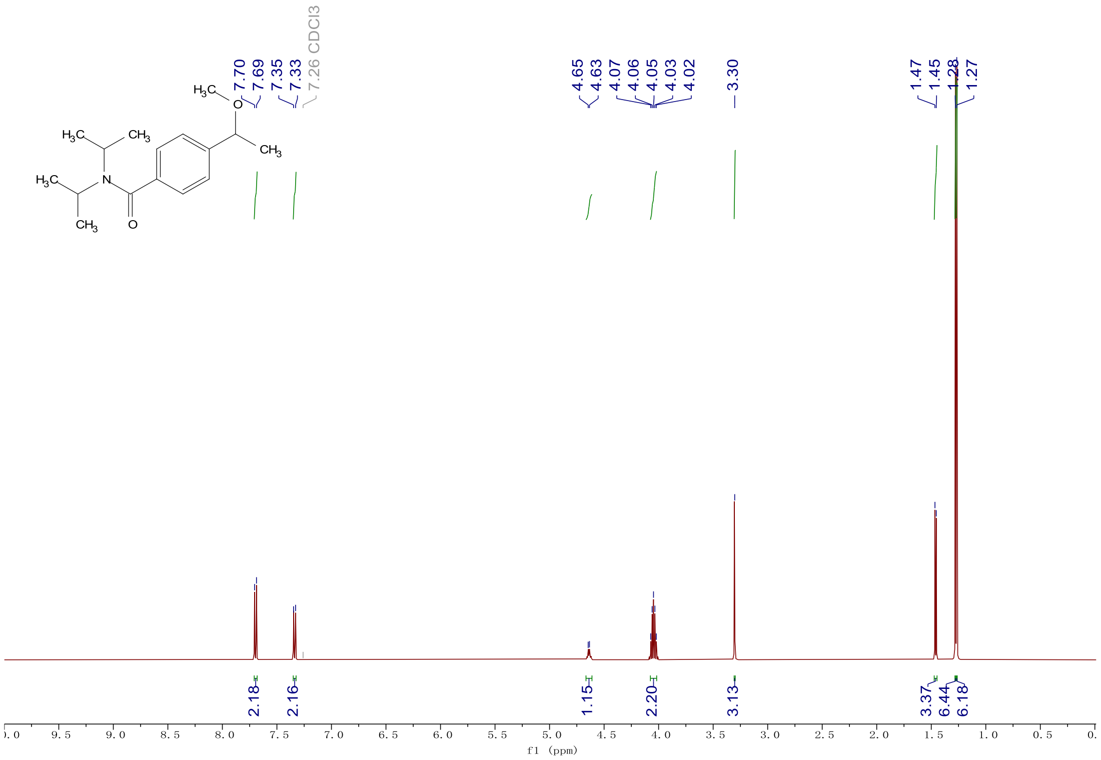

^{1}H NMR (400 MHz, $CDCl_3$) of **6s**

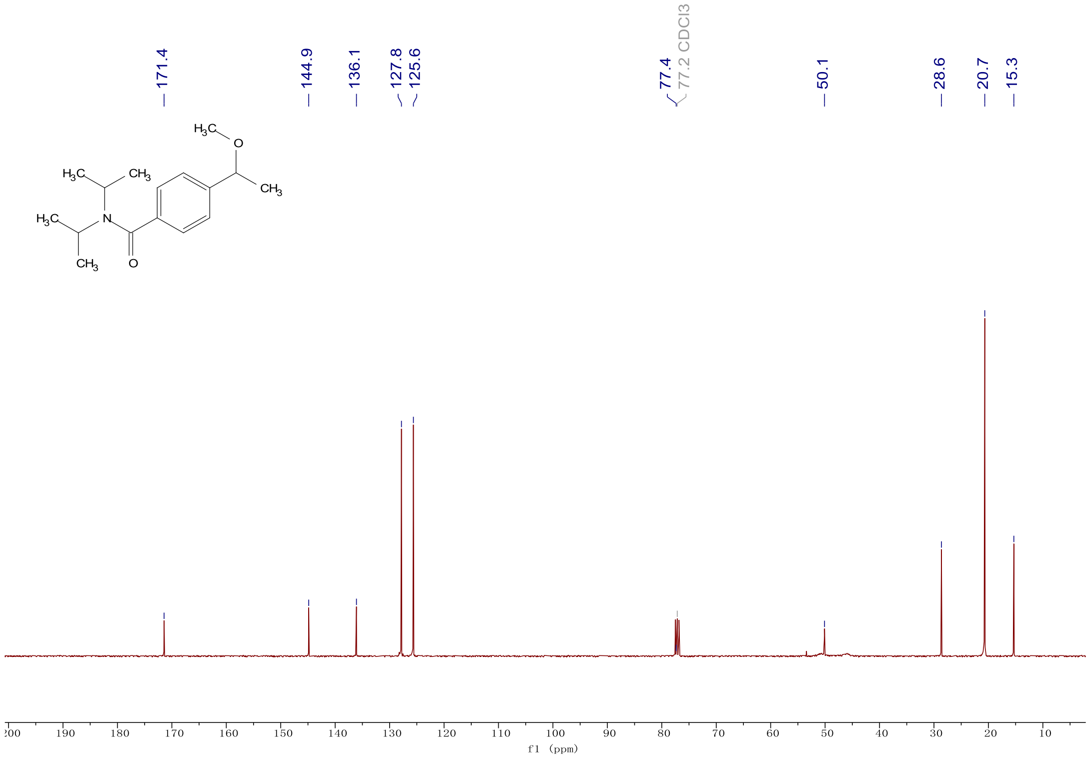

^{13}C NMR (101 MHz, $CDCl_3$) of **6s**

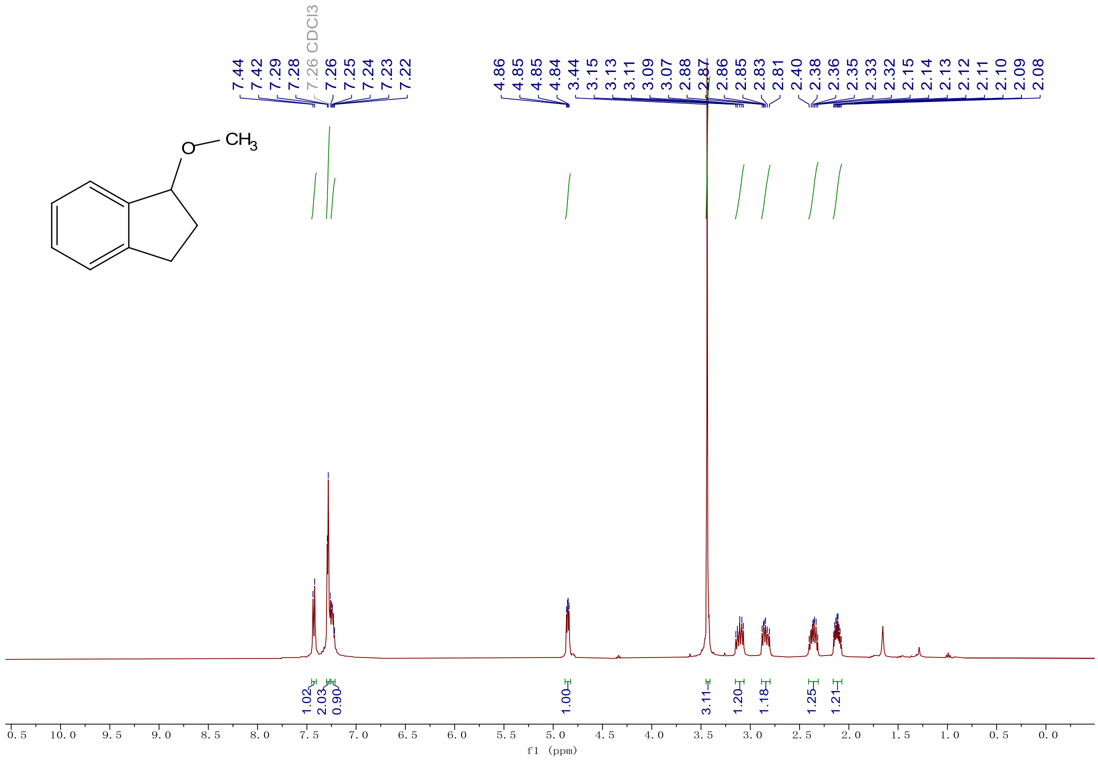

^1H NMR (400 MHz, $CDCl_3$) of **6t**

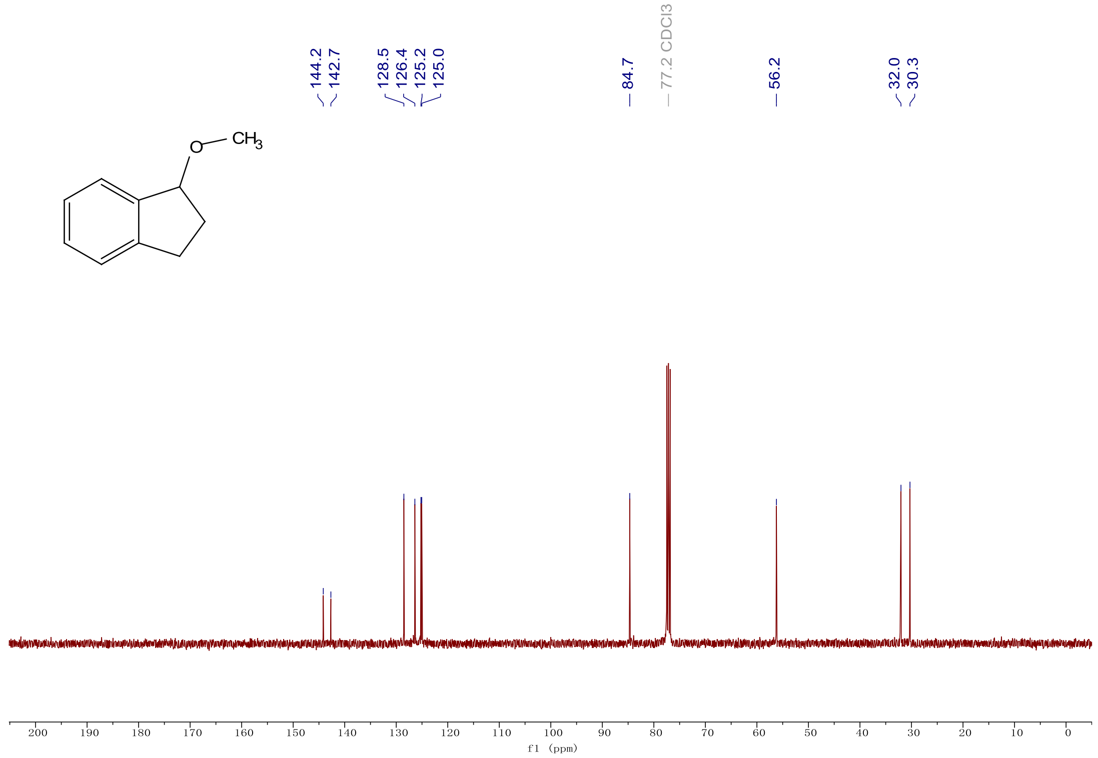

^{13}C NMR (101 MHz, $CDCl_3$) of **6t**

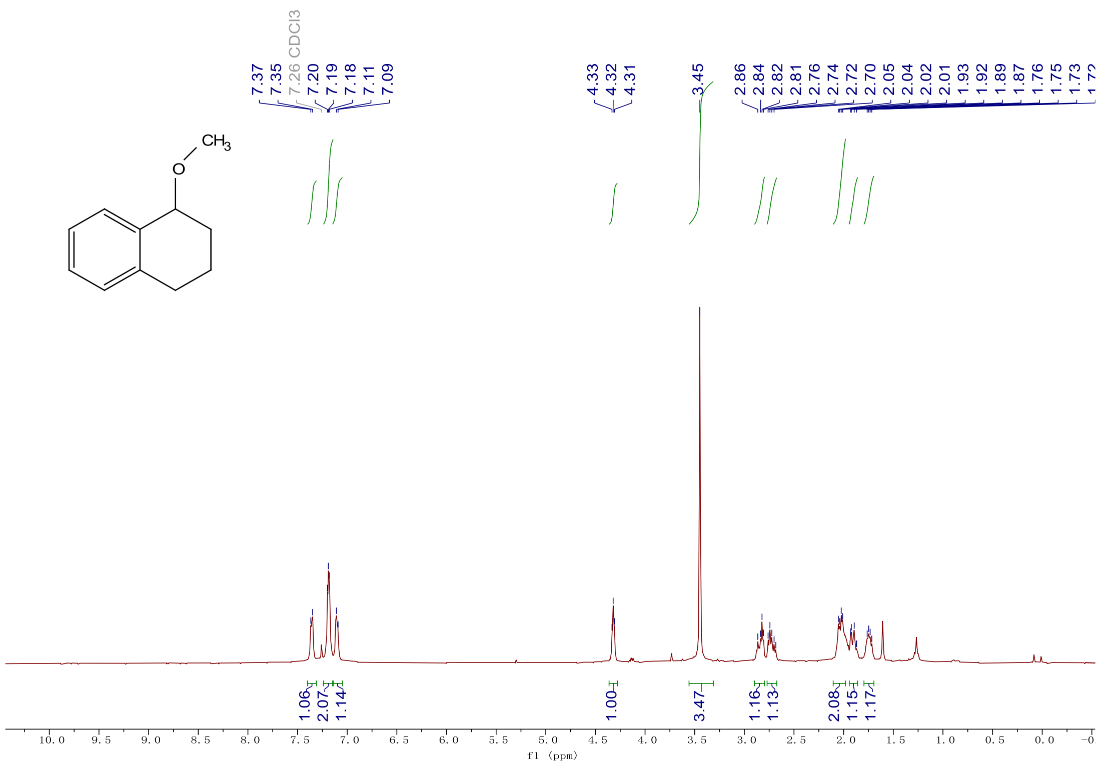

^{1}H NMR (400 MHz, $CDCl_3$) of **6u**

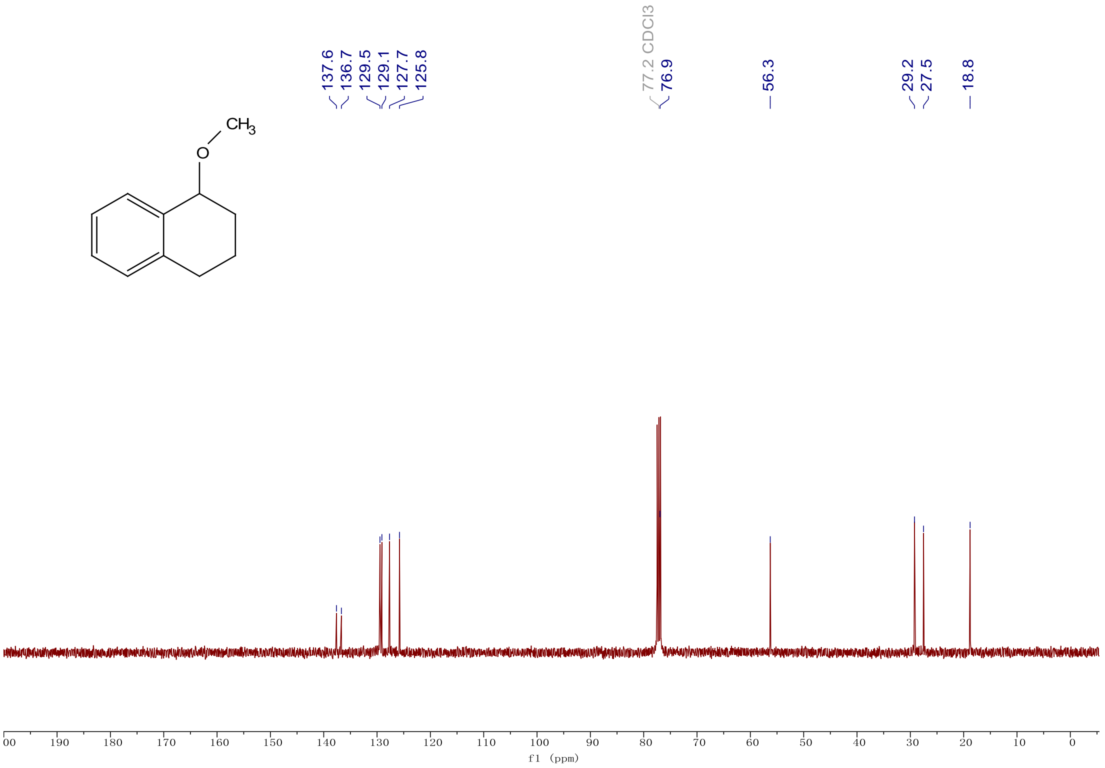

^{13}C NMR (101 MHz, $CDCl_3$) of **6u**

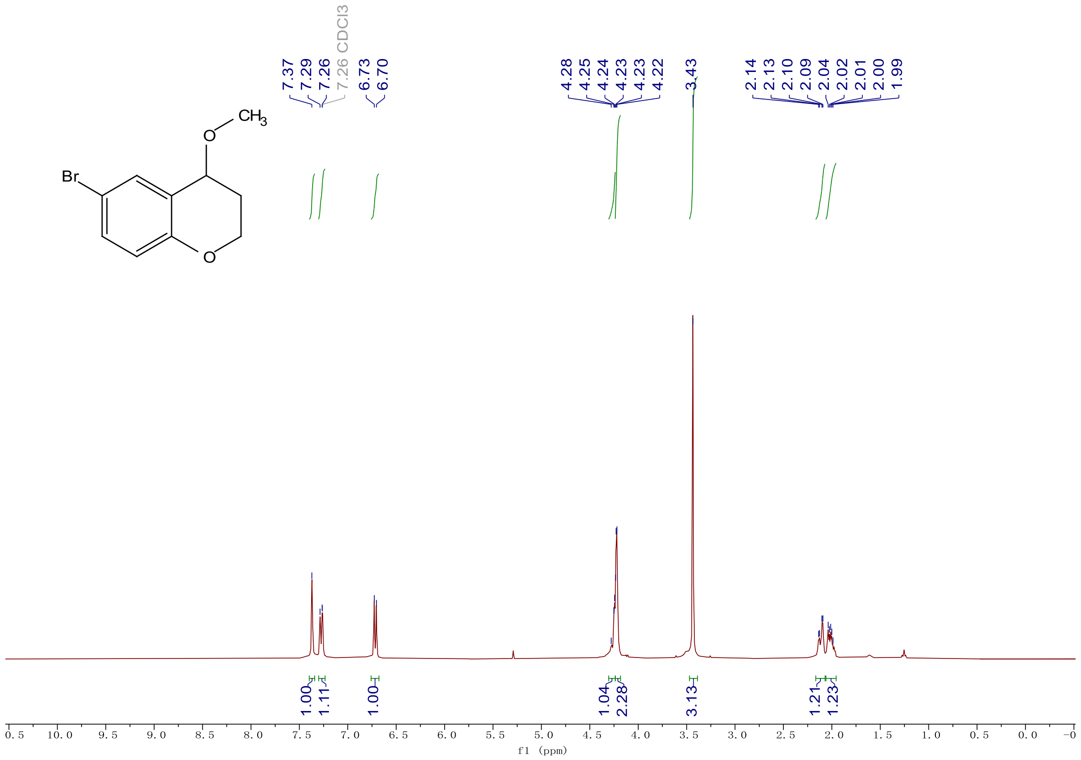

^{1}H NMR (400 MHz, $CDCl_3$) of **6v**

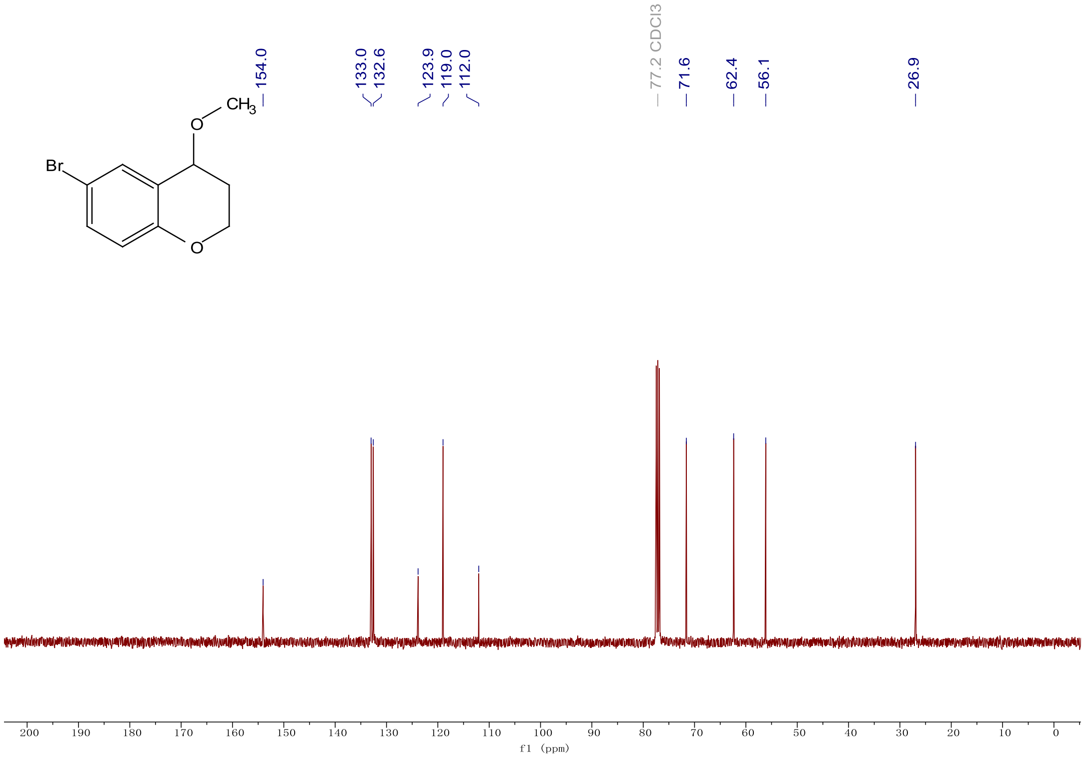

^{13}C NMR (101 MHz, $CDCl_3$) of **6v**

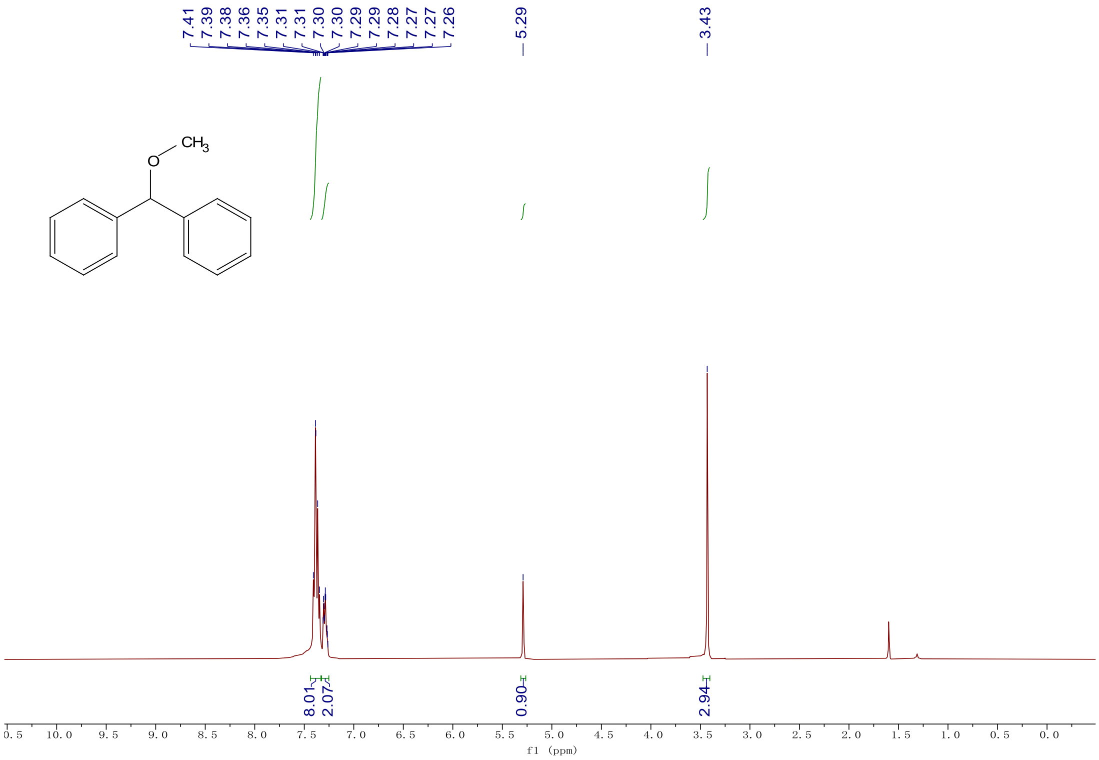

^{1}H NMR (400 MHz, $CDCl_3$) of **6w**

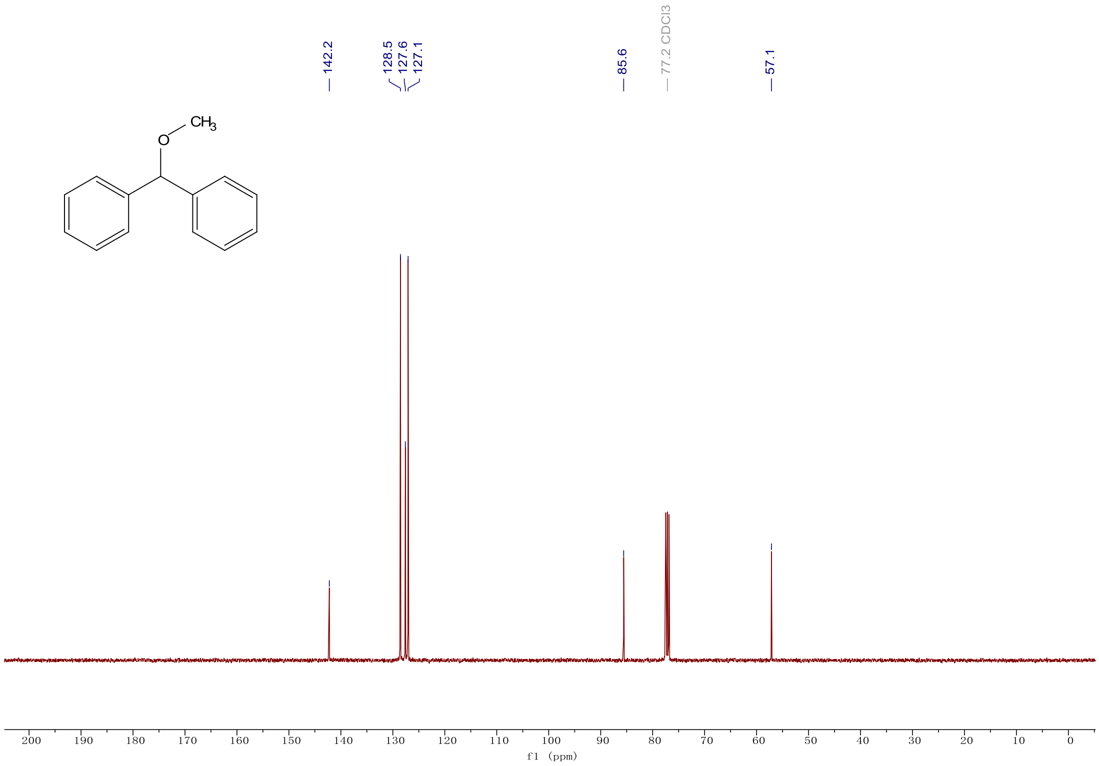

^{13}C NMR (101 MHz, $CDCl_3$) of **6w**

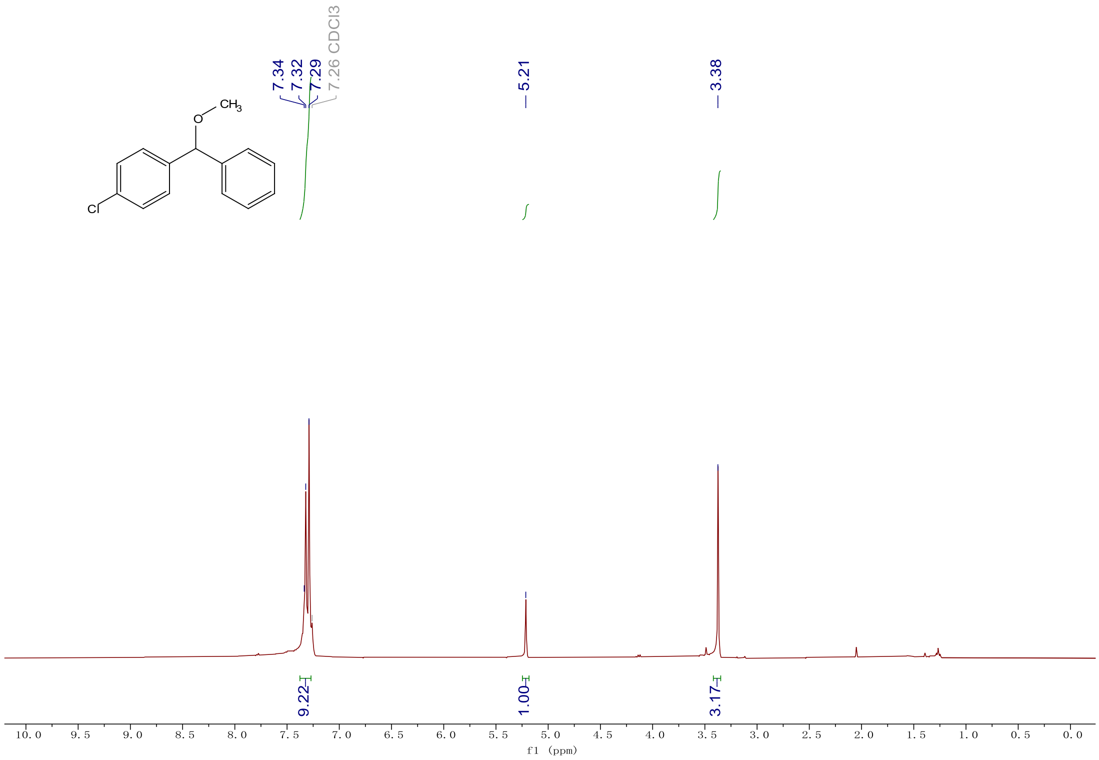

^{1}H NMR (400 MHz, $CDCl_3$) of **6x**

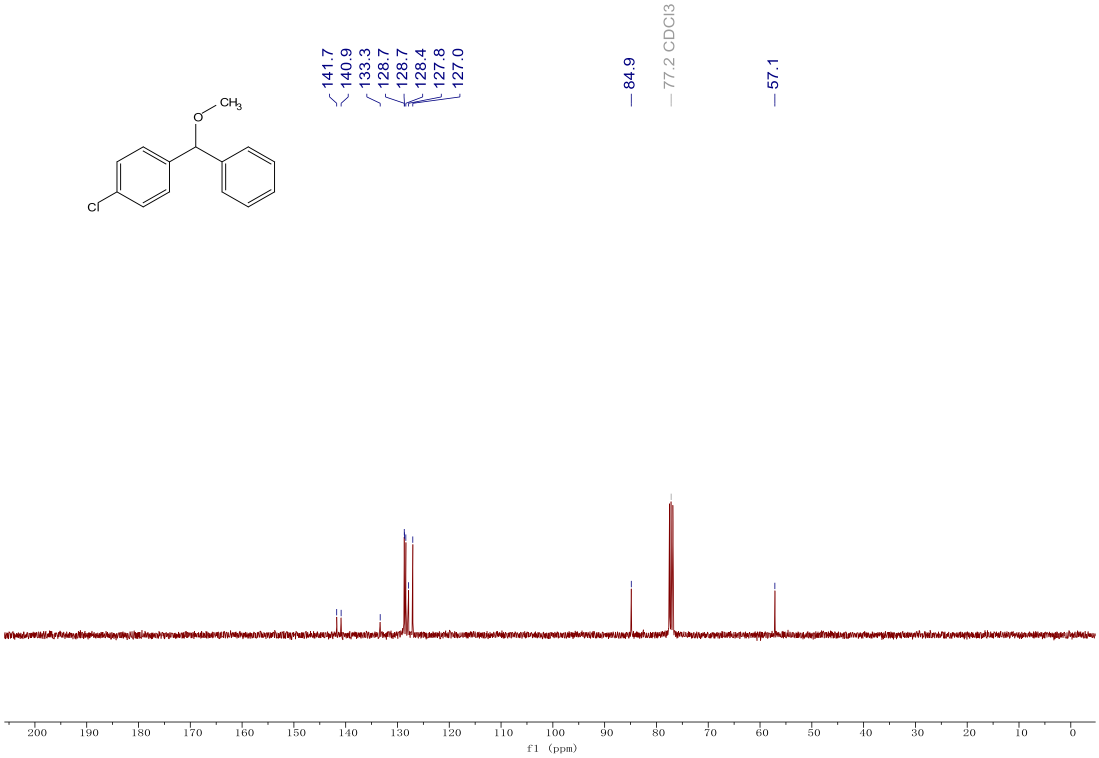

^{13}C NMR (101 MHz, $CDCl_3$) of **6x**

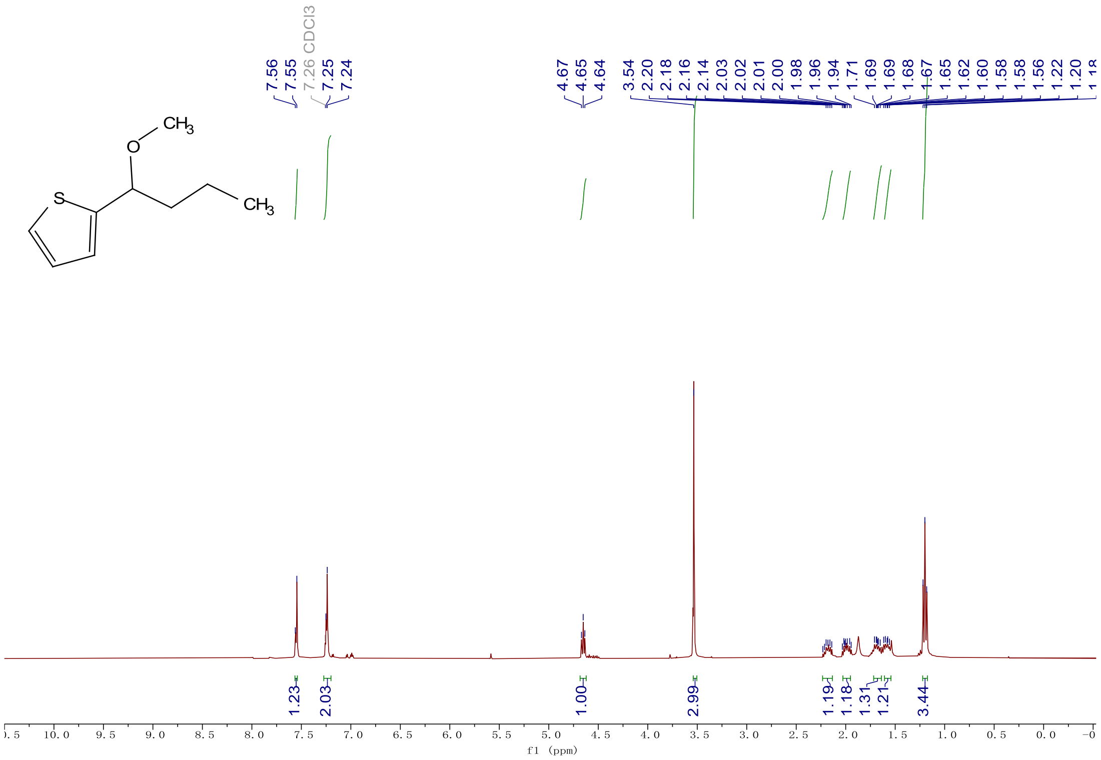

^{1}H NMR (400 MHz, $CDCl_3$) of **6y**

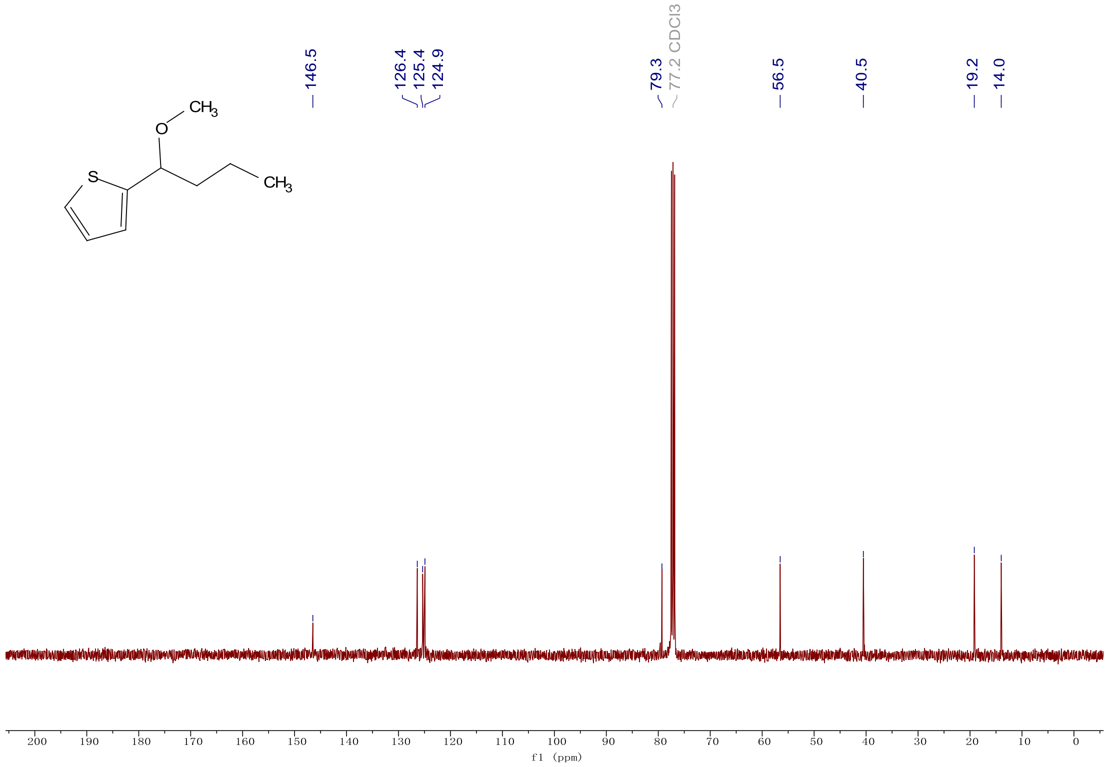

^{13}C NMR (101 MHz, $CDCl_3$) of **6y**

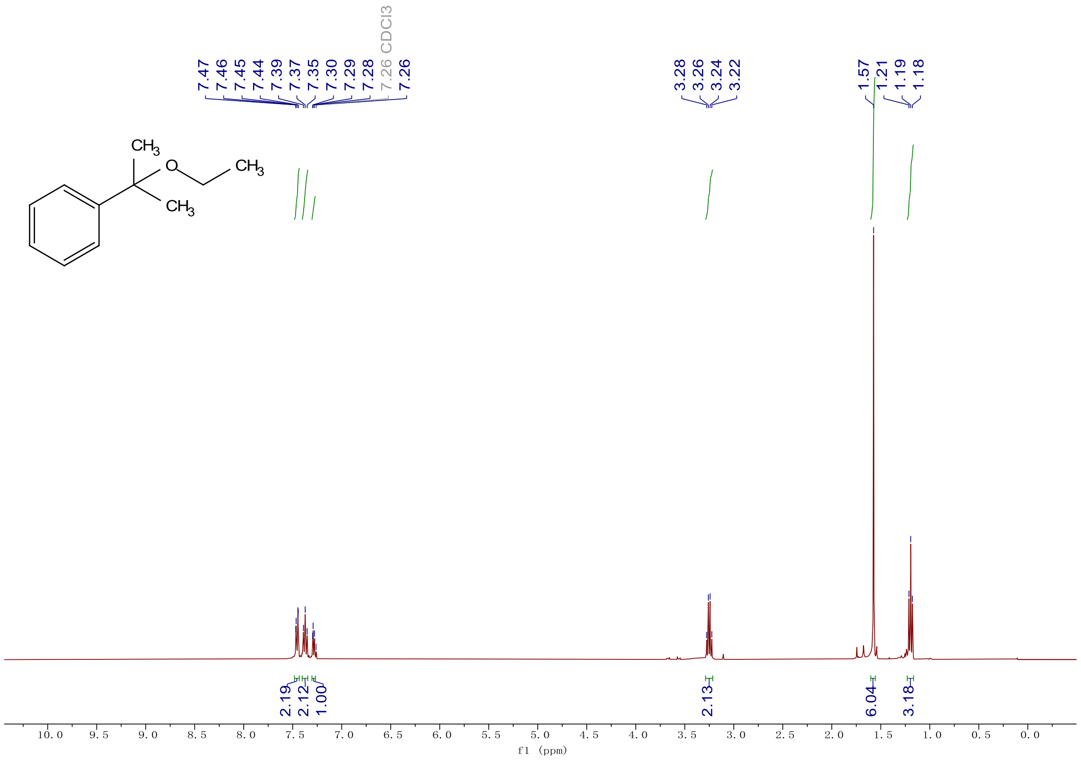

^{1}H NMR (400 MHz, $CDCl_3$) of **10a**

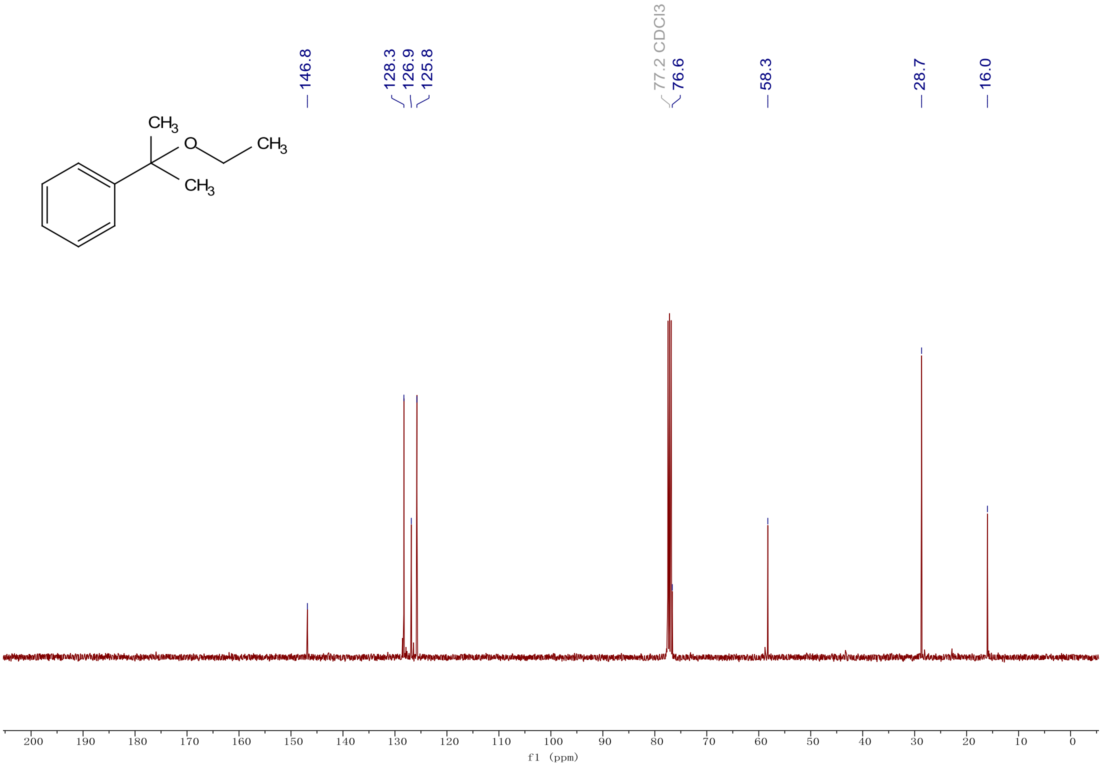

^{13}C NMR (101 MHz, $CDCl_3$) of **10a**

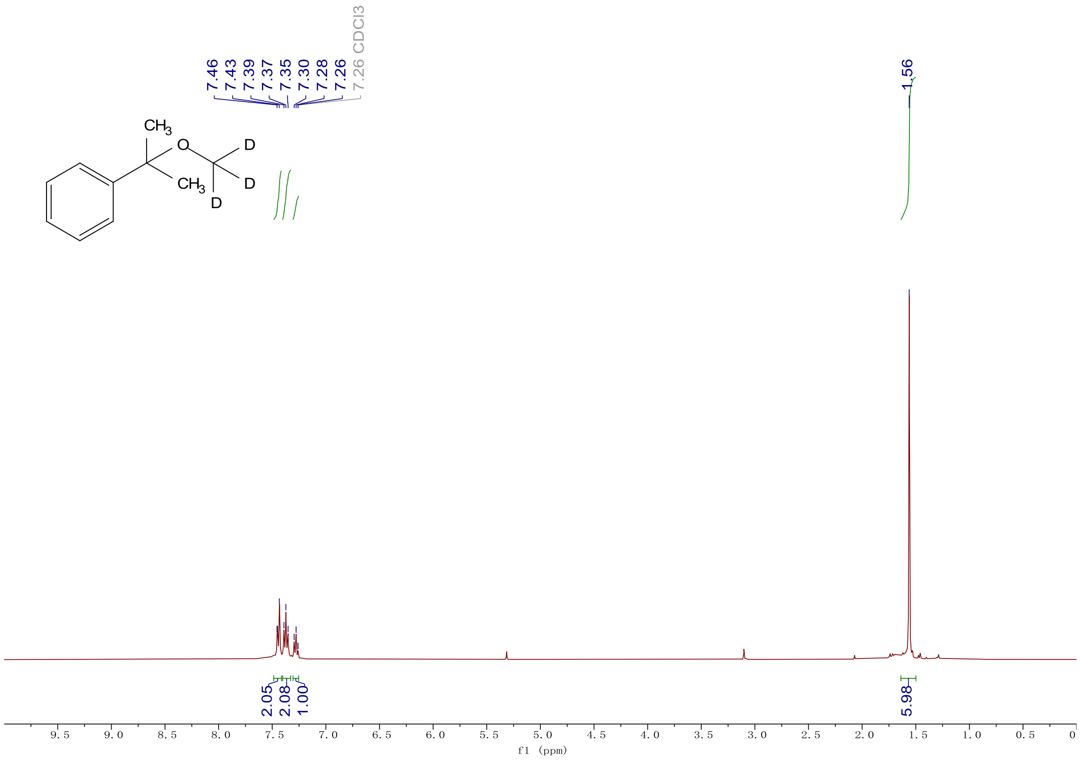

^{1}H NMR (400 MHz, $CDCl_3$) of **10b**

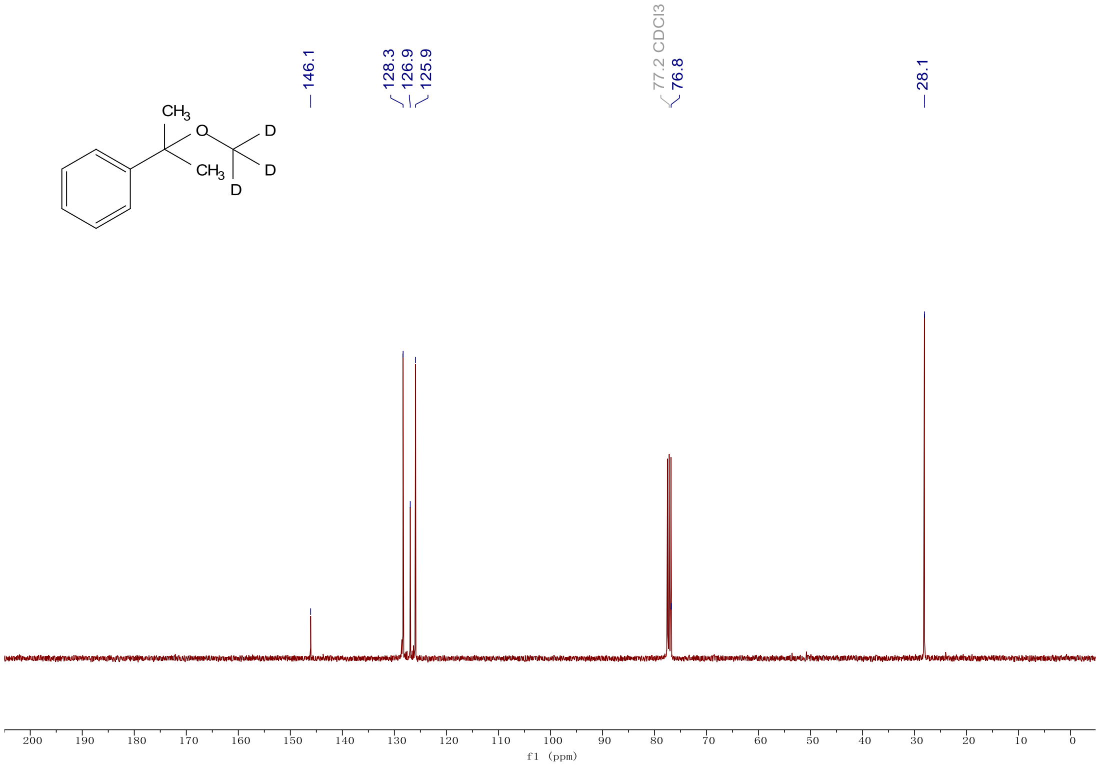

^{13}C NMR (101 MHz, $CDCl_3$) of **10b**

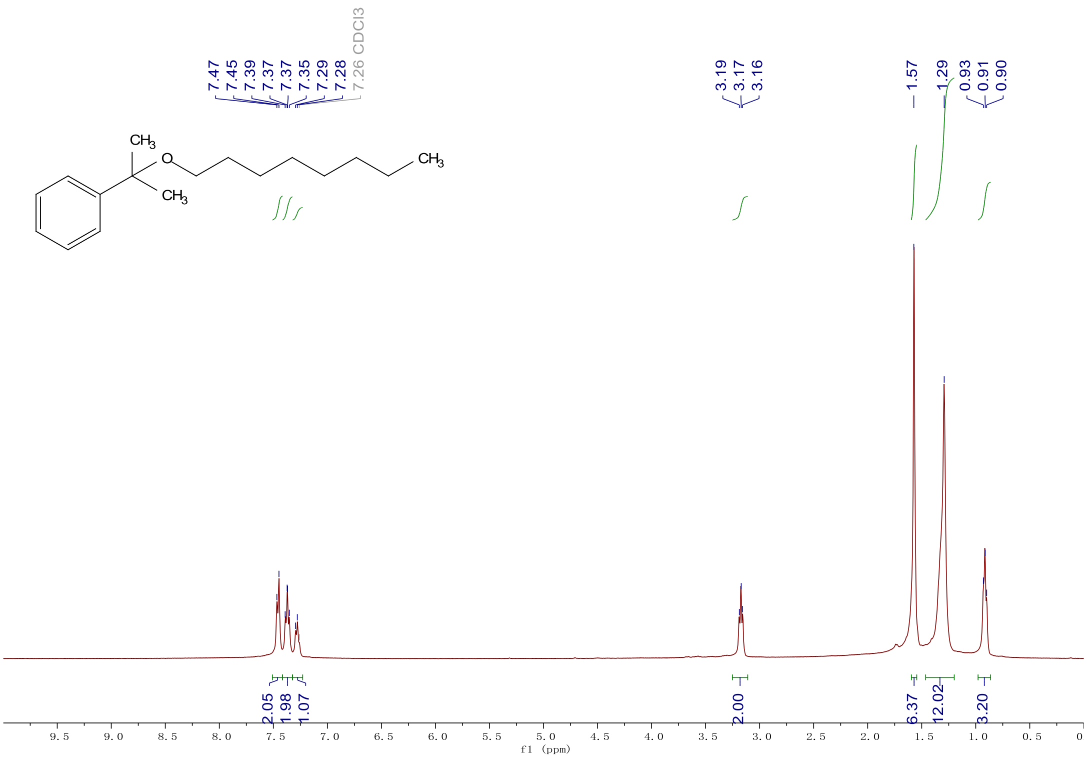

^{1}H NMR (400 MHz, $CDCl_3$) of **10c**

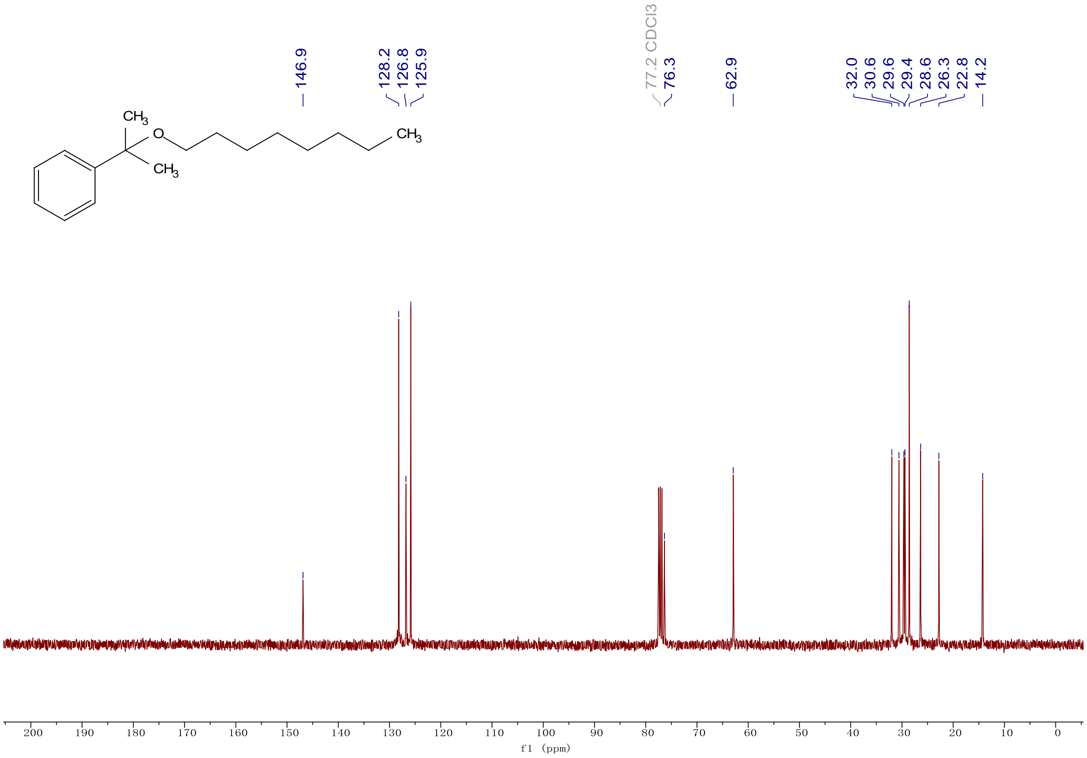

^{13}C NMR (101 MHz, $CDCl_3$) of **10c**

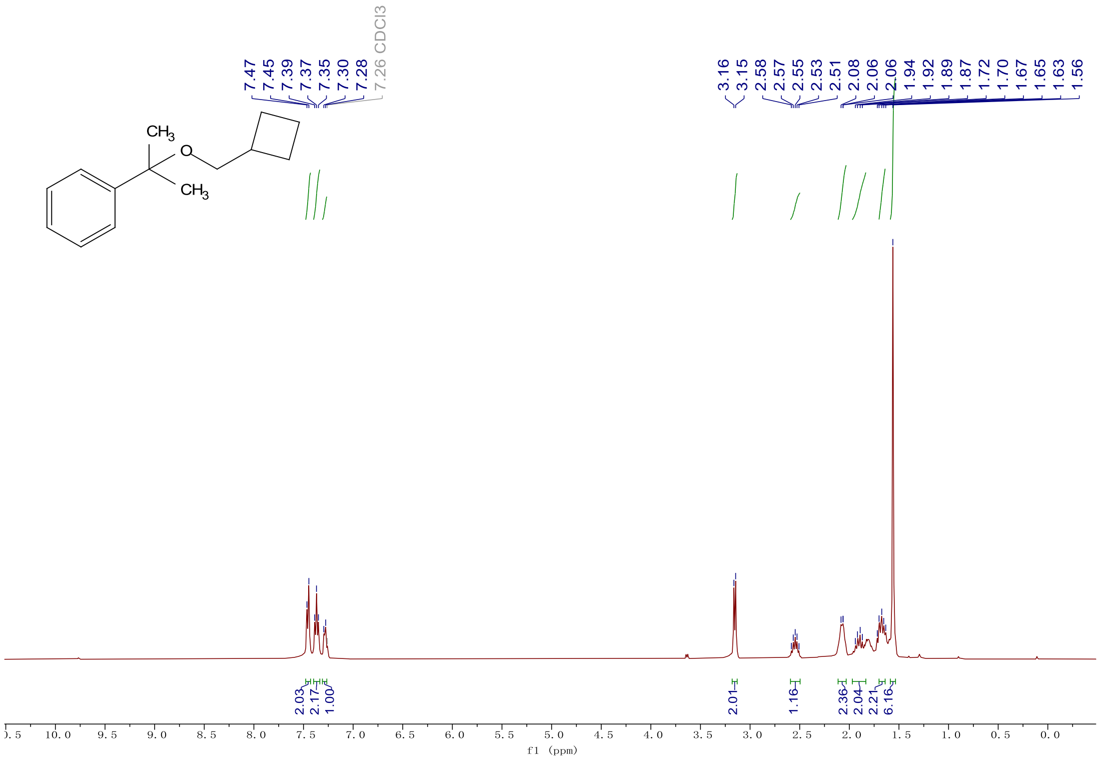

^{1}H NMR (400 MHz, $CDCl_3$) of **10d**

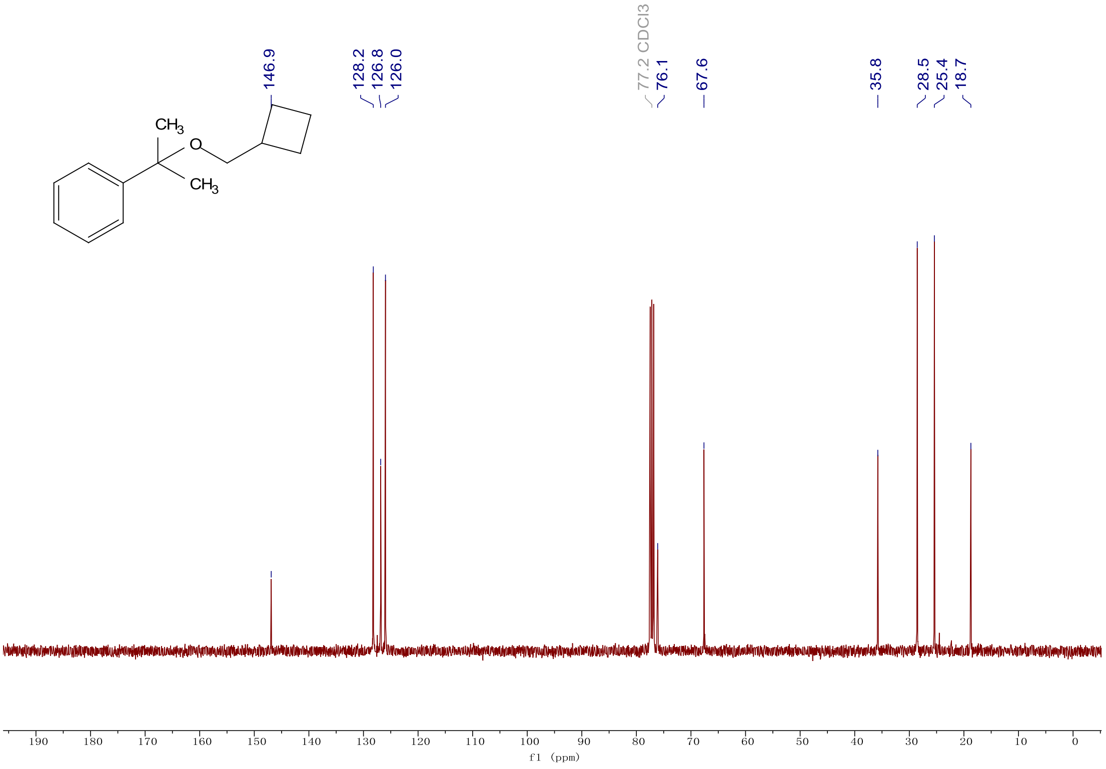

^{13}C NMR (101 MHz, $CDCl_3$) of **10d**

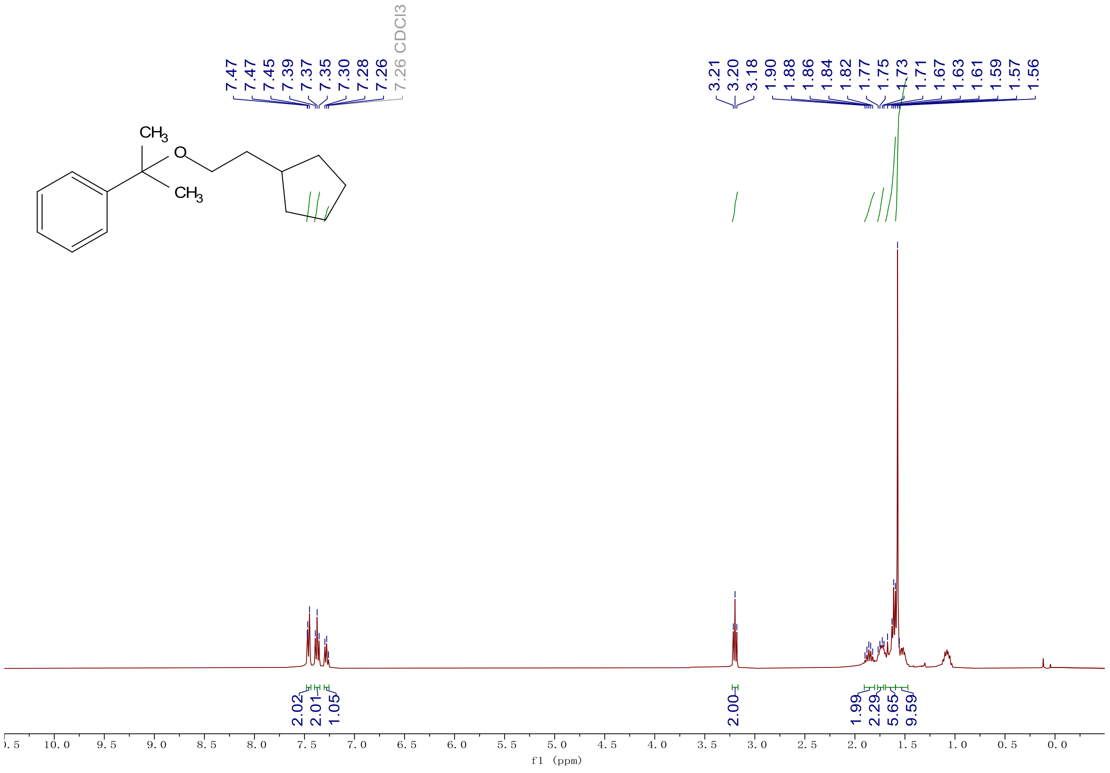

^{1}H NMR (400 MHz, $CDCl_3$) of **10e**

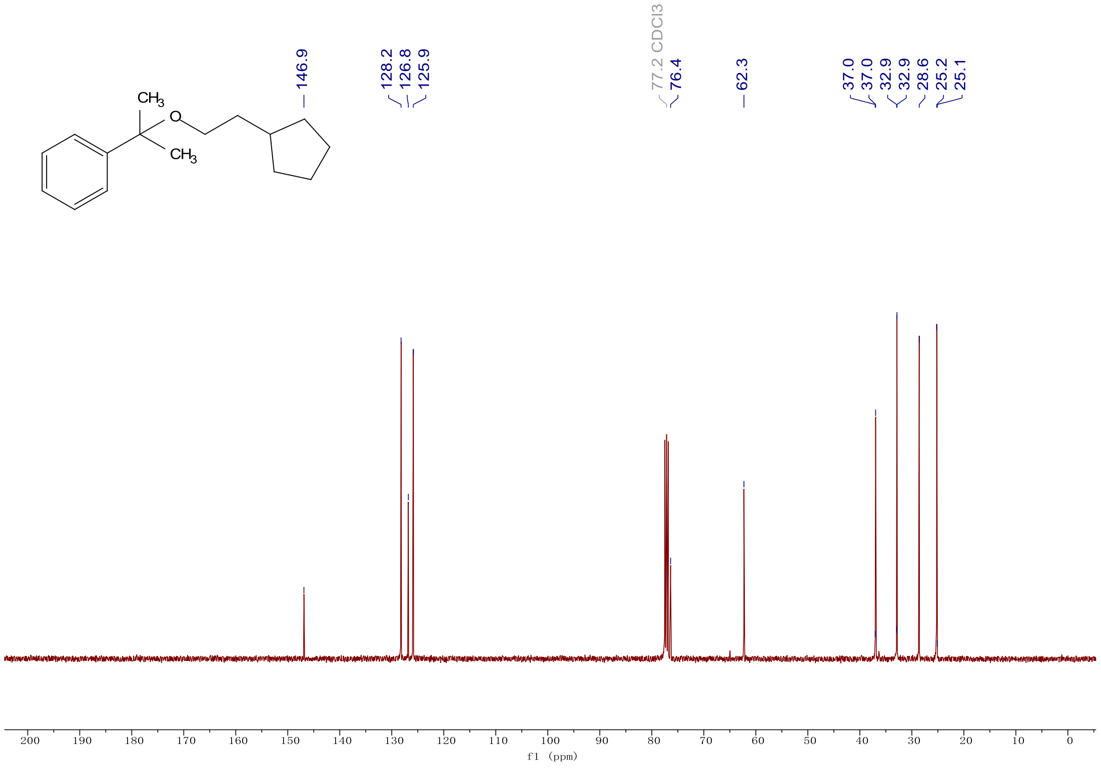

^{13}C NMR (101 MHz, $CDCl_3$) of **10e**

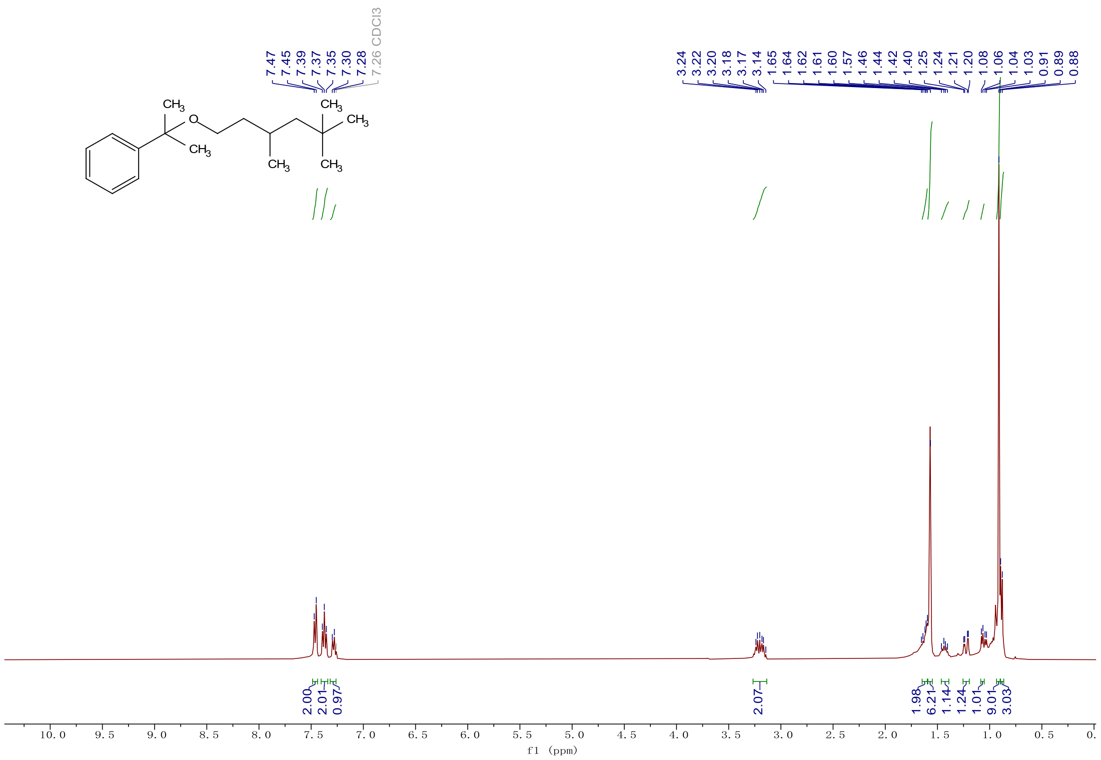

^{1}H NMR (400 MHz, $CDCl_3$) of **10f**

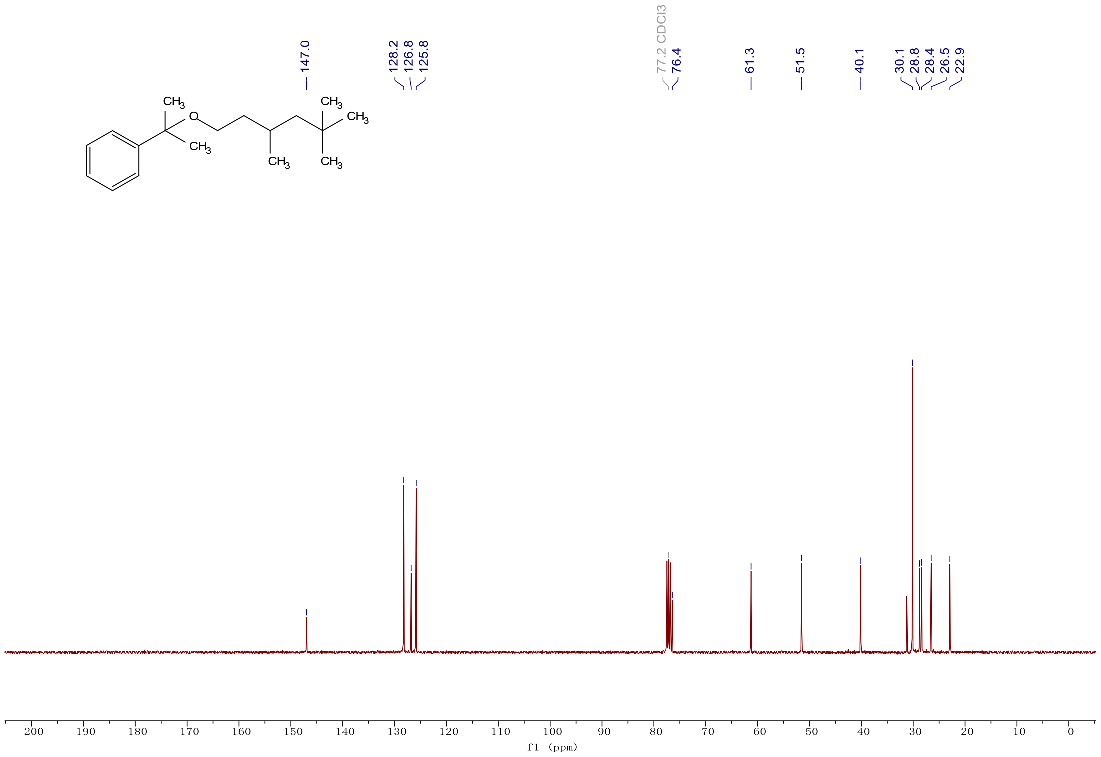

^{13}C NMR (101 MHz, $CDCl_3$) of **10f**

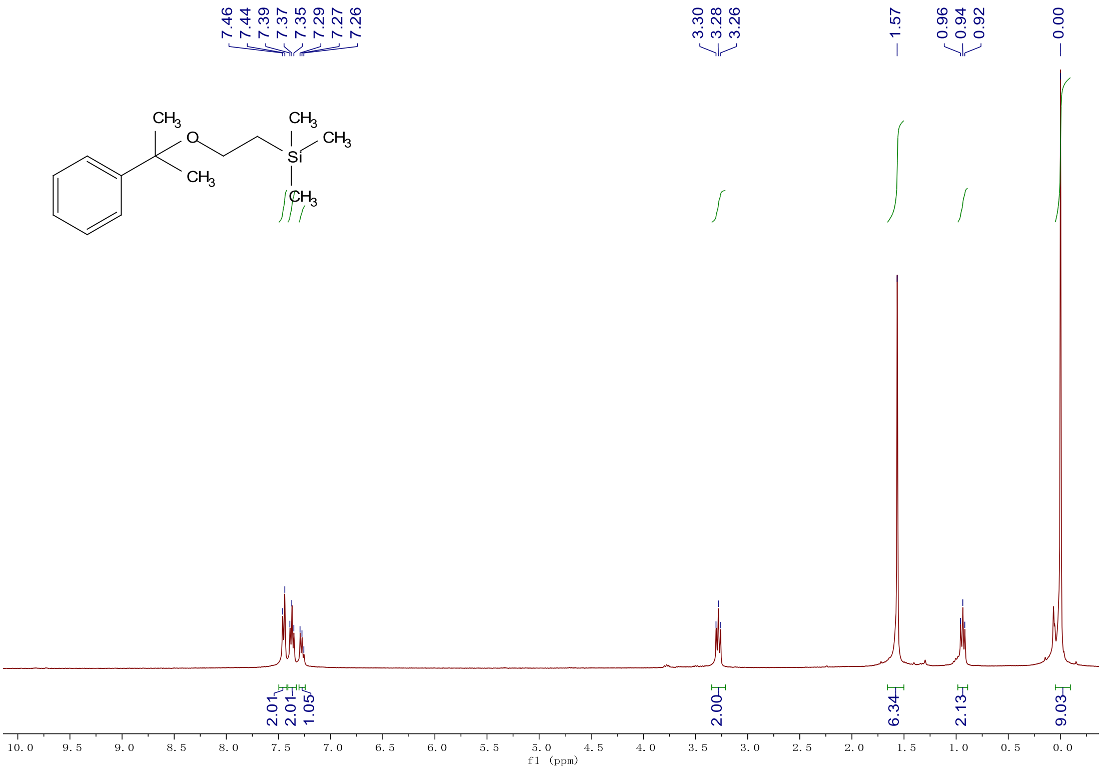

^{1}H NMR (400 MHz, $CDCl_3$) of **10g**

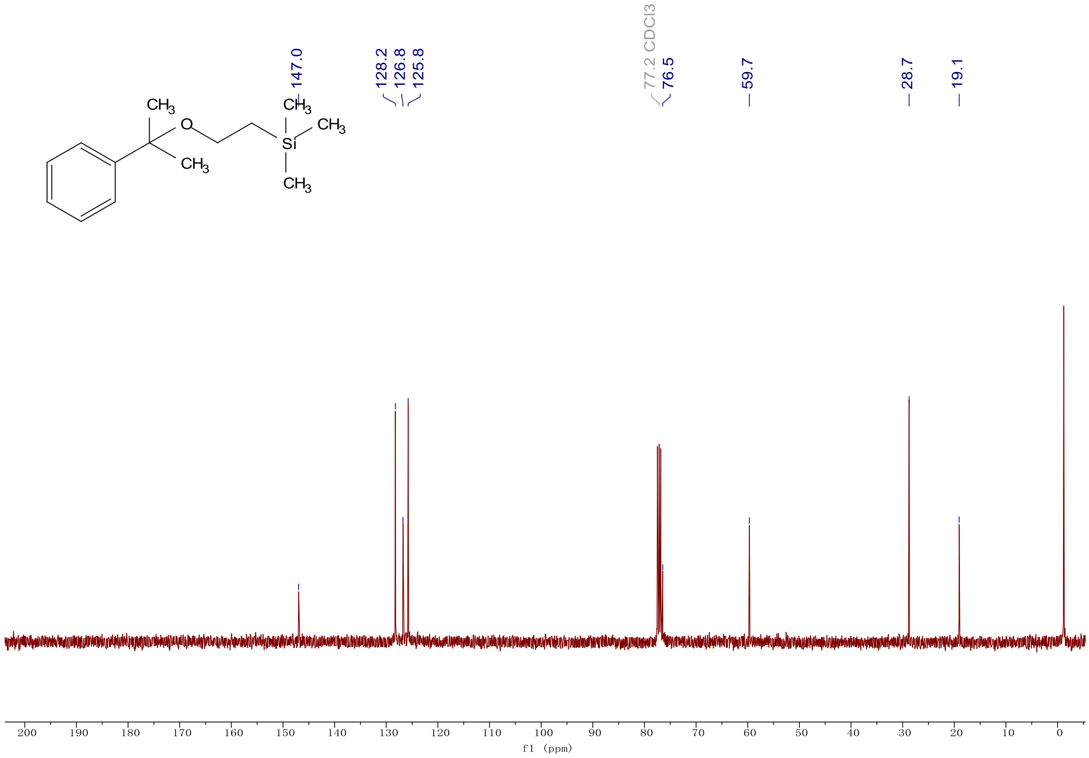

^{13}C NMR (101 MHz, $CDCl_3$) of **10g**

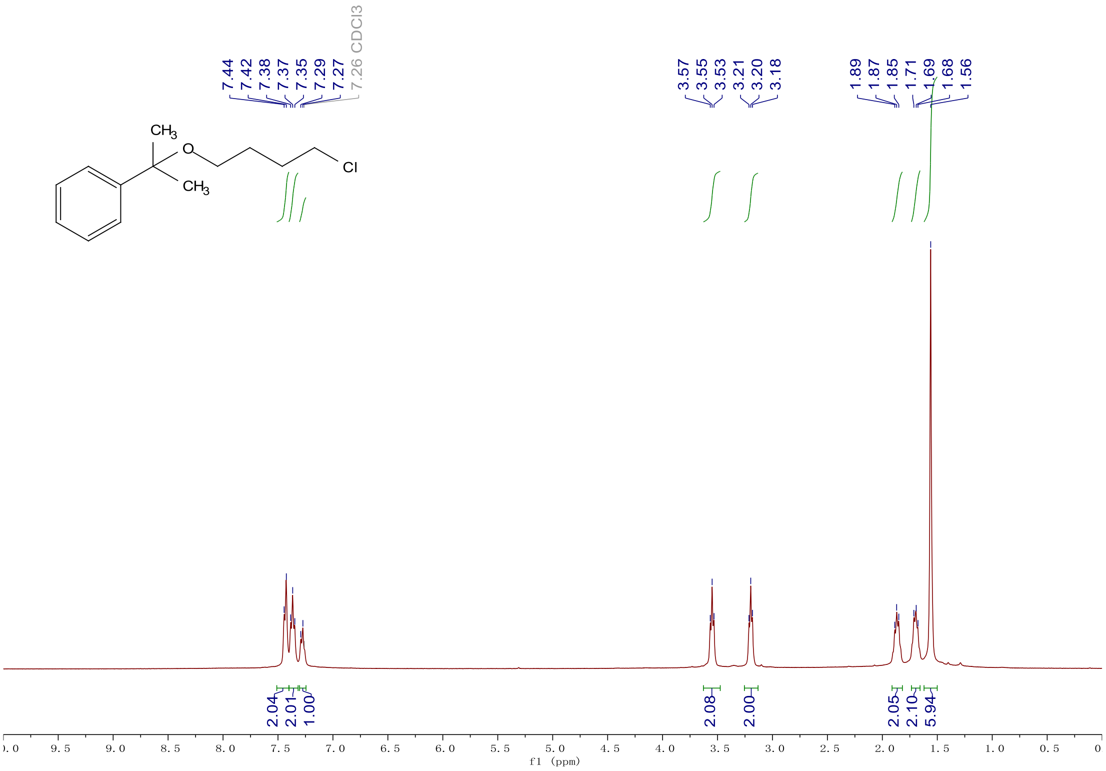

^1H NMR (400 MHz, $CDCl_3$) of **10h**

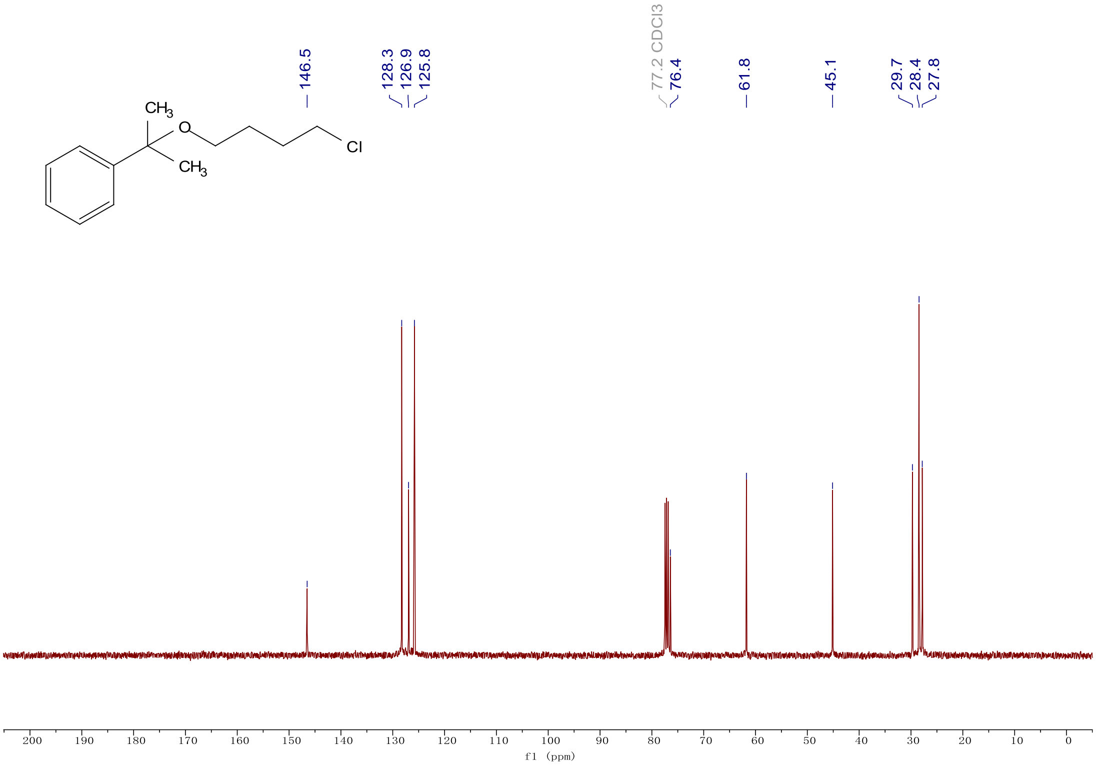

^{13}C NMR (101 MHz, $CDCl_3$) of **10h**

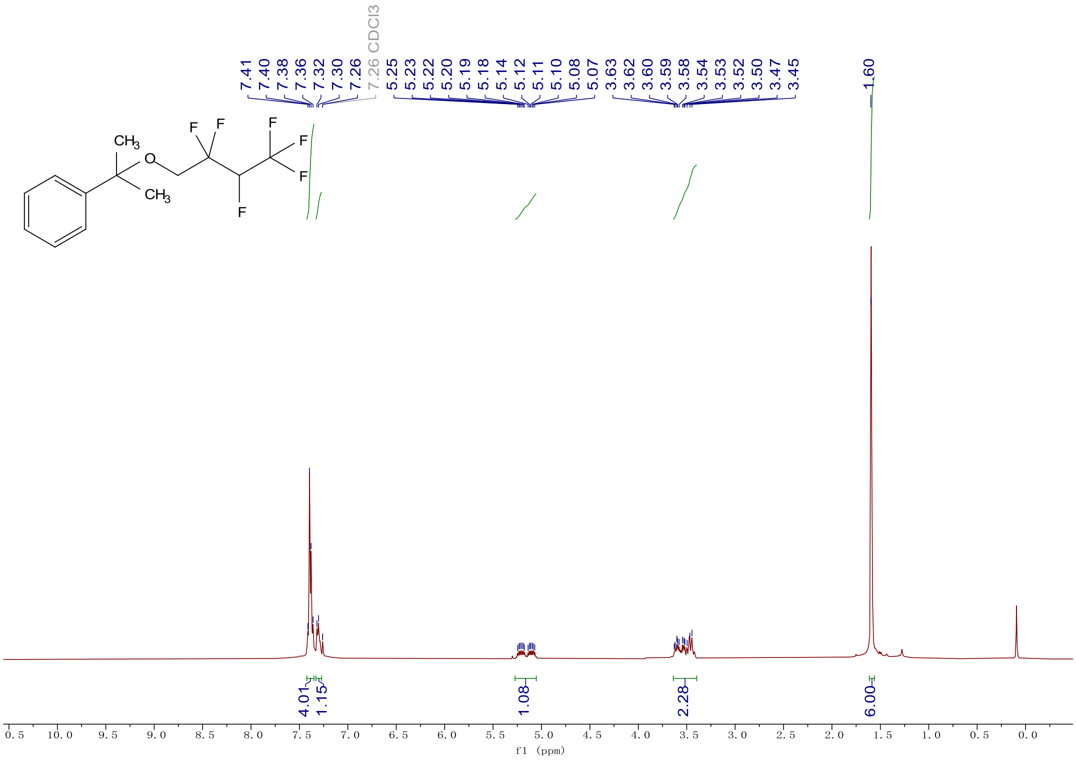

^{1}H NMR (400 MHz, $CDCl_3$) of **10i**

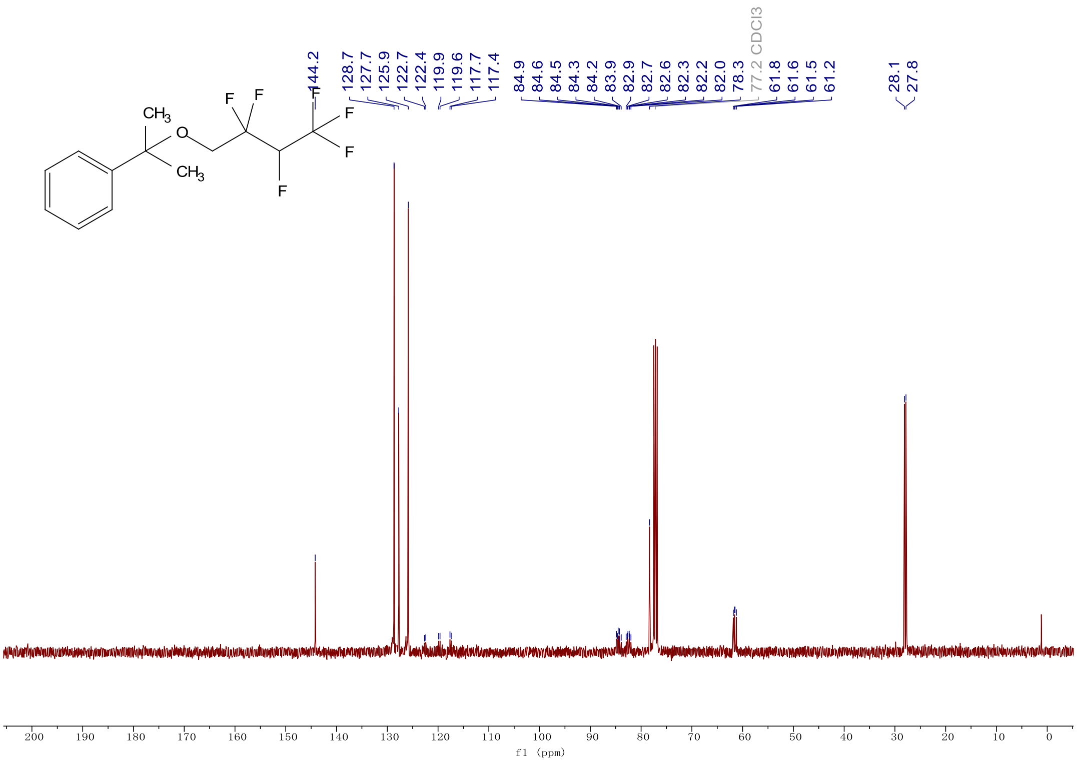

^{13}C NMR (101 MHz, $CDCl_3$) of **10i**

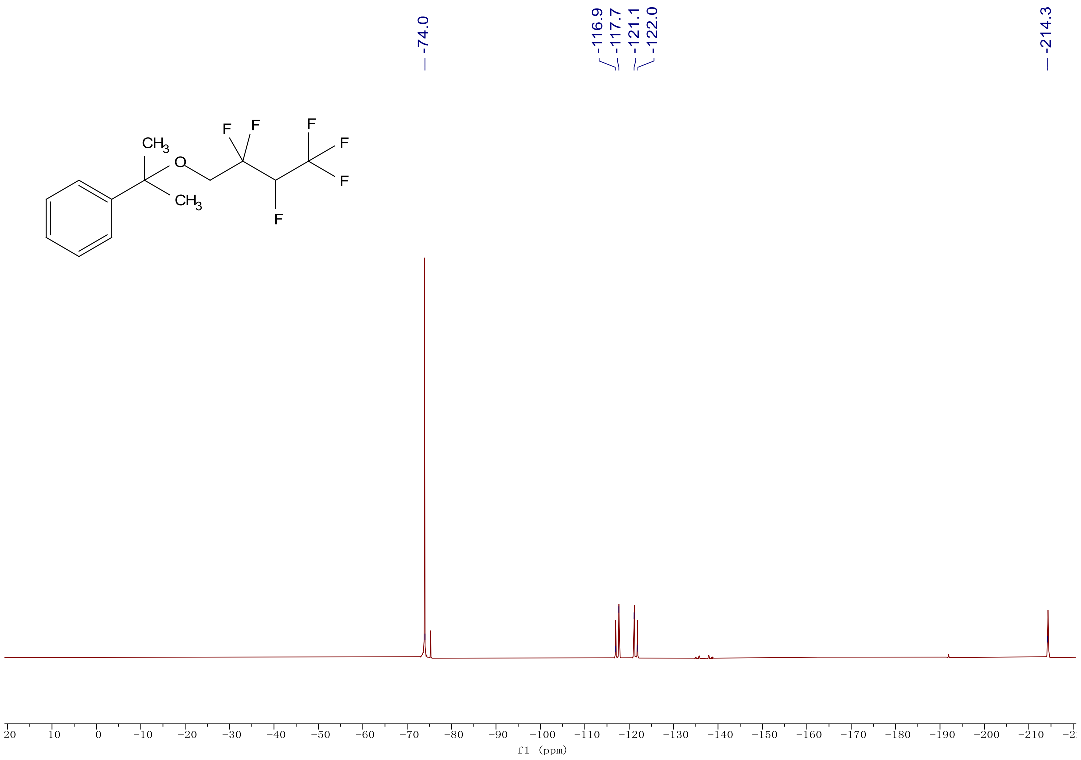

^{19}F NMR (377 MHz, CDCl$_3$) of **10i**

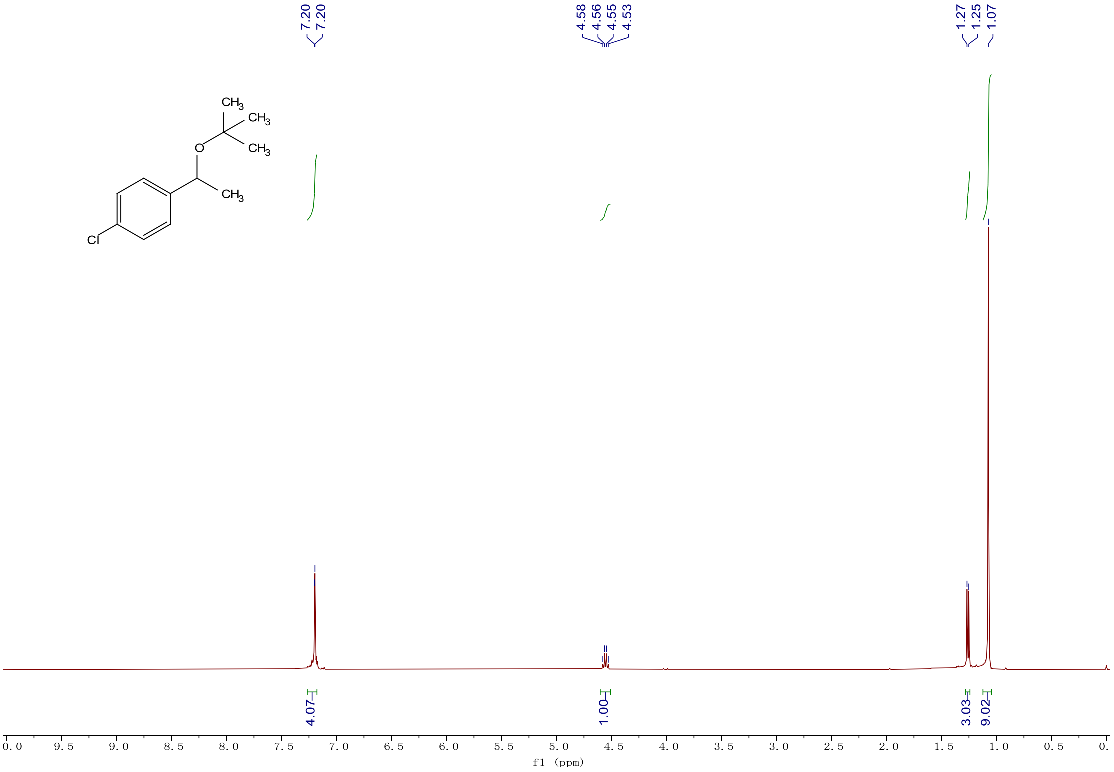

^{1}H NMR (400 MHz, CDCl$_3$) of **10j**

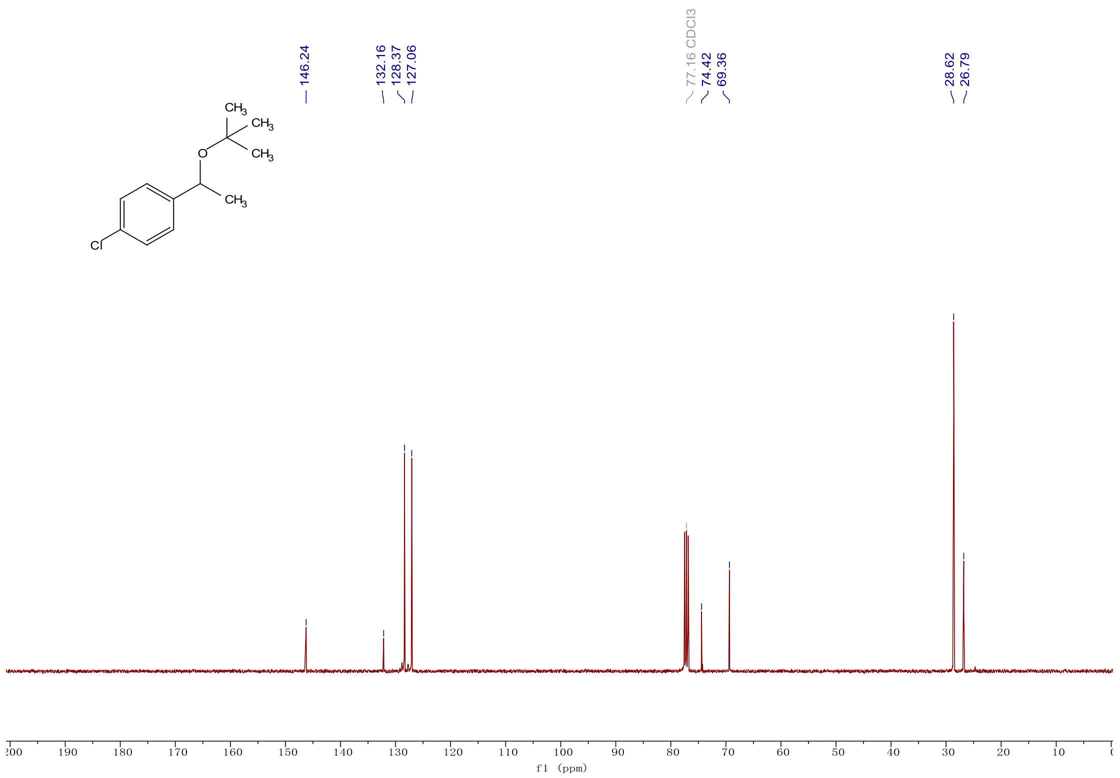

^{13}C NMR (101 MHz, $CDCl_3$) of **10j**

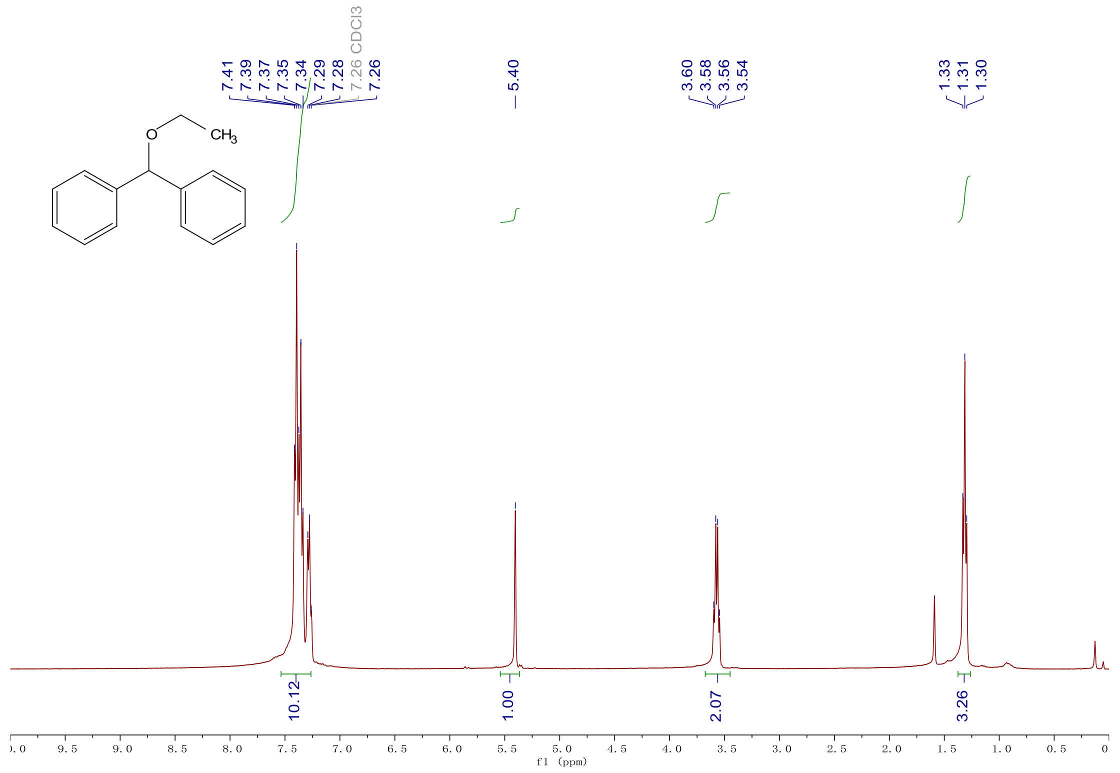

^{1}H NMR (400 MHz, $CDCl_3$) of **10k**

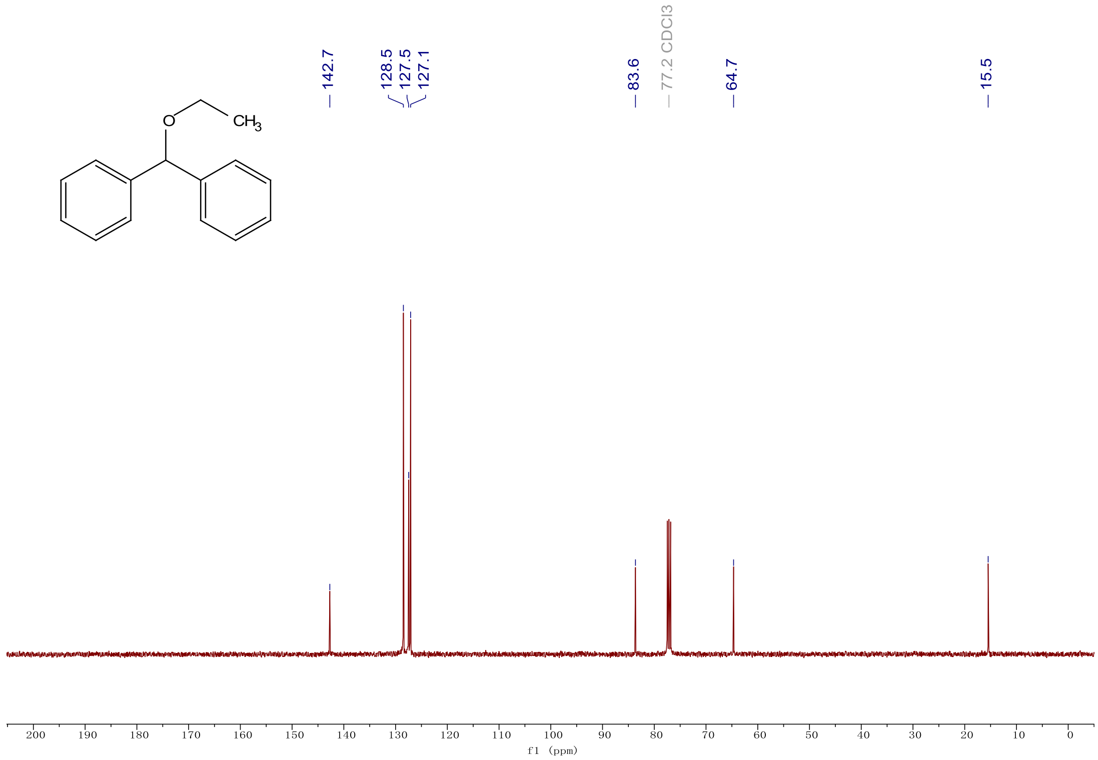

^{13}C NMR (101 MHz, $CDCl_3$) of **10k**

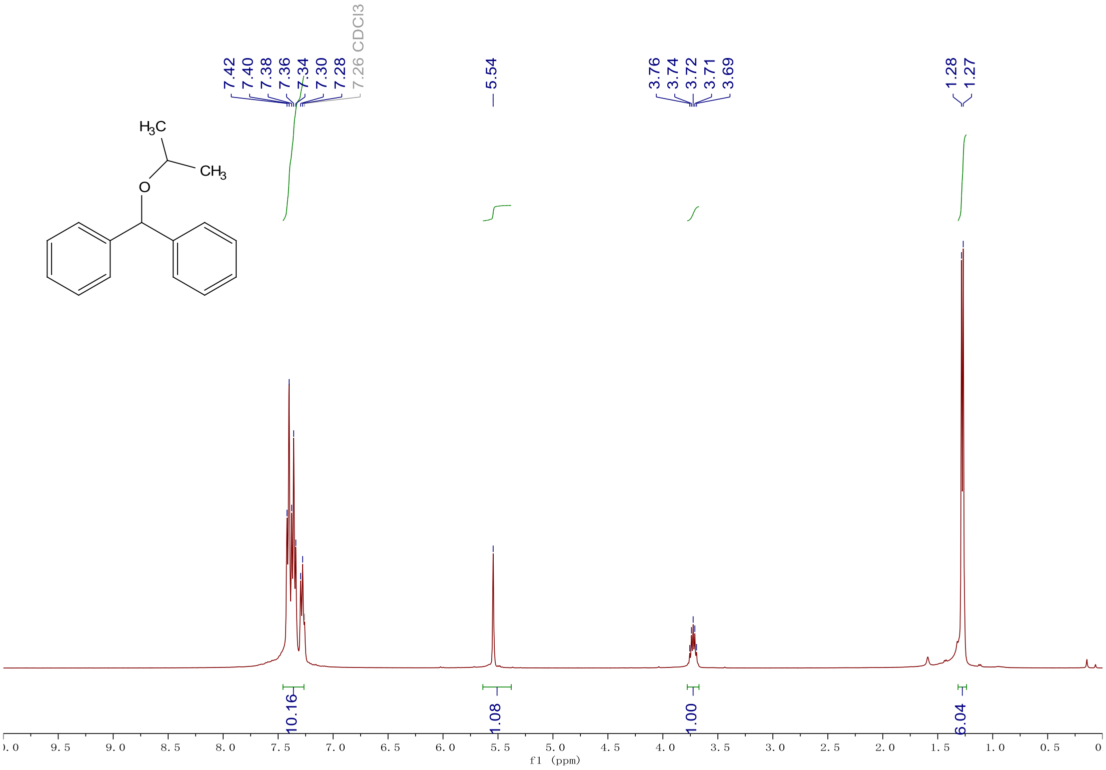

^{1}H NMR (400 MHz, $CDCl_3$) of **10l**

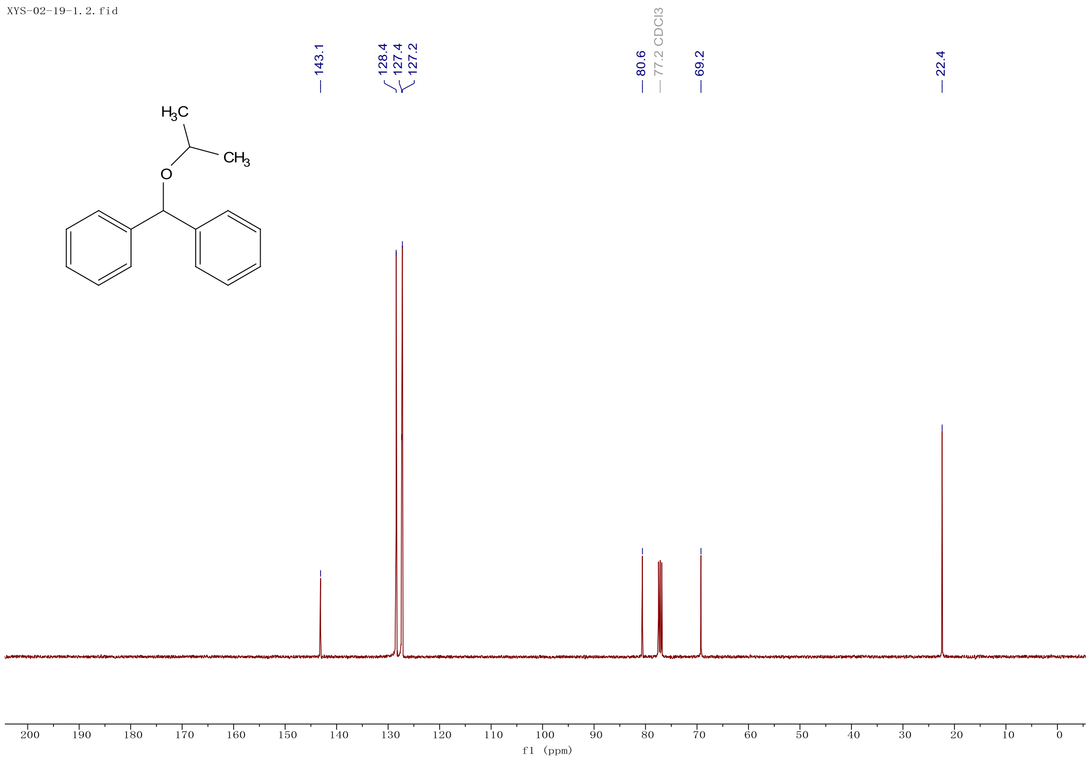

^{13}C NMR (101 MHz, $CDCl_3$) of **10l**

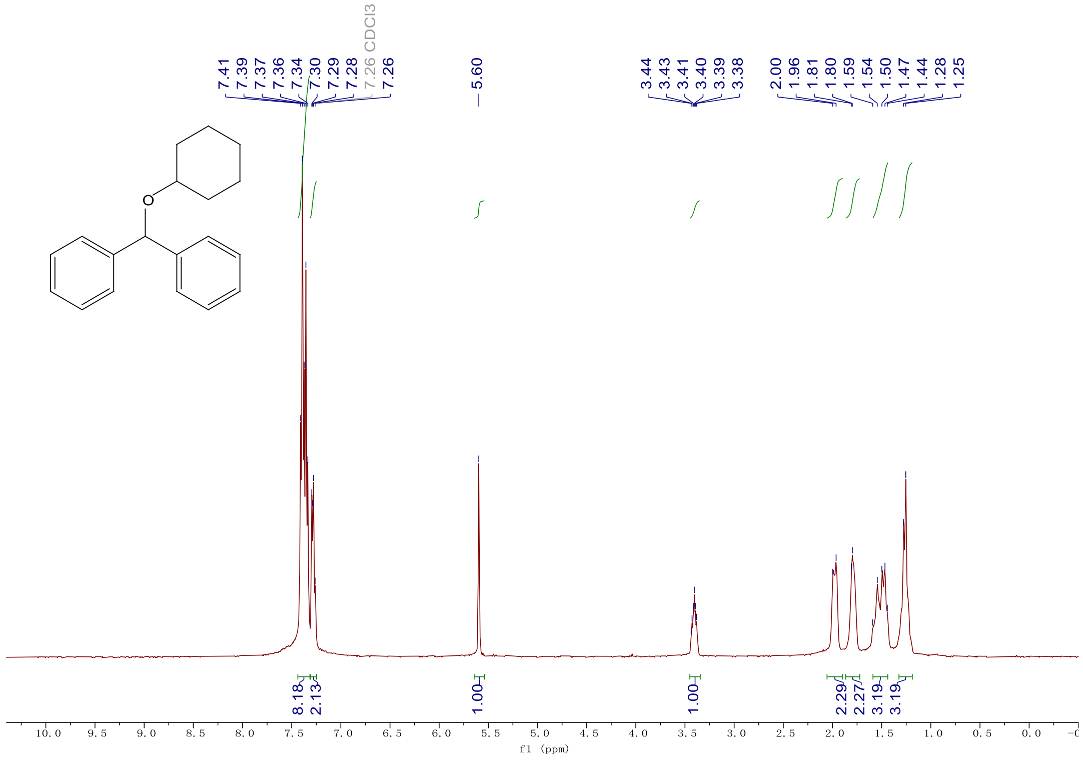

^{1}H NMR (400 MHz, $CDCl_3$) of **10m**

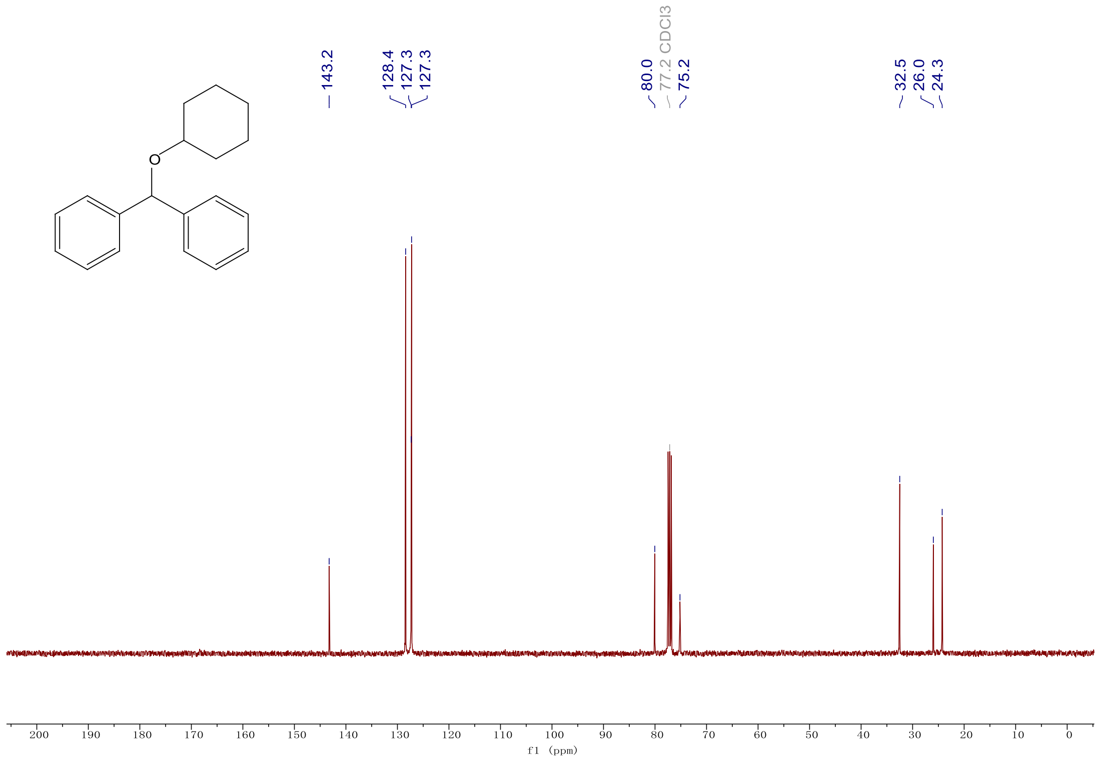

^{13}C NMR (101 MHz, $CDCl_3$) of **10m**

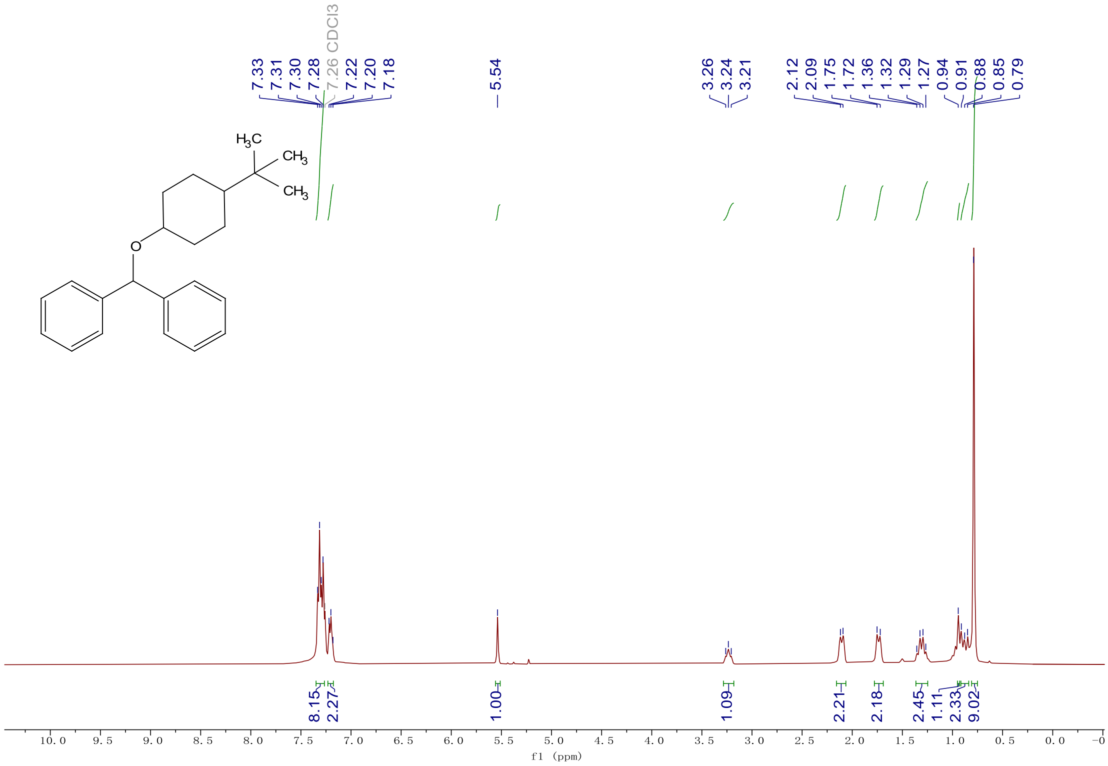

^{1}H NMR (400 MHz, $CDCl_3$) of **10n**

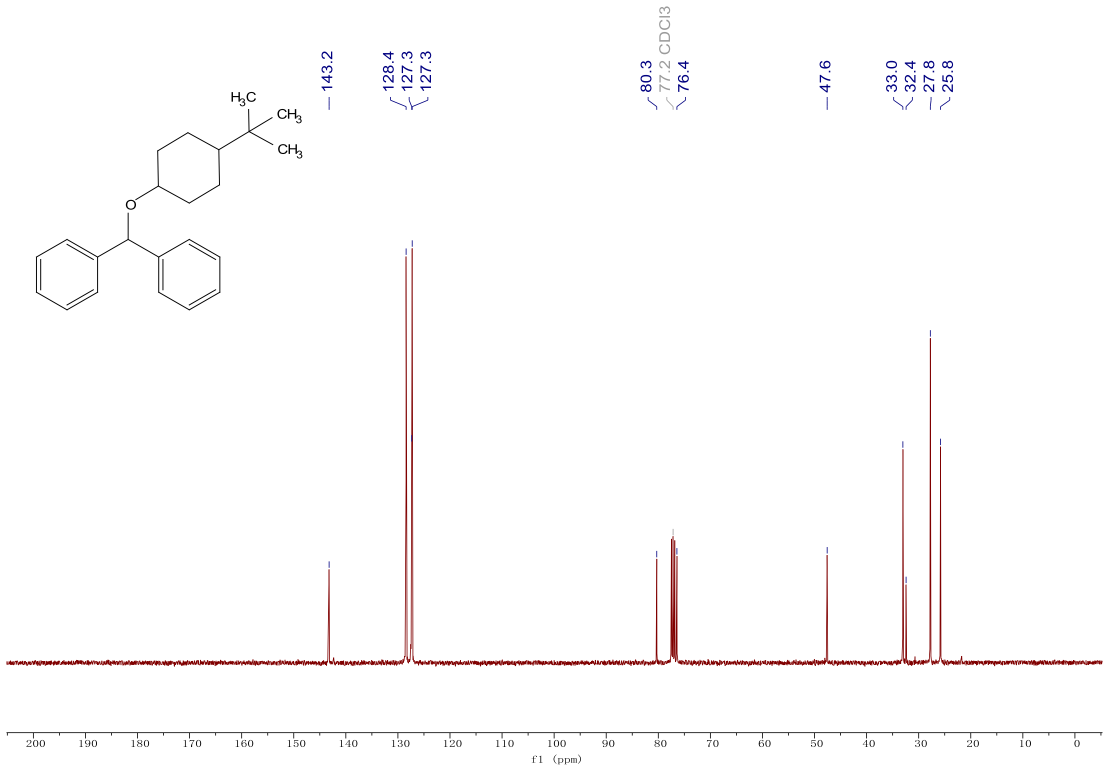

^{13}C NMR (101 MHz, $CDCl_3$) of **10n**

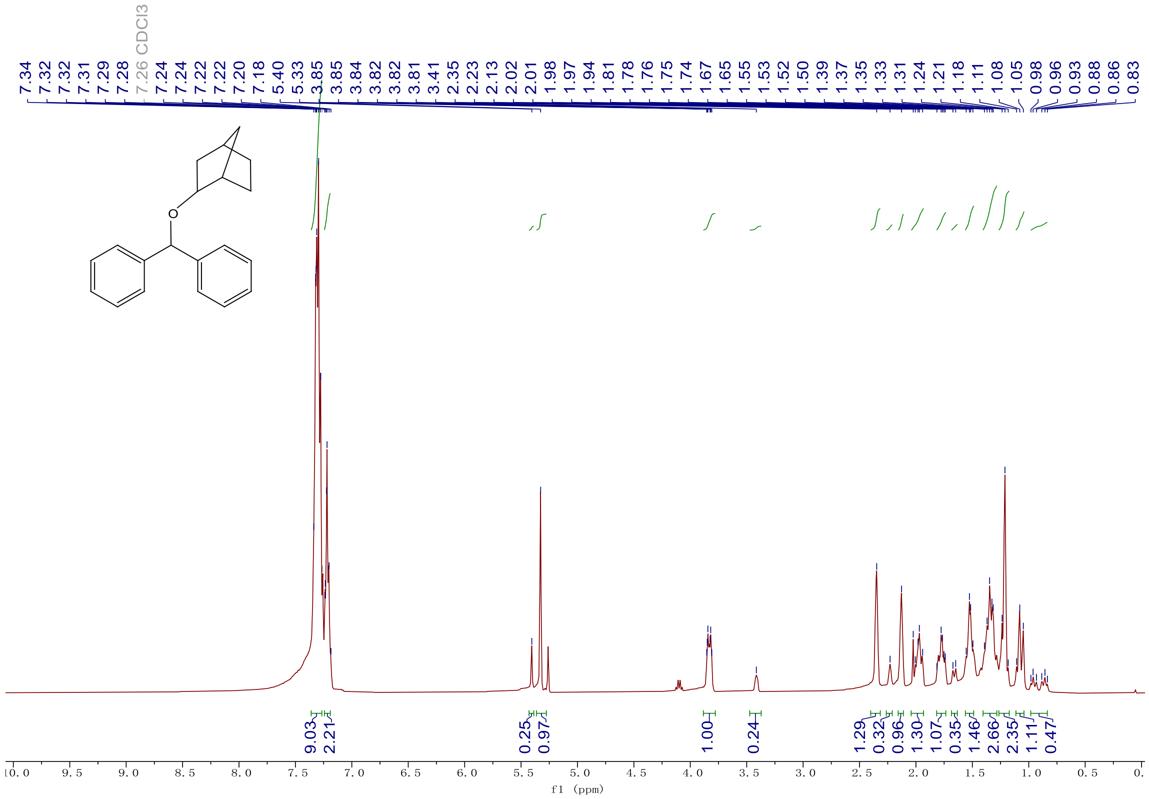

^{1}H NMR (400 MHz, $CDCl_3$) of **10o**

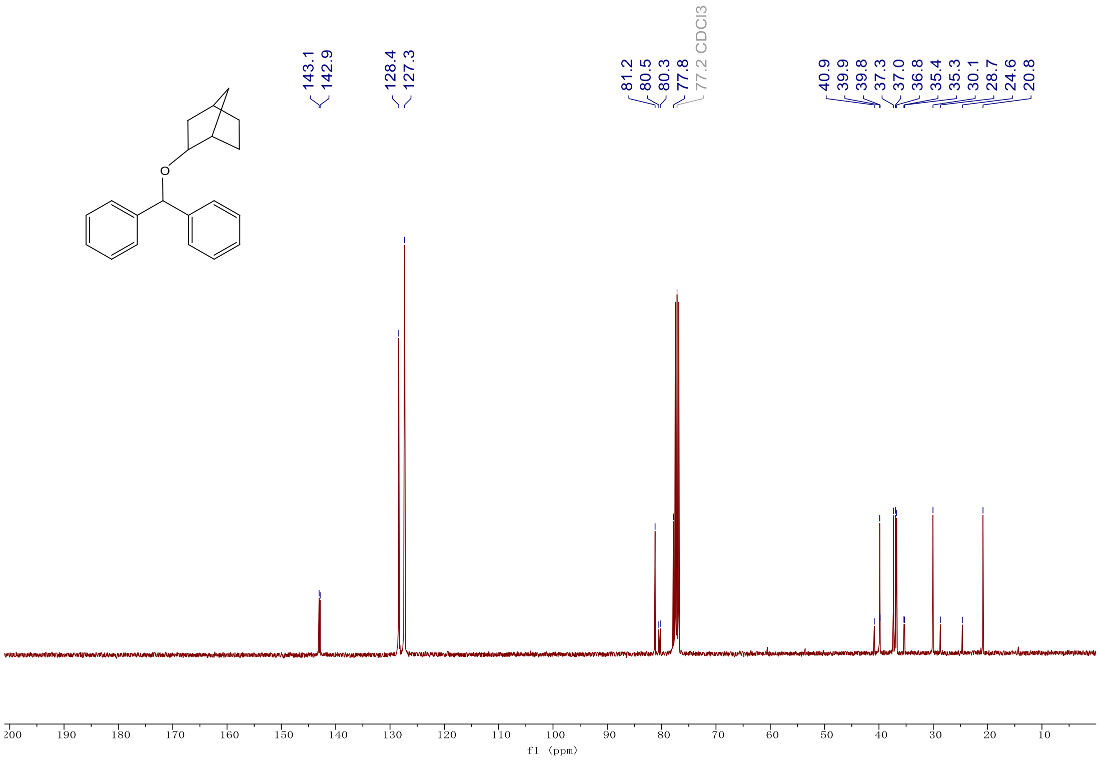

^{13}C NMR (101 MHz, $CDCl_3$) of **10o**

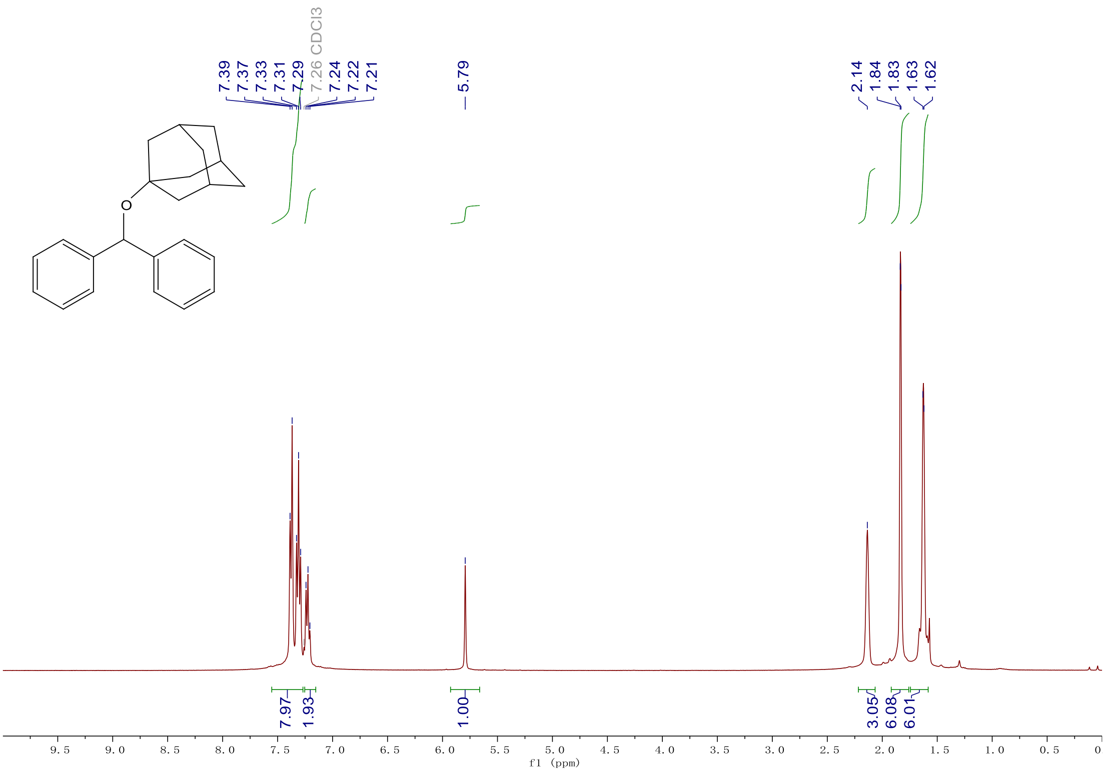

^{1}H NMR (400 MHz, $CDCl_3$) of **10p**

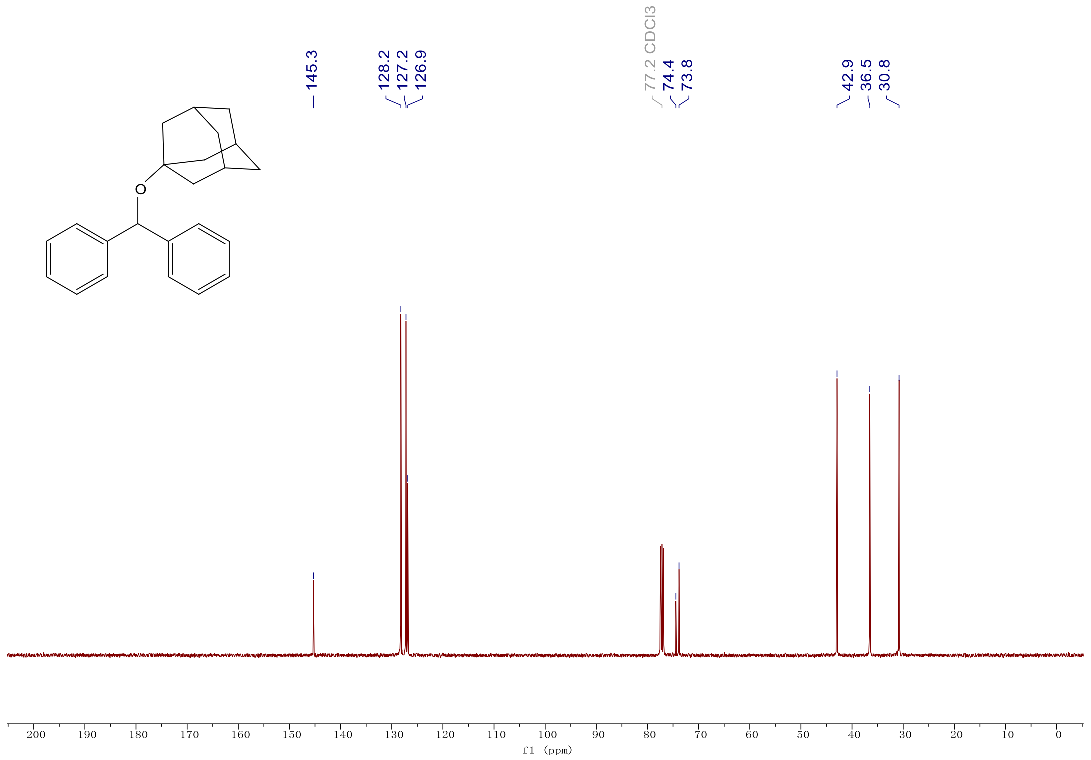

^{13}C NMR (101 MHz, CDCl$_3$) of **10p**

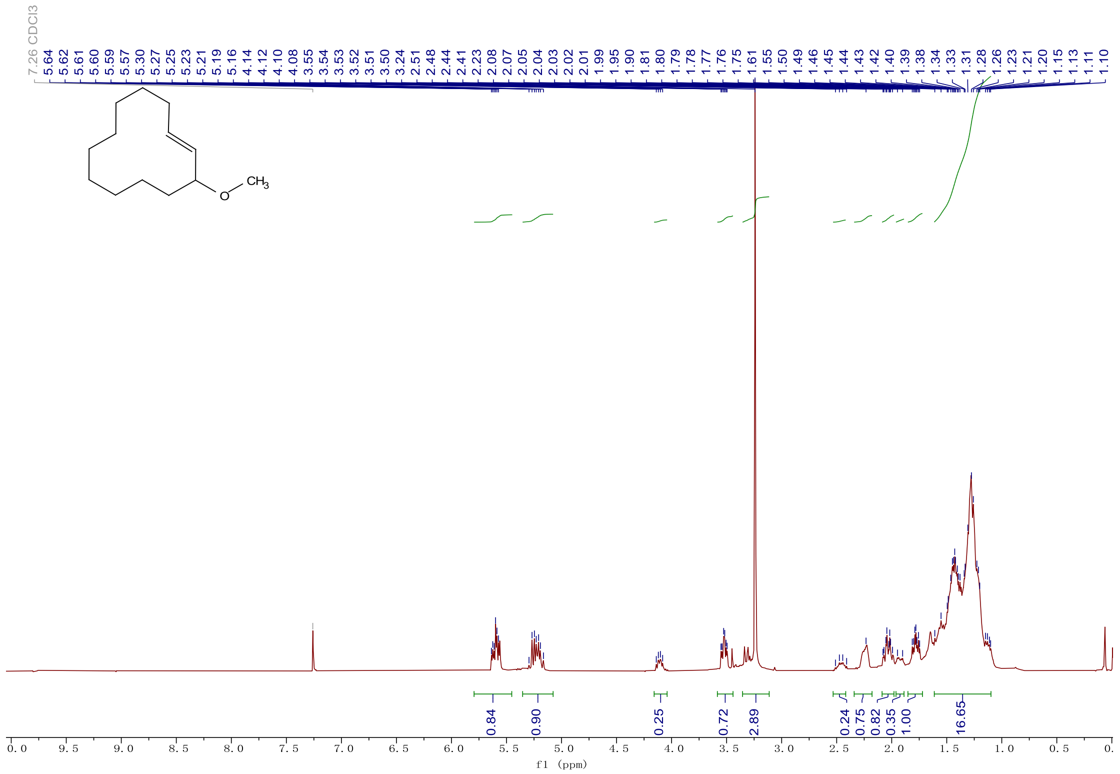

^{1}H NMR (400 MHz, CDCl$_3$) of **11a**

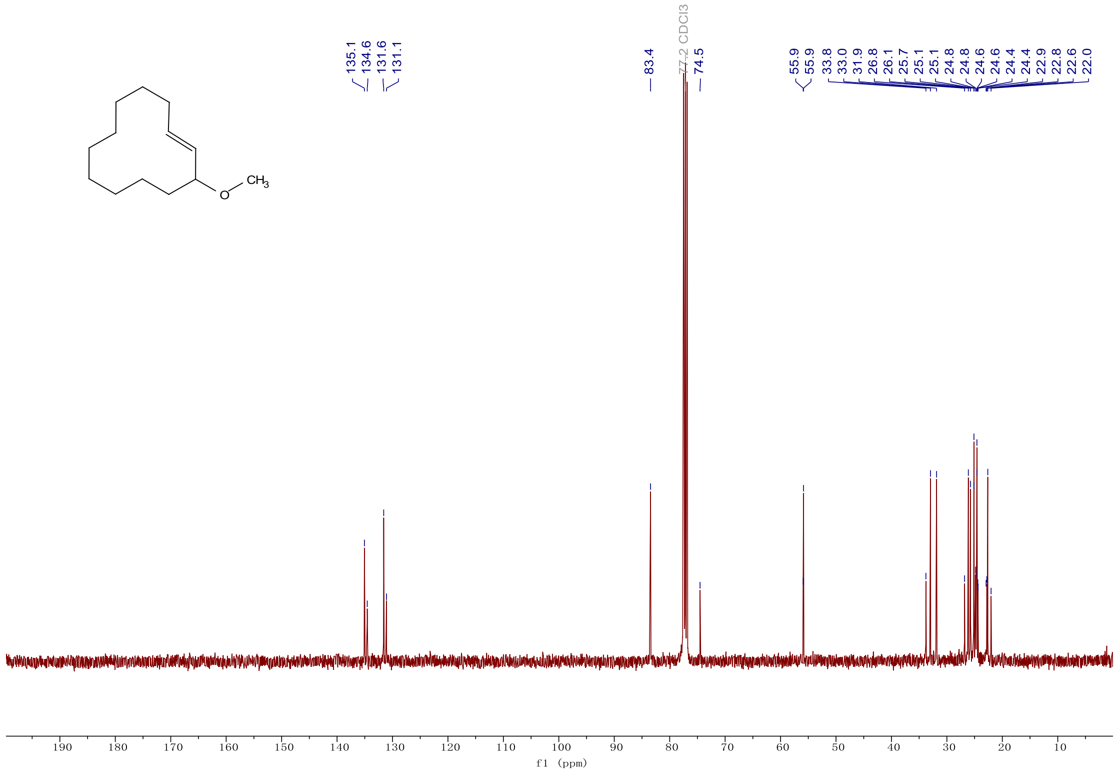

^{13}C NMR (101 MHz, $CDCl_3$) of **11a**

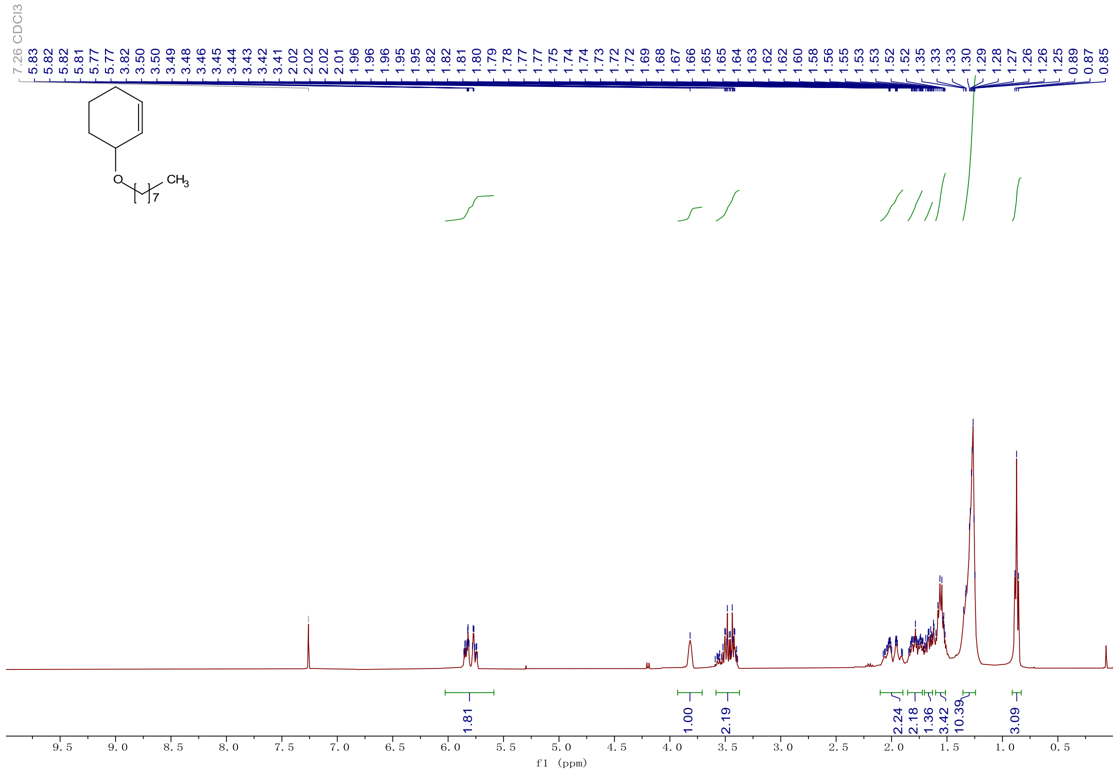

^{1}H NMR (400 MHz, $CDCl_3$) of **11b**

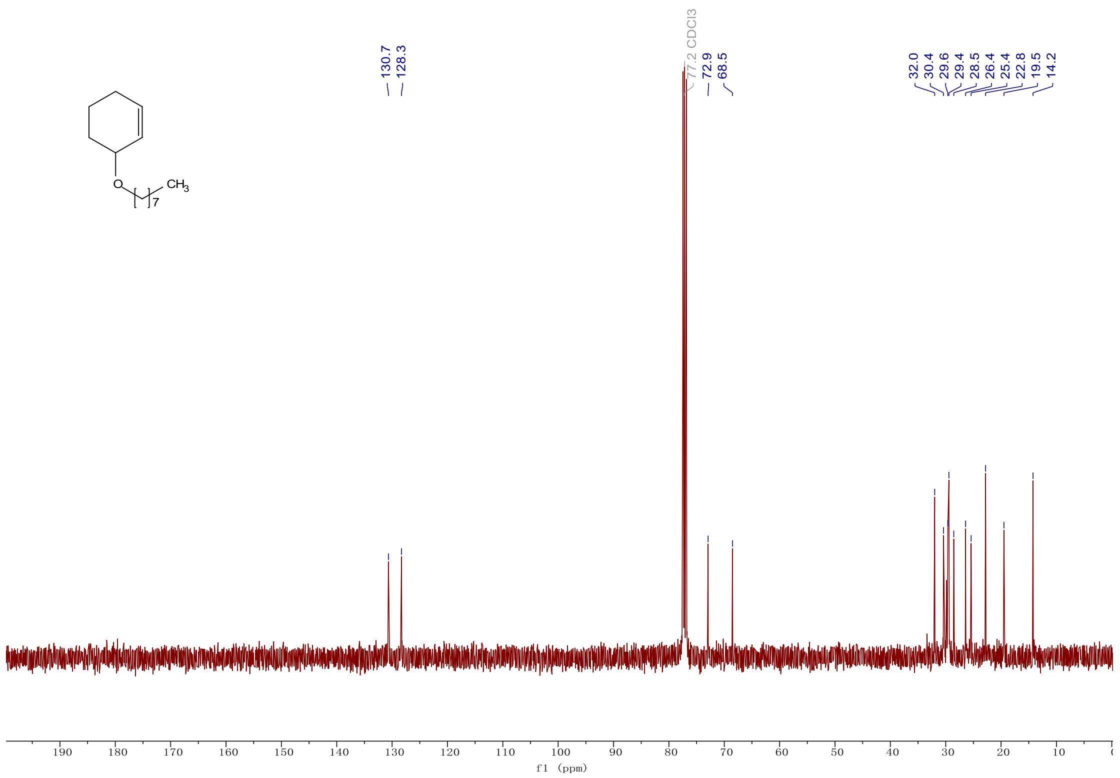

^{13}C NMR (101 MHz, $CDCl_3$) of **11b**

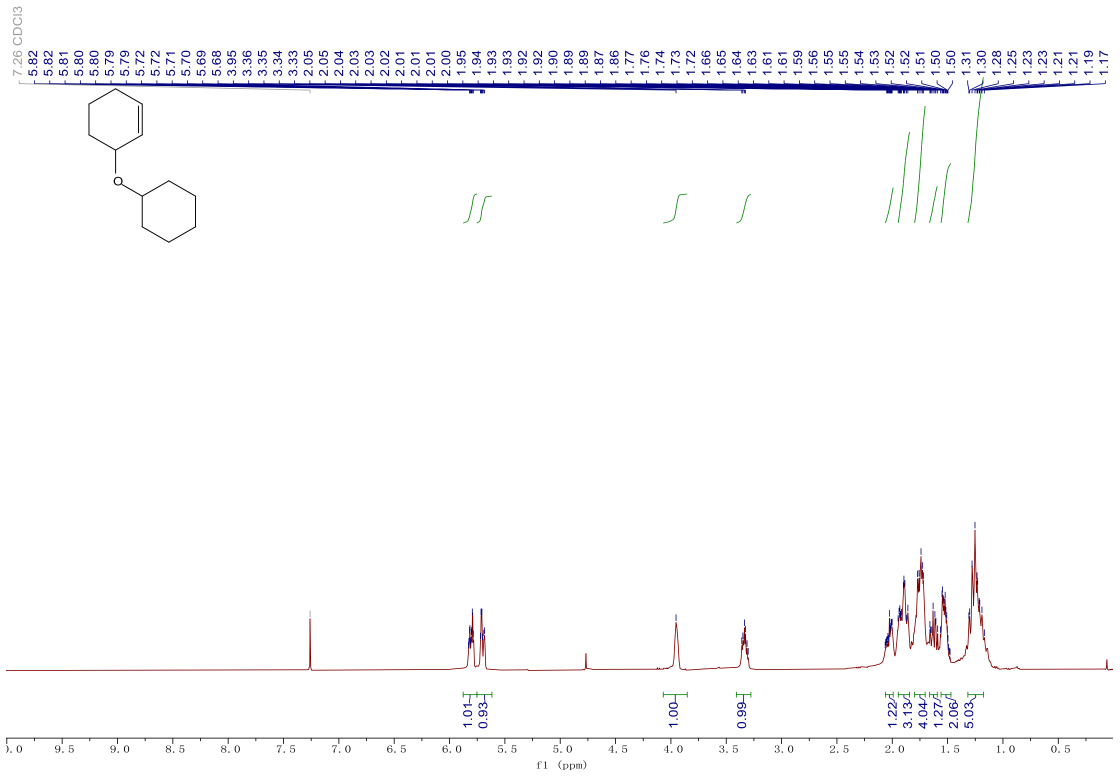

^{1}H NMR (400 MHz, $CDCl_3$) of **11c**

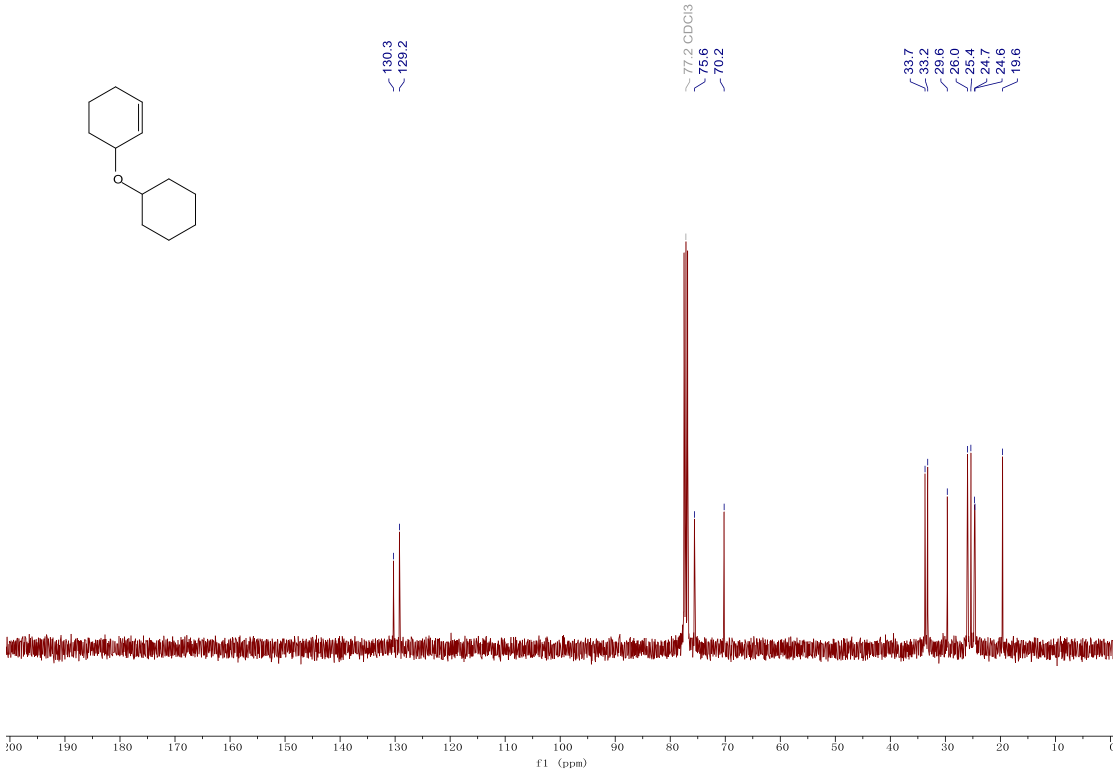

^{13}C NMR (101 MHz, $CDCl_3$) of **11c**

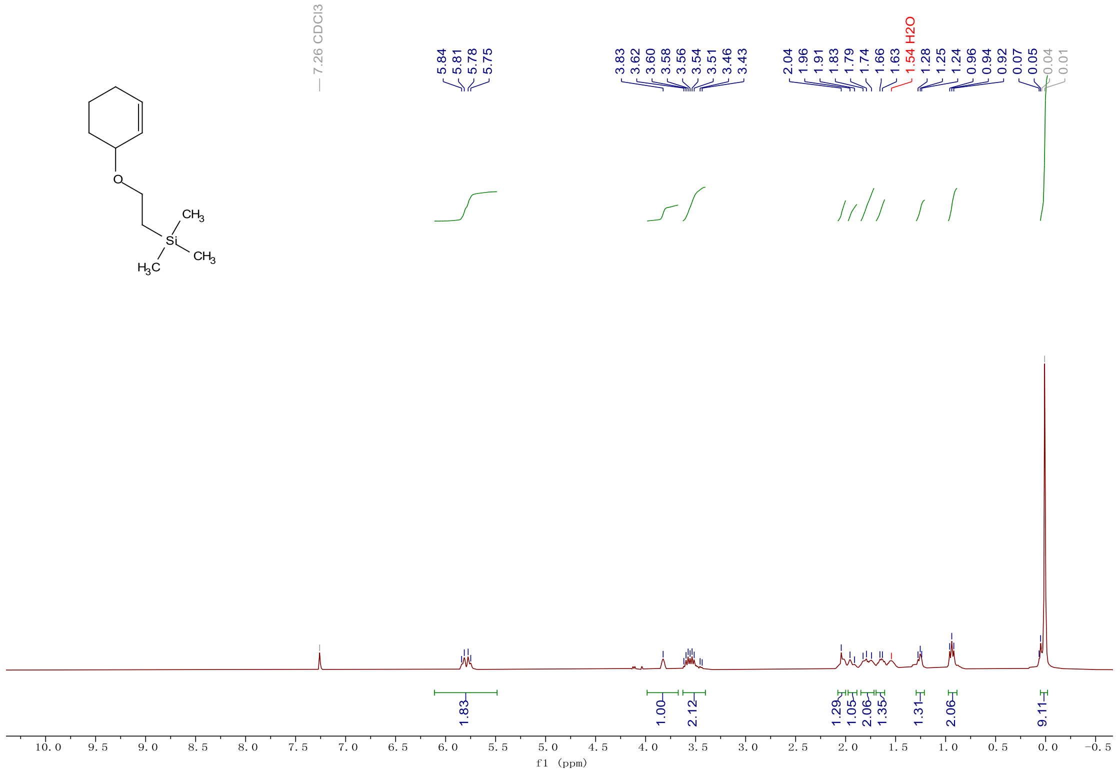

^{1}H NMR (400 MHz, $CDCl_3$) of **11d**

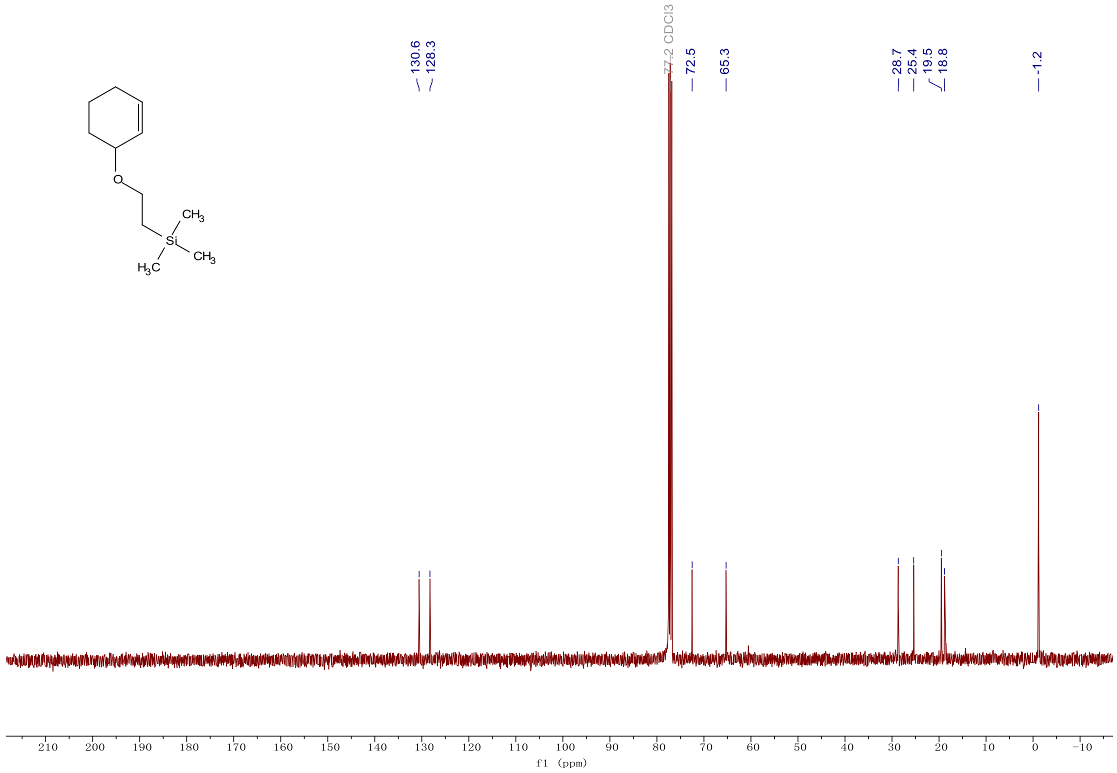

^{13}C NMR (101 MHz, $CDCl_3$) of **11d**

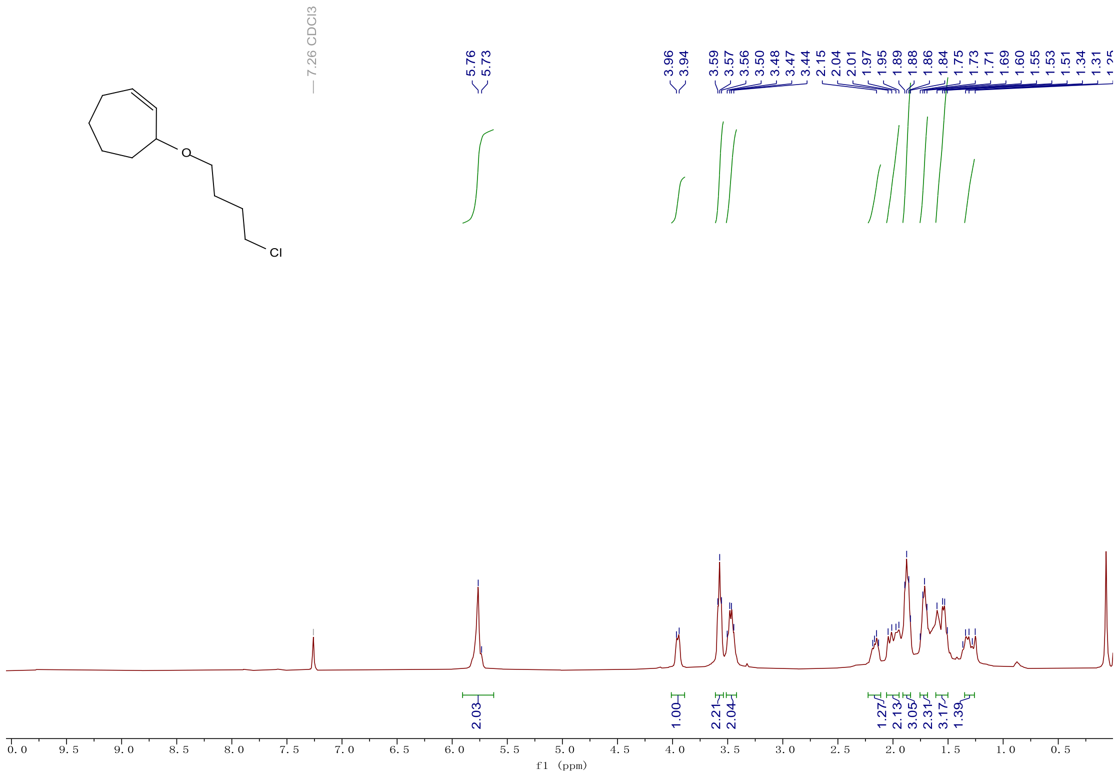

^{1}H NMR (400 MHz, $CDCl_3$) of **11e**

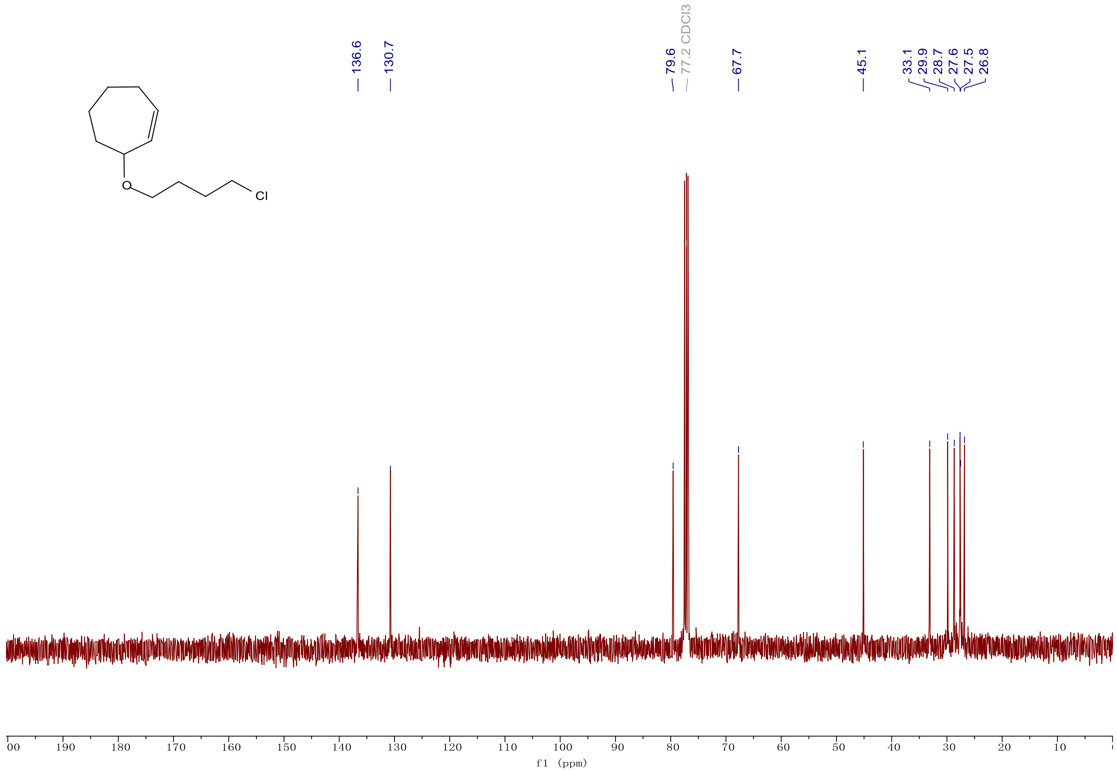

^{13}C NMR (101 MHz, $CDCl_3$) of **11e**

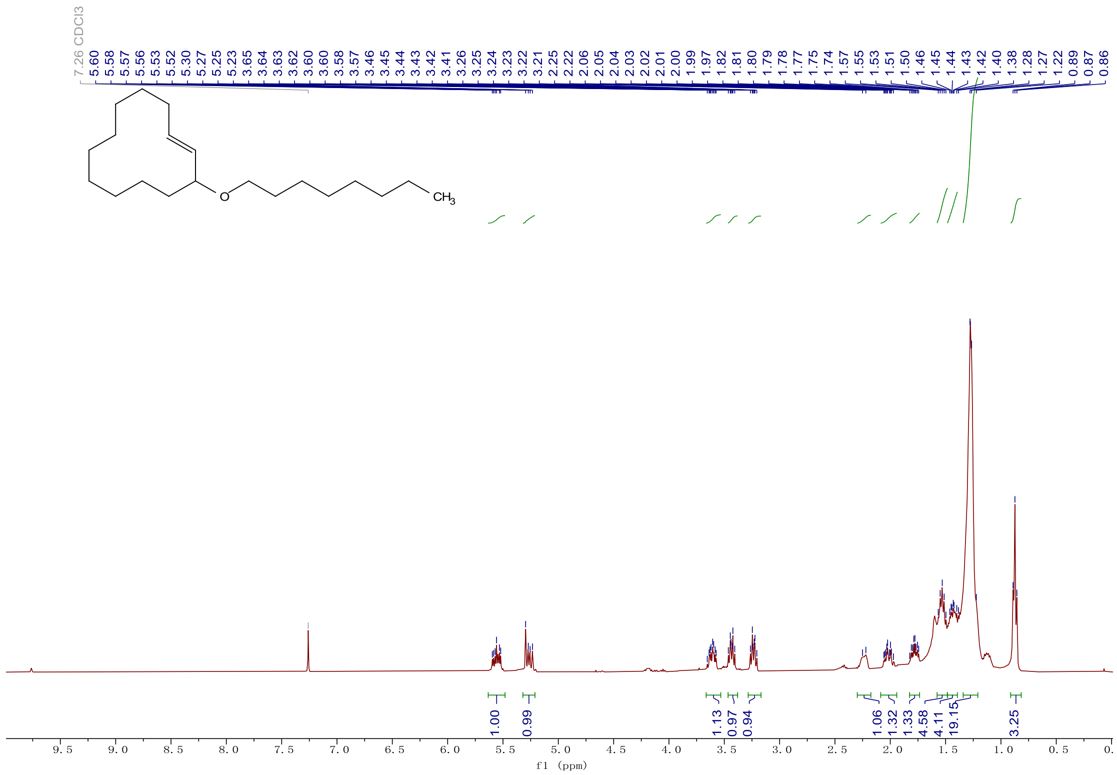

^{1}H NMR (400 MHz, $CDCl_3$) of **11f**

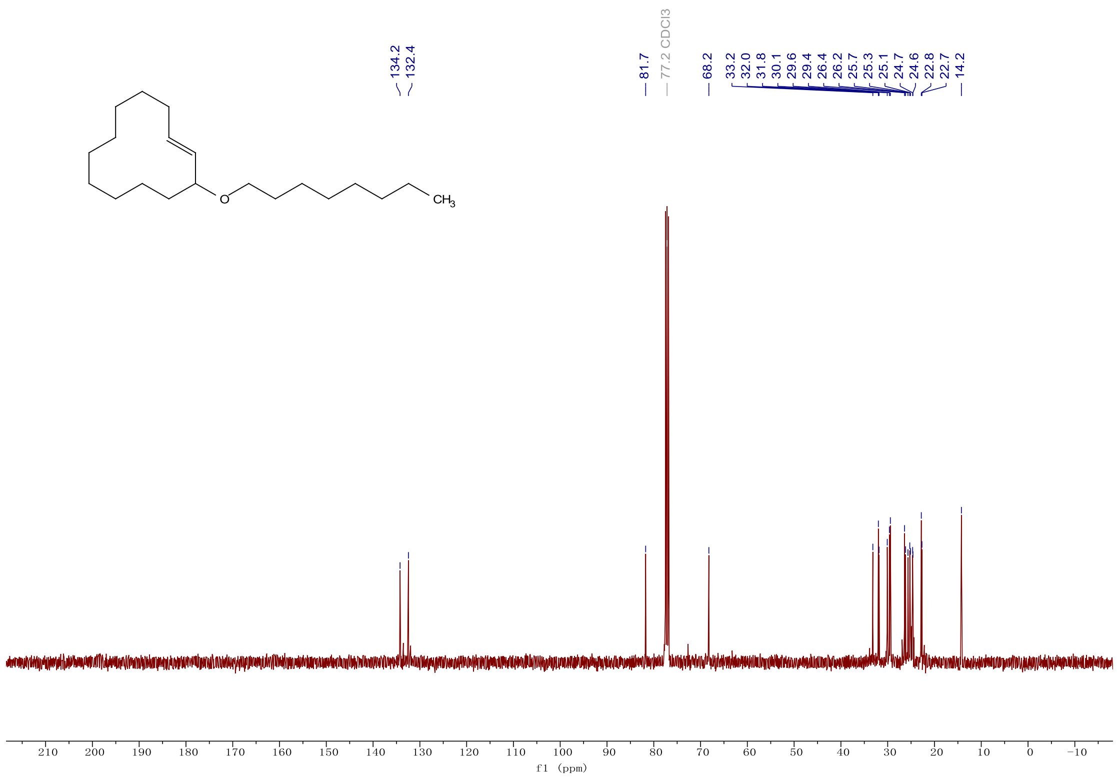

^{13}C NMR (101 MHz, $CDCl_3$) of **11f**

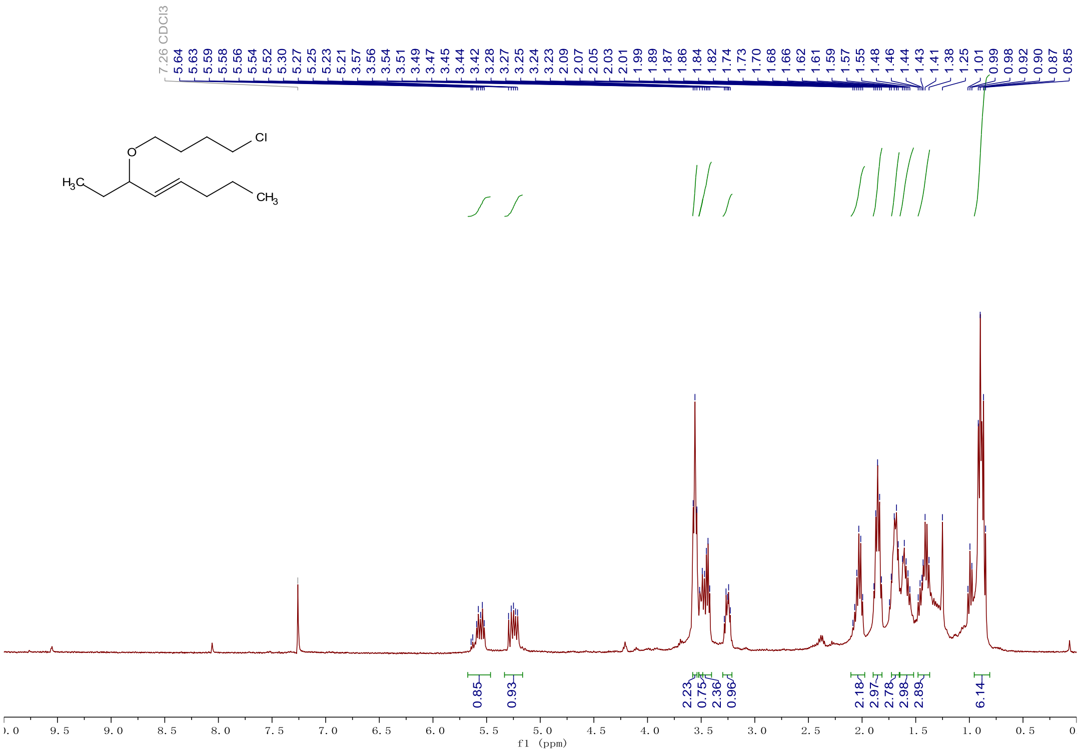

^{1}H NMR (400 MHz, $CDCl_3$) of **11g**

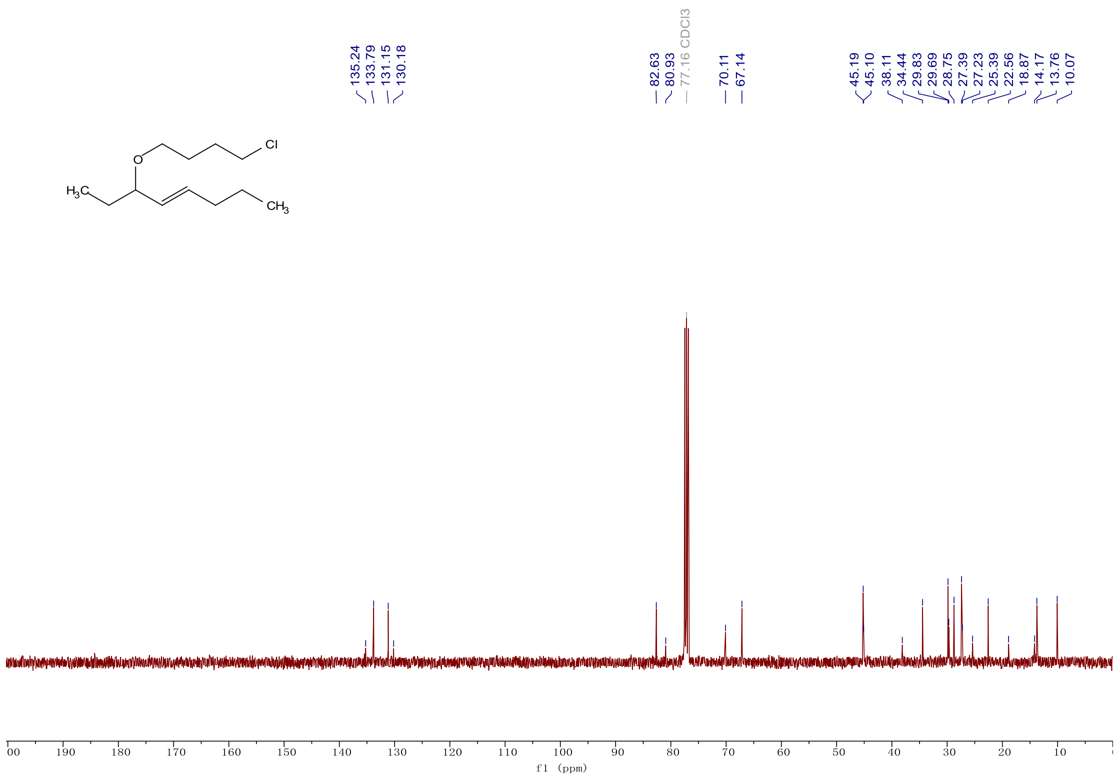

^{13}C NMR (101 MHz, $CDCl_3$) of **11g**

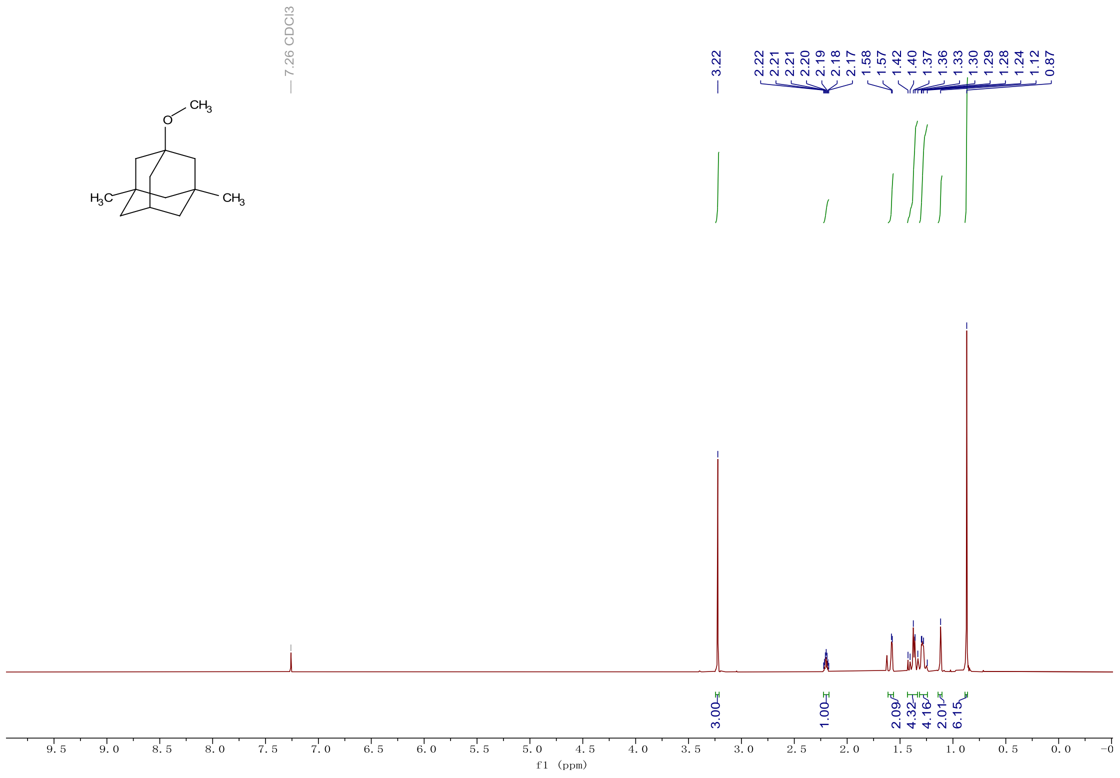

^{1}H NMR (400 MHz, $CDCl_3$) of **11h**

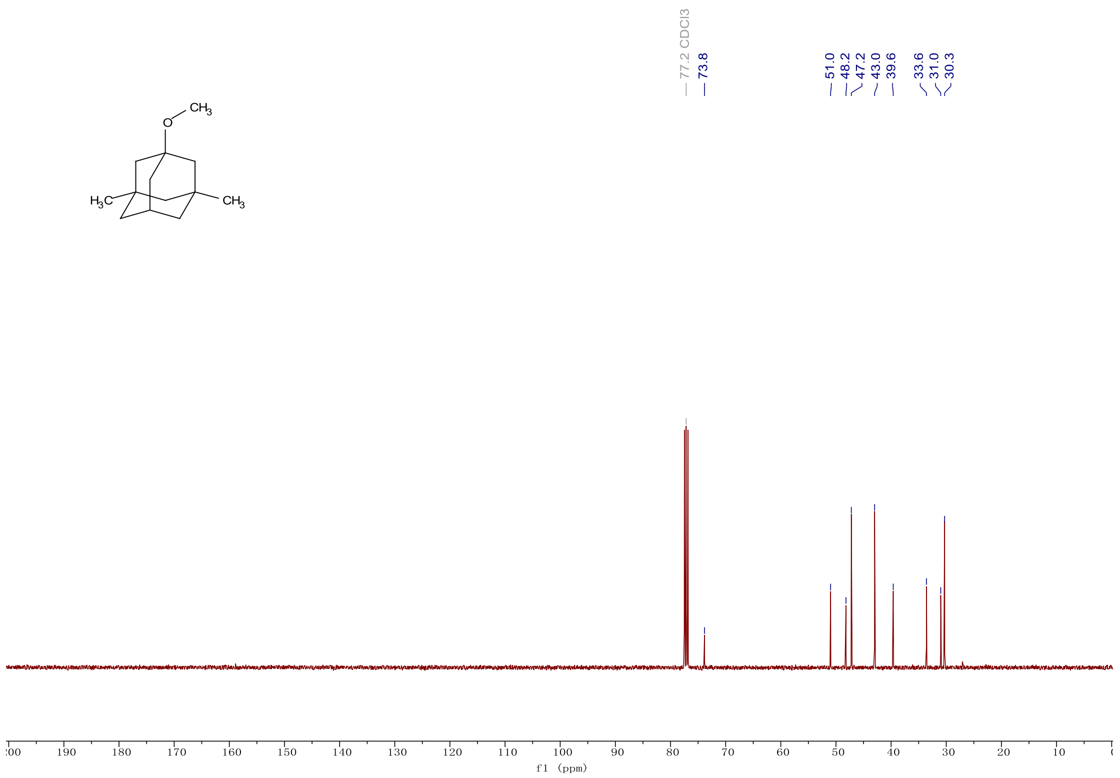

^{13}C NMR (101 MHz, $CDCl_3$) of **11h**

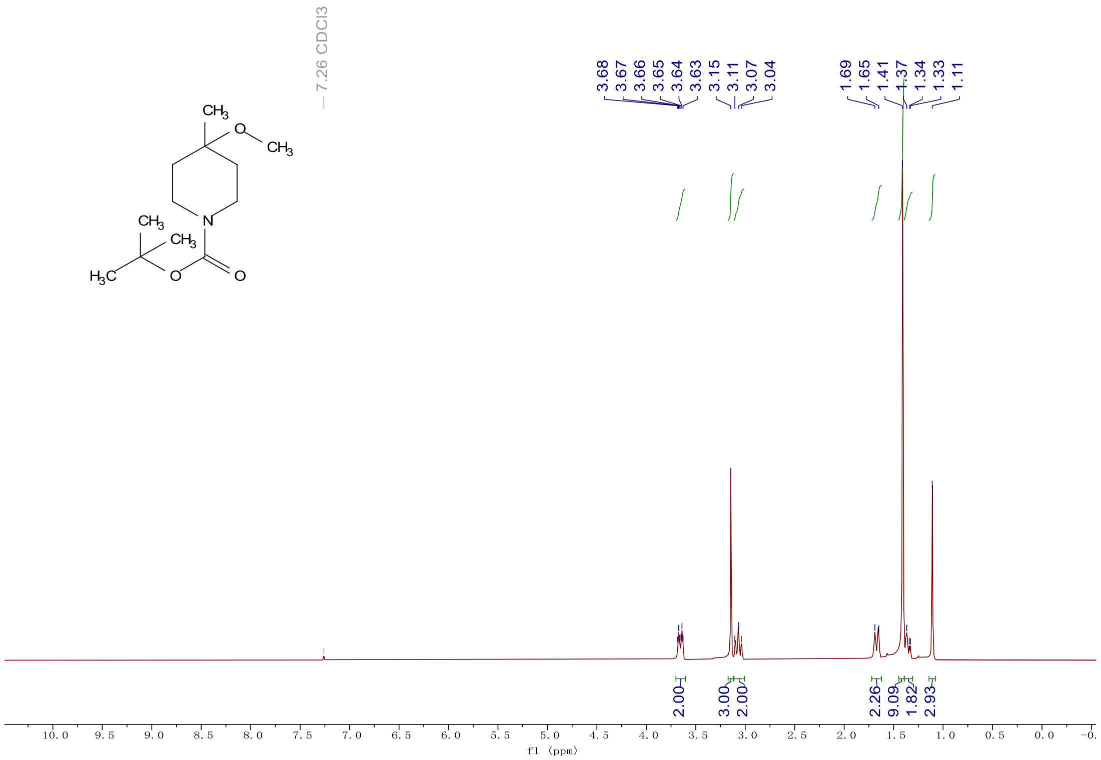

^{1}H NMR (400 MHz, $CDCl_3$) of **11i**

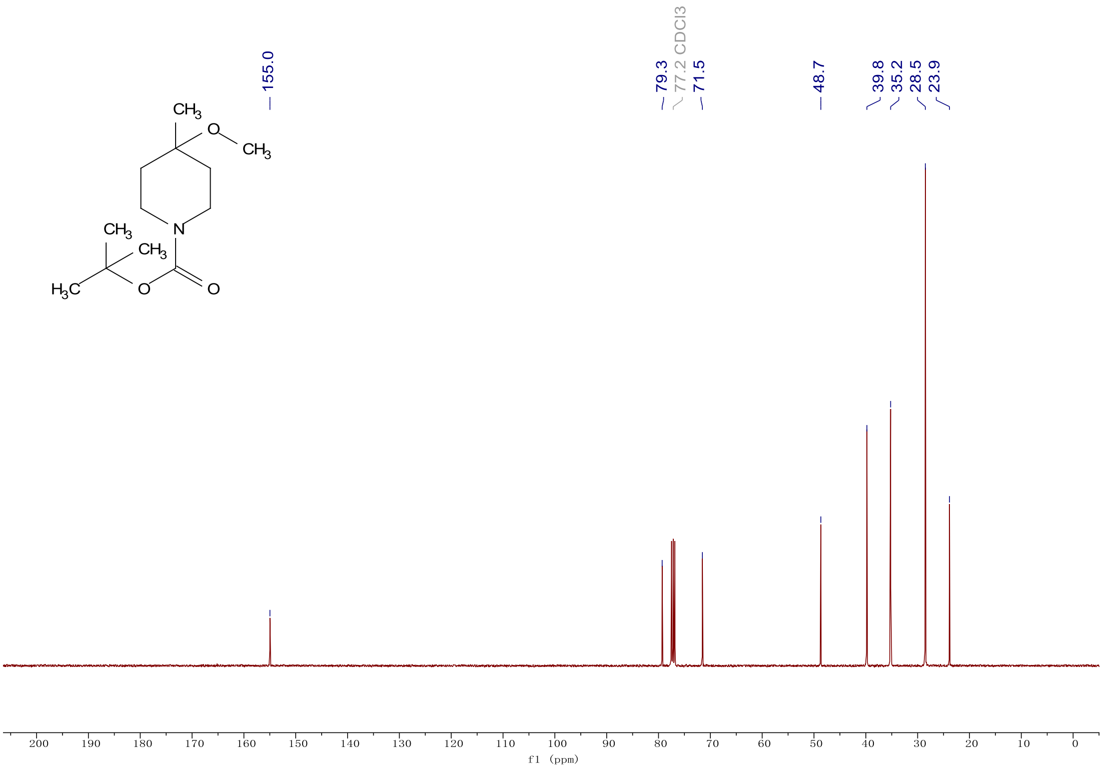

^{13}C NMR (101 MHz, $CDCl_3$) of **11i**

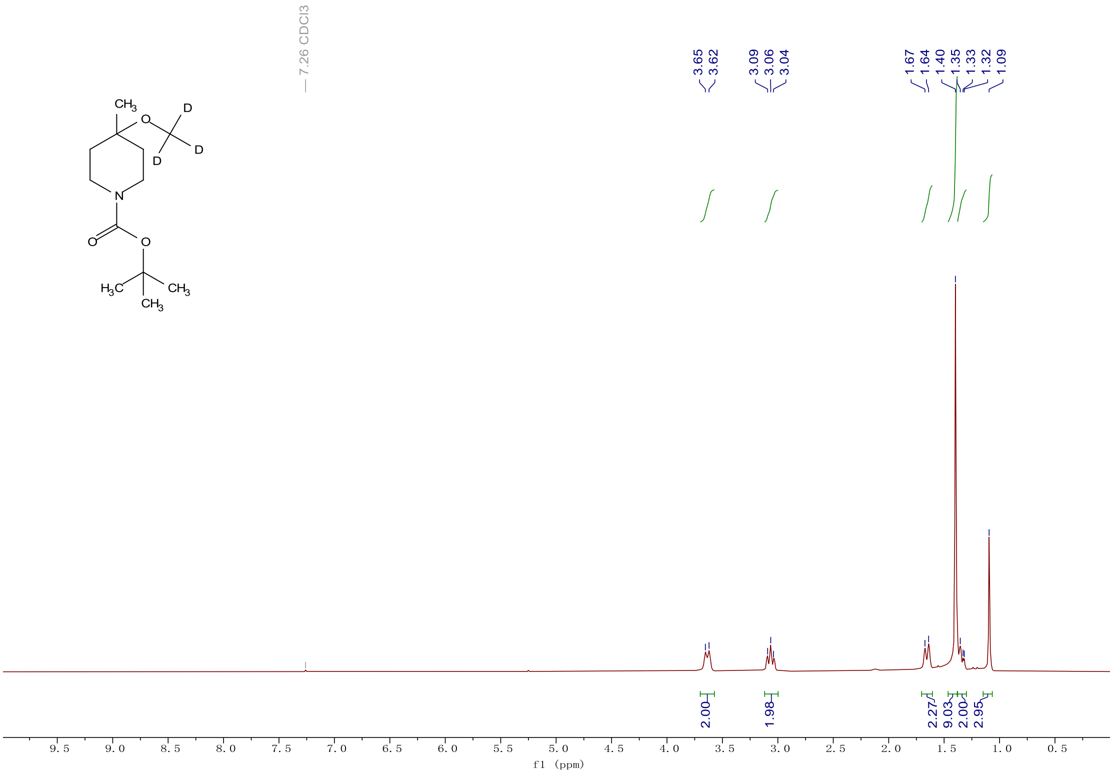

^{1}H NMR (400 MHz, $CDCl_3$) of **11j**

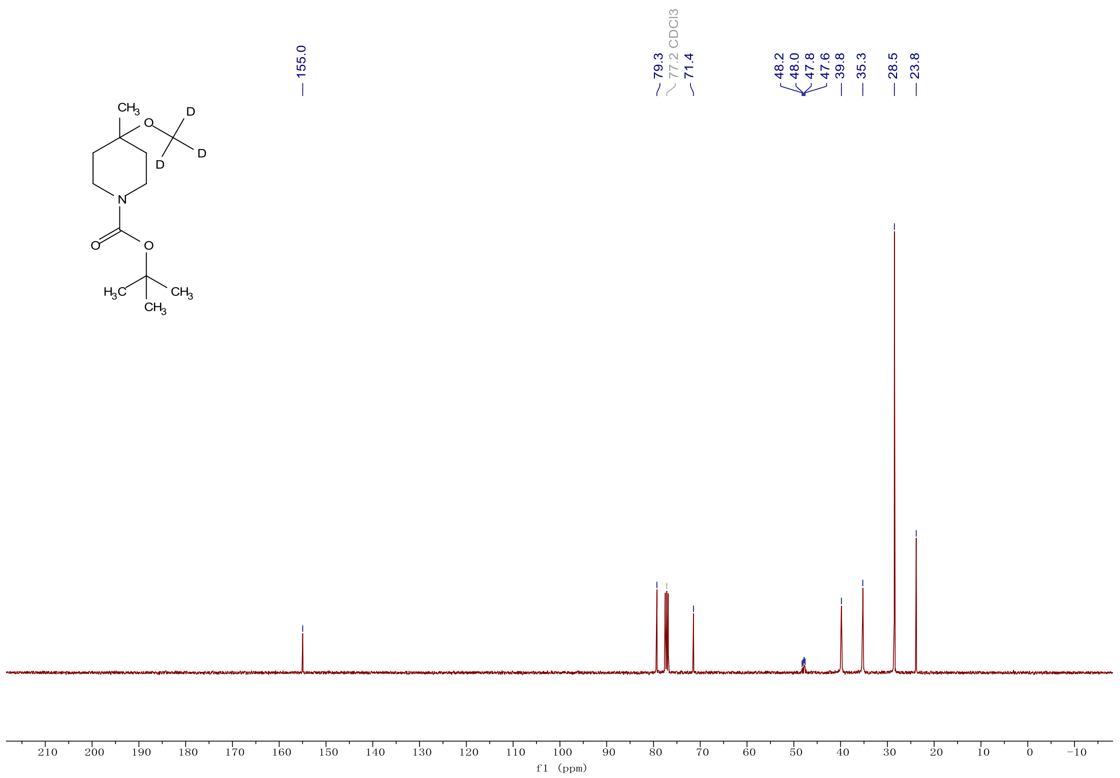

^{13}C NMR (101 MHz, $CDCl_3$) of **11j**

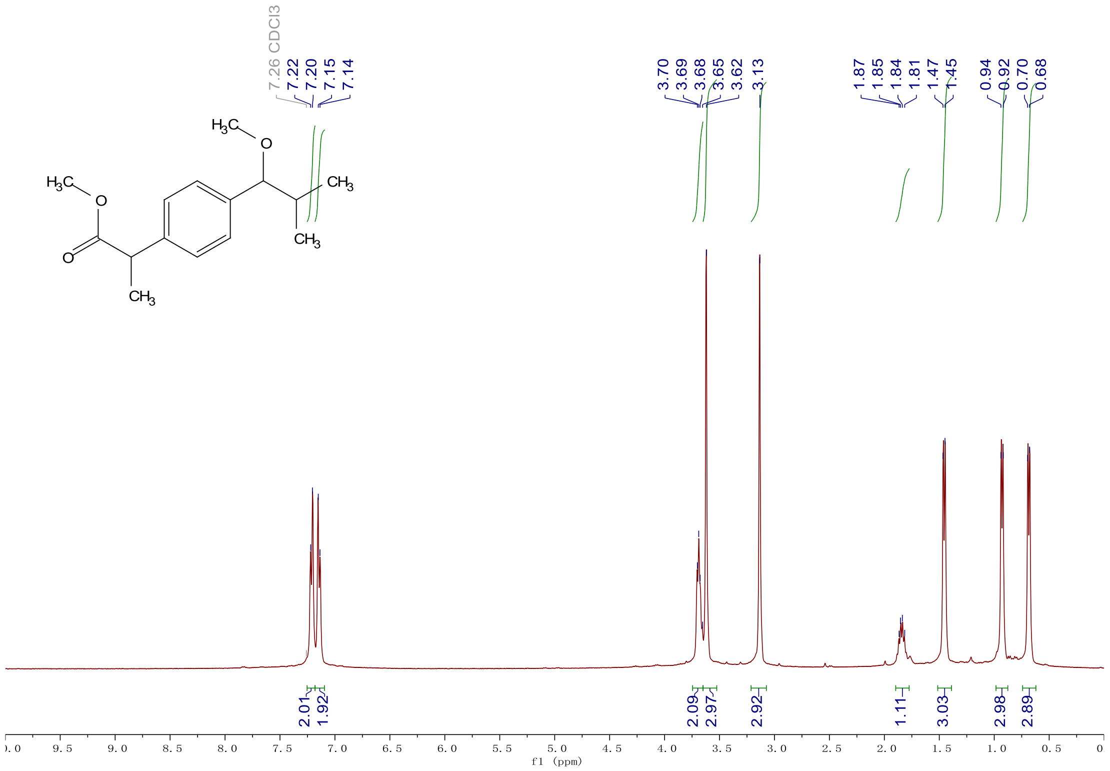

^{1}H NMR (400 MHz, $CDCl_3$) of **12a**

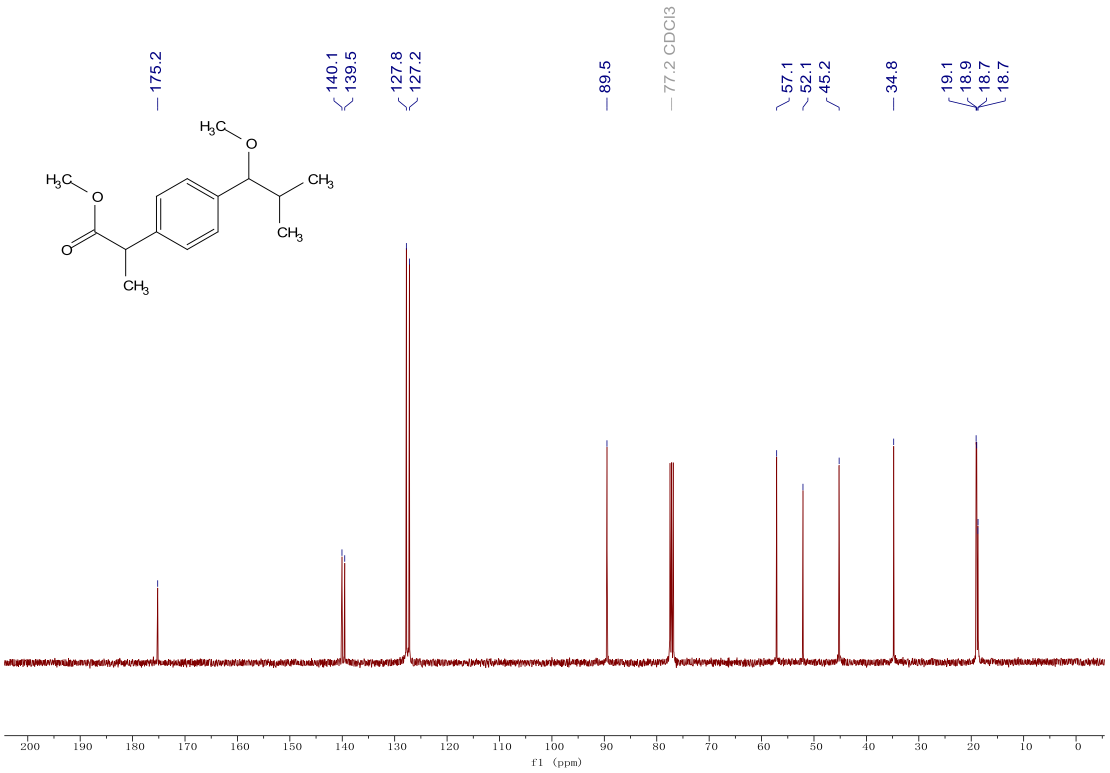

^{13}C NMR (101 MHz, $CDCl_3$) of **12a**

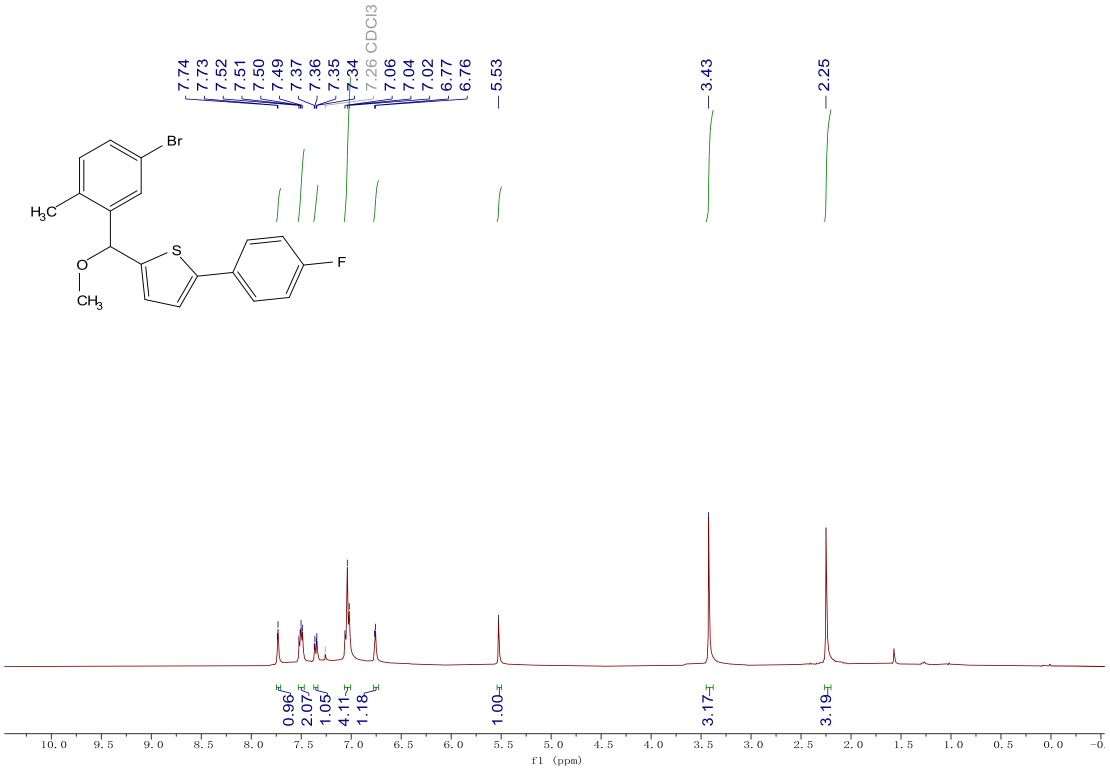

^{1}H NMR (400 MHz, CDCl$_3$) of **12b**

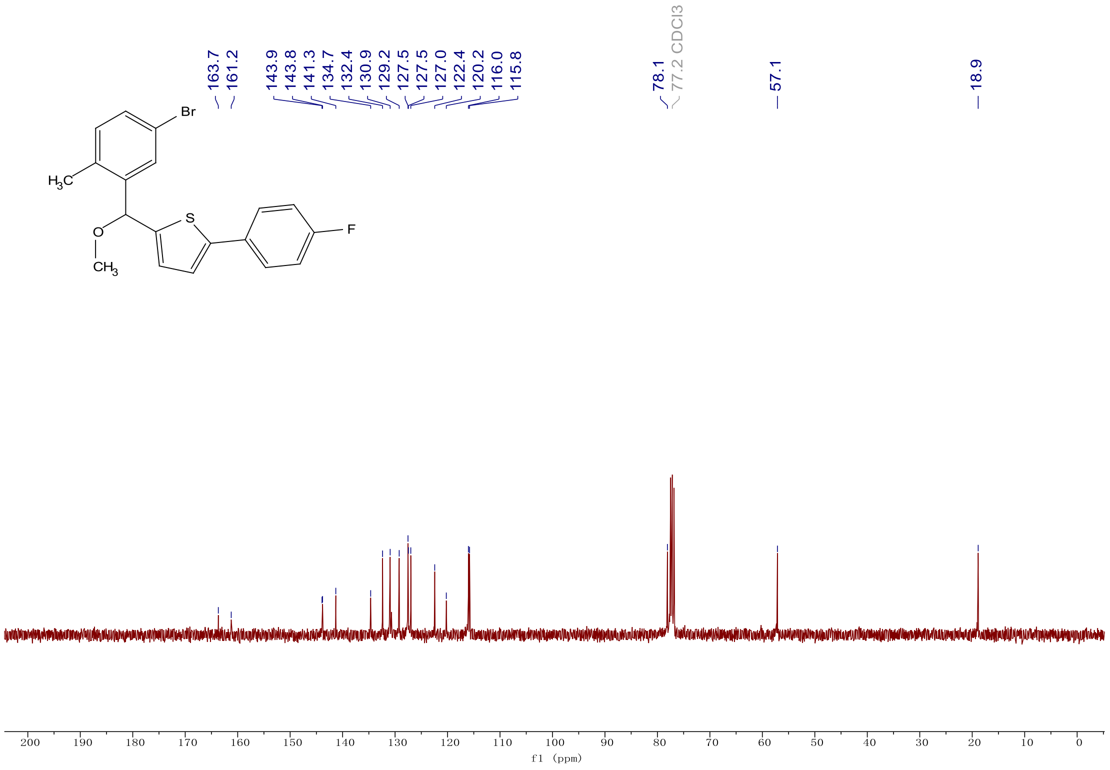

^{13}C NMR (101 MHz, CDCl$_3$) of **12b**

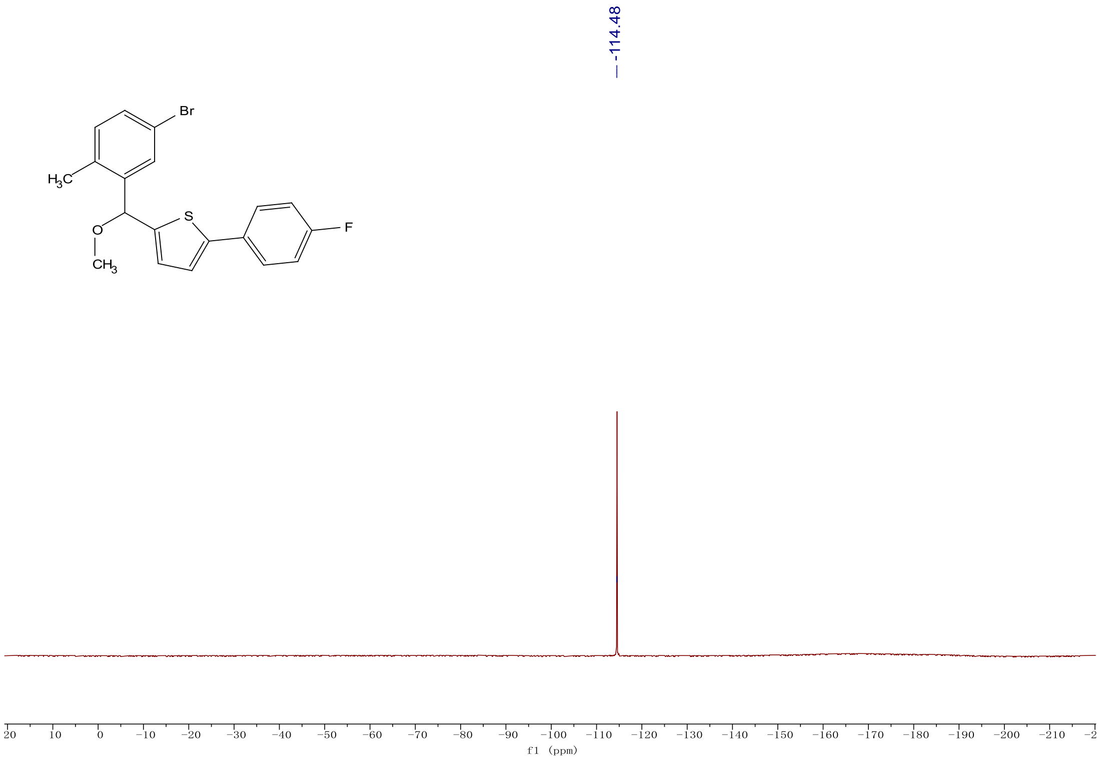

^{19}F NMR (376 MHz, $CDCl_3$) of **11b**

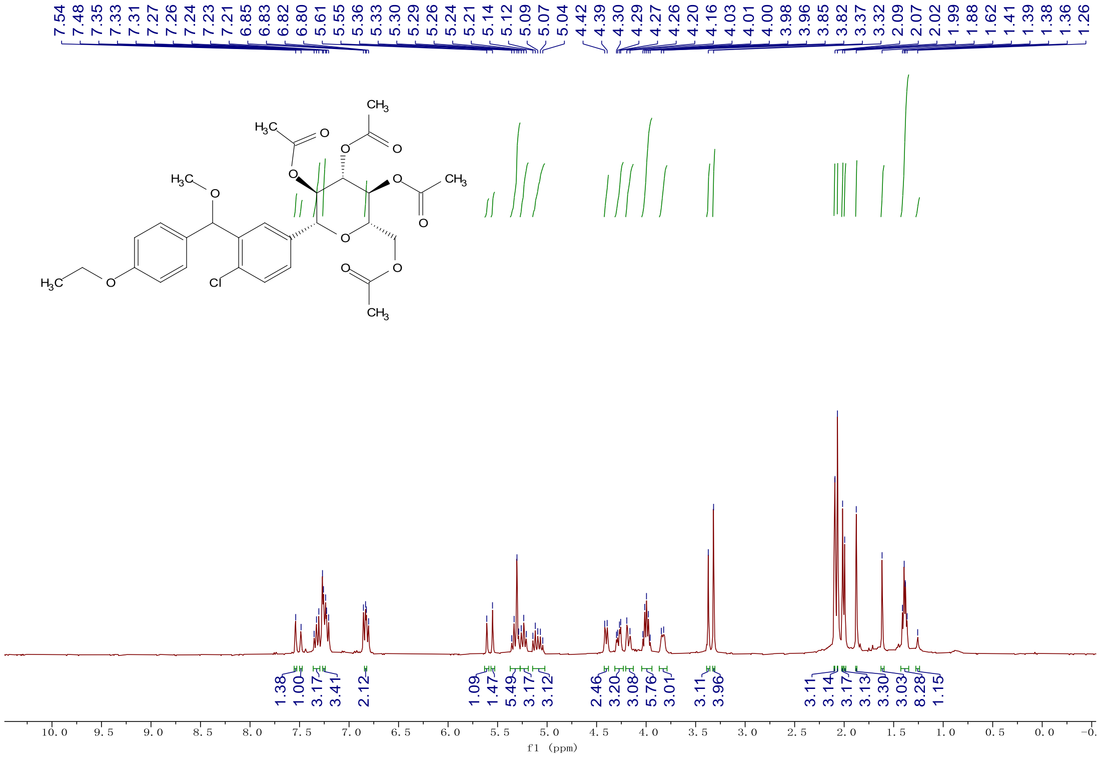

^{1}H NMR (400 MHz, $CDCl_3$) of **12c**

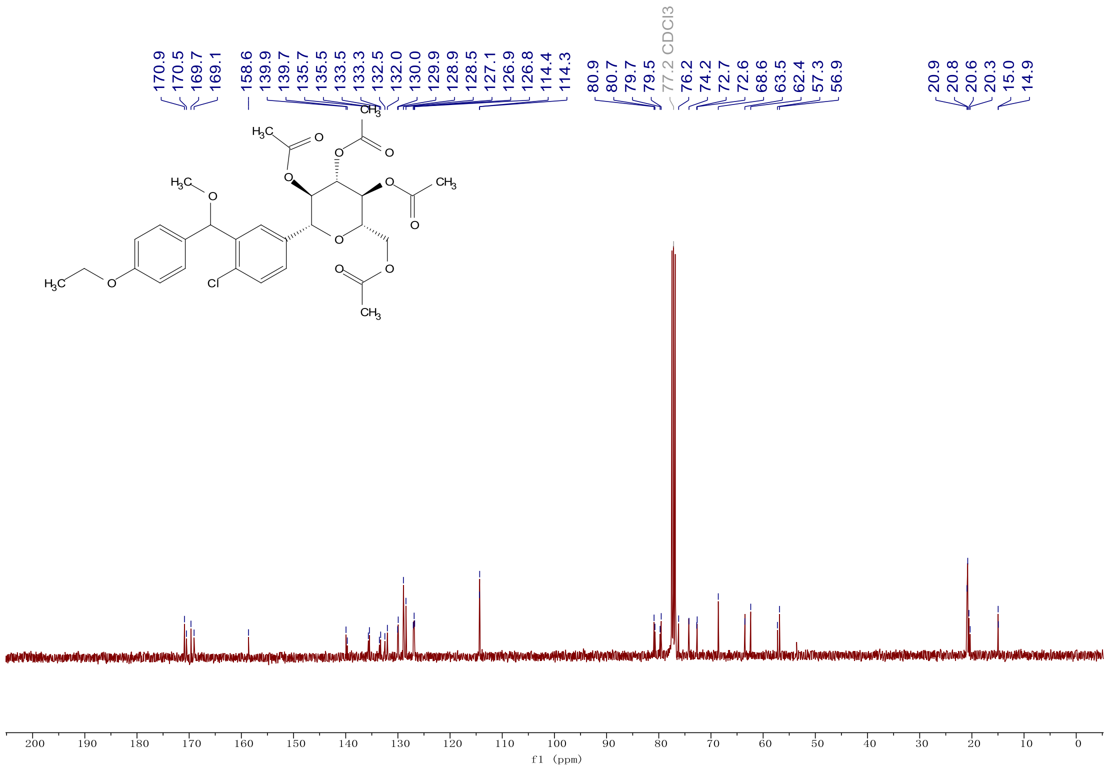

^{13}C NMR (101 MHz, CDCl$_3$) of **12c**

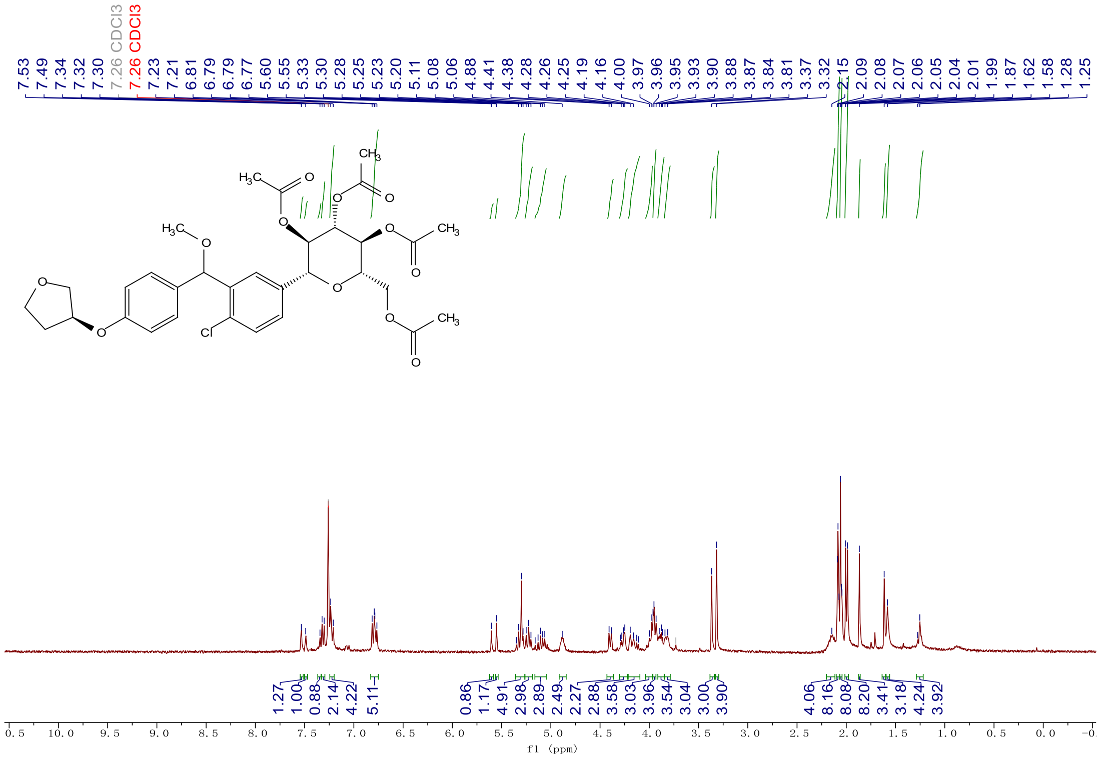

^{1}H NMR (400 MHz, CDCl$_3$) of **12d**

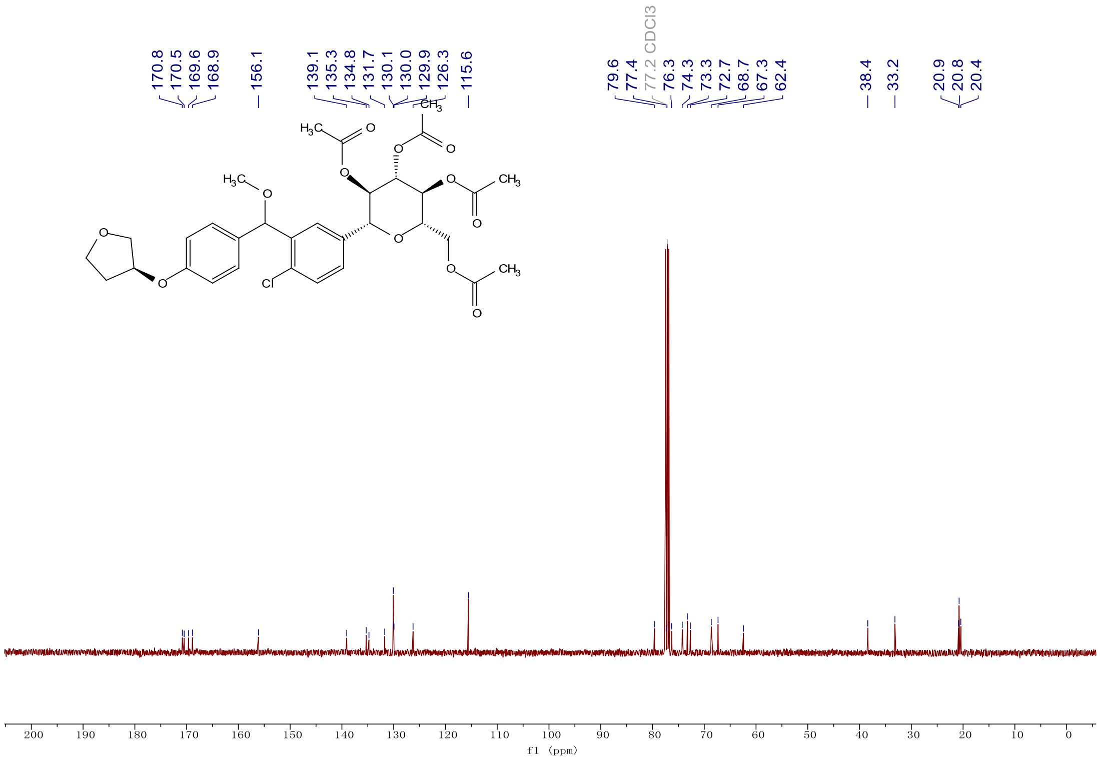

^{13}C NMR (101 MHz, $CDCl_3$) of **12d**

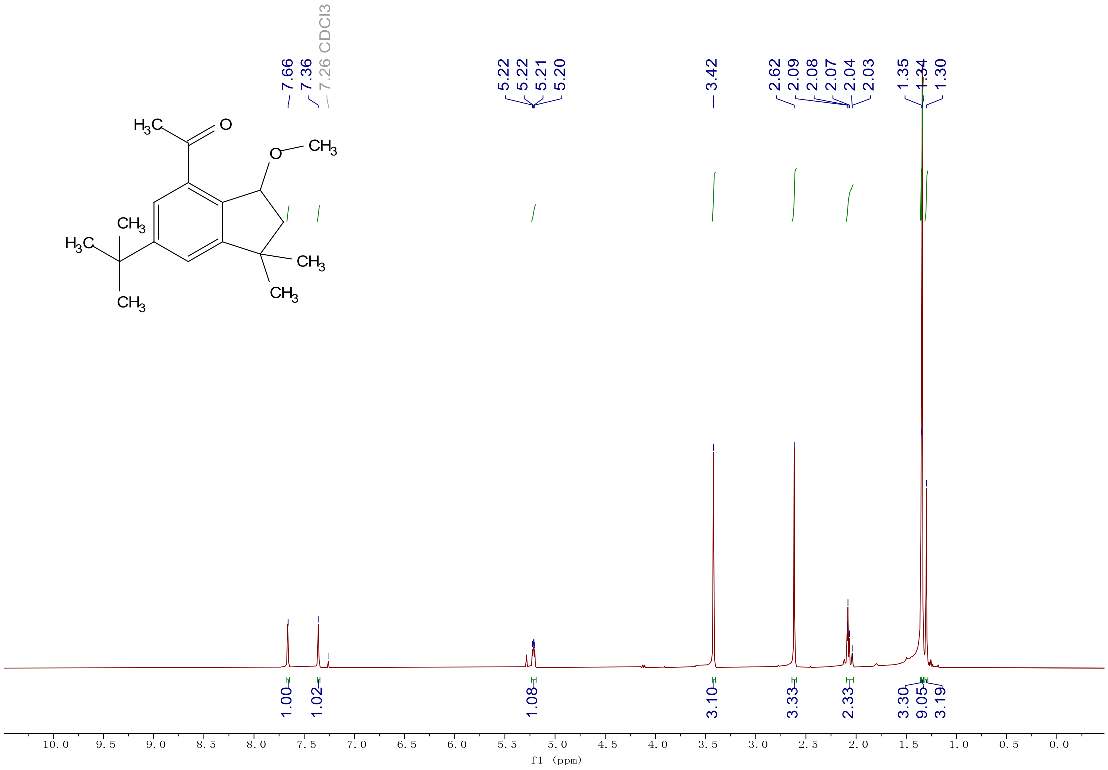

^{1}H NMR (400 MHz, $CDCl_3$) of **12e**

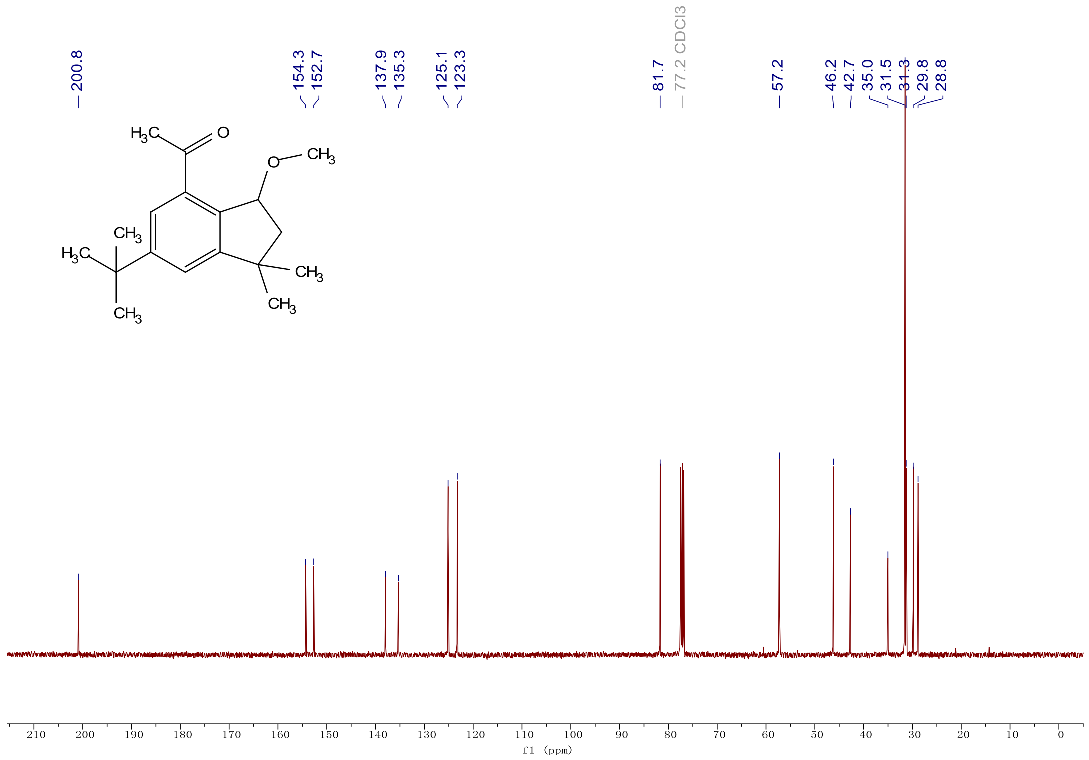

^{13}C NMR (101 MHz, $CDCl_3$) of **12e**

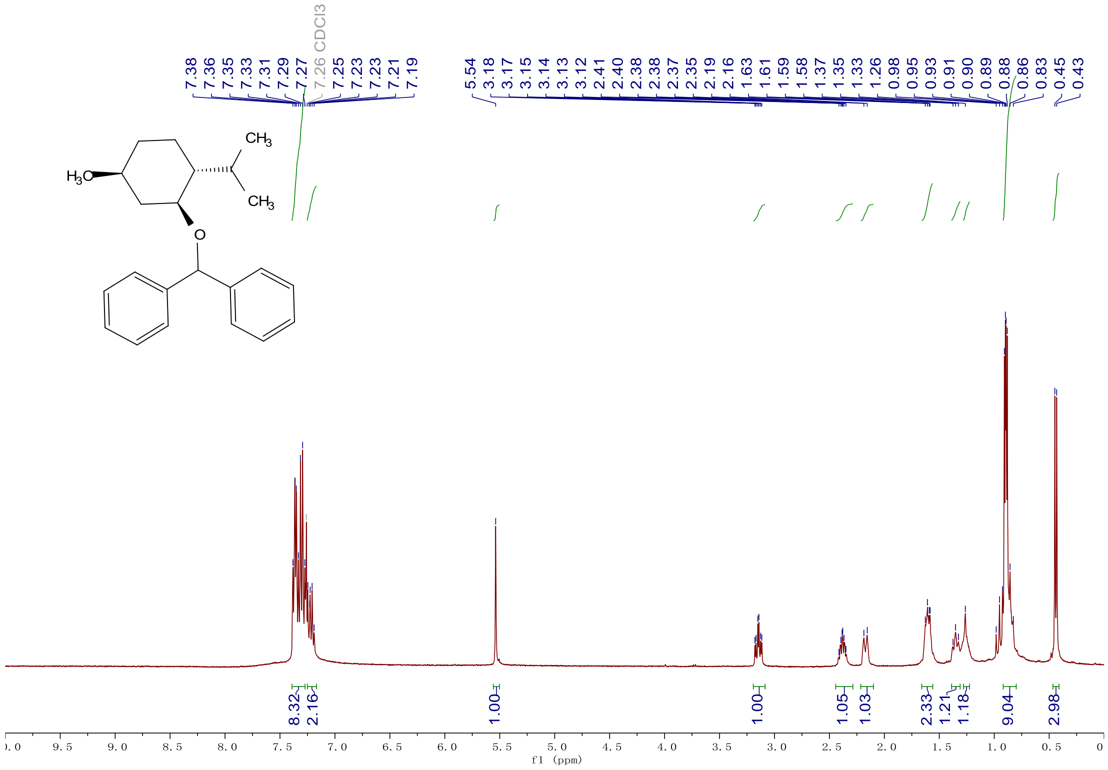

^{1}H NMR (400 MHz, $CDCl_3$) of **12f**

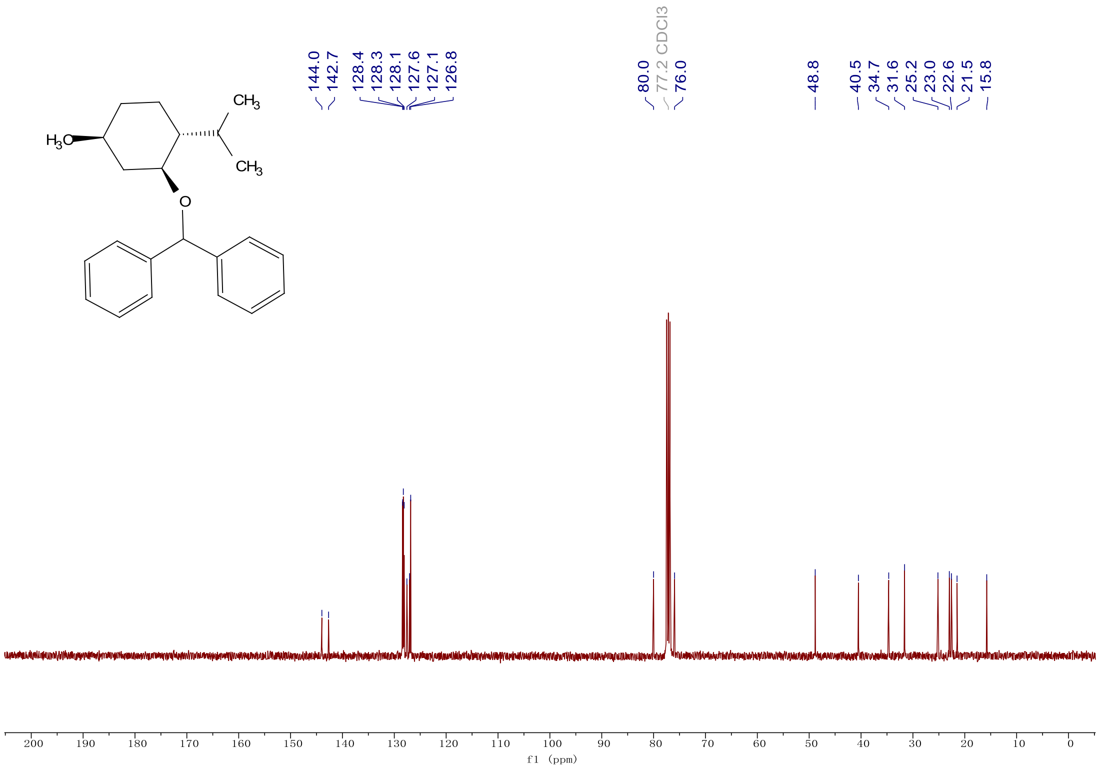

^{13}C NMR (101 MHz, $CDCl_3$) of **12f**

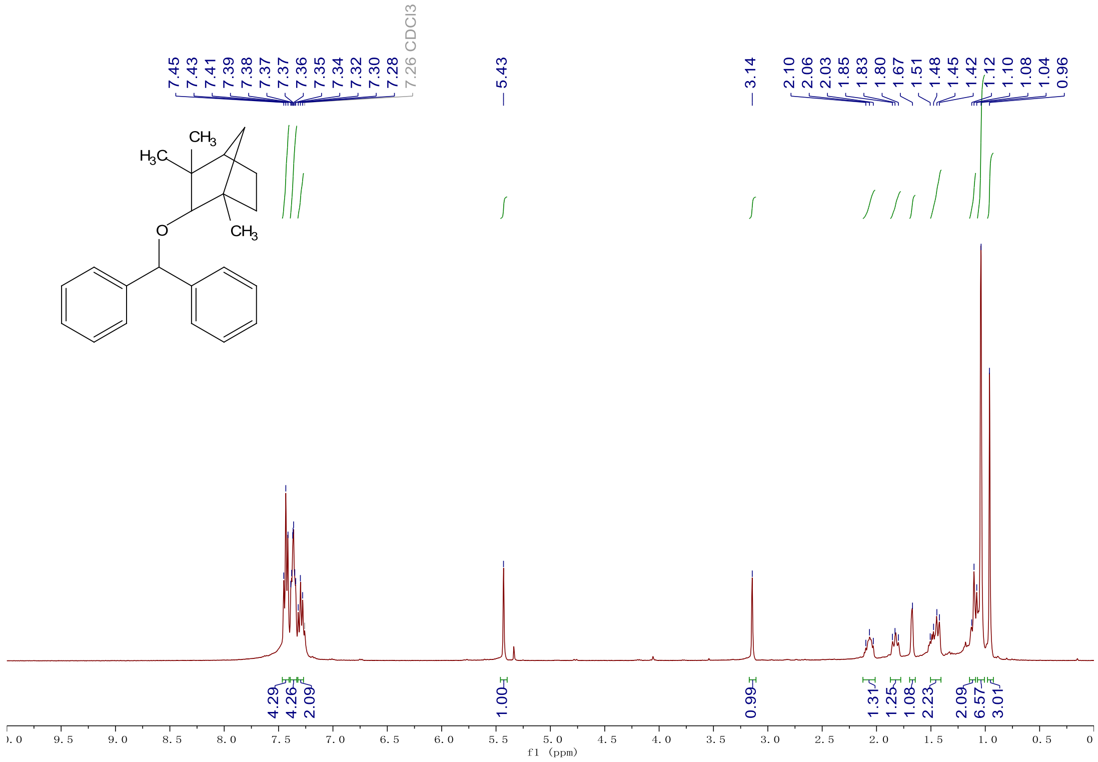

^{1}H NMR (400 MHz, $CDCl_3$) of **12g**

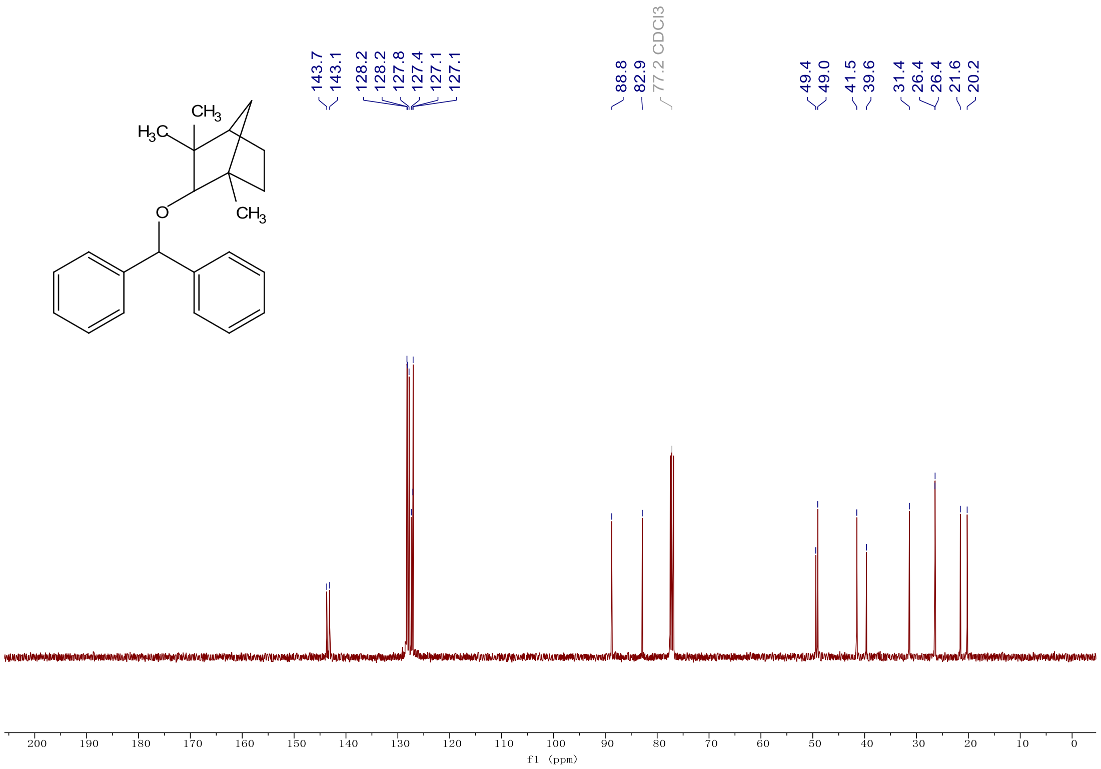

^{13}C NMR (101 MHz, $CDCl_3$) of **12g**

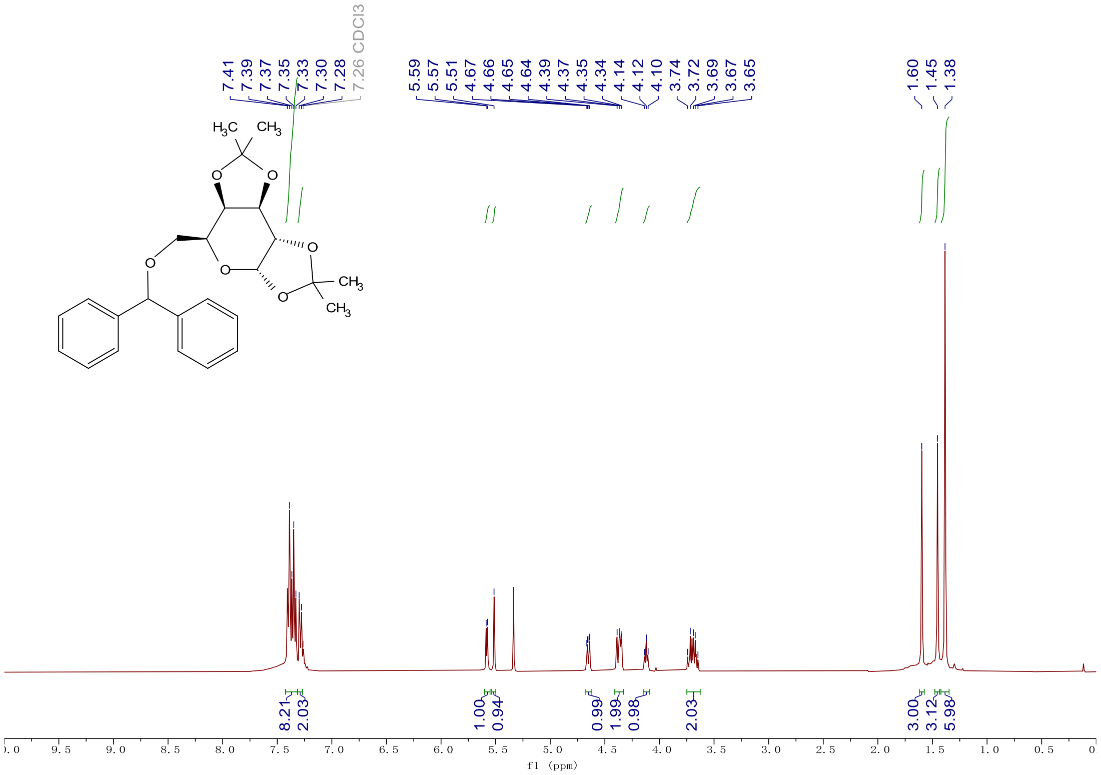

^{1}H NMR (400 MHz, $CDCl_3$) of **12h**

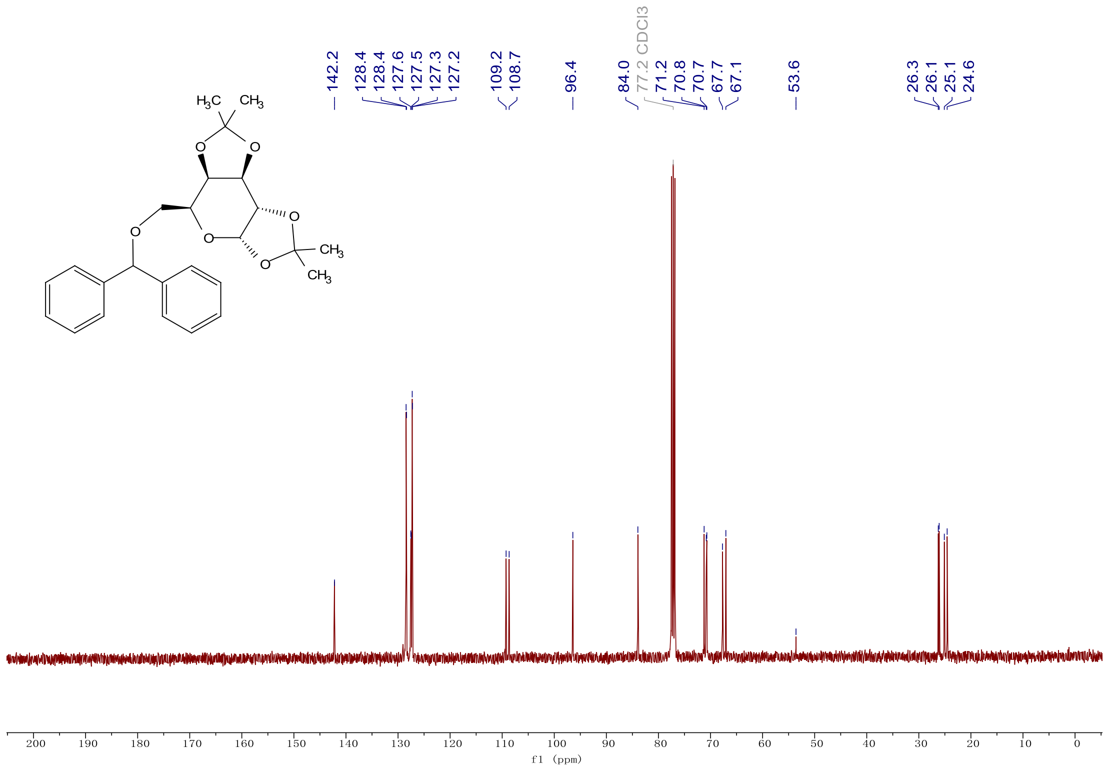

^{13}C NMR (101 MHz, $CDCl_3$) of **12h**

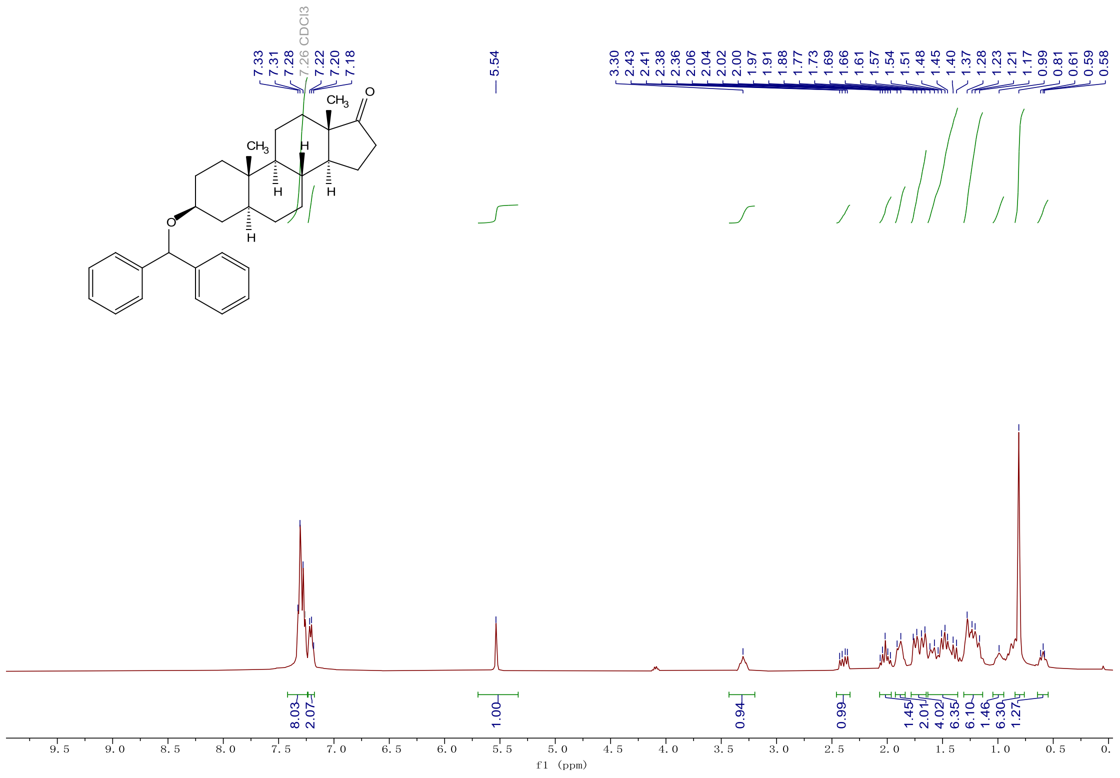

^{1}H NMR (400 MHz, $CDCl_3$) of **12i**

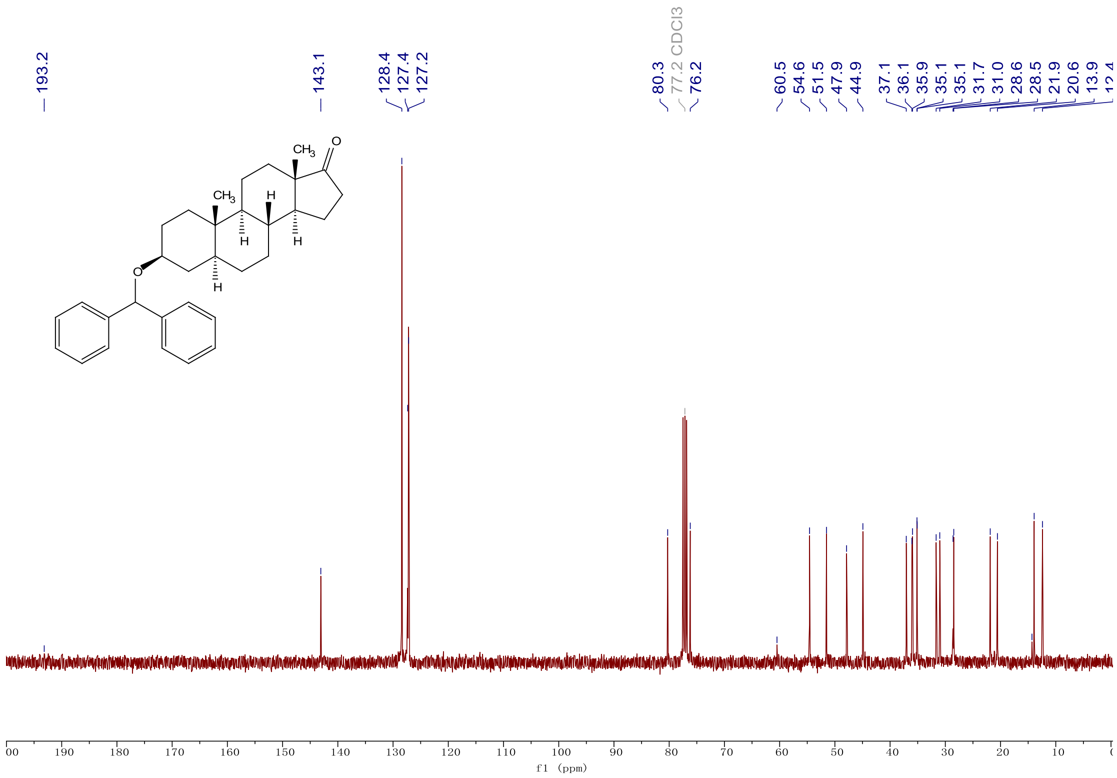

^{13}C NMR (101 MHz, $CDCl_3$) of **12i**
